# Computational Tools in the Discovery of FABP4 Ligands: A Statistical and Molecular Modeling Approach †

**DOI:** 10.3390/md17110624

**Published:** 2019-10-31

**Authors:** Giuseppe Floresta, Davide Gentile, Giancarlo Perrini, Vincenzo Patamia, Antonio Rescifina

**Affiliations:** 1 Department of Drug Sciences, University of Catania, V.le A. Doria, 95125 Catania, Italy; davide.gentile@unict.it (D.G.); vincenzo.patamia@unict.it (V.P.); 2 Department of Chemical Sciences, University of Catania, V.le A. Doria, 95125 Catania, Italy; gperrini@unict.it; 3 Consorzio Interuniversitario Nazionale di ricerca in Metodologie e Processi Innovativi di Sintesi (C.I.N.M.P.S.), Via E. Orabona, 4, 70125 Bari, Italy

**Keywords:** FABP4, A-FABP, aP2, antidiabetes, antiobesity, antiatherosclerosis, anticancer, computational tools, computer-aided drug discovery

## Abstract

Small molecule inhibitors of adipocyte fatty-acid binding protein 4 (FABP4) have received interest following the recent publication of their pharmacologically beneficial effects. Recently, it was revealed that FABP4 is an attractive molecular target for the treatment of type 2 diabetes, other metabolic diseases, and some type of cancers. In past years, hundreds of effective FABP4 inhibitors have been synthesized and discovered, but, unfortunately, none have reached the clinical research phase. The field of computer-aided drug design seems to be promising and useful for the identification of FABP4 inhibitors; hence, different structure- and ligand-based computational approaches have been used for their identification. In this paper, we searched for new potentially active FABP4 ligands in the Marine Natural Products (MNP) database. We retrieved 14,492 compounds from this database and filtered through them with a statistical and computational filter. Seven compounds were suggested by our methodology to possess a potential inhibitory activity upon FABP4 in the range of 97–331 nM. ADMET property prediction was performed to validate the hypothesis of the interaction with the intended target and to assess the drug-likeness of these derivatives. From these analyses, three molecules that are excellent candidates for becoming new drugs were found.

## 1. Introduction

Fatty acids (FAs) are a class of carboxylic acids that have many functions of vital importance in humans [1]. It was recently reported that elevated levels of FAs in plasma lead to specific physiological disorders [2], among these is type 2 diabetes [3], obesity [4] and atherosclerosis [5]. FAs have poor solubility in water, and, to overcome this problem, they are always associated with carrier proteins to facilitate trafficking in aqueous environments. Some examples of these carrier proteins are albumin, lipocalins, and fatty acid-binding proteins (FABPs) [6].

The adipocyte FABP (also called A-FABP, aP2, or FABP4) is a highly expressed FABP in adipocytes. Its levels are regulated by peroxisome-proliferator-activated receptor-c agonists, as well as by the levels of insulin and free FAs [7]. Studies conducted in FABP4 knockout mice have shown that this carrier protein has a crucial role in many aspects of the metabolic syndrome [8,9], with a potential role in future clinical treatments of this disorder. Indeed, the lack of a gene that codifies FABP4 partially prevents the advancement of insulin resistance and obesity in mice. Thus, small molecules that inhibit the physiological function of FABP4 can mimic the phenotype of FABP4-deficient mice, and might be useful candidates for the treatment of metabolic syndromes. It was also reported that FABP4 is highly expressed in macrophages [10]. Macrophages are an essential site of FABP action, and total or macrophage-specific FABP4-deficiency leads to a marked defense against early and advanced atherosclerosis [11].

The family of FABP proteins also has a significant role in cancer cells and cancer progression [12]. Up to now, modified FABP expression was described in different types of cancers, such as prostate, bladder, renal cell carcinoma, and other types of cancer cells [13,14,15]. Despite this, the biological functions of FABPs in cancer remain mostly unclear [16].

**Figure 1:**
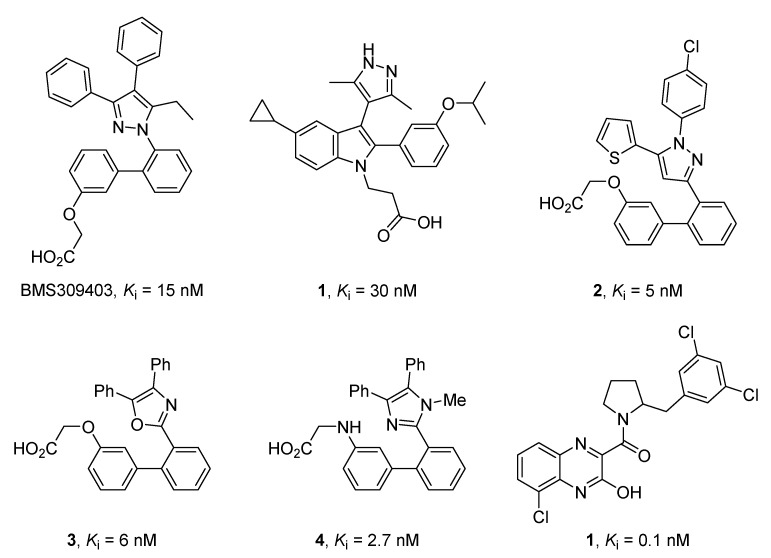
Structures of selected potent FABP4 inhibitors belonging to various chemical classes.

Recently, a variety of effective FABP4 inhibitors have been developed [17], but, unfortunately, none of them are currently in the clinical research phase (Figure 1).

Computer-aided drug design shows a promising and useful tool for the identification of novel molecules able to bind FAPB4. Notably, different structure-based computational approaches (docking-based virtual screening studies) have already been performed in this context with different libraries of compounds, leading to important results [18,19].

In line with our recent interest in the development of QSAR models and related applications [20,21,22,23,24,25,26,27], we recently produced the first 3D-QSAR model for the description of a dataset of selective and potent FAP4 inhibitors [28,29]. The 3D-QSAR model was then combined with a scaffold-hopping analysis, allowing the design of new potent molecules able to interact with the binding site and inhibit FABP4. Finally, three of the ligands suggested by the scaffold-hopping analysis were synthesized and tested in vitro, yielding IC_50_ values between 3.70 and 5.59 M.

Given the excellent result in identifying novel structures and in assisting in the design of novel FABP4 binders with this 3D-QSAR model, in this work, we decided to combine the ligand-based approach with a structure-based one (docking) to screen a large dataset of marine products for the identification of novel hit-compounds among the marine word. This purpose was pursued using a statistical and computational approach, as already successfully reported, for the identification of sigma-2 receptor ligands [30] and heme oxygenase 1 inhibitors [31].

## 2. Results

### 2.1. Design and Application of the Three Filters Used for the MNP Database Screening

**Figure 2:**
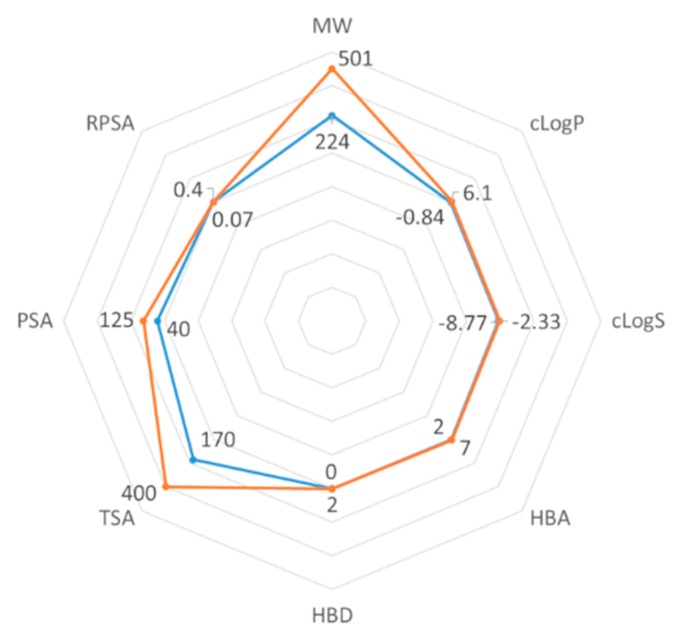
Radar plot representation of the range values (minimum in blue and maximum in orange) of the eight selected descriptors associated with the 120 FABP4 inhibitors.

The first filter used for the identification of FABP4 ligands was a statistical (based on 2D and 3D descriptors) one, as already used successfully by us [31]. We selected 2922 molecules among the MNP database by a statistical/2D descriptors filter using DataWarrior software [32]. The appropriate range of values to be considered for each chosen descriptor was obtained by analyzing the most potent and selective compounds present in a recently published dataset of FABP4 ligands, giving a total of 120 entities [28,29]. Therefore, the ranges for molecular weight (MW, 224/501), cLogP (−0.84/6.1), cLogS (−8.77/−2.33), H-bond-acceptors (HBA, 2/7), H-bond-donors (HBD,0/2), total surface area (TSA, 170/400), polar surface area (PSA, 40/125), and relative polar surface area (RPSA, 0.07/0.4) (Figure 2), belonging to the 120 potent and selective FABP4 inhibitors, were associated with each descriptor and applied to the dataset of 14,492 MNP molecules to give 2922 marine filtered compounds.

These skimmed molecules were then subjected to a second filtration using a mixed ligand- and structure-based approach. Firstly, the 3D molecular structures of the 2922 marine compounds were aligned to our previous published 3D-QSAR model for the FABP4 protein, and the compounds were then evaluated, as previously reported, employing Forge software (v10.4.2, Cresset, New Cambridge House, United Kingdom) [33]. Over the whole dataset of the first filtered marine natural products, 1854 molecules resulted in an excellent or good description by the model. This means that most of the features in the evaluated molecules were well described by the training set of the 3D-QSAR model, and the predicted activity could be considered reliable. Among these compounds, 198 molecules resulted in a predicted pIC_50_ activity between 6.0 and 7.6. The 3D molecular structures of the 2922 marine compounds were then passed to the structure-based approach, adapting the docking procedure already reported for the identification of FABP4 inhibitors [34,35]. The AutoDock software (v. 4.2.6, Molecular Graphics Lab at The Scripps Research Institute, La Jolla, CA, USA) [36] was used for all docking studies. The validation of the adopted docking procedure was assessed by using linear regression analysis upon a benchmark data set of 34 known FABP4 inhibitors (Table S1, Figure S1).

**Figure 3:**
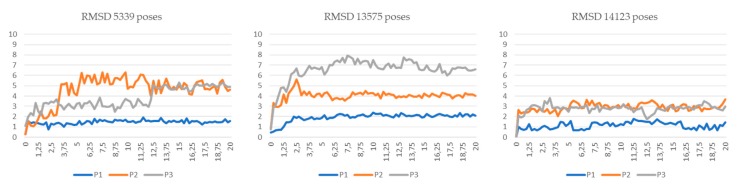
MD simulations of the three selected poses P1–P3 for each of the three selected ligands 5339, 13575, and 14123.

All of the generated binding poses were manually inspected in order to ensure correct positioning within the binding pocket with respect to the interactions of ligand moieties with the amino acid residues relevant for the catalytic activity. The residues Phe19, Met20, Ala33, Pro38, Lys58, Phe57, Ala75, Glu72, Arg106, and Arg126 play an important role in the interactions of FABP4 with inhibitors [37,38]. Initially, these residues were used as a filter to discard the incorrect poses derived from the docking. In addition, molecular dynamics (MD) simulation studies of three of the most promising compounds (Table 1, 5339, 14123, and 13575) were conducted to verify the effectiveness of the poses selected. In particular, for each selected ligand, we performed three 20 ns MD simulations using three different poses, named P1–P3, where the P1 ones are those that visually satisfy the aforementioned reported key interactions, whereas the others two (P2 and P3) lack some of them. The results reported in Figure 3 as root-mean-square deviation (RMSD) fluctuations of the ligand coordinates clearly highlight the two unfavorable poses, i.e., those with higher RMSD fluctuations [39]. These present even less persistence of the hydrogen bond interactions with the key residues. On the contrary, the best poses (P1) show very low RMSD fluctuation.

In Figure S2, it is possible to note that the compound 5339 forms a hydrogen bond with the residue Arg106, *π*-*π* stack interactions with the residue Pro38, and hydrophobic interactions with the residues Phe16, Ala 33, Ala36, Phe57, Ile62, and Ala75 with aromatic regions of the ligand. Compound 13575 establishes hydrogen bonds with the residues Tyr19 and Ala75, and the hydrophobic region of the molecule establishes hydrophobic interactions with the residues Phe16, Ala33, Pro38, Phe57, and Ile 62 (Figure S3). Finally, compound 14123 shows a hydrogen bond with the residue Arg106, while other hydrophobic interactions with Phe16, Met20, Ala 33, Pro38, Ala40, Ala, Ile 62, and Ala75 reinforce the bond with the hydrophobic region of FABP4 (Figure S4).

Re-docking experiments conducted after 20 ns of MD simulation of P1 poses gave a calculated p*K*_i_ value of 7.92, 7.96, and 7.96 for the ligands 5339, 13575, and 14123, respectively, which are slightly better than those calculated on the crystallographic laying of the FABP4 receptor, according to a correct arrangement of the ligand in the catalytic pocket.

The 3D-QSAR and docking evaluation results are reported in Table S2.

### 2.2. Merged Ligand- and Structure-Based Filters

The results derived from the ligand-based calculation (i.e., 3D-QSAR evaluation) and the structure-based calculation (i.e., docking calculations) were then merged with the aim to create a final filter. For this purpose, the best (lowest calculated IC_50_ or *K*_i_) 2% and 5% of the molecules obtained from each of the two approaches were retrieved, and those simultaneously present in both filters were selected (Table 1). The 2% filter resulted in only one molecule (5339), whereas the 5% one returned six other molecules (14123, 13575, 7846, 3164, 2076 and 1534).

**Figure 4:**
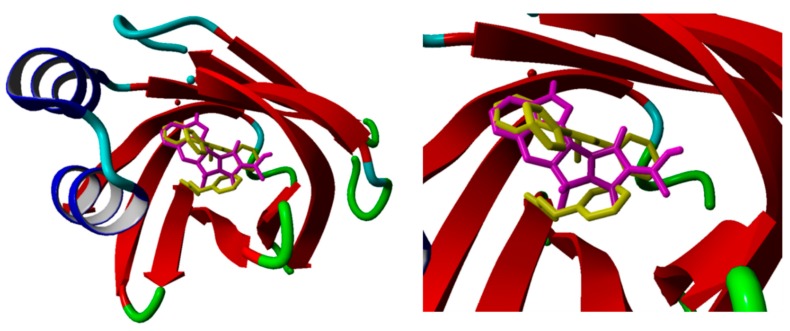
Best-docked pose of compound 5339 (magenta) superposed with the co-crystallized structure of the BMS309403 (yellow) FABP4 inhibitor in the binding pocket of the enzyme (PDB ID: 2NNQ) (right). Zoom of the catalytic pocket (left).

All the binding poses retrieved from the molecular docking calculations of the seven compounds reported in Table 1, which showed the classical interactions of the most common FABP4 ligands, are reported in the Supplementary Material (Figures S2–S8). In particular, the best-docked pose of compound 5339, chosen as representative, superposed with the co-crystallized structure of the BMS309403 FABP4 inhibitor in the binding pocket of the enzyme shows that the two compounds are partially overlapped and occupy almost the totality of the catalytic orthosteric site (Figure 4).

Among the seven filtered best potential inhibitors, molecule 5339 (indole alkaloid) has been reported as an inhibitor of the Ca^2+^-ATPase of the sarcoendoplasmic reticulum (SERCA) [40]. Compound 14123 (steroid) was identified as cytotoxic and an anti-tumor agent [41]. Compound 13575 (diterpene) was tested for its cytotoxicity against several tumor cells, but it lacked any activity [42]. Compound 7846 is a cembrane diterpenoid; similar compounds were reported to exert growth-inhibition effects toward tumor cells [43]. Compound 3164 (pentacyclic hydroquinone) was reported as cytotoxic by acting on DNA topoisomerase I [44]. Compound 2076 (alkaloid) was reported as cytotoxic against several tumor cell lines [45]. Compound 1534 (sesquiterpene) has anti-inflammatory activity by acting as a phospholipase A2 (PLA2) inhibitor [46]. Interestingly, PLA2 catalyzes the hydrolysis of phospholipids to produce free fatty acids. The fatty acid is the substrate for the biosynthesis of eicosanoids, which are known to mediate inflammation. Based on this mode of action, compounds that inhibit PLA2 activity have been targeted as potential therapeutic agents in the treatment of inflammation. The association between PLA2 and FABP4 in the regulation of inflammatory responses has already been proven [47], and dual inhibition of such proteins would be advantageous in inflammation treatment. Moreover, compound 1534 is a sesquiterpene, and this class of natural products, together with steroids, diterpenes, diterpenoids, quinones, and alkaloids, has already been identified as a candidate for the inhibition of FABP4 [48].

### 2.3. ADMET Properties

As the interaction of an inhibitor with an enzyme cannot guarantee its suitability as a drug, to further strengthen the results of 3D-QSAR and docking studies, we also performed in silico ADMET studies on the seven molecules reported in Table 1. The ability to reach targets in bioactive form was assessed using the SwissADME (http://swissadme.ch) and pkCSM (http://biosig.unimelb.edu.au/pkcsm/) web platforms. Importantly, the technologies implemented in these platforms are able to predict, with a fair degree of certainty, the false-positive results commonly observed in biochemical assays of small molecules [49].

**Figure 5:**
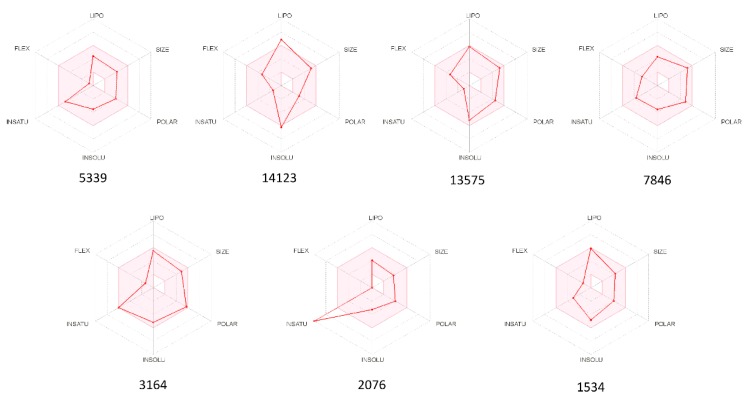
Radar plots of the six drug-likeness parameters used to predict the oral bioavailability of the seven investigated compounds. The colored zone is a suitable physicochemical space for oral bioavailability. LIPO (Lipophility): −0.7 < XLOGP3 < 5.0; SIZE: 150 g/mol < MW < 500 g/mol; POLAR (Polarity): 20 Å^2^ < TPSA < 130 Å^2^; INSOLU (Insolubility): 0 < Log *S* (ESOL) < 6; INSATU (Insaturation): 0.25 < Fraction Csp^3^ < 1; FLEX (Flexibility): 0 < Num. rotatable bonds < 9. All results have been obtained from the SwissADMET web server [50].

The oral availability of our proposed bioactive compounds is shown in the bioavailability radar plots (Figure 5), which provide a graphical snapshot of the drug-likeness parameters of the investigated molecule. Notably, five compounds (5339, 13575, 7846, 3164, and 1534) have been predicted as orally bioavailable, whereas compounds 14123 and 2076 present only one off-shoot relative to the lipophilicity (LIPO) and unsaturation (INSATU) vertexes, respectively, leading to suboptimal physicochemical properties for their oral bioavailability.

In addition to the Lipinski rule of five [51], another four drug-likeness rules named Ghose [52], Egan [53], Veber [54], and Muegee [55], were contemporarily satisfied by six compounds with the exception of molecule 14123 (Table 2). Instead, the stringent lead-like criteria of Teague [56] were passed by compounds 5339 and 2076. As lead-likeness tests are intended to provide leads with high affinity in high-throughput screens that allow for the discovery and exploitation of additional interactions in the lead-optimization phase, molecules 5339 and 2076 are excellent candidates for investigation based on the scaffold hopping approach.

Finally, the outcome of the pan assay interference structures (PAINS) model [57], conceived to exclude small molecules that are likely to show false positives in biological assays, post only one alert for compound 1534, concerning the presence of a quinone moiety.

**Figure 6:**
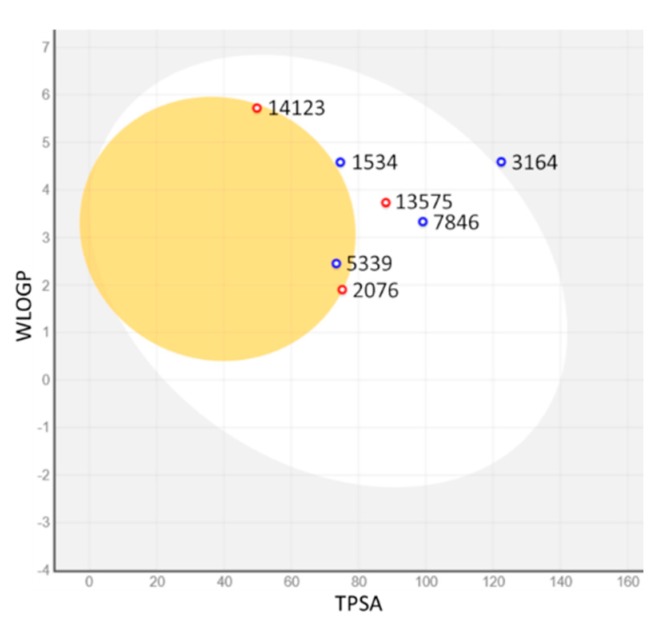
BOILED-Egg plot. Points located in the BOILED-Egg’s yolk (yellow) represent the molecules predicted to passively permeate through the blood–brain barrier (BBB), whereas the ones in the egg white are relative to the molecules predicted to be passively absorbed by the gastrointestinal tract; the blue dots indicate the molecules for which it was expected to be effluated from the central nervous system (CNS) by the P-glycoprotein, whereas the red ones point to the molecules predicted not to be effluated from the CNS by the P-glycoprotein.

Human gastrointestinal absorption (HIA) and blood–brain barrier penetration (BBB), relative to the absorption and distribution parameters, respectively, have been graphically represented by the extended and renewed version of the Edan–Egg model, named the Brain or IntestinaL EstimateD (BOILED) permeation predictive model (BOILED-Egg). The visual analysis of Figure 6 highlights that all investigated molecules, with the exception of 3164, were predicted to be passively absorbed by the gastrointestinal tract, and three of them, 5339, 14123, and 2076, passively permeate through the BBB, the first with the aid of the P-glycoprotein and the other two without it. These data are reflected in the values shown in Table 3.

Regarding the absorption parameters, compounds 5339, 14123, 13575, and 2076 present a promising oral availability, due to the optimal Caco-2 cell permeability and HIA (>0.9 and >90%, respectively, Table 3), and skin permeability (log*K*_p_ < −2.5, Table 3).

The volume of distribution (VD_ss_) and unbound fraction are two of the most important pharmacokinetic drug parameters. Values of the VD_ss_ > 0.45 indicate that the drug will be distributed in tissue, whereas values < −0.15 indicate that the drug will be distributed in plasma. So, VD_ss_ describes the extent of drug distribution, and the unbound fraction describes the portion of free drug in plasma that may extravasate. Except for compounds 3164, the other ones showed intermediate values of VD_ss_, and should have an adequate plasma distribution profile, with a fraction of the unbound drug between 0 and 0.157. These values indicate that the molecules can be well distributed and present a significant unbound fraction in the plasma, thus becoming available to interact with the pharmacological target. Only compound 3164 is entirely unable to penetrate the central nervous system (CNS).

The predicted values of the total clearance (Table 3), which measure the efficiency of the body in eliminating a drug, indicate that all compounds have a good renal elimination (1.5–8.4 mL/min/kg) and are not substrates of the renal organic cation transporter 2 (OCT2), with the exception of compound 13575. Finally, compounds 2076 and 14123 did not pass the AMES and Minnow toxicity tests, respectively, whereas all others did not present any particular toxicity problems.

The overall lecture of Table 3 highlights that compounds 5339, 13575, and 1534 could be excellent candidates as drugs, or could lead to further studies and manipulations.

## 3. Materials and Methods

### 3.1. Dataset of Compounds

The chemical structures of the marine dataset were retrieved from Marine Natural Products (MNP, http://docking.umh.es/). The full list of the 2922 molecules that passed the first statistical filter, including the MNP ID, SMILES, ligand-, and structural-based evaluation results, are available in the Supplementary Material (Table S1).

### 3.2. Structure Preparation and Minimization

The structures of all the molecules used in this study were built using Marvin Sketch (18.24, ChemAxon Ltd., Budapest, Hungary) [59]. A first molecular mechanics energy minimization was used for 3D structures created from the SMLES, and the Merck molecular force field (MMFF94) present in Marvin Sketch [59] was used. The protonation states were calculated assuming a neutral pH. The PM3 Hamiltonian, as implemented in the MOPAC package (MOPAC2016 v. 18.151, Stewart Computational Chemistry, Colorado Springs, CO, USA) [60,61,62], was then used to further optimize the 3D structures before the alignment for the 3D-QSAR filter and the docking calculations.

### 3.3. Compound Alignment for the 3D-Ligand Based Filter

The alignment and evaluation of the 2922 selected marine products were performed as follows. Firstly, the 3D structures of the molecules were imported into the software Forge (v10.4.2, Cresset, New Cambridge House, Hertfordshire, UK). The molecules were then aligned by a maximum common substructure algorithm using a customized and validated set-up [20,22,24], in the FABP4 3D-QSAR model already published by us [28,29]. Before the alignment, the filed points of each molecule were generated using the XED (extended electron distribution) force field in Forge. The conformational analysis was done using a maximum number of 500 conformers using a gradient cutoff for conformer minimization of 0.1 kcal/mol and a similarity threshold, below which two conformers are assumed identical, of 0.5 Å. The energy window was set to 2.5 kcal/mol, and all the conformers with calculated energy outside the selected energy window were discarded.

### 3.4. Molecular Docking

Flexible ligand docking experiments were performed by employing AutoDock 4.2.6 software implemented in YASARA (v. 19.5.5, YASARA Biosciences GmbH, Vienna, Austria) [63,64], using the three-dimensional crystal structure of substrate-free fatty acid-binding protein 4 in complex with BMS309403 (PDB ID: 2NNQ) obtained from the Protein Data Bank (PDB, http://www.rcsb.org/pdb), and the Lamarckian genetic algorithm (LGA). The maps were generated by the program AutoGrid (4.2.6) with a spacing of 0.375 Å and dimensions that encompass all atoms extending 5 Å from the surface of the structure of the crystallized ligands. All parameters were inserted at their default settings as previously reported [30]. In the docking tab, the macromolecule and ligand were selected, and GA parameters were set as ga_runs = 100, ga_pop_size = 150, ga_num_evals = 25,000,000, ga_num_generations = 27,000, ga_elitism = 1, ga_mutation_rate = 0.02, ga_crossover_rate = 0.8, ga_crossover_mode = two points, ga_cauchy_alpha = 0.0, ga_cauchy_beta = 1.0, number of generations for picking worst individual = 10.

All crystallographic water and SO_4_^2−^ buffer molecules and ions were removed. Linear regression analysis was used to validate the correspondence between the experimental p*K*_i_ values and the calculated ones from docking scores, utilizing a benchmark data set of 34 known FABP4 inhibitors possessing p*K*_i_ values in the range of 1–5000 nM, to be reliable, chosen from those reported in the reference [17] (Table S1).

### 3.5. Molecular Dynamics Simulations

The molecular dynamics simulations of the FABP4/ligand complexes were performed with the YASARA Structure package. A periodic simulation cell with boundaries extending 8 Å [65] from the surface of the complex was employed. The box was filled with water, with a maximum sum of all water bumps of 1.0 Å, and a density of 0.997 g mL^−1^ with explicit solvent. YASARA’s p*K*_a_ utility was used to assign p*K*_a_ values at pH 7.4 [66], and the cell was neutralized with NaCl (0.9% by mass); in these conditions. Water molecules were deleted to readjust the solvent density to 0.997 g/mL. The final system dimensions were approximately 40 × 40 × 40 Å^3^. The ligand force field parameters were generated with the AutoSMILES utility [67], which employs semiempirical AM1 geometry optimization and assignment of charges, followed by the assignment of the AM1BCC atom and bond types with refinement using the RESP charges, and finally the assignments of general AMBER force field atom types. Optimization of the hydrogen bond network of the various enzyme–ligand complexes was obtained using the method established by Hooft et al. [68], to address ambiguities arising from multiple side-chain conformations and protonation states that are not well resolved in the electron density. A short MD was run on the solvent only. The entire system was then energy minimized using first a steepest descent minimization to remove conformational stress, followed by a simulated annealing minimization until convergence (<0.01 kcal/mol Å). The MD simulation was then initiated, using the NVT ensemble at 298 K and integration time steps for intramolecular and intermolecular forces every 1.25 fs and 2.5 fs, respectively. Finally, 20 ns MD simulations without any restrictions were conducted, and the conformations of each system were recorded every 200 ps.

### 3.6. In Silico ADMET Studies

In silico molecular studies were conducted with the use of SwissADME [50] and pkCSM [58] web platforms.

## 4. Conclusions

Here, we described the screening of a collection of marine compounds retrieved from the MNP database in search of new potentially active FABP4 inhibitors. The whole dataset was first filtered using a statistical filter, employing 2D and 3D descriptors, and then the 2992 filtered molecules were further evaluated in both a ligand- and a structure-based approach. For the ligand-based evaluation, we used an already successful implemented 3D-QSAR model for FABP4, whereas, for the structure-based evaluation, a docking analysis was performed, and the poses of some selected ligands were validated by MD simulations. The results of both filters were then crossed between them for the 5% more active molecules, highlighting seven compounds possessing a calculated mean activity in the range of 97–331 nM. Interestingly, some of these seven compounds have already been tested for their cytotoxicity, and for anti-inflammatory actions, that could also be due to their activity in FABP4 inhibition.

These seven compounds represent a good starting point for the discovery of novel potent and selective FABP4 inhibitors from natural products; in particular, compounds 5339, 13575, and 1534 possess very good predicted ADMET properties, which make them excellent candidates for becoming new drugs. There is now the need for further in vitro and/or in vivo studies of these marine compounds to experimentally confirm their activity as FABP4 inhibitors. Furthermore, the extension of our research to other compounds (with high-expected activities) in Table S1 would also be a way to go.

## Figures and Tables

**Table 1:** Structure, calculated pIC_50_ and p*K*_i_, and their mean, of the selected marine products.

MNP ID	Structure	pIC_50_ (QSAR)	p*K*_i_ (Docking)	Mean
5339 ^a^	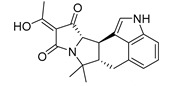	6.30	7.66	6.98
14123 ^b^	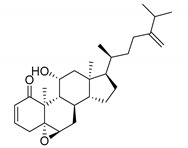	6.30	7.41	6.85
13575 ^b^	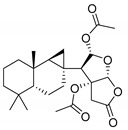	6.10	7.95	7.02
7846 ^b^	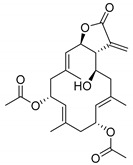	6.40	7.35	6.87
3164 ^b^	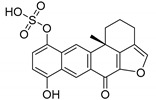	6.30	7.17	6.73
2076 ^b^	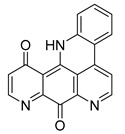	6.10	7.68	6.89
1534 ^b^	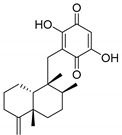	6.10	6.87	6.48

^a^ Present in the first 2% of both ligand- and structure-based filters. ^b^ Present in the first 5% of both ligand- and structure-based filters.

**Table 2:** Drug-likeness, lead-likeness, and PAINS parameters of compounds reported in Table 1 ^a^.

MNP ID		5339	14123	13575	7846	3164	2076	1534
**Drug-likeness**	Lipinski violations	0	1	0	0	0	0	0
	Ghose violations	0	2	0	0	0	0	0
	Veber violations	0	0	0	0	0	0	0
	Egan violations	0	0	0	0	0	0	0
	Muegge violations	0	1	0	0	0	0	0
Lead-likeness violations		0	2	2	1	2	0	1
PAINS alerts		0	0	0	0	0	0	1

^a^ All results were obtained from the SwissADMET web server [50].

**Table 3:** Pharmacokinetic and toxicity evaluated parameters of compounds reported in Table 1 ^a,b^.

MNP ID		5339	14123	13575	7846	3164	2076	1534
**Absorption**	Caco-2 permeability	0.967	1.318	1.700	0.916	−0.363	1.236	0.596
	Human intestinal absorption	92.279	95.061	97.33	88.869	68.223	98.368	91.388
	Skin permeability	−3.198	−2.864	−2.829	−3.486	−2.735	−2.895	−3.482
**Distribution**	VD_ss_ (human)	0.458	−0.177	0.031	−0.276	−1.88	0.145	−0.014
	Fraction unbound (human)	0.157	0.000	0.055	0.338	0.021	0.029	0.067
	BBB permeability	0.536	0.016	−0.707	−0.616	−0.802	0.021	−0.053
	CNS permeability	−2.124	−1.628	−2.183	−2.859	−3.041	−2.176	−1.869
**Excretion**	Total clearance	0.553	0.501	0.228	1.374	0.181	0.444	0.925
	Renal OCT2 substrate ^b^	No	No	Yes	No	No	No	No
**Toxicity**	AMES toxicity	No	No	No	No	No	Yes	No
	Oral rat acute toxicity (LD_50_)	2.564	2.175	2.119	2.823	2.664	2.305	2.341
	Minnow toxicity	−0.338	−0.467	0.169	2.126	−0.189	0.260	0.038

^a^ All results were obtained from the pkCSM web server [58]. ^b^ Semaphore flags: green = good, yellow = tolerable, red = bad. ^b^ Unimportant, because the total clearance is high.

## Supplementary Materials

The following are available online at https://www.mdpi.com/1660-3397/17/11/624/s1, Table S1: Chemical structures of the FABP4 inhibitors used as benchmark for docking methodology validation, including both experimental and calculated p*K*_i_ binding affinities, Figure S1: Linear regression plot of experimental vs. calculated p*K*_i_ values reported in Table S1, Figure S2: Docking binding pose of 5339, Figure S3: Docking binding pose of 14123, Figure S4: Docking binding pose of 13575, Figure S5: Docking binding pose of 7846, Figure S6: Docking binding pose of 3164, Figure S7: Docking binding pose of 2076, Figure S8: Docking binding pose of 1534, Table S2: Chemical structures of the marine dataset that passed the first statistical filter including the ligand- (pIC_50_) and structure-based (p*K*_i_) calculated binding affinity.
