# Supplementary material for: Computational Tools in the Discovery of FABP4 Ligands: A Statistical and Molecular Modeling Approach
Source: Mar Drugs. 2019 Oct 31;17(11):624. doi: 10.3390/md17110624 (PMC6891735; doi:10.3390/md17110624)
Supplement: Supplementary file 1 [file marinedrugs-17-00624-s001.pdf]

marine drugs

MDPI

Article

Computational tools in the discovery of FABP4 ligands: a statistical and molecular modeling approach

Giuseppe Floresta [1,*], Davide Gentile [1], Giancarlo Perrini [2], Vincenzo Patamia [1] and Antonio Rescifina [1,3,*]

[1] Department of Drug Sciences, University of Catania, V.le A. Doria, 95125 Catania, Italy; davide.gentile@unict.it (D.G.), vincenzo.patamia@unict.it (V.P.)

[2] Department of Chemical Sciences, University of Catania, V.le A. Doria, 95125 Catania, Italy; gperrini@unict.it

[3] Consorzio Interuniversitario Nazionale di ricerca in Metodologie e Processi Innovativi di Sintesi (C.I.N.M.P.S.), Via E. Orabona, 4, 70125 Bari, Italy

* Correspondence: giuseppe.floresta@unict.it (G.F.); arescifina@unict.it (A.R.)

Table of content

Table S1. Chemical structures of the FABP4 inhibitors used as benchmark for docking mythology validation, including both experimental and calculated pK_i binding affinities. S2

Figure S1. Linear regression plot of experimental *vs*. calculated pK_i values reported in Table S1 S3

Figure S2. Docking binding pose of 5339 S3

Figure S3. Docking binding pose of 14123 S4

Figure S4. Docking binding pose of 13575 S4

Figure S5. Docking binding pose of 7846 S4

Figure S6. Docking binding pose of 3164 S5

Figure S7. Docking binding pose of 2076 S5

Figure S8. Docking binding pose of 1534 S5

Table S2. Chemical structures of the marine dataset that passed the first statistical filter including the ligand- (pIC$_{50}$) and structure-based (pK_i) calculated binding affinities S5

Table S1. Chemical structures of the FABP4 inhibitors used as benchmark for docking methodology validation, including both experimental and calculated pK_i binding affinities.

Compound[a]	SMILES	Exp. pK_i	Calcd. pK_i
BMS309403	CCC1=C(C2=CC=CC=C2)C(C3=CC=CC=C3)=NN1C4=CC=CC=C4C5=CC=CC(OCC(O)=O)=C5	7.82	7.79
**1a**	[H]C(C(O)=O)(OC1=CC(C2=CC=CC=C2C3=NN(C(C4=CC=CC=C4)=C3)C5=CC=C(C=C5)[H])=CC=C1)[H]	7.49	7.20
**1b**	[H]C(C([O-])=O)OC1=CC(C2=CC=CC=C2C3=NN(C4=CC=C(Cl)C=C4)C(C5=CC=CC=C5)=C3)=CC=C1	7.89	7.82
**1c**	[H]C(C(O)=O)(OC1=CC(C2=CC=CC=C2C3=NN(C(C4=CC=CO4)=C3)C5=CC=C(C=C5)Cl)=CC=C1)[H]	8.15	8.24
**1d**	[H]C(C(O)=O)(OC1=CC(C2=CC=CC=C2C3=NN(C(C4=CC=CS4)=C3)C5=CC=C(C=C5)Cl)=CC=C1)[H]	8.30	7.35
**1f**	[H]C(CC)(C([O])=O)OC1=CC(C2=CC=CC=C2C3=NN(C4=CC=C(Cl)C=C4)C(C5=CC=CS5)=C3)=CC=C1	8.22	7.33
**2a**	O=C([O-])CCCCOC1=C(C2=NC(C3=CC=CC=C3)=C(C4=CC=CC=C4)O2)C=CC=C1	7.24	7.28
**2b**	O=C(COC1=CC=CC(C2=C(C=CC=C2)C3=NC(C4=CC=CC=C4)=C(O3)C5=CC=CC=C5)=C1)[O-]	8.22	8.19
**3a**	O=C(COC1=CC(C2=CC=CC=C2C3=NC(C4=CC=CC=C4)=C(C5=CC=CC=C5)N3)=CC=C1)[O-]	8.03	7.65
**3b**	O=C(CNC1=CC(C2=CC=CC=C2C3=NC(C4=CC=CC=C4)=C(C5=CC=CC=C5)N3)=CC=C1)[O-]	7.74	8.50
**3d**	CN1C(C2=CC=CC=C2C3=CC=CC(NCC([O-])=O)=C3)=NC(C4=CC=CC=C4)=C1C5=CC=CC=C5	8.57	8.30
**3g**	[O-]C(CNC1=CC(C2=CC=CC=C2C3=NC(C4=CC=CC=C4)=C(C5=CC=CC=C5)N3CC(C)C)=CC=C1)=O	6.89	6.81
**3f**	[O-]C(CNC1=CC(C2=CC=CC=C2C3=NC(C4=CC=CC=C4)=C(C5=CC=CC=C5)N3CC)=CC=C1)=O	8.47	8.33
**18**	O=C([O-])CCN1C(C2=CC=CC(OC(C)C)=C2)=C(C3=C(C)NN=C3C)C4=C1C=CC(C5CC5)=C4	7.52	7.52
**19**	O=C([O-])CCN1C(C2=CC=CC=C2)=C(C3=C(OC)C=NC=C3)C4=C1C=CC(C5CC5)=C4	7.00	6.89
**20**	O=C([O-])CCN1C(C2=CC=CC=C2)=C(C3=CC=CC=C3)C4=C1C=CC(C5CC5)=C4	6.92	7.11
**33**	ClC(C=C1)=CC2=C1N=C(C)C(C(O)=O)=C2C3=CC=CC=C3	6.98	6.52
**34a**	ClC1=CC2=C(N=C(C([O-])=O)C(C(C)C)=C2C3=CC=CC([H])=C3)C([H])=C1	7.92	7.99
**34b**	ClC1=CC2=C(N=C(C([O-])=O)C(N3CCCCC3)=C2C4=CC=CC([H])=C4)C([H])=C1	7.66	7.68
**34c**	ClC1=CC2=C(N=C(C([O-])=O)C(N3CCCCC3)=C2C4=CC=CC([H])=C4)C(Cl)=C1	7.80	7.57
**34d**	ClC1=CC2=C(N=C(C([O-])=O)C(N3CCCCC3)=C2C4=CC=CC([H])=C4)C(C)=C1	7.89	7.77
**34e**	ClC1=CC2=C(C=C1)N=C(C(C(C)C)=C2C3=CC=CC(C(C)C)=C3)C([O-])=O	7.80	7.99
**34f**	ClC1=CC2=C(N=C(C3=NN=NN3)C(N4CCCCC4)=C2C5=CC=CC([H])=C5)C=C1	7.96	7.25
**34g**	ClC1=CC2=C(N=C(C3=NNC(O3)=O)C(N4CCCCC4)=C2C5=CC=CC([H])=C5)C=C1	7.70	7.65
**34h**	ClC1=CC2=C(N=C(C3=NNC(O3)=S)C(N4CCCCC4)=C2C5=CC=CC([H])=C5)C=C1	8.15	8.22
**39**	O=C(/C(S1)=C\C2=CC(OCC)=C(O)C=C2)N(CCC([O-])=O)C1=S	5.60	5.83
**41**	O=S(NC1=CC(SCC([O-])=O)=C(O)C2=CC=CC=C21)(C3=CC=C(Cl)C=C3)=O	5.64	5.71

**42**	O=C([O-])C(C1C2C(Cl)C(SC3=CC=CC=C3[N+]([O-])=O)C1)[NH+]4CC5C(CCC5)C6=CC=CC2=C64	5.50	5.25
**42a**	[O-]C(C1C2C(C=CC2)C3=C(C)C=C(C)C4=C3[NH+]1CC5C4C=CC5)=O	6.21	7.01
**42b**	[O-]C(C1C2C(C=CC2)C3=CC(Br)=CC4=C3[NH+]1CC5C4C=CC5)=O	6.54	6.88
**42c**	[O-]C(C1C2C(C=CC2)C3=CC(C)=CC4=C3[NH+]1CC5C4C=CC5)=O	6.42	6.57
**42d**	[O-]C(C1C2C(C=CC2)C3=CC(Cl)=CC4=C3[NH+]1CC5C4C=CC5)=O	6.16	6.22
**44**	CC1=C(C)C=CC(/C=C2NC(/C(S/2)=C/C3=CC=CN3C4=CC=CC(C([O-])=O)=C4)=O)=C1	5.44	5.12
**45**	O=S(C1=CC(OCCC)=C(Cl)C=C1)(NC2=CC=C(C([O-])=O)C=C2)=O	5.37	5.44

[a] The numbers correspond to that reported in the reference [1].

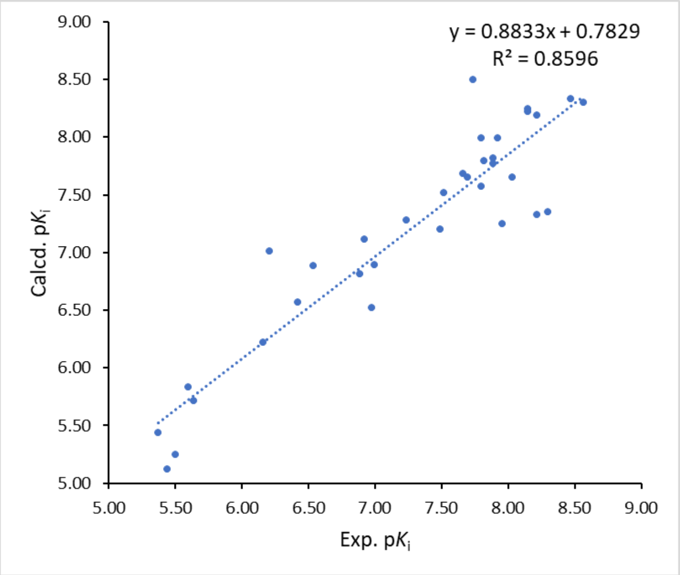

Figure S1. Linear regression plot of experimental *vs*. calculated pK_i values reported in Table S1.

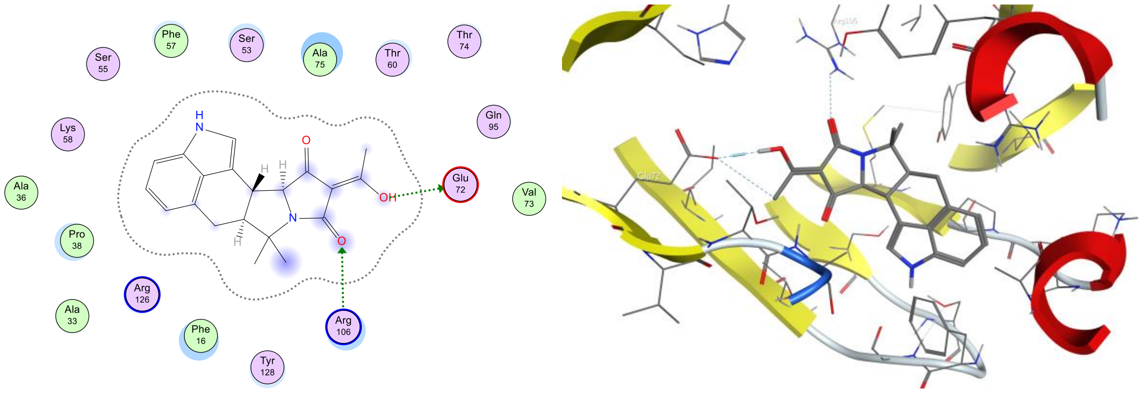

Figure S2. Docking binding pose of 5339.

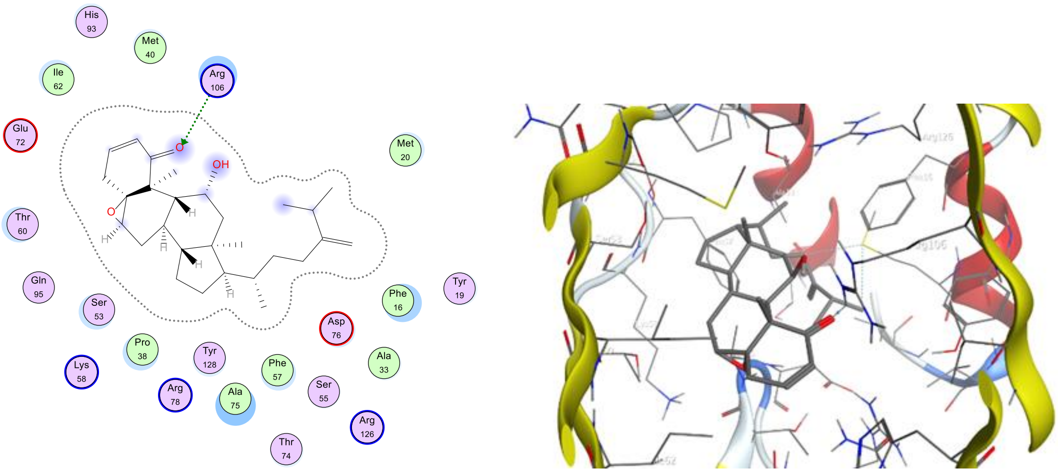

Figure S3. Docking binding pose of 14123.

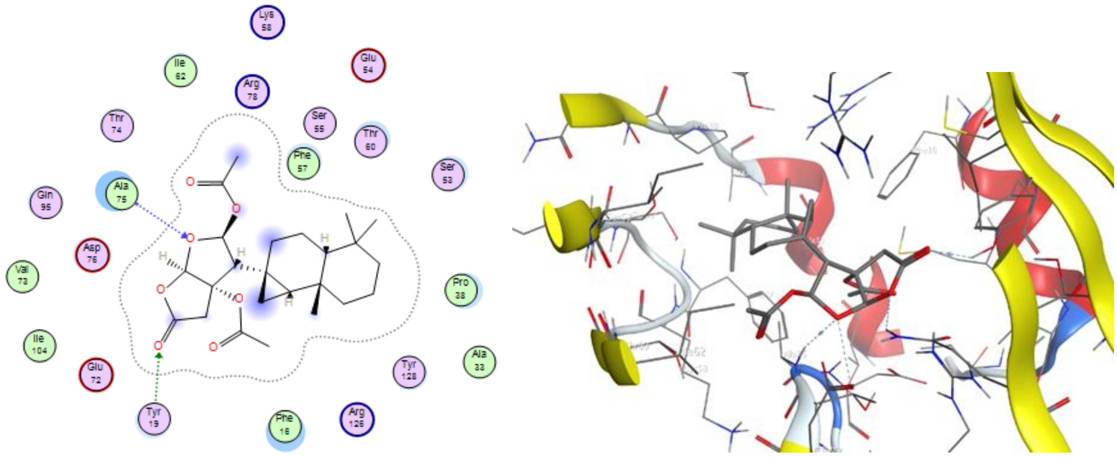

Figure S4. Docking binding pose of 13575.

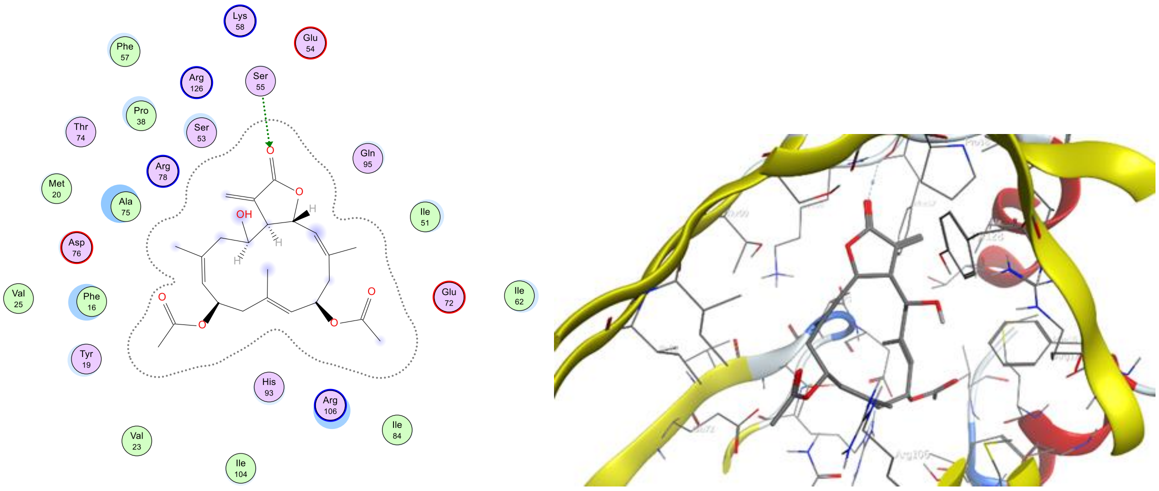

Figure S5. Docking binding pose of 7846.

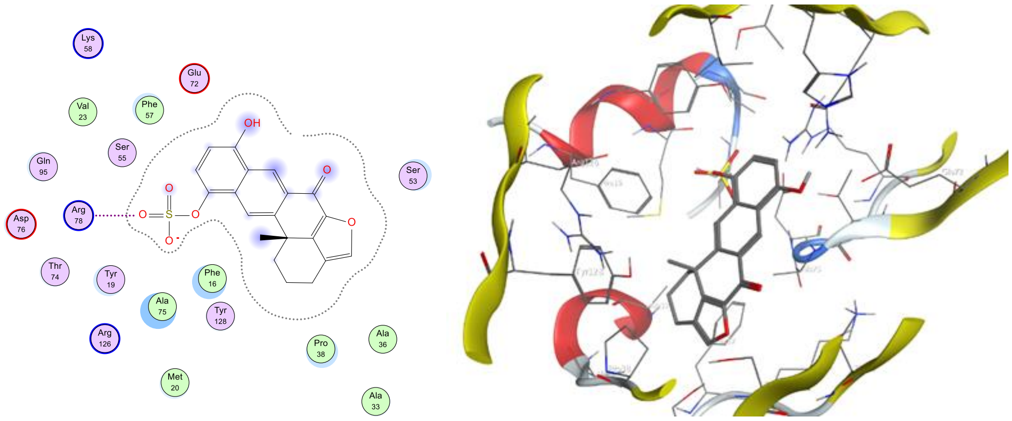

Figure S6. Docking binding pose of 3164.

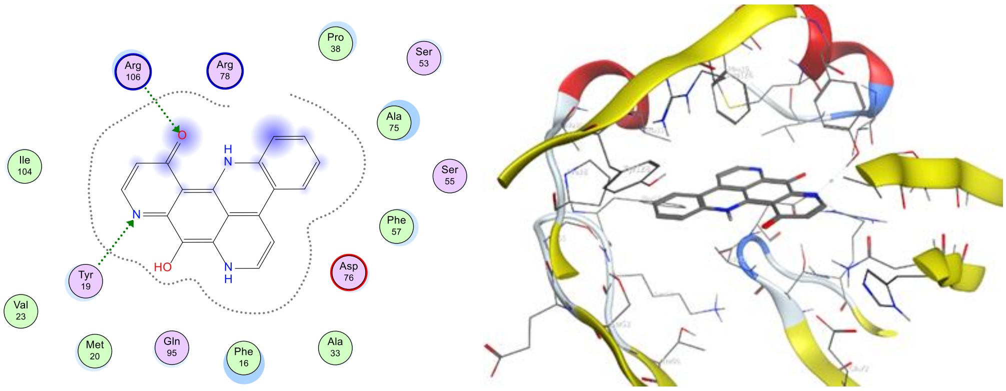

Figure S7. Docking binding pose of 2076.

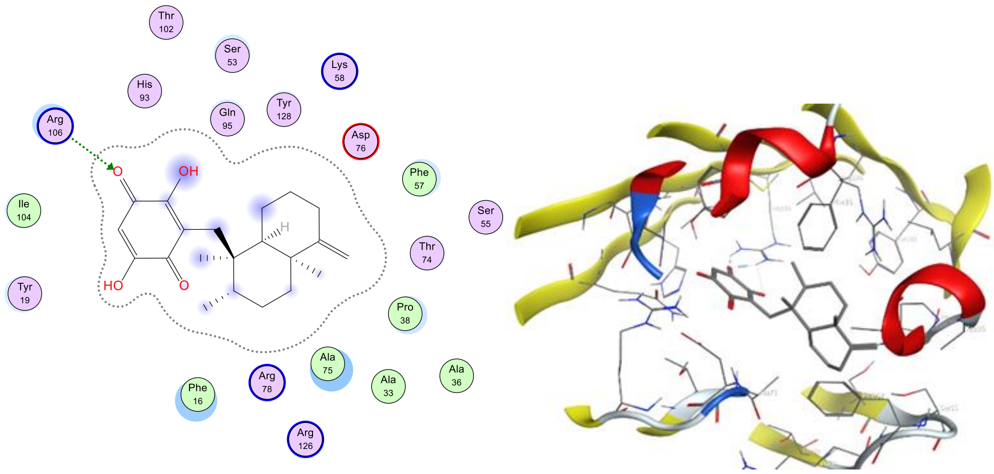

Figure S8. Docking binding pose of 1534.

Table S2. Chemical structures of the marine dataset that passed the first statistical filter including the ligand- (pIC_{50}) and structure-based (pK_i) calculated binding affinities.

MNP ID[a]	SMILES	pIC_{50}[b]	3D-QSAR description	pK_i[c]
13469	O1c2c(CC13C1(C(C45C(CC1)C(CCC4)(C)C(OC5)=O)CCC3(O)C)C)cc(O)cc2	5.4	Excellent	9.3
1404	n1c2c3c(c4c1cccc4)ccnc3c1nccc3c1c2nc1c3cccc1	5.3	Bad	8.9
3305	O1C2n3c4c(c5c(c6c7c(n(c46)C1(C)C(OC)C(O)C2)cccc7)C(=O)NC5)c1c3cccc1	5.5	Good	8.7
1256	O(C)c1c2nccc3-c4c(N5c(c(C6C(C)C5(C)C(=O)NC6=O)c1)c23)cccc4			8.7
2549	O=C1C2C3(Nc4c1cccc4)C=1C(=NC2)CCNC=1c1nc2c(c4c1c3ncc4)cccc2	6.6	Good	8.6

3556	O1C(C1(C)C)C1N2C3N(CCC34C3(CC2)c2c1cccc2N(C3Nc1c4cccc1)C)C(=O)C			8.6
1589	O(C)c1c2nccc3-c4c(N5c(c(C6C(C)C5(C)C(=O)NC6=O)c1)c23)cccc4			8.4
3085	O1C(COC(c2ccccc2)(c2ccccc2)c2ccccc2)C(O)CC1N1C=CC(=O)NC1=O	4.5	Bad	8.4
2550	O=C1C2C3(Nc4c1cccc4)c1c(nccc1NC2)-c1nc2c(c4c1c3ncc4)cccc2	5.2	Excellent	8.3
711	O1C2CC(OC(=O)C)C3(C(Cc4c(occ4C)C=C(C=CC3)C)C12C)C	4.2	Poor	8.3
8107	O(C)c1c2nccc3-c4c(N5c(c(C6C(C)C5(C)C(=O)NC6=O)c1)c23)cccc4			8.2
3983	O1C2CC(CC=C3CC(OC3=O)CC(C(OC)=O)C(=O)CC(=O)C12C)C(C)=C	5.1	Poor	8.1
13477	o1c2c3c(c1)C(=O)CCC3(c1c(cc3c(c1)C(=O)C=CC3=O)C2=O)C	4.9	Excellent	8.1
8421	O1C2CCC1(C)C(OC(=O)C)C(OC(=O)C)C=1C(OC(=O)C=1C)CC(=CCCC2C)C	4.8	Poor	8.0
4152	OC12C=C(C3C4(C1N1C(C4)C=CCCCC1)CN(CC3)CCCCC=CCC2)CO	4.2	Good	8.0
13575	O1C2OC(=O)CC2(OC(=O)C)C(C23C(C2)C2(C(CC3)C(CCC2)(C)C)C)C1OC(=O)C	6.1	Bad	7.9
1257	O(C)c1c2nccc3-c4c(N5c(c(C6C(C)(C5C)C(=O)NC6=O)c1)c23)cccc4	5.0	Excellent	7.9
12059	O=C1N(C)C(=O)C=2N(C3Nc4c(cccc4)C3(C=2)c2c3c([nH]c2)cccc3)C1=O	5.0	Poor	7.9
8422	O1C2CCC1(C)C(OC(=O)C)C(OC(=O)C)C=1C(OC(=O)C=1C)CC(=CCCC2C)C	5.8	Excellent	7.9
5744	O1C(C2(C(CC1=O)=C1C(CC2)C2(C)C(CC(OC)=O)C(C)(C)C(=O)C(C1)C2=O)C)c1ccoc1			7.9
488	ClC1CC2C3(SC(=O)NC3C1(C=C)C)c1c3c(cccc3[nH]c1)C2(C)C	5.9	OK	7.9
7410	S1CC(=O)Nc2c3nccc4c3c3n(c5c4cccc5)c(N(C)C)cc3c12	4.9	Bad	7.9
4852	O=C1NC(C=2N(C1Cc1c3c([nH]c1)cccc3)C(=O)c1c(N=2)cccc1)C	5.0	OK	7.9
2216	OC1C2C(C3CCC(C(O)(C=C(CC(C)C)C)C)C3(C1)C)C(=O)CC1CCC=CC12C	5.9	Poor	7.9
160	O1C2C(CCCC(=O)C(CC(O)C1(C)C)=C2CC1(Oc2c(cc(O)cc2C)CC1)C)(C)C	4.8	Poor	7.9
7313	O1C2C(CC3C(C)(C(OC(=O)C)C(=O)C=C3C)C(OC(=O)C(=CC)C)CCC(=C2)C)=C(C)C1=O	5.6	Bad	7.8
6794	O1C2CCC(C)C(CCC3=C(C)C(=O)CC4C(C)(C)C(=O)CCC34C)C2(CCC(=O)C1(C)C)C	5.2	Poor	7.8
7354	O(C(=O)C=1c2[nH]c3c(c2C=C(c2[nH]c4c(c2C=1)cccc4)C(OC)=O)cccc3)C	5.1	Good	7.8
2912	OC12C=C(C3C4(C1N1C(C4)C=CCCCC1)CN(CC3)CCCCC=CCC2)C=O	3.1	OK	7.8
1280	O1C2CC(O)(CC(=O)C=C(C)C(=O)CC(CCC(=C2)C1=O)C(C)=C)C	5.7	OK	7.8
8139	ClC=1CCC2OC(C(OC(=O)CC(OC(=O)C)C(=CCC=1)C)C2)C1OC(=O)CC(=C1)C	5.4	Poor	7.8
6935	O1CC(=C(c2c3c([nH]c2)cccc3)C1=O)c1c2c([nH]c1)cccc2	5.3	Excellent	7.8
5891	O=C1C2=CC(=O)CCC2(C2C(C3CCC(C(CC=CC(OO)(C)C)C)C3(CC2)C)C1)C	4.7	Bad	7.8
7850	O1C2C(CCC(=CC(OC(=O)C)CC(=CC(O)CC(=C2)C)C)C)C(=C)C1=O	5.6	Poor	7.7
6663	BrC1=CC23C4=C(NC(SC2=CC1=O)C3)C(=O)c1[nH]cc2c1c4ncc2	4.9	Excellent	7.7
169	O1C2C(CCCC(=O)C(CC(O)C1(C)C)=C2CC1(Oc2c(cc(O)cc2C)CC1)C)(C)C	5.1	Poor	7.7
10265	O1C23C(OC(=O)C12C)C=C(CCC(OC(=O)C)C1(C(C(C)C(=O)C=C1)C3O)C)C	5.4	Excellent	7.7
9401	Oc1ccc(cc1)C=1NC(=C2N(C=1)C(=O)C(=N2)Cc1ccccc1)Cc1ccccc1	5.6	Bad	7.7
348	O1C2C(CCC(=CCCC(=CC(OC(=O)C)CC(=C2)C)C)C)C(=C)C1=O	4.1	Poor	7.7
4301	O1C2=C3C(C1)C(O)CCC3(c1c(cc3c(c1)C(=O)C=CC3=O)C2=O)C	5.6	Poor	7.7
2076	O=C1C=2C(=NC=C1)C(=O)c1nccc-3c1C=2Nc1c-3cccc1	6.1	Good	7.7
7770	O(C(=O)CC(O)C)C1CC2C(C3CC(=O)C(=CC13C)C(=O)C)(CCC1C3(CC3C)CCCC12C)C	5.4	Good	7.6
5339	OC=1C2N(C(=O)C=1C(=O)C)C(C1C2c2c3c(C1)cccc3[nH]c2)(C)C	6.3	OK	7.6
1065	BrC1=CC23C4=C(NC(SC2=CC1=O)C3)C(=O)c1[nH]cc2CCN=C4c12	5.5	Poor	7.6
11051	O1CC23C4CCC(O)(C)C(CC5=CC(=O)C=C(OC)C5=O)C4(CCC2C(CCC3)(C)C1=O)C	5.0	Excellent	7.6
1869	O1C2n3c4c(c5c(CNC5=O)c5c6c(n(c45)C1(C)C(OC)C(O)C2)cccc6)c1c3cccc1	5.0	Good	7.6
13431	O1C2CCC(O)(C)C(CCC3(C=4C(OC(C)(C)C(O)CC=4)CCC3C)C)C2(CCC(=O)C1(C)C)C	4.4	Bad	7.6
6066	S(OC=C1CCC2C(CCC3C(CCCC23C)(C)C)(C)C1CCc1ccoc1)(O)(=O)=O	5.2	Good	7.6
3750	O1C=C2C(CCC=C2C)(c2c(cc3c(c2)C(=O)C=CC3=O)C1=O)C	4.9	Good	7.6

2999	O=C1C=C(c2nc3c(c4c2c1ncc4)cccc3)C(O)CNC(=O)C(=CC)C	4.0	Good	7.6
346	O1C2C(C(=C)C1=O)C(OC(=O)C)CC(=CCCC(=CC(OC(=O)C)CC(=C2)C)C)C	5.3	Bad	7.6
13678	O1C(=O)C(C2CC3OC3(CCC(OC(=O)C)C3(OC(CC3)C1(CC2)C)C)C)=C	6.0	Excellent	7.6
7472	O1C2CC(C(=C)C1=O)C(O)CC(=CCCC(=CCCC2(O)C)C)C	4.9	Good	7.6
5275	O1C23CC(O)CC(=O)C2(C2C(C4CCC(C(CCC(C(C)C)C)C)C4(CC2OC(=O)C)C)CC13)C	4.0	OK	7.6
14256	O1C2OC(=O)C3C2C2(C(CC3)C3(C(CC2OC(=O)C)C(CCC3)(C)C)C)C1O	4.7	Poor	7.6
14229	o1c2c3c(c1)C(=O)CCC3(c1c(cc3c(c1)c(O)ccc3O)C2=O)C	4.8	Good	7.6
12164	O(C(=O)C)C1CC2(C(=C1C(C)C)C(OC(=O)C)CC1(C)C(O)(C2)C(CCC1O)=C)C	6.0	Good	7.6
7757	O1C(C2=C(C3(C(CC2)C2(C(CC3OC(=O)CC(O)C)C3(C(CC2)C2(CC2C)CCC3) C)C)C)C1=O)C	5.2	Good	7.6
2478	O1C2c3oc(CC(CC(OC(=O)C)C4=CC(OC4=O)CC12C)C(C)=C)c(c3)C=O	5.7	Poor	7.6
1009	O1C(=O)C(CCC1C(C)C1CCC2C3C(CCC12COC(=O)C)C1(C(=CC(=O)C=C1)C C3)C)C	4.6	Bad	7.6
13810	O=C1CC2C(CCC3C(C)(C)C(=O)CCC23C)(C)C1=C(C=O)C	5.2	Good	7.6
11480	O1C2C(CCC(O)(C)C(O)CCC(=CCCC(=C2)C)C)=C(C)C1=O	5.1	Excellent	7.6
9400	Oc1ccc(cc1)-c1nc(Cc2ccccc2)c(nc1)NC(=O)Cc1ccccc1	5.1	Bad	7.6
14086	S(OC=C1CCC2C(CCC3C(CCCC23C)(C)C)(C)C1CCc1ccoc1)(O)(=O)=O	4.7	Poor	7.5
8920	O1C(C(C)C)C(=O)N2C3=C(CC2)c2nc4c(c5c2c(ncc5)C13O)cccc4	4.4	Excellent	7.5
1950	O1C(OC(=O)C)C2C(C(O)CC3C4(C(CCC23C)C(CCC4)(C)C)C)C1OC(=O)C	4.2	OK	7.5
9043	O1C2C(C(=C)C1=O)C(O)CC(=C)C(OC(=O)C)CCC(=CC(OC(=O)C)CC(=C2)C) C	5.3	OK	7.5
14443	O1C2C(CC=C(C3OOC(CC3)(C)C(OC(=O)C)CCC(=C2)C)C)C(=C)C1=O	5.4	OK	7.5
14126	O1C23C1CC1C(C2(C)C(=O)C=CC3)C(O)CC2(C1CCC2C(CCC(C(C)C)C)C)C	5.4	Poor	7.5
9396	O1C=C(C2C(CN3C(C2)C2(CC3)c3c(NC2=O)cccc3)C1C)C(OC)=O	3.9	Excellent	7.5
9214	O(C)C1c2c(C(=O)N(C)C1OC)c(O)c1nccc3-c4c(Nc2c13)cccc4	5.5	Excellent	7.5
7182	Brc1c(cc2C3C(CCC3C(C)C)(C)C(O)(C(OC)=O)C(=O)c2c1O)C	4.7	Excellent	7.5
8909	s1c2c(nc1)c1nccc3-c4c(Nc(c13)c2CCNC(=O)C(CC)C)cccc4	4.0	Excellent	7.5
4298	S(C)C=1C(=O)c2nccc3c2c(nc2c3cccc2)C=1CCNC(=O)C(=CC)C	4.4	Good	7.5
1667	ClC1CC2C(O)(C([NH+]=[CH-])C1(C=C)C)C(=O)c1c(cccc1NC=O)C2(C)C	4.7	Excellent	7.5
13410	S=C=NC(CC1C2C3C(CCC(C)C3([NH+]=[CH-])CCC2=C)C(C1)C)(C)C	3.8	Good	7.5
12388	O1C2C(CC3OC3(CCC=C(CCC=C(C)C2O)C)COC(=O)C)C(=C)C1=O	4.5	Good	7.5
11973	O1CC(C2CC(C)C(OC(=O)CCC)C3C2C2OC3CC(=CCC(OC(=O)C)C12C)C)C	5.5	OK	7.5
10174	O1C2c3oc(CC(CCC4=CC(OC4=O)CC12C)C(C)=C)c(c3)C(OC)=O	5.7	Poor	7.5
9349	O1C2=CC(=O)C3(O)CC(OC3C2=CC2C3(C(CCC12C)C(CCC3)(C)C)C)OC	6.2	Poor	7.5
8530	O1CC2=C(CCC3C2(CCC2C(C)=C(O)C(=O)CC23C)CO)C1=O	5.2	Good	7.5
7386	O1C23C(C4CCC(C(CCCC(C)C)C)C4(CC12)C)=CC(OC(=O)C)C1(O)CC(O)CC C13C	6.2	OK	7.5
6936	O=C(c1c2c([nH]c1)cccc2)c1nccc2c1[nH]c1c2cccc1	6.0	Excellent	7.5
6889	O1C23C1(C1CCC(C(C=CC(C(C)C)C)C)C1(CC2)C)C(O)C1OC12CC(O)CCC23 C	5.4	Poor	7.5
6829	Oc1c2c3C(=C(C)C(=O)C(=O)c3c(c1)C)C(CC2C)C=C(C)C	5.7	Excellent	7.5
5477	OC1(CC(O)C=C(CC(=O)C(CCC(C=C1)C(C)C)(COC(=O)C)C)C)C	6.1	OK	7.5
5274	O1C23CC(O)CC(=O)C2(C2C(C4CCC(C(C=CC(C(C)C)C)C)C4(CC2OC(=O)C) C)CC13)C	5.6	Poor	7.5
3281	Oc1ccc(cc1)-c1nc(Cc2ccccc2)c(nc1)NC(=O)Cc1ccccc1	5.2	Poor	7.5
1643	OC1CC2=CCC3C4CCC(=NO)C4(CCC3C2(CC1)C)C	4.8	Poor	7.5
938	o1c2c3c(CCCC3(c3c(cc4c(c3)C(O)CCC4O)C2=O)C)c1	5.3	Poor	7.5
162	O1C2C(CCCC(=O)C(C=CC1(C)C)=C2CC1(Oc2c(cc(O)cc2C)CC1)C)(C)C	5.2	Excellent	7.5
5269	O=C1N2C(CCC2)C(=O)NC1Cc1c2c([nH]c1CC=C(C)C)cccc2	5.5	Excellent	7.5
447	O1CC2C(CCC3C2(COC(=O)C)C(OC(=O)C)CC2C(CCCC23C)(C)C)C1=O	6.2	OK	7.5
9329	O1C23C(CCC(C)C2(CC2=C1C(=O)C=C(N)C2=O)C)C(CCC3)(C)C	5.1	OK	7.4

3742	O1C2=CC(=O)C(O)=CC2=C(C2C3(C(CCC12C)C(CCC3)(C)C)C)C#N	4.4	OK	7.4
11107	O1C2CCC(=CCCC(=CC3OC(=O)C(C3CC(=O)C12C)=C)C)C	5.4	Excellent	7.4
6778	O=C1C(=CC(=O)C=C1C)CC1(C2CCC(=C(C)C)C(CCC(O)=O)C2(CCC1C)C)C	5.6	Poor	7.4
3000	O=C1C=C(c2nc3c(c4c2c1ncc4)cccc3)C(OC)CNC(=O)C=C(C)C	4.7	Good	7.4
4292	s1c2c(nc1)c1nccc3-c4c(Nc(c13)c2CCNC(=O)C=C(C)C)cccc4	5.1	Poor	7.4
916	O=C1C=C(c2nc3c(c4c2c1ncc4)cccc3)CCNC(=O)C=C(C)C	5.7	Good	7.4
14123	O1C23C1CC1C(C2(C)C(=O)C=CC3)C(O)CC2(C1CCC2C(CCC(C(C)C)=C)C)C	6.3	OK	7.4
11162	O1CC2C(CC3C(OC(=O)C=C3C)C2=C)C(=CC=CC(O)(C)C)C1=O	5.1	Poor	7.4
9456	O1C=C(C2C(CN3C(C2)C2(CC3)c3c(NC2=O)cccc3)C1C)C(OC)=O	4.4	Good	7.4
7851	O1C2C(CCC(=CCCC(=CC(OC(=O)C)CC(=C2)C)C)C)C(=C)C1=O	3.5	Bad	7.4
5731	O1C2CCC(C)C(O)(CCC3(C4C5(OC(C(O5)CC4)(C)C)CCC3C)C)C2(CCC(=O) C1(C)C)C	5.0	Bad	7.4
4837	ClC1=C(O)C(=O)c2c(cc3c(c2)C(=O)c2occ4c2C3(CCC4)C)C1=O	5.4	OK	7.4
822	O=C1N2C(Cc3c([nH]c4c3cccc4)C2C=C(C)C)C(=O)N2C1CCC2	5.7	Poor	7.4
13896	OC12C(CCCC1(C)C)C(CCC(=CCn1c3c(nc1)N(C)C(=NC3=O)C)(C)C(CC2)C	6.0	Good	7.4
12188	O1CC(C2CC(C)C(OC(=O)CCC)C3C2C2OC3CC3(OC3CC(OC(=O)C)C12C)C) C	4.7	Poor	7.4
4822	O1C2CCC1(C)C(O)CC1C(OC(=O)C1=C)C(O)C(CCCC2=C)C	4.8	Excellent	7.4
2708	O1CC(C2CC(C)C(OC(=O)C)C3C2C2OC3CC(=CCCC12C)C)C	5.1	Good	7.4
13809	O=C1CC2C(CCC3C(C)(C)C(=O)CCC23C)(C)C1=C(C=O)C	3.7	Excellent	7.4
9211	O(C)c1c2c3c(nc1)C(=O)C1=C(C=CN(C)C1=O)c3nc1c2cccc1	5.8	Excellent	7.4
8369	OC1C2CC(O)CCC2(C=2C(C3CCC(C(CC(=O)C=C(C)C)C)C3(CC=2)C)C1)C	4.6	Poor	7.4
7848	O1C2C(C(=C)C1=O)C(OC(=O)C)CC(=CCCC(=CC(O)CC(=C2)C)C)C	4.7	Poor	7.4
3403	O1C2=C(C3C(CCC(=C3)C)C1(CCC=C(C)C)C)C(=O)c1c(C2=O)c(O)cc(O)c1	4.1	Poor	7.4
3287	O1C2C(C(=C)C1=O)C(OC(=O)C)CC(=CC(OC(=O)C)CC(=CCCC1(OC12)C)C) C	5.6	Excellent	7.4
2075	Oc1c-2c(ncc1)C(=O)c1nccc3c1c-2nc1c3cccc1	5.2	Good	7.4
495	ClC1CC2C(c3c4c(cccc4[nH]c3)C2(C)C)C([NH+]=[CH-])C1(C)C1OC1	5.3	Excellent	7.4
460	Oc1cc2[nH]c3c(CC4N(C3C=C(C)C)C(=O)C3N(CCC3)C4=O)c2cc1	5.9	Poor	7.4
12603	O(C(=O)C)C1CC=2C(CC3(O)C1(C)C(OC(=O)C)CCC3=C)(CCC=2C(C)C)C	4.6	OK	7.4
9106	OC1C2C(C3CCC(C(CCC(C(C)C)C)C)C3(C1)C)CC(O)C1=CC=CC(=O)C12C	5.4	Bad	7.4
7935	OC=1C(=O)c2c3c(C=1C)c(cc(c3c(O)cc2C)C)C=C(C)C	4.8	Excellent	7.4
5539	Brc1cc2[nH]cc(c2cc1)C1=C(c2n(cnc2)C)C(=O)NC1=O	5.1	Excellent	7.4
3692	O1C2CCC(C)C(CCC3=C(C)C(=O)C(O)=C4C(C)(C)C(=O)CCC34C)C2(CCC(=O)C1(C)C)C	5.4	Poor	7.4
2707	O1CC(C2CC(C)C(OC(=O)CCC)C3C2C2OC3CC(=CCCC12C)C)C	4.5	OK	7.4
2417	O1C23C1C(O)C=1C4CCC(C(C=CCC(C)C)C)C4(CCC=1C2(CCC(O)C3)C)C	4.8	Poor	7.4
2015	O1C2OC(OC(=O)C)C(C(OC(=O)C)C1=O)C2C(=C)C1CCC2C1(CCCC2(C)C)C			7.4
6996	O1C(O)C2=C(C3(C(CC2)C2(C(CC3)C(CCC2)(C(O)=O)C)C)C)C1=O	5.2	Excellent	7.4
4859	O=C1NC(C=2N(C1Cc1c3c([nH]c1)cccc3)C(=O)c1c(N=2)cccc1)C	5.0	Excellent	7.3
4667	O1C(=O)C(C2C3C(C4OC3C1(CCC(O)C(C4)=C)C)C(OC(=O)CCC)(CC2)C)C	3.6	OK	7.3
2998	O=C1C=C(c2nc3c(c4c2c1ncc4)cccc3)C(O)CNC(=O)C=C(C)C	4.6	Poor	7.3
2571	O1C2N3CC1(CC3C1C23C(CCC1C(OC)=O)C1(C(CC3OC(=O)C)C(CCC1)(C)C)C)C			7.3
5273	O1C23CC(O)CC(=O)C2(C2C(C4CCC(C(CCC(C(C)C)=C)C)C4(CC2OC(=O)C) C)CC13)C	3.7	Poor	7.3
395	BrC1=CC2(C=C(Br)C1=O)C1=C(NCC2)C(=O)c2[nH]cc3CCN=C1c23	5.4	Good	7.3
13420	O=C1CC2C3(CC(C2CCC3C)(C)C)C1CN1C=2N=CNC(=O)C=2NC1=O			7.3
11161	O1CC2C(CC3C(OC(=O)C=C3C)C2=C)C(=CC=CC(O)(C)C)C1=O	5.2	Bad	7.3
10708	O1C2n3c4c(c5c(CNC5=O)c5c6c(n(c45)C1(C)C(OC)C(NC)C2)cccc6)c1c3cccc1	4.9	Poor	7.3
5215	ClC1=CC(O)C=CC(C2C3(N4C(CCC3)CC=C(C4)C(OCC1)=O)CCC2)C	4.1	Good	7.3
11044	S1C2=CC(=O)C=CC23C2=C(NC1C3)C(=O)c1[nH]cc3CCN=C2c13	4.9	Excellent	7.3

7846	O1C2C(C(=C)C1=O)C(O)CC(=CC(OC(=O)C)CC(=CC(OC(=O)C)CC(=C2)C)C)C	6.4	Poor	7.3
7758	O1C(C2=C(C3(C(CC2)C2(C(CC3OC(=O)CC(O)C)C3(C(CC2)C2(CC2C)CCC3)C)C)C)C1=O)C	4.9	Poor	7.3
6857	O1C(C2C3(CC=C4C(=CC(C5CC(O)CCC45C)(C)C)C3(CC2)C)C1=O)(CCCC(O)(C)C)C	4.8	Poor	7.3
5818	o1c2c(cc1)C(=O)CC1C3(C(CCC12C)C(CCC3)(C)C)COC(=O)C	5.4	Good	7.3
5123	O1C2(OC(=CC3(CCCC23C)C)CC(=CCC2=CC(=O)C=C(C)C2=O)C)C=CC1(C)C	4.3	Bad	7.3
4306	O1C2C3C(C1CC(O)(C)C(OC(=O)CC(C)C)CCC2(OC(=O)C)C)C(CCC3C(C)C)=C	4.4	OK	7.3
3691	O1C2CCC(O)(C)C(CCC3=C(C)C(=O)C(O)=C4C(C)(C)C(=O)CCC34C)C2(CCC(=O)C1(C)C)C	4.4	Bad	7.3
2570	O1C2N3CC1(CC3C1C23C(CCC1C(OC)=O)C1(C(CC3OC(=O)CCC)C(CCC1)(C)C)C)C			7.3
2458	O1C2C(CC(O)C(CCC(=CCCC(=C2)C)C)C(=O)C)C(=C)C1=O	5.3	Poor	7.3
14257	O1C2OC(=O)C3C2C2(C(CC3)C3(C(CC2O)C(CCC3)(C)C)C)C1O	4.3	Excellent	7.3
9979	O=C1C2N(C(=O)C1=C(O)C)C(C1C2C2c3c(C1)cccc3N(C)C2=O)(C)C	5.0	Excellent	7.3
7289	O1C2CC(=O)CC(CC=C3C(O)C(OC3=O)CC1(CC2=O)C)C(C)=C	5.4	Excellent	7.3
6784	O=C1NC(C=2N(c3c1cccc3)C(=O)c1c(N=2)cccc1)C	5.0	Excellent	7.3
1010	O1C(=O)C(CCC1C(C)C1CCC2C3C(CCC12C)C1(C(CC(=O)CC1)CC3)C)C	5.6	Good	7.3
486	ClC1CC2C(c3c4c(cccc4[nH]c3)C2(C)C)C([NH+]=[CH-])C1(C)C1OC1	4.2	Poor	7.3
12947	O(C(=O)C)C1C2C(C3CCC(C(CCC(O)=O)C)C3(C1)C)CCC1=CC(=O)CCC12C	3.7	Poor	7.3
10086	OC1CC2=CCC3C4CCC(C(CC=CC(O)(C)C)C)C4(CCC3C2(CC1)C)C	5.1	OK	7.3
8235	OC1CC2=CC(=O)C3C4CCC(C(CC#CC(O)(C)C)C)C4(CCC3C2(CC1)C)C	4.5	Poor	7.3
8022	O1C(O)C(=CC1=O)C(O)CC1C2(C(CCC1=C)C1(C(CC2)C(CCC1)(C)C)C)C	4.9	OK	7.3
6548	O1C(OC)C2=C(CCC3C4(C(CCC23C)C(CCC4)(C(O)=O)C)C)C1=O	5.4	Excellent	7.3
5218	O1CC2=C(CC(OC(=O)C)C3C2(C)C(OC(=O)C)CC2C(CCCC23C)(C)C)C1=O	5.4	OK	7.3
4838	ClC1=C(O)C(=O)c2c(cc3c(c2)C2(CCCc4c2c(oc4)C3=O)C)C1=O	5.1	Excellent	7.3
4276	O(C)c1ccc(cc1)C(=O)Cn1cc(c(-c2ccc(O)cc2)c1C(OC)=O)-c1ccc(O)cc1	5.0	Poor	7.3
3402	Brc1c2c(C(=O)C=3OC(C4C(C=3C2=O)C=C(CC4)C)(CCC=C(C)C)C)c(O)cc1O	5.0	Good	7.3
1006	O1C(=O)C(C)=C(CC1C(C)C1CCC2C3C(CCC12C)C1(C(=CC(=O)C=C1)CC3)C)C	3.9	Bad	7.3
869	BrC1=CC23C4=C(NC(SC2CC1=O)C3)C(=O)c1[nH]cc2CCN=C4c12	5.2	Excellent	7.3
11857	O1C2=CC(=O)C(O)=CC2=CC2C3(C(CCC12C)C(CCC3)(C)C)C	5.4	Poor	7.3
11601	O1C2(CCC=C(CCC(=CC=C(CCC2O)C)C(C)C)C1=O)C	4.9	Excellent	7.3
11090	O1C2C(CC3OC3(CCC=C(CCC=C(C)C2=O)C)C)C(=C)C1=O	5.3	Poor	7.3
7491	O(C(=O)C)C1CCC2(C(CCC3(C2CC(=O)C3=C(C=O)C)C)C1(C(O)=O)C)C	6.0	Bad	7.3
5994	O1C2CCC1(C)C(OC(=O)C)CC1C(OC(=O)C1=C)C(O)C(CCCC2=C)C	4.3	Poor	7.3
2913	OC12C=C(C3C4(C1NCCCCC=CCC4)CN(CC3)CCCCC=CCC2)C=O	3.0	Poor	7.3
1155	BrC1=CC23C4=C(NC(SC2CC1=O)C3)C(=O)c1[nH]cc2c1c4ncc2	5.1	Excellent	7.3
13812	O=C1CC2C(CCC3C(C)(C)C(=O)CCC23C)(C)C1=C(C=CC=C(C(O)=O)C)C	6.0	Good	7.2
5104	O1C2C(=CC1=O)C(O)CC1C2(C)C(OC(=O)C)CC2C1(CCC1C(CCCC12C)(C)C)C	5.1	Good	7.2
3831	O1C2(C=CC1(O)C(=CC1C(CC2OC(=O)Cc2ccccc2)C(=CC(=O)C1C(C)C)C)C)C	4.7	Poor	7.2
3001	O=C1C=C(c2nc3c(c4c2c1ncc4)cccc3)C(OC)CNC(=O)C(=CC)C	3.8	Poor	7.2
2070	O1C2n3c4c(c5c(CNC5=O)c5c6c(n(c45)C1(C)C(OC)C(N(C)C)C2)cccc6)c1c3cccc1	5.7	Poor	7.2
13184	O1C2c3oc(CC(CCC45OC4C(OC5=O)CC12C)C(C)=C)c(c3)C=O	5.1	Poor	7.2
5443	OC12N(C(c3[nH]c4c(c3C1=O)cccc4)C=C(C)C)C(=O)C1N(CCC1)C2=O	5.7	Excellent	7.2
11745	O1C2C(CC(O)C(CCC(=CCCC(=C2)C)C)C(=O)C)C(=C)C1=O	5.4	Excellent	7.2
6792	O1C23C(OC(O)(CC2)C1(C)C)CCC(C)C3(CCC=1C2(C(OC(C)(C)C(=O)CC2)CCC=1C)C)C	5.0	Poor	7.2

3455	O1C=C(C2C(C(CCC=C(CC2)C)=C)C1OC(=O)C)C(OC(=O)C)CC=C(C(OC)=O)C	5.8	Poor	7.2
3164	S(Oc1c2c(cc3c(c2)C2(CCCc4c2c(oc4)C3=O)C)c(O)cc1)(O)(=O)=O	6.3	OK	7.2
8934	O1C2CCC(=C)C(CCC3C4(C(OC(C)(C)C(=O)CC4)CCC3(O)C)C)C2(CCC(O)C1(C)C)C	3.5	Poor	7.2
4827	O1C2(CCC(CC(O)C3(OC(CC3)C(O)(CCC2=O)C)C)C(=C)C1=O)C	4.9	Poor	7.2
2910	OC1CC2=CCC3C4CCC(=NO)C4(CCC3C2(CC1)C)C	5.0	Good	7.2
1123	O1CC(C(COC(=O)C)C(O)C1=O)C1(C2C(CC1)C(CCCC2=C)(C)C)C	5.6	Good	7.2
13492	O1C2CCC(O)(C)C(CCC3C(=C)C(=O)CC4C(CCC4(O)C)C3(C)C)C2(CCC(=O)C1(C)C)C	5.2	Poor	7.2
10654	O1C(CC(=CCCC(C=CC=C(CCCC2=CC(=O)N(C2)CC(O)=O)C)C)C)C(O)=C(C)C1=O	5.5	Bad	7.2
10087	OC1CC2=CCC3C4CCC(C(CC=CC(O)(C)C)C)C4(CCC3C2(CC1)C)C	4.3	Bad	7.2
6633	Oc1cc2c(N3C(=Nc4c(cccc4)C3=O)C(NC2=O)C)cc1	5.3	Excellent	7.2
6583	O(C)c1cc2C3=NC(CC(=O)C)C(c4c([nH]c5c4cccc5)C3=Nc2cc1)CC(=O)C	5.0	Poor	7.2
459	O1C2OC(=O)CC2C(C(=CC2(CC(CCC2)(C)C)C)C=C)C1OC(=O)C	6.0	Good	7.2
14259	O1C2OC(=O)C3C2C2(C(CC3)C3(C(CC2OC(=O)CCC)C(CCC3)(C)C)C)C1O	4.4	Excellent	7.2
12229	O1C2=CC(O)(CCC(OC(=O)CC(C)C)C3(C(CC2=C(C)C1=O)C(=CCC3OC(=O)C)C)C)C	4.4	Excellent	7.2
12103	O1CC2=C(C3(C(CC2)C2(C(CC3O)C3(C(CC2)C(CCC3)(C)C)C)C)C)C1=O	4.9	Bad	7.2
6995	O1C(O)C2=C(CCC3C4(C(CCC23C)C(CCC4)(C(O)=O)C)C)C1=O	4.2	OK	7.2
4062	[nH]1c2c(CCN=C2c2c3c([nH]c2)cccc3)c2c1cccc2	5.2	Poor	7.2
3317	O1C(CC=C(CCC=2C3(C(CCC=2C)C(CCC3)(C)C)C)C1O)C1=CC(OC1O)=O	4.1	Excellent	7.2
1657	O1C2C(CCC3C(CCCC23C)(C)C)(C)C(COC(=O)C)C(CC(OC)=O)C1=O	5.8	Good	7.2
1596	S1CC(N(CO)C1=O)C1(OC2CCC(C=CCCC(=CC(OC(C1)C2)=O)C)C)O	4.6	Excellent	7.2
666	S=C=NC1(C2C(C(CC1)C1(OC(CC1)C([NH+]=[CH-])(C)C)C)C([NH+]=[CH-])C(O)(CC2)C)C	5.1	OK	7.2
13941	O1C2OC(=O)CC2C(C2(C3C(CC2)C(CCCC3=C)(C)C)C)C1OC(=O)C	4.9	Bad	7.2
12503	O(C(=O)C)C1C2C3C(CCC2(C)C(C1)C=C)C1(C(=CC(=O)C=C1)CC3)C	4.6	Good	7.2
9192	O1C2CC3C4C(C)(C(=O)CC3(CC(=O)C12C)C)C(=O)C=C4C(C)C	6.5	Excellent	7.2
7377	Oc1ccc(cc1CC1(C2CCC=C(C)C2(CCC1C)C)C)C(OC)=O	4.9	OK	7.2
6691	O1C2C(CC3OC2C(CCCC(=CCCC3(O)C)C)C)C(=C)C1=O	6.0	Excellent	7.2
5625	O(C(=O)C1C2(CCC3(C(CCC=C3C)C2(CCC1C)C)C)C)CC(O)COC(=O)C	3.9	Bad	7.2
5126	O=C=NC1(C2C3C(CC1)C(CC1CC(C)C(C(C13)CC2)(C#N)C)C)C	4.6	Poor	7.2
4310	O1C2C3C(C1CC(O)(C)C(O)CCC2(OC(=O)CCC)C)C(CCC3C(C)C)=C	5.7	OK	7.2
3834	O1C2(C=CC1(O)C(=CC1C(CC2OC(=O)C=C(C)C)C(=CC(=O)C1C(C)C)C)C)C	5.1	Good	7.2
3499	OC1CC2=CC(=O)C3C4CCC(C(CCC(C(O)(C)C)=C)C)C4(CCC3C2(CC1)C)C	6.3	Bad	7.2
3399	O1CC(C2CC(C)C(=O)C3C2C2OC3CC(=CCC(OC(=O)CCCCCCC)C12C)C)C	5.1	Poor	7.2
1416	O1CC2(C3C(=O)C(C4C(CC(=CC4=O)C)C3(C=C3N4C5(OC(CC(C5)C)C4)CCC23C)C)C)CC1=O			7.2
1079	O(C(=O)C1C2(CC=C3C(CCCC3(C)C)C2(CCC1C)C)C)CC(O)COC(=O)C	5.8	OK	7.2
919	O=C1C=C(c2nc3c(c4c2c1ncc4)cccc3)CCNC(=O)C(=CC)C	5.9	Good	7.1
13330	O=C1C=2C(=CC(=O)N(C=2)C)c2nc3c(c4c2c1ncc4)cccc3	5.1	Excellent	7.1
6443	Oc1c2c(ncc1)-c1nc3c(c4c1c(ncc4)C2=O)cccc3	5.2	Excellent	7.1
11977	O1C2C3C(C1CC(=CCCC2(OC(=O)C)C)C)C(C)=C(OC(=O)C)CC3C(OC(=O)C)(C)C	5.6	Excellent	7.1
8908	s1c2c(nc1)c1nccc3-c4c(Nc(c13)c2CCNC(=O)C(C)C)cccc4	4.4	Poor	7.1
8120	O1c2c3c(cccc3O)c(O)c3c2-c2c(c(ccc2OC3=O)C)C1=O	5.1	Excellent	7.1
3498	OC1CC2=CC(=O)C3C4CCC(C(C=CC(=O)C)C)C4(CCC3C2(CC1)C)C	5.7	Poor	7.1
2853	s1c2c3cc(n4c3c3c(c5c4cccc5)ccnc3c2nc1)N(C)C	5.3	Excellent	7.1
2680	O1CC(C2CC(C)C(OC(=O)C)C3C2C2OC3CC(=CCCC12C)C)C	5.0	Good	7.1
1127	O1C2OC(OC(=O)C)C(C2C2(CCC3=C(CCCC3(C)C)C2C)C)C(OC(=O)C)C1=O	5.4	Bad	7.1

14124	O1C23C1CC1C(C2(C)C(=O)C=CC3)C(O)CC2(C1CCC2C(C=CC(C(C)C)C)C)C	4.5	Poor	7.1
12766	O1C2CC(=O)CC(CC=C3C(O)C(OC3=O)CC1(CC2=O)C)C(C)=C	4.7	Good	7.1
11471	O=CNC(CC1C2C3C(CCC(C)C3([NH+]=[CH-])CCC2=C)C(C1)C)(C)C	4.7	Excellent	7.1
8832	OC12C3=C(C(=O)C(O)=C1C)C(CCC3C(CC2C=C(C)C)C)C	6.0	Excellent	7.1
8673	O1C2CC3(C(CCC(=C)C(OO)CCC12C)C(=C(C)C)C(=O)C3)C	5.3	Excellent	7.1
3062	O1C2=C3C(C1)C(O)CCC3(c1c(cc3c(c1)C(=O)CCC3O)C2=O)C	5.3	Bad	7.1
2722	O1CC(C2(C3C(CC2OC(=O)C)C(CCCC3=C)(C)C)C)C(CC1=O)COC(=O)C	5.3	Poor	7.1
1535	OC1=C(CC2(C3CCCC(=C)C3(CCC2C)C)C)C(=O)C(NCCc2ccccc2)=CC1=O	5.3	Good	7.1
245	O1C2C(CC=C(CCC=C(CCC=C(C2)C)C)COC(=O)C)C(=C)C1=O	5.4	OK	7.1
13045	O1CC12C1OC3C=C(CCC3(COC(=O)C=C(CCO)C)C2(C)C(O)C1)C	4.5	OK	7.1
12033	N#CC1(CC2CC(C3C4C(CCC(C24)C1C)C(CC3)(C#N)C)C)C	5.7	Good	7.1
10665	O1C2(CCC(CC3OC3(CCC=C(CCC2=O)C)C)C(=C)C1=O)C	5.4	Excellent	7.1
7486	O(C(=O)C)C1CCC2(C(CCC3(C2CC(=O)C3=C(C=O)C)C)C1(C(OC)=O)C)C	5.9	OK	7.1
6599	O(C(C)c1c2nc3c(nc2ccc1)c(ccc3)C(O)=O)C(=O)c1ccccc1	6.0	Bad	7.1
6134	O1C(O)C(=CC1=O)C(OC(=O)C)CC1(C=2C(CCC1C)C(CCC=2)(CCCC(C)=C)C)C	4.9	Poor	7.1
4793	O1c2c(cc(O)c(O)c2)C(=O)C2C3(C(CCC12C)C(CCC3)(C)C)C	5.6	Good	7.1
2345	s1c2c(nc1)c1nccc3-c4c(Nc(c13)c2CCNC(=O)CC(C)C)cccc4	4.9	Good	7.1
1279	O1C2CC(CC(=O)C=C(C)C(=O)CC(CCC(=C2)C1=O)C(C)=C)=C	5.0	Good	7.1
14447	O1C2C=C(CCC(C(C)=C)C(OC(=O)C)c3oc(cc3C)C2C(C)=C)C1=O	5.7	OK	7.1
12431	O1C(O)C2C(=CCC3C4(C(CCC23C)C2(C(CC4)C(CCC2)(C)C)COC(=O)C)C)C1=O	5.8	Good	7.1
10923	O(C(=O)C=1c2n(c3c(c2C=C(c2n(c4c(c2C=1)cccc4)C)C(OC)=O)cccc3)C)C	4.9	OK	7.1
10838	O1C2C(CC3OC3(CCC=C(CCC=C(C2)C)C)COC(=O)C)C(=C)C1=O	5.6	Poor	7.1
9690	O1C2C(CC(O)C(=CCCC(=CCCC(=C2)C)C(OC)=O)C)=C(C)C1=O	4.4	Poor	7.1
8447	O1C2C(CC3OC3(CCC=C(CCCC(C)C2OC(=O)C)C)C)C(=C)C1=O	4.5	Excellent	7.1
6998	O1C(O)C2=C(C3(C(CC2)C2(C(CC3)C(CCC2)(CO)C)C)C)C1=O	5.0	Good	7.1
4244	O(CC1C2(C(CC=C1C=O)C1(C(CC2)C(CCC1)(C)C)C)COC(=O)C)C(=O)C	5.6	Poor	7.1
2105	O(C(=O)C)c1c(CC2(C3CCC=C(C)C3(CCC2C)C)C)c(O)ccc1O	5.3	Poor	7.1
997	O1C2CCC(O)(C=CCC(=CC3OC(=O)C(C3CC(OC(=O)C)C12C)=C)C)C	5.2	Excellent	7.1
14477	O1C2C(CCC(=CCCC(O)(C)C(O)CCC(=C2)C)C)=C(C)C1=O	4.2	Excellent	7.1
13728	O(C(=O)C)CC(c1c2c([nH]c1)cccc2)c1c2c([nH]c1)cccc2	4.4	Good	7.1
13364	O1C2C=C(CC(=O)C=C(CCC=C(CC(OC(=O)C)C2C(C)C)C)C)C1=O	5.3	Poor	7.1
6266	O1C2C=C(CCC(C(C)=C)C(O)C(=O)C(=CC(=O)C3CC23C)C)C1=O	5.4	Poor	7.1
5669	O1CC2C(CC(OC(=O)C)C3C2(C=O)C(OC(=O)C)CC2C(CCCC23C)(C)C)C1=O	5.4	Poor	7.1
5622	O(C(=O)C=C(CCC1C2(C(CCC1=C)C(CCC2)(C)C)C)C)CC(O)COC(=O)C	3.5	Bad	7.1
4792	Clc1c2C=C3C4(C(CCC3(Oc2cc(O)c1O)C)C(CCC4)(C)C)C	3.8	OK	7.1
4757	O1C=C(C2C(C(CC=CC(OC(=O)C)(CC2)C)=C)C1OC(=O)C)C(=O)CC=C(C)C	4.1	OK	7.1
3657	O1C(C2C(C=CCC=CCC=CCCCC1=O)C(O)C(O)C2)CC	4.7	Bad	7.1
2479	O1C2c3oc(CC(CCC4=CC(OC4=O)CC12C)C(C)=C)c(c3)C=O	5.4	Excellent	7.1
1014	O1C(=O)C(C)=C(CC1C(C)C1CCC2C3C(CCC12COC(=O)C)C1(C(=CC(=O)C=C1)CC3)C)C	5.3	Bad	7.1
13771	O1C2(OC)C3=C(CCC(CCC(CC(O)C=C(C2)C)=C)C3(C)C)C1=O	4.9	Excellent	7.1
13069	O(C(=O)C)C1CC(C2CC=C(C(C1C(CCC=C(C)C)C)C2OC(=O)C)C=O)=C	4.7	Poor	7.1
8605	OC12CC(O)CCC1(C1C(C3CCC(C(CCC(C(COC(=O)C)C)=C)C)C3(CC1)C)CC2=O)C	5.2	Bad	7.1
8555	OC1(CCC2C3C(CCC12C)C1(C(CC(O)CC1)=CC3)C)C(=O)C	3.6	Excellent	7.1
6070	OC1CC2=CCC3C4CCC(C(CCC(OC)=O)C)C4(CCC3C2(CC1)C)C	4.7	Poor	7.1
6049	O1C2C(=CC1=O)C(OC(=O)C)CC1C2(C)C(O)CC2C1(CCC1C(CCCC12C)(C)C)C	4.5	Bad	7.1
5959	O1CC2=C(C3(C(CC2)C2(C(CC3O)C3(C(CC2)C(CCC3)(CO)C)C)C)C)C1=O	3.8	Excellent	7.1
5756	OC1C2C3CCC(C(CCCC(CO)C)C)C3(CCC2C2(C(C1)=CC(=O)CC2)C)C	5.5	Bad	7.1

4297	S(C)C=1C(=O)c2nccc3c2c(nc2c3cccc2)C=1CCNC(=O)CC(C)C	4.4	Poor	7.1
3783	O1C2C3C(C1CC(=C)C(=O)CCC2(OC(=O)C)C)C(OC(=O)C)(CCC3C(C)C)C	5.7	Bad	7.1
3338	O1C(CC=C(CCC=2C3(C(CCC=2C)C(CCC3)(C)C)C)CO)C(=CC1=O)CO	4.1	Poor	7.1
2938	O1C(=O)C(CCC2C3(C(CCC12C)C(CCC3)(C)C)C)=CCC(OC(=O)C)C1=CC(OC1)=O	4.5	Bad	7.1
2519	O1C2C3C(CCC3(CCC(=CC(=O)CC(C2)C)C)C)=C(C)C1=O	4.8	Good	7.1
2381	O1C2OC(=O)CC2C(C2(C3C(CC2)C(CCCC3=C)(C)C)C)C1O	4.0	Good	7.1
2104	OC1=CC(=O)C=C(CC2(C3CCC=C(C)C3(CCC2C)C)C)C1=O	4.9	Good	7.1
1020	ClC1=CC(O)(C2Cc3c(occ3C)CC(=CCCC2(C)C1=O)C)C	5.3	Excellent	7.1
1019	o1cc(c2CC3C(CCC=C(Cc12)C)(C)C(=O)C=CC3(O)C)C	4.9	Excellent	7.1
12945	Oc1cccc(C)c1C(OC(C)c1c2nc3c(nc2ccc1)c(ccc3)C(O)=O)=O	5.8	OK	7.0
12346	O1C2C(CC(O)C(CCC(=CCCC(=C2)C)C)C(=O)C)C(=C)C1=O	5.6	OK	7.0
5889	OC1C2=CC(=O)CCC2(C2C(C3CCC(C(CC=CC(OO)(C)C)C)C3(CC2)C)C1)C	5.0	Bad	7.0
5220	O1CC2(C3C(CCC2C1=O)C1(C(CC3O)C(CCC1)(C)C)C)C	4.7	Excellent	7.0
1666	O=C1C2C(CCC(C=C)(C)C2[NH+]=[CH-])C(c2c1c(NC=O)ccc2)(C)C	3.7	OK	7.0
14185	O1C2c3oc(CC(CCC45OC4C(OC5=O)CC12C)C(C)=C)c(c3)C(OC)=O	4.2	Good	7.0
12773	O(C(=O)C)C1CC(C(=CCC=C(C)C)C)C(C2C(C)C(O)CC2=O)C1C=O	6.1	Excellent	7.0
12391	O1C2C(CC3OC3(CCC=C(CCC=C(C)C2O)C)COC(=O)C)C(=C)C1=O	6.3	Excellent	7.0
12079	O1C2CCC(=CCCC(=CC3OC(=O)C(C3CC(OC(=O)C)C12C)=C)C)C	4.1	Good	7.0
11944	O1CC(C2CC(C)C(OC(=O)CCC)C3C2C2OC3CC(=CCC(OC(=O)C)C12C)C)C	4.7	Excellent	7.0
11844	ClC=1C=2C(OC3(C(C=2)C2(C(CC3)C(CCC2)(C)C)C)C)=CC(=O)C=1O	4.6	Bad	7.0
7414	O1c2c(C(CCC3(C)C(CCC=C3C)C)(C)C1C)c(O)cc1c2C(=O)C(C)=C(O)C1=O	4.6	OK	7.0
7263	OC1=C(C)C(=O)C23C4(CC(=CC2CC(C3CCC4C)C)C)C1=O	4.4	Good	7.0
6249	O1C23C=4C(CCC2(C)C(CC13)C(C=CC(C(C)C)C)C)C1(C(CC(=O)CC1)C(=O)C=4)C	4.5	Bad	7.0
5670	O1CC2C(=CC(OC(=O)C)C3C2(C=O)C(OC(=O)C)CC2C(CCCC23C)(C)C)C1=O	6.0	Good	7.0
3784	O1C2C3C(C1C=C(C)C(=O)CCC2(OC(=O)C)C)C(OC(=O)C)(CCC3C(C)C)C	6.0	OK	7.0
3639	O1C2C(CC3OC3(CCC=C(CCCC(C)C2=O)C)C)C(=C)C1=O	4.3	Excellent	7.0
2106	O(C(=O)C)C1=C(CC2(C3CCC=C(C)C3(CCC2C)C)C)C(=O)C=CC1=O	4.9	Excellent	7.0
1658	O1C2OC(=O)CC2C(C2(C3C4(C(CC2)C(CCC4)(C)C)C3)C)C1OC(=O)C	4.5	Excellent	7.0
11413	O1C2=C(CC13C1(C(CCC3C)C(CCC1)(C)C)C)C(=O)C(OC)=CC2=O	4.9	Poor	7.0
11393	O(C(=O)C(=O)c1c-2n(C=Cc3c-2[nH]c2c3cccc2)c2c1cccc2)CC	4.2	Poor	7.0
7944	Oc1c2N=C3N(c4c(cccc4)C(=O)NC3C)C(=O)c2ccc1	5.1	Excellent	7.0
7417	O1C2=C(C=3C(CCC(OC)(C=3)C)C1(CCC=C(C)C)C)C(=O)c1c(C2=O)c(O)cc(O)c1	5.3	Poor	7.0
7273	O1C2(O)C3(C(C4C5(C(CCC(C)C5(O)C2=O)C(C4)C)C3=O)C1(C)C)C	5.2	OK	7.0
5673	Oc1ccc(O)cc1C1C2=C(CCC(C)C1=C)C(CCC2)(C)C	4.6	Excellent	7.0
3165	O1C2=C3C(C1)C(O)CCC3(c1c(cc3c(c1)C(O)CCC3=O)C2=O)C	5.0	OK	7.0
2679	O1CC(C2CC(C)C(OC(=O)CCC)C3C2C2OC3CC(=CCCC12C)C)C	4.7	Excellent	7.0
2520	O1C2C3C(CCC3(CCC(CC(=O)CC(C2)C)=C)C)=C(C)C1=O	5.2	Good	7.0
1624	Oc1ccc(cc1CC1(C2CCCC(=C)C2(CCC1C)C)C)C(OC)=O	4.8	Good	7.0
633	ClC1CC2C(C([NH+]=[CH-])C1(C=C)C)C(=O)c1c(cccc1NC=O)C2(C)C	4.9	Excellent	7.0
14455	O(C)c1c2c(cc(C)c1C(=O)C)cc1c(C(=O)C(C)(C)C(=O)C1(C)C)c2O	4.6	Poor	7.0
14114	O=C1N(Cc2ccccc2)C(=O)N(c2nccnc12)Cc1ccccc1	5.8	OK	7.0
6937	O=C1C23C(CCC2(C)C(CC1)C(C=CC(C(C)C)C)C)C1(C(=CC(=O)CC1)C(=O)C3)C	2.9	Bad	7.0
5071	O1C2C3C(C1CC(=C)C(N(C(=O)C)C)CCC2(OC(=O)C)C)C(OC(=O)C)(CCC3C(C)C)C	5.0	Bad	7.0
4982	O1C2CCC(C)C(CCC3=C(C)C(=O)CC4C(C)(C)C(O)CCC34C)C2(CCC(=O)C1(C)C)C	5.0	Bad	7.0
3769	O1C2=CC(=O)C(=O)C=C2C=C2C3(C(CCC12C)C(CCC3)(C)C)C	4.1	Bad	7.0
3394	O1C23C(CCC(C)C2(CC2=C1C(=O)C=C(OC)C2=O)C)C(CCC3)(C)C	4.4	Excellent	7.0

2294	O(C(=O)C(=O)c1c-2n(C=Cc3c-2[nH]c2c3cccc2)c2c1cccc2)C	4.0	Poor	7.0
949	ClC1C2OC(=O)C(C)C2(O)C(O)C2C(C=CC(=O)C2C)(C)C(OC(=O)C)CCC1=C	6.3	Excellent	7.0
448	O1CC2C3C(CCC2C1=O)C1(C(CC3COC(=O)C)C(CCC1)(C)C)C	6.4	Excellent	7.0
13943	O1C2C(CC3C(C)(C(OC(=O)C)CCC(=C2)C)C(OC(=O)C)CC=C3C)=C(C)C1=O			7.0
12996	O1C2CCC(=CCC(CCC(=CC(OC(=O)C)CC12C)C)C(OC(=O)C)(C)C)C	4.5	Bad	7.0
12851	O=C1CC2C(C3CC=C(C=O)C(C=O)C13C)(CCC1C(CCCC12C)(C)C)C	4.3	Bad	7.0
12081	O1C(CC=C(CCC=C(CCC=2C(CCCC=2C)(C)C)C)C1O)C1=CC(OC1OC(=O)C)=O	6.7	Bad	7.0
10292	n1cccc(c1-c1cccnc1)-c1cc(ncc1)-c1cccnc1	6.1	Poor	7.0
9943	O1C2(CCC(CC3OC3(CCC=C(CCC2O)C)C)C(=C)C1=O)C	4.8	Excellent	7.0
9929	O(C(=O)C)CC(NC(=O)C(NC(=O)c1ccccc1)Cc1ccccc1)Cc1ccccc1	4.1	Poor	7.0
7379	Oc1c(cc(cc1O)C(OC)=O)CC1(C2CCC=C(C)C2(CCC1C)C)C	4.1	OK	7.0
4189	O(C(COC(=O)c1ccccc1)c1cc(ccc1)CC)C(=O)c1ccccc1	4.5	Poor	7.0
3322	O1CC(CC1=O)C(O)CC=C(CCC=1C2(C(CCC=1C)C(CCC2)(C)C)C)CO	3.9	Good	7.0
3249	o1ccc(C(C)C)c1CC(C(O)=O)C1CCC2C3C(CCC12C)C1(C=C(O)C(=O)CC1CC3)C	4.9	Bad	7.0
2564	OC12CC(O)CCC1(C1C(C3CCC(C(C=CCC(C)C)C)C3(CC1)C)=CC2=O)C	3.8	Good	7.0
2346	s1c2c(nc1)c1nccc3-c4c(Nc(c13)c2CCNC(=O)CC)cccc4	4.9	Good	7.0
1418	O1CC2(C3C(=O)C(C4C(CC(=CC4=O)C)C3(C=C3N4C5(OC(CC(C5)C)C4)CCC23C)C)C)CC1=O			7.0
909	O1C2C3C(CCC3(CC=C(CC(=O)CC(C2)C)C)C)=C(C)C1=O	4.7	Poor	7.0
12767	O1C2CC(=O)CC(CCC3=CC(OC3=O)CC1(CC2=O)C)C(C)=C	5.4	Excellent	7.0
12230	o1cc(c2CC3C(C)(C(OC(=O)C)C(=O)CC(=Cc12)C)C(OC(=O)C)CC=C3C)C	5.5	OK	7.0
11491	O1CC2C(CCC(=CC(O)CC2=C)C)C(=CC=CC(O)(C)C)C1=O	6.3	Excellent	7.0
11163	Clc1ccc(cc1)C(C(C)C)C(OC(C#N)c1cc(Oc2ccccc2)ccc1)=O	5.3	Bad	7.0
9823	O1C2CCC1(C)C(O)C1OC(=O)C(O)(C1C(=O)CC(=CCCC2C)C)C	6.4	Excellent	7.0
8792	O1CC(=CC1=O)C(O)CC=C(CCC1(C2CCCC(=C)C2(CC(O)C1C)C)C)C	4.8	Bad	7.0
8395	ClC1CC(C2C(C3CC(OC3(CC2=O)C)C2CC(=O)NC2=O)(C1)C)(C)C	4.3	OK	7.0
5781	S(OCC(CCCC(C)C1CCC2C=3C(CCC12C)C1(C(CC(O)CC1)C(=O)C=3)C)C)(O)(=O)=O	5.1	Poor	7.0
5638	OC1(CCC2C3C(CCC12C)C1(C(CC(O)CC1)=CC3)C)C(=O)C	5.1	Excellent	7.0
4981	O1C2CCC3(OC3)C(CCC3=C(C)C(=O)C(O)=C4C(C)(C)C(=O)CCC34C)C2(CCC(=O)C1(C)C)C	5.0	Good	7.0
4229	o1cc2c(CCC3C2(CCC2C(C)(C)C(=O)C(O)CC23C)CO)c1	4.4	Good	7.0
3400	O1CC(C2CC(C)C(OC(=O)CCC)C3C2C2OC3CC(CCC(=O)C12C)=C)C	4.8	Good	7.0
3291	OC1CC2=CC(O)C3C4CCC(C(C=CCC(C)C)C)C4(CCC3C2(CC1)C)C	5.4	Poor	7.0
2985	O1C(CC=C(CCC=C(CCC=2C(CCCC=2C)(C)C)C)C1O)C1CC(OC1)=O	4.3	OK	7.0
1482	O1C2OC(=O)CC(C2C2(C3C4(C(CC2)C(CCC4)(C)C)C3)C)C1OC(=O)C	5.7	Bad	7.0
1125	O1C2OC(=O)CC(C2C2(CCC3=C(CCCC3(C)C)C2C)C)C1OC(=O)C	6.0	Excellent	7.0
11468	O(C(=O)C)C1CC2C(C=C(C)C(=O)C=CC1(O)C)C2(CCC=C(C)C)C			7.0
11460	O(C(=O)C)C1CC2C(C=C(C)C(=O)C=CC1(O)C)C2(CCC=C(C)C)C			7.0
11414	O1C2=C(C=C3C4(C(CCC13C)C(CCC4)(C)C)C)C(=O)C(OC)=CC2=O	4.6	OK	7.0
10797	Oc1ccc(O)cc1CC1C2(C(CC=C1C)C(CCC2)(C)C)C	4.7	Excellent	7.0
9716	O1C23C1(C1CCC(C(CCCC(C)C)C)C1(CC2)C)CC(O)C1CC(O)CCC13C	5.1	Poor	7.0
7316	O1C2C(CC3C(C)(C(OC(=O)C)C(=O)C=C3C)C(OC(=O)c3ccccc3)CCC(=C2)C)=C(C)C1=O	6.0	Poor	7.0
6323	OC1CC2=CC(=O)C3C4CCC(C(CCC(C(C)C)=C)C)C4(CCC3C2(CC1)CO)C	4.7	OK	7.0
6011	O(C(=O)C)C1CC2C(CCCC2(C2CCC(C(OC)=O)C(C=O)C12C)C)(C)C	4.8	Poor	7.0
5995	Brc1cc2NC(=O)C(c2cc1)=C1Nc2c(cccc2)C1=O	4.3	OK	7.0
5216	O1CC2(O)C(=CC(OC(=O)C)C3C2(C)C(OC(=O)C)CC2C(CCCC23C)(C)C)C1=O	4.8	Good	7.0
5127	O=C=NC1(C2C3C4C(CC2)C([NH+]=[CH-])(CCC4C(CC3CC1C)C)C)C	4.4	Poor	7.0

4820	ClC1C(=CC2C3(C(CCC2(O)C)C(CCC3)(C)C)C)C(O)=CC1=O	5.5	OK	7.0
4682	O1C2C(CC3OC3(CCC(O)C(CCCC(C)C2OC(=O)C)=C)C)C(=C)C1=O	4.4	Bad	7.0
4491	O1c2c(CCC1(CC=1CC3(CCCC3(C)C(=O)C=1CC(O)(C)C)C)C)c(O)c(cc2OC)C	4.3	Bad	7.0
4227	o1cc2c(CCC3C2(CCC2C(C)(C)C(O)C(=O)CC23C)CO)c1	5.3	Good	7.0
3796	O(C)C1=CC(=O)C(O)=C(CC2C3(C(CCC2=C)C(CCC3)(C)C)C)C1=O	4.4	Excellent	7.0
3104	O1C2C3C(CC1=O)C(OC(=O)C)CCC3(C)C(C2)C(CCC=C(C)C)C	4.8	OK	7.0
2218	O=C1c2c(N3C1=Nc1c(cccc1)C3=O)cccc2	4.4	Good	7.0
908	OC1=C(CC2(C3CCCC(=C)C3(CCC2C)C)C)C(=O)C(N)=CC1=O	5.3	Poor	7.0
163	O1C(OC(=O)C)c2c(ccc(C3(CC(CCC3)(C)C)C)c2C(=O)C)C1=O	5.8	Good	7.0
1534	OC1=C(CC2(C3CCCC(=C)C3(CCC2C)C)C)C(=O)C(O)=CC1=O	6.1	Excellent	6.9
13405	O1C2C3C(C1CC(O)(C)C(OC(=O)CCC)CCC2(OC(=O)C)C)C(=C)C(O)CC3C(C)C	4.1	Poor	6.9
9778	O1C2C3(C(C4C5(C(CCC(C)C5(O)C2=O)C(C4)C)C3=O)C1(C)C)C			6.9
7083	S=C=NC(CC1C2C3C(CCC2=C)C([NH+]=[CH-])(CCC3C(C1)C)C)(C)C	5.1	OK	6.9
5131	S=C=NC(CC1C2C3C(CCC2=C)C([NH+]=[CH-])(CCC3C(C1)C)C)(C)C	4.9	Poor	6.9
3933	O1C2CC(=O)CC(CC(CC=C3C(O)C(OC3=O)CC1(CC2=O)C)C(C)=C	5.9	Excellent	6.9
3912	O1C2CC(=O)CC(CC(CC=C3C(O)C(OC3=O)CC1(CC2=O)C)C(C)=C	5.1	Excellent	6.9
1785	O1C2C3OC(CC(=O)C=C(C)C(=O)CC(CCC3(O)C1=O)C(C)=C)(C2)C	4.7	Excellent	6.9
1430	O1C2C3C(C1CC(=C)C(=O)CCC2(OC(=O)CCC)C)C(CCC3C(C)C)=C	5.1	Excellent	6.9
626	O1C2OC(=O)C3C2C2(C(CC3)C3(C(C(CCC3)(C)C)C(OC(=O)C)C2)C)C1OC(= O)C	5.9	OK	6.9
12875	O1C=C(C2C(C(CCC=C(CC2)C)=C)C1OC(=O)C)C(OC(=O)C)C(OC(=O)C)C=C(C)C	6.2	Bad	6.9
10774	O1C2(C3C(CCC3C)C(=CC2C=C(CCCc2ccoc2)C)C)C(=O)C(C)=C1O	5.0	OK	6.9
9821	O1C2CCC1(C)C(O)C1OC(=O)C(=C1C(=O)CC(=CCCC2C)C)C	3.9	Excellent	6.9
8040	ClC1C(C2CC(OC(=O)C)C(=C)C(CC(O)C3CC(=O)NC3=O)C2(CC1Cl)C)(C)C	4.8	Bad	6.9
7415	Brc1c(O)c2c(cc1O)C(=O)C1=C(OC(C3C1C=C(CC3)C)(CCC=C(C)C)C)C2=O	4.5	Poor	6.9
5888	OC1C2=CC(=O)CCC2(C2C(C3CCC(C(CCC(OO)C(C)=C)C)C3(CC2)C)C1)C	5.4	Bad	6.9
5358	O1C2(CCC(CC(O)C(=CCC=C(CCC2O)C)C)C(=C)C1=O)C	5.4	Excellent	6.9
3684	O1CC(C2(C3C(CC2)C(CCCC3=C)(C)C)C)C(CC1=O)COC(=O)C	6.4	Good	6.9
1659	O1CC(C2(C3C(CC2)C(CCCC3=C)(C)C)C)C(CC1=O)COC(=O)C	6.1	Good	6.9
1484	O1C(O)C(=CC1=O)C(OC(=O)C)CC1C2(C(CCC1C=O)C1(C(CC2)C(CCC1)(C) C)C)C	5.0	Poor	6.9
1008	O1C(=O)C(CCC1C(C)C1CCC2C3C(CCC12C)C1(C(=CC(=O)C=C1)CC3)C)C	4.7	Excellent	6.9
13425	O1C23CC(=O)CC(CC2C2C4C(C1(CC4OC2=O)C)C3=O)C(C)=C	4.5	Good	6.9
13331	O1C2C1(C=CC(=O)C(=CC1C(CCC(CC2=O)C)C1(C)C)C)C	5.9	Excellent	6.9
11379	Brc1cc2n3c(-c4[nH]c5c(c4C=C3)cccc5)c(c2cc1)C(=O)C(OCC)=O	4.9	OK	6.9
6310	S1CC(=O)Nc2c1c(c1Nc3c(-c4c1c2ncc4)cccc3)CCN(C)C	4.9	Bad	6.9
5156	OC1CCC2(C(CCC3(C2CC(=O)C3=C(C=CC=C(C=O)C)C)C)C1(C(O)=O)C)C	5.9	Poor	6.9
2465	O1C2C3C(C1CC(=CCCC2(OC(=O)C)C)C)C(=CC(OC(=O)C)C3C(C)C)C	4.4	OK	6.9
1566	O1C(CCCC=CC=CC(=O)CC(O)CC=CC=CC(O)CC=CC=CC1=O)C	4.7	Poor	6.9
1126	O1C2OC(=O)CC(C2C2(CCC3=C(C2C)C(O)CCC3(C)C)C)C1OC(=O)C	6.0	Good	6.9
630	O1C2OC(=O)C3C2C2(C(CC3)C3(C(C(CCC3)(C)C)C(OC(=O)C)C2)C)C1O	5.2	Excellent	6.9
552	O1C2CC(=Cc3oc(CC(CCC4(OC24)C1=O)C(C)=C)c(c3)C)C	4.6	OK	6.9
13606	o1c2c(c3c1c(C=O)c(O)c(O)c3)C1(C(CCC2C)C(CCC1)(C)C)C	5.5	Good	6.9
12808	O1C2(CCC=C(CCC(=CC=C(CCC2OC(=O)C)C)C(C)C)C1=O)C	3.3	OK	6.9
10992	O(C(=O)C)C1CC(O)C2(C(C1)CCC1C3CCC(C=C)C3(CCC12)C)C	3.9	Good	6.9
10982	O1C2CC(CCC3(OC3CCC(=CCCC2(O)C)C)C)C(C)C1=O	5.0	Good	6.9
9819	O1C2CCC1(C)C(O)CC=1C(OC(=O)C=1C)CC(=CCCC2C)C	4.6	Excellent	6.9
9508	OC1CCC2C3C(CCC12C)C1(C(CC(O)CC1)=CC3)C	5.2	Excellent	6.9
8589	O1C2C(CCC(=CCCC(C)C(=O)CCC(=C2)C)C)=C(C)C1=O	4.5	Excellent	6.9
8021	O1C(CC2C3(C(CCC2=C)C2(C(CC3)C(CCC2)(C)C)C)C)C(=CC1=O)CO	6.2	Excellent	6.9

7378	Oc1c(cc(cc1O)C(OC)=O)CC1(C2CCC=C(C)C2(CCC1C)C)C	4.3	Good	6.9
6265	O1C2C=C(CCC(C(C)=C)C(O)C(=O)C(=CC(=O)C3CC23C)C)C1=O	5.9	Excellent	6.9
5302	O=C1CCC2(C3C(C4CCC(C(CC(O)C(OC)=O)C)C4(CC3)C)CCC2=C1)C	4.5	Bad	6.9
4814	O1C2CCC(=CCC3(C(CCC12C)C(C=C3)=C(COC(=O)C)COC(=O)C)C)C	5.2	Bad	6.9
3549	O1c2c(cc(OC)cc2C)CCC1(CC=1CC2(CCCC2(C)C(=O)C=1CC(O)(C)C)C)C	5.0	Bad	6.9
2857	O1C2C3C(C1CC(C)C(=O)CCC2(OC(=O)CCC)C)C(=C)C(O)CC3C(C)C	5.1	Good	6.9
1264	O1C(OC(=O)C)C2C(CC=C(C3(CC(CCC3)(C)C)C)C2=CC)C1=O	5.6	Good	6.9
920	O=C1C=C(c2nc3c(c4c2c1ncc4)cccc3)CCNC(=O)CC(O)(C)C	3.0	Good	6.9
338	Oc1c(cc(cc1O)C(OC)=O)CC1(C2CCCC(=C)C2(CCC1C)C)C	4.8	Poor	6.9
58	O1C2OC(=O)C3C2C2(C(CC3)C3(C(C(O)C2OC(=O)CCC)C(CCC3)(C)C)C)C1O	4.7	Excellent	6.9
13434	O1CC2C3(C(CCC3C(=CC=CC(O)(C)C)C=O)(C)C(O)CC2)C1=O	6.6	Excellent	6.9
12997	O1C2CCC(=CCC(CCC(=CC(OC(=O)C)CC12C)C)C(O)(C)C)C	5.2	Excellent	6.9
12992	O1OC(CCC1(CCC1(C2CCCC(=C)C2(CCC1C)C)C)C)C(C(O)=O)C	5.6	Bad	6.9
11192	Clc1ccc(cc1)C(C(C)C)C(OC(C#N)c1cc(Oc2ccccc2)ccc1)=O	5.4	OK	6.9
10862	O1C=C(C2C(C(CC(O)C=C(CC2)C)=C)C1O)C(OC(=O)C)C(OC(=O)C)C=C(C)C	5.1	OK	6.9
8393	ClC1CC2(C(C(C1)(C)C)C(=O)CC(=C)C2CC(O)C1CC(=O)NC1=O)C	4.9	OK	6.9
7931	O1C(=O)C(=C2CC3C(C4OC3(CC4)C)(CCC=C(CC12O)C)C)C	5.2	Good	6.9
7521	O1C(CCC1C([NH+]=[CH-])(C)C)(C)C1C2C(CCC(C#N)(C)C2O)C(CC1O)(C#N)C	6.3	Excellent	6.9
7297	O1C23C1C=CC(=O)C2(C1C(C2CCC(C(CCC(C(C)C)C)C)C2(CC1O)C)CC3O)C	5.1	Excellent	6.9
6125	O1C2=C(C3=C(C1)C(CCC1(O)C3=CC(=O)C=C1)C)CCCC2(C)C	6.0	Good	6.9
6058	O1C23C1C(O)C1C4CCC(C(CCC=C(C)C)C)C4(CCC1C2(CCC(O)C3)C)C	4.5	Poor	6.9
4936	O1C2C(C=CCC1=O)=C(C1C(C3CCC(C(C=CC(C(C)C)C)C)C3(CC1O)C)C2)C	4.8	Good	6.9
4929	O1CC(C2C3C(C4OC3C1(C)C(O)(CC(=O)C(C4)=C)C(=O)CCCCCCC)C(O)(CC2)C)C	5.1	OK	6.9
4811	O1C2CCC(=CCC3(C(CCC12C)C(=C(C)C)C(=O)C3)C)COC(=O)C	5.5	Poor	6.9
4663	O1C(=O)C(C2C3C(C(O)(CC2)C)C(OC3C1(C=CC=O)C)CC(=O)C)C	5.5	Excellent	6.9
4441	O1C=2C(=CC13C1(C(CCC3C)C(CCC1)(C)C)C)C=C(O)C(=O)C=2C=O	4.5	Excellent	6.9
2965	OC1(CCC2C3C(CCC12C)C1(C(=CC(=O)CC1)CC3)CC)C(=O)COC(=O)C	4.4	Bad	6.9
367	O1C2OC(=O)CC2C(C2(CCC3C(=CCCC3(C)C)C2C)C)C1OC(=O)C	5.4	Good	6.9
194	O1OC(CCC1(CCC1(C2CCCC(=C)C2(CCC1C)C)C)C)C(C(O)=O)C	5.6	Excellent	6.9
9834	OC(=O)c1ccccc1N1Cc2[nH]c3c(c2C=C1)cccc3	4.3	Excellent	6.9
9656	O1C2C(c3c1cc(OC)cc3)COc1c2cc2c(occ2)c1	4.5	Poor	6.9
9277	O=C1C=C(c2nc3c(c4c2c1ncc4)cccc3)C(OC)CNC(=O)C	6.8	Excellent	6.9
9148	O1C(C)(C)C1CC=C(COC(=O)C)C1CCC2(OC2CCC(=C)C1C(OC)=O)C	5.5	Bad	6.9
7782	O1C2C1(CC=C(CCC=C(CCC=C(CO)C)C)C)C(=O)C=C(COC(=O)C)C2O	4.4	Bad	6.9
7779	O1C2C1(CC=C(CCC=C(CCC=C(C)C)C)C)C(=O)C=C(CO)C2OC(=O)C	5.0	Poor	6.9
7237	O1C23C(CCC(C2)C)C2(C(C1(O)C(CC2=O)C(C)C)C(=C3)C(OC)=O)C			6.9
7191	O1C23C(CC=C(C2)C)C2(C(C(=O)C(CC12O)C(C)C)C(=C3)C(OC)=O)C	5.0	Poor	6.9
5958	O1CC2=C(C3(C(CC2)C2(C(CC3O)C3(C(CC2)C(CCC3)(CO)C)C)C)C)C1=O	4.3	Excellent	6.9
4593	O1C2CCC(C)C(O)(CCC3C4(C5(OC(C(O5)CC4)(C)C)CCC3C)C)C2(CCC(=O)C1(C)C)C			6.9
4228	o1cc2c(CCC3C2(CCC2C(C)(C)C(O)C(=O)CC23C)CO)c1	5.6	Good	6.9
4190	O(C(COC(=O)c1ccccc1)c1ccc(cc1)CC)C(=O)c1ccccc1	5.3	OK	6.9
3654	O1C(C2C(C=CCC=CCCCCC1=O)C(O)C(O)C2)CC	5.2	Good	6.9
2858	O1C2CCC(=CCCC(=CC3OC(=O)C(C3CC(=O)C12C)CN(C)C)C)C	4.1	Excellent	6.9
2522	O1C(C2C(C3(C(CC2O)C2(C(CC3O)C3(C(CC2)C(CCC3)(C)C)C)C)C)C1=O)C	4.8	Bad	6.9
2107	O(C)C1=CC(=O)C(CC2(C3CCC=C(C)C3(CCC2C)C)C)=C(O)C1=O	6.1	Good	6.9
1769	O1C2(CC(O)C1(C)C)C1(CCCC1(C)C2C(=O)C(Cc1cc(OC)cc(C)c1O)C(C)=C)C	3.0	Poor	6.9
1122	O1CC(C(COC(=O)C)C(OC(=O)C)C1=O)C1(C2C(CC1)C(CCCC2=C)(C)C)C	5.7	Good	6.9
1096	O1C2C3C(C1CC(=C)C(OO)CC(OC(=O)C)C2(O)C)C(=CCC3C(C)C)C	5.1	Good	6.9

658	O1C(Cc2c(C1=O)c(O)cc1c2c(O)c2c(c1)C(C)(C)C(=O)C(CCC(=O)C)(C)C2=O)C	4.8	OK	6.9
13344	P(Oc1ccccc1)(Oc1ccccc1)(=O)NC1CCCCCCC1	6.0	Good	6.9
12853	O=C1CC2C(CCC3C(C)(C)C(=O)CCC23C)(C)C1=C(C=CC=C(C(=O)C=CC(=O)C)C)C	4.8	Bad	6.9
11584	o1cc2c(CCC3C4(C(CCC23C)C(COC(=O)C)(C)C(OC(=O)C)C(=O)C4)C)c1	3.8	Bad	6.9
11168	O1C(O)C2C(CCC(C3(CC(CCC3)(C)C)C)=C2C(C(OC)=O)C)C1=O	5.7	OK	6.9
10592	O1C(C=CC2C(CC=CCCCC1=O)C(=O)CC2OC(=O)C)CCCCC	5.6	Excellent	6.9
8858	O1C2C3C(C4C(C(C2C(=O)C)C(=O)C3)C(=O)CC(C4)C(C)=C)C1=O	4.3	Poor	6.9
8300	O1C(=CC(CCCC(=CCCc2cc(oc2)Cc2ccoc2)C)C)C(O)=C(C)C1=O	4.0	Bad	6.9
7569	O1C(c2c(c3CC(C)(C)C(O)c3cc2C)C1=O)CO[N+](=O)[O-]	5.1	Poor	6.9
7271	O(C(=O)C)C=1C(=O)C2(O)C3(C(CCC2C)C(CC3C=C(C)C)C)C(=O)C=1C	4.6	Good	6.9
6546	O1C(OC)C2=C(C3(C(CC2)C2(C(CC3)C(CCC2)(C(O)=O)C)C)C)C1=O	5.0	Excellent	6.9
5706	O(C(=O)C)C1CC2=CC(O)C3C(C2(CC1)C)C(O)CC1(C3CCC1C=C)C	4.1	Poor	6.9
5637	O1C2CCC1(C)C(O)CC1(C(CC=C2C)C(=C(C)C)C(=O)C1)C	5.4	Excellent	6.9
5031	ClC1CCC(OC1(C)C)(C)C1C2C(CCC(O)(C)C2[NH+]=[CH-])C(NC=O)(CC1)C	5.4	Excellent	6.9
4937	O1C2C(C=CCC1=O)=C(C1C(C3CCC(C(CCC(C(C)C)C)C)C3(CC1O)C)C2)C	4.6	Bad	6.9
4359	O1C2C(CCC(=CCCC(=CCC=C(C2)C)C(OC)=O)C)C(COC)C1=O	5.1	Poor	6.9
3600	O(C(=O)C)C1C2C(CC=C2C)C(=C)C(O)CC1C(C(OC(=O)C)C(OC(=O)C)C=C(C)C)C	4.6	Poor	6.9
3258	S(O)(=O)(=O)CCNC1=CC(=O)C=C(CC2(C3CCC=C(C)C3(CCC2C)C)C)C1=O	6.3	OK	6.9
2808	O1C2C=C(C(OC)C(O)C(Cc3oc(cc3C(OC)=O)C2C(C)=C)C(C)=C)C1=O	5.8	Good	6.9
2806	O1C2C=C(C(O)C(O)C(Cc3oc(cc3C(OC)=O)C2C(C)=C)C(C)=C)C1=O	5.3	Good	6.9
1329	O(C)c1cc2[nH]c3c(CC4N(C3C=C(C)C)C(=O)C3N(CCC3)C4=O)c2cc1	5.1	OK	6.9
631	ClC1CC2C(=CC1(C=C)C)C(=O)c1c(cccc1NC=O)C2(C)C	3.5	Excellent	6.9
198	OC1(C2CC(=O)C(C=CCC(O)(C=CC2(CC1)C)C)C)C(C)C	4.2	Excellent	6.9
7503	OC1CC2=CCC3C4CCC(=NO)C4(CCC3C2(CC1)C)C	4.8	Good	6.8
999	O1C2CCC(O)(C=CCC(=CC3OC(=O)C(C3CC(=O)C12C)=C)C)C	5.9	Excellent	6.8
12227	S1CC(NC1=O)C1(OC2CCC(C=CC=CCCC(=CC(OC(C1)C2)=O)C)C)O	4.8	Poor	6.8
11736	O1C=C(C2C(C(CCC=C(CC2)C)=C)C1OC(=O)C)C(OC(=O)C)C(OC(=O)C)C=C(C)C	5.4	Good	6.8
9155	O1C2CC(O)(C3C2C(C2C(=CC(=O)CC(C2)C(C)=C)C3=O)C1=O)C	4.5	Good	6.8
6124	Oc1ccc(O)cc1C1C2=C(CCC(C)C1=C)C(CCC2)(C)C	5.6	OK	6.8
3485	O1c2cc(ccc2OC1)Cc1n(C)c(nc1Cc1cc2OCOc2cc1)N	4.1	Good	6.8
12940	O=C1C2N(C(=O)C1=C(O)C)C(C1C2c2c3c(C1)cccc3[nH]c2)(C)C	4.7	Excellent	6.8
8082	ClC1C(C2C(C3CC(OC3(CC2=O)C)C2CC(=O)NC2=O)(CC1Cl)C)(C)C	4.7	Excellent	6.8
6008	O1CC23C(C(CCC2C2(C(C(OC(=O)CCC)C3O)C(CCC2)(C)C)C)C(OC)=O)C1=O	5.0	Poor	6.8
5122	O1C2(OC(=CC3(CCCC23C)C)CC2(Oc3c(cc(O)cc3C)CC2)C)C=CC1(C)C	4.4	Bad	6.8
4835	o1c2c3c(CCCC3(c3c(cc4c(c3)C(=O)C=C(OC)C4=O)C2=O)C)c1	4.6	OK	6.8
3596	O1C2CCC3(OC3CCC(=CC=C(C(C)=C)C(O)CC12C)C)C	4.7	Excellent	6.8
324	Fc1c(N2CCN(CC2)C)c(F)c2N(C=C(C(O)=O)C(=O)c2c1N)c1ccccc1	5.8	Poor	6.8
13010	O=C1C=C(NC)C(=O)C=C1CC1(C2CCC=C(C)C2(CCC1C)C)C	5.8	Excellent	6.8
12749	O1C2(CCC=C(CCC(=CC=C(CCC2=O)C(OC)=O)C(C)C)C1=O)C	4.5	Excellent	6.8
11035	O1CC2C(CCC(=CC(O)CC2=C)C)C(C=CCC(O)(C)C)C1=O	4.4	Excellent	6.8
10678	O(C(=O)C)C1(CC(O)C2C(CCC2C(OC(=O)C)(C)C)(C=CC(CC=C1)C)C)C	4.3	Good	6.8
9822	O1C2CCC1(C)C(O)C1OC(=O)C(C1C(=O)CC(=CCCC2C)C)C	4.7	Good	6.8
9126	O1C(CCC1C(O)(C)C)(C(O)CCC(=C)C1OC2(C(OC(CC2)C2(OC(=O)CC2)C)CC1)C)C	5.0	Bad	6.8
7184	Brc1c(cc2C3C(CCC3C(C)C)(C)C(OC(=O)C)C(=O)C(=O)c2c1O)C	6.5	Good	6.8
6431	O1C2C(CCC(=CCCC(O)(C)C(O)CCC(=C2)C)C)=C(C)C1=O	6.0	Good	6.8
5219	O1CC2C(=CC(OC(=O)C)C3C2(C)C(OC(=O)C)CC2C(CCCC23C)(C)C)C1=O	5.4	Good	6.8
5204	O1C(O)C(=CC1=O)C(O)CC=C(CCC1(C2CCCC(=C)C2(CCC1C)C)C)C	4.6	Good	6.8

4746	O1C2C1(C(=O)C(=CCCC=CC(=CC(=CC)C(OC)=O)C)C)C(=O)NC2(O)C	5.4	Poor	6.8
3862	O1C(C(C)C2OC(CC)=C(C)C(=O)C2C)C(C)C(=O)C(C)=C1CC	6.6	Good	6.8
3299	O1CC(=CC1=O)CCC=C(CCC1C2C3(C)C1(CCC3(C)C(CC2=O)C)C)C	6.3	Poor	6.8
2718	O1C2C(CCC(=CCCC(O)(C)C(O)CCC(=C2)C)C)=C(C)C1=O	4.9	Excellent	6.8
14297	O1C=C2C(C3(C(C(OC(=O)C)C2OC(=O)C)C(CCC3)(C)C)C)C1OC(=O)C	6.0	Good	6.8
12465	O1C2CCC(O)(C)C(CCC3C(C4C(CC=C3C)C(O)(CC4)C)(C)C)C2(CCC(=O)C1(C)C)C	5.6	Poor	6.8
12163	O(C(=O)C)C1CC2C(CC1C(=O)C)(C)C(=O)CC1C2(CCC2C(CCCC12C)(CC)C)C	5.1	Bad	6.8
10983	O1C2CC(CCC3(OC3CCC(=CCCC2(O)C)C)C)C(=C)C1=O	5.4	Good	6.8
8546	O1C23CCCC(=C)C2(CCC(C)C3(CC2=C1C(=O)C=C(OC)C2=O)C)C	5.8	Excellent	6.8
8545	O1C23CCCC(=C)C2(CCC(C)C3(CC2=C1C(=O)C=C(OC)C2=O)C)C	5.5	OK	6.8
7946	ClC1CC2C(C3(C)C1(O)CC=CC3=O)C(O)CC1(C2CCC1C(C=CC(C(C)C)C)C)C	4.0	Poor	6.8
6679	O1C2C(CC3OC2C(CCCC(=CCCC3(OC(=O)C)C)C)C)C(=C)C1=O	6.2	Excellent	6.8
6214	O1C2CC(=O)CC(CCC3=CC(OC3=O)CC1(CC2=O)C)C(C)=C	5.3	Excellent	6.8
6056	OC1C2CC(O)CCC2(C)C(=O)C(=C1)C1CCC(C(C=CCC(C)C)C)C1(CCOC(=O)C)C	5.0	OK	6.8
6013	O1C(O)C2C(CCC3C2(C)C(OC(=O)C)CC2C(CCCC23C)(C)C)C1=O	5.3	OK	6.8
5901	O=C1c2c(NCC1)c1nc3c(c4C=Nc(c14)c2N)cccc3	4.2	Poor	6.8
5782	S(OCC(CC=CC(C)C1CCC2C=3C(CCC12C)C1(C(CC(O)CC1)C(=O)C=3)C)C)(O)(=O)=O	5.6	Bad	6.8
5217	O1CC2(O)C(=CC(OC(=O)C)C3C2(CCC2C(CCCC23C)(C)C)C)C1=O	5.6	Excellent	6.8
5047	O1C2(C(CC1=O)C1(C(C3(C(CC1)C(CCC3)(C)C)C)C(OC(=O)C)C2)COC(=O)C)C	5.0	Poor	6.8
4919	O1C(=O)C(=C2CC3C(C)(C(OC(=O)C)CCC(=CC12O)C)C(OC(=O)C)CC=C3C)C			6.8
3682	O1C2OC(=O)CC2C(C2(C3C(CC2)C(CCCC3=C)(C)C)C)C1O	4.0	Excellent	6.8
3292	OC1CC2=CC(O)C3C4CCC(C(C=CCC(C)C)C)C4(CCC3C2(CC1)C)C	3.8	Poor	6.8
2122	O1C2C3=C4COC3(O)C(O)C1(CCC=C(CCC4(C)C(C2)C)C)C	4.9	Excellent	6.8
1786	O1C2CC(=CC(=O)C=C(C)C(=O)CC(CCC3(OC23)C1=O)C(C)=C)C	5.1	Good	6.8
59	O1C2OC(=O)C3C2C2(C(CC3)C3(C(C(OC(=O)CCC)C2O)C(CCC3)(C)C)C)C1O	3.7	Poor	6.8
13722	O1C2=C(C=CC1(CCC1(CCC=C(C)C1C)C)C)C(=O)C(OC)=CC2=O	4.9	Good	6.8
13011	O=C1C(=CC(=O)C=C1NC)CC1(C2CCC=C(C)C2(CCC1C)C)C	4.1	Poor	6.8
12058	S(C)C12N(C3Nc4c(cccc4)C3(C1)c1c3c([nH]c1)cccc3)C(=O)C(SC)N(C)C2=O	5.0	Excellent	6.8
11193	Clc1ccc(cc1)C(C(C)C)C(OC(C#N)c1cc(Oc2ccccc2)ccc1)=O	5.2	Poor	6.8
11091	O=C1C2C(C3CCC(C(=O)CO)C3(C1)C)CCC1=CC(=O)C=CC12C	4.7	OK	6.8
10542	O1C(=O)C(=CC1=CC(CC=CC(=CCCc1cc(oc1)Cc1ccoc1)C)C)CO	4.5	Poor	6.8
9486	Oc1cc(C)c(CCC(=CCC2=CC(=O)C=CC2=O)C)c(C)c1C=O	5.3	Poor	6.8
7525	OC1CCC2(C(CCC3(C2CC(=O)C3=C(C=CC=O)C)C)C1(CO)C)C	5.1	OK	6.8
7500	O(C(=O)C)C1CCC2(C(CCC3(C2CC(=O)C3=C(C=CC(O)=O)C)C)C1(C(O)=O)C)C	7.2	Poor	6.8
7499	O(C(=O)C)C1CCC2(C(CCC3(C2CC(=O)C3=C(C(O)=O)C)C)C1(C(O)=O)C)C	6.1	Good	6.8
6471	ClC1CCC(OC1(C)C)(C)C1C2C(CCC(O)(C)C2[NH+]=[CH-])C(=CC1)C	5.1	Good	6.8
6018	O1C2C1(C(OC)=O)C(=O)C1(C(C3(C(CC1)C(CCC3)(C)C)C)C2OC(=O)C)C	5.1	Good	6.8
5838	O1C2(CC(O)C1(C)C)C1(CCCC1(C)C2C(=O)C(Cc1cc(OC)cc(C)c1O)C(C)=C)C	5.8	Poor	6.8
5157	OC1CCC2(C(CCC3(C2CC(=O)C3=C(C=CC=O)C)C)C1(C(O)=O)C)C	5.0	Excellent	6.8
4225	O1c2c(CC(O)C1(CCC=C(CCC=C(C)C)C)C)c(O)cc1c2CN(CC(=O)C)C1=O	4.2	Bad	6.8
2281	OC1(C=2N(C=CC3C=2Nc2c3cccc2)c2c1cccc2)CC(=O)C	3.3	Good	6.8
2118	O1C2C1(CCC=C(CCC1(C)C(C)C(O)C=C(C1(O)C)C2=O)C)C	5.1	Excellent	6.8
2068	O1C2CC(=CC3(O)C(C(=O)C(=C3)C)C(CCC3(OC23)C1=O)C(C)=C)C	6.3	Excellent	6.8
1377	O1C2CCC1(C)C(O)C(O)C=1C(OC(=O)C=1C)CC(=CCCC2C)C	4.7	Excellent	6.8

14470	O(C(=O)C)C1CC2C(C3CC(O)C(CC13C)C(=O)C)(CCC1C(CCCC12C)(CC)C)C	5.1	Good	6.8
13762	O1C(C)(C)C1CC=C1C2C(C(CC(O)C=C(CC2)C)=C)C(OC1)O	4.4	Good	6.8
13373	O=C1C=C2CCC3C4CCC(C(C(OC(=O)C)CC=C(C(OC)=O)C)C)C4(CCC3C2(C=C1)C)C	5.3	Bad	6.8
13202	O1C(C=2C(C3(C(CC=2)C2(C(CC3O)C3(C(CC2)C(CCC3)(C)C)C)C)C)C1O)C	4.4	OK	6.8
13111	OC1C2(C3CCC(=C)C(C(OC)=O)C3(CCC2C(CC1=O)(C)C)C)C	4.4	Excellent	6.8
12345	O1C2C(CC(OC(=O)C)C(CCC(=CCCC(=C2)C)C)C(=O)C(=C)C1=O	4.8	Good	6.8
12146	O=C1CC2C3(C(CCC2(C2CC=C(C(=O)C)C(C=O)C12C)C)C(CCC3)(C)C)CO	5.1	Bad	6.8
11108	O1C2C(CC(OC(=O)C)C(CCC(=CCCC(=C2)C)C)C(=O)C(=C)C1=O	5.7	Good	6.8
10679	O(C(=O)C)C1(CC(OC(=O)C)C2C(CCC2C(O)(C)C)(C=CC(CC=C1)C)C)C	5.2	Good	6.8
10664	O1C2(CCC(CC3OC3(CC(O)C=C(CCC2O)C)C)C(=C)C1=O)C	5.3	Excellent	6.8
9204	O=C1CC2(CC(=O)C(=CCC2C2C1(C)C(=O)C=C2C(C)C)C)C	5.8	Excellent	6.8
8827	O1C2=C(CC3(C4CCCC(=C)C4(CCC13C)C)C)C(=O)C(OC)=CC2=O	4.7	OK	6.8
6544	O1C2C1(CC1C3(C(CCC1=C)C(CCC3)(C)C)C)C(=O)C=C(COC(=O)CC(O)=O)C2O	5.1	Bad	6.8
6412	O1C(=O)C2(C(C3C4(C(CCC(C)C4=O)C(C3)C)C2=O)C1(C)C)C	4.7	Excellent	6.8
6255	O1C(O)C(=CC1=O)C=CC1C2(C(CCC1C(O)=O)C1(C(CC2)C(CCC1)(C)C)C)C	6.2	Good	6.8
6055	O1C2CC(=Cc3oc(C(O)C(CCC(=C2)C1=O)C(C)=C)c(c3)C)C	5.5	Excellent	6.8
5890	O=C1C2=CC(=O)CCC2(C2C(C3CCC(C(CCC(OO)C(C)=C)C)C3(CC2)C)C1)C	4.6	Bad	6.8
5290	O1C2C=C(CCC(OC(=O)CCC)C3(C(C(C)C(=O)C=C3)C(=O)C12C(C(OC)=O)C)C)C	6.3	Excellent	6.8
5270	O1C2C(CCC(=CCCC(=CCCC(O)(C2)C)C)C)C(=C)C1=O	6.2	Excellent	6.8
4920	O1C(=O)C(=C2CC3C(C)(C(OC(=O)CC)CCC(=CC12O)C)C(OC(=O)C)CC=C3C)C			6.8
4226	o1cc2c(CCC3C4(C(CCC23C)C(C)(C)C(O)C(=O)C4)C)c1	4.7	OK	6.8
2239	O1C2(c3c(CC(C)C2(CC1C1(OOC(CC1)C(C(O)=O)C)C)C)cc(cc3)C)C	4.9	Bad	6.8
1878	O1C2C(c3oc(CC(C(C)=C)C(O)C=C(C2N(C)C)C1=O)c(c3)C(OC)=O)C(C)=C	4.8	Excellent	6.8
1789	s1c2c(nc1)C=1CCN3C=1C1=C(C=C[NH+](C)[C-]12)c1c3cccc1	4.0	Good	6.8
1211	O1C2(CC3OC(C4C3C(CCC4C(C)C)=C)C1(CCC2OC(=O)C)C)C			6.8
1143	O1C2C=C(CCC(CC(=O)C(=CC(=O)C3CC23C)C)C(C)=C)C1=O	5.2	Excellent	6.8
14433	O1C(C2(C3C2(C)C(=O)C(C)C3C(C(=O)CC)=C)C)=C(C)C(=O)C(C)=C1OC	5.5	Good	6.8
13358	O1CC2C(=CCC3C2(C)C(OC(=O)C)CC2C3(CCC3C(CCCC23C)(COC(=O)C)C)C)C1=O	5.3	Poor	6.8
12969	O1C2C(c3oc(CC(C(C)=C)C(O)C=C(C2OC)C1=O)c(c3)C(OC)=O)C(C)=C	5.4	OK	6.8
12786	O1C(C2C(C3(C(CC2O)C2(C(CC3O)C3(C(CC2)C(CCC3)(C)C)COC(=O)C)C)C)C1=O)C	4.9	Excellent	6.8
12228	S1CC(NC1=O)C1(OC2CCC(C=CCCC(=CC(OC(C1)C2)=O)C)C)O	4.2	Bad	6.8
11435	O(C)c1cc(c2c(c1)C(=O)c1c(C2=O)c(O)ccc1)C	5.1	Poor	6.8
9193	O1C2CC3C4C(CCC3(CC(O)C12C)C)(C)C(=O)C=C4C(C)C	5.2	Excellent	6.8
9191	O1C2CC3C4C(C)(C(=O)CC3(CC(O)C12C)C)C(=O)C=C4C(C)C	6.1	Good	6.8
9147	O1C(OC)C(C2C(C(CCC=C(CC2)C)=C)C1OC)=CC=CC(O)(C)C	5.7	Poor	6.8
8922	OC12CC(O)CCC1(C1C(C3CCC(C(CCC(C(C)C)C)C)C3(CC1)C)=CC2=O)C	5.6	Bad	6.8
8165	O1C(CC=C(CCC2(C3CCCC(=C)C3(CCC2C)C)COC(=O)C)C)C(=CC1=O)CO	5.2	Bad	6.8
2814	O(C(=O)CC(CCC=1C2(C(CCC=1C)C(CCC2)(C)C)C)C)CC(O)COC(=O)C	4.5	Bad	6.8
1537	OC1=C(CC2(C3CCCC(=C)C3(CCC2C)C)C)C(=O)C(NCC(C)C)=CC1=O	4.5	OK	6.8
1258	O(C)c1cc(c2Nc3c(-c4c2c1ncc4)cccc3)C(OC)=O	4.6	Excellent	6.8
963	OC12C(CCCC1(C)C)C(CCC(=CCn1c3c(nc1)N(C=NC3=NC)C)C)(C)C(CC2)C	4.0	Poor	6.8
568	Brc1cc2[nH]c3c(c2cc1)ccnc3C(=O)Cc1ccccc1	5.7	Excellent	6.8
3337	O1C2OC(=O)C=C2C(O)CC=C(CCC=2C3(C(CCC=2C)C(CCC3)(C)C)C)C1O	4.7	Good	6.7
12806	O1C2(CCC=C(CCC(=CC=C(CCC2O)C)C(C)C)C1=O)C	5.3	Good	6.7
12064	O1C(CCC1C([NH+]=[CH-])(C)C)(C)C1C2C(CCC(O)(C)C2[NH+]=[CH-])C(NC=O)(CC1)C	4.3	Poor	6.7

12001	O1C(C)(C)C1C(OC(=O)C)CC(C)C1CCC2C3C(CCC12C)C1(C(=CC(=O)CC1)C C3)C	4.3	Bad	6.7
11475	O1c2c(cc(O)cc2)CC2C3(C(CCC12C)C1(C(CC3)C(CCC1)(C(O)=O)C)C)C	5.2	Bad	6.7
11261	O1CC(=C2CC(O)C(=CCCC(=CCCC(=CC12)C)C(OC)=O)C)C	4.3	Good	6.7
10900	O1CC=2C(C3(C(CC=2)C2(C(CC3OC(=O)C)C3(C(CC2)C(CCC3)(C)C)C)C)C)C 1O	6.0	Poor	6.7
9276	S(C)c1c2c3c(nc4c2cccc4)C(=CC(=O)c3nc1)CCNC(=O)C	5.3	Excellent	6.7
9026	O1C(=CC(CCC=C(CCCC2=CC(OC2=O)(OC)Cc2ccoc2)C)C)C(O)=C(C)C1=O	3.3	Bad	6.7
9025	O1C(=CC(CCCC(=CCCC2=CC(OC2=O)(OC)Cc2ccoc2)C)C)C(O)=C(C)C1=O	3.8	Bad	6.7
6738	O1C2Oc3cc(OC)c4c(Oc5c(C4=O)c(O)ccc5OC)c3C2C=C1	4.7	Excellent	6.7
6625	O1C23C1C(O)C=1C(CCC4(C=1CCC4C(C=CC(C(C)C)C)C)C)C2(CCC(O)C3)C	5.5	Excellent	6.7
5165	OC1CCC2(C(CCC3(C2CC(=O)C3=C(C=CC=C(C=O)C)C)C)C1(C(O)=O)C)C	6.2	Poor	6.7
3496	OC1CC2=CC(=O)C3C4CCC(C(C=CC(=O)C(C)C)C)C4(CCC3C2(CC1)C)C	6.0	Poor	6.7
1748	OC1C2C(C3CCC(C(CCCC(C)C)C)C3(C1)C)CC(=O)C1CC(=O)CCC12C	5.2	Good	6.7
1721	OC1CC2C(C=3C(C4CCC(C(C=CCC(C)C)C)C4(CC=3)C)=CC2O)(CC1)C	5.2	Poor	6.7
1655	O1C(C=CC2C(CC=CCCCC1=O)C(=O)CC2OC(=O)C)CC=CCC	4.5	Excellent	6.7
1138	O1C2OC(=O)CC(C2C2(CCC3=C(C2C)C(O)CCC3(C)C)C)C1OC(=O)C	4.3	Good	6.7
619	O=C1Nc2c(cccc2)C12C1C2([NH+]=[CH-])C(CCC1C(C)=C)(C=C)C	3.5	Excellent	6.7
489	ClC1CC2C(O)(c3c4c(cccc4[nH]c3)C2(C)C)C([NH+]=[CH-])C1(C=C)C	3.4	Excellent	6.7
13133	O1C2OC(=O)CC2C(C(=C)C2CCC3C2(CCCC3(C)C)C)C1OC(=O)C	4.5	Good	6.7
11951	O1C2CCC(C3C(CCC12C)C(=COC3OC(=O)C)C(OC(=O)C)CC=C(C)C)=C	4.7	OK	6.7
11945	O1CC(C2CC(C)C(O)C3C2C2OC3CC(=CCC(O)C12C)C)C	5.2	Excellent	6.7
9154	O1C2CC(O)(C3C2C(C2=C(CC(=O)CC(C2)C(C)=C)C3=O)C1=O)C	6.1	OK	6.7
9109	O1C2C(CCC(=CC(OC(=O)C)CC(=CC(OC(=O)C)CC(=C2)C)C)C)C(=C)C1=O	5.1	Good	6.7
9031	O1C(CC(=CCCC(C=CC(OC)C(O)(CCCc2ccoc2)C)C)C)C(O)=C(C)C1=O	3.7	Bad	6.7
8919	O=C1c2nccc3c2c(nc2c3cccc2)-c2c1[nH]cc2	4.2	Excellent	6.7
8606	OC12CC(O)CCC1(C1C(C3CCC(C(CCC(C(COC(=O)C)C)=C)C)C3(CC1)C)CC2 =O)C	4.7	Bad	6.7
7315	O1C2C(CC3C(C)(C(OC(=O)C)C(=O)C=C3C)C(OC(=O)CC)CCC(=C2)C)=C(C) C1=O	6.1	Excellent	6.7
7314	O1C2C(CC3C(C)(C(OC(=O)C)C(=O)C=C3C)C(OC(=O)C(C)C)CCC(=C2)C)=C(C)C1=O	6.0	OK	6.7
4660	O1C2C34N5C6(OC(CC(C6)C)C5)CCC3(C)C(C3C(C5C(CC3=O)C(=O)C=C(C5) C)(C4)C)(C)C12O			6.7
3539	O1C2CC(CCC(=CCCC(=CCCC2(O)C)C)C)C(=C)C1=O	4.1	Excellent	6.7
2082	O=C1c2c(NC=C1)c1nc3c(c4C=Nc(c14)c2NC)cccc3	4.7	OK	6.7
1595	S1CC(N(CO)C1=O)C1(OC2CCC(C=CC=CCCC(=CC(OC(C1)C2)=O)C)C)O	4.4	Poor	6.7
1151	O1C=C(C2C(C(CCC=C(CC2)C)=C)C1OC(=O)C)C(OC(=O)C)C=C=C	5.4	Poor	6.7
1078	O(C(COC(=O)C1C2(CC=C3C(CCCC3(C)C)C2(CCC1C)C)C)CO)C(=O)C	4.9	Bad	6.7
996	O1C2C(CCC3(OC3CCC(O)(C=CCC(=C2)C)C)C)C(=C)C1=O	5.5	Good	6.7
719	O1C(C2OC1(C1C3(CCCC3(C)C1(O)C2)C)CC(=CCc1cc(OC)cc(C)c1O)C)(C)C	6.1	Good	6.7
587	O1C(CC=C(CCC=C(CCC2(CCCC2C)C(C)=C)C)C1O)C1=CC(OC1O)=O	6.6	Bad	6.7
12352	O1C2C(=CC1=O)C(OC(=O)C)CC1C2(C)C(O)CC2C1(CCC1C(CCCC12C)(C)C) C	4.2	OK	6.7
12214	O1c2c(C3C1c1c(OC3)cc3occc3c1)ccc(OC)c2OC	3.8	Excellent	6.7
11563	O1CC2C(CCC3(OC3C(O)CC2=C)C)C(=CC=CC(O)(C)C)C1=O	4.6	Poor	6.7
11411	O(C)C1=CC(=O)C(O)=C(C=C2C3(C(CCC2C)C(CCC3)(C)C)C)C1=O	4.2	Poor	6.7
9156	O1C2CC(=O)CC(CC(OC)C3=CC(OC3=O)CC1(CC2=O)C)C(C)=C	6.1	Excellent	6.7
4836	o1c2c3c(CCCC3(c3c(cc4c(c3)C(=O)C(OC)=CC4=O)C2=O)C)c1	5.7	Good	6.7
4718	O1CC(=CC1=O)C(O)CC1CC2C(CCCC2(C2CCC(CC12C)=C)C)(C)C	5.5	Excellent	6.7
4230	o1cc2c(CCC3C2(CCC2C(C)(C)C(=O)C(O)CC23C)CO)c1	4.6	OK	6.7
3401	O1CC(C2CC(C)C(OC(=O)C)C3C2C2OC3CC(CCC(=O)C12C)=C)C	4.8	Good	6.7

2989	O1C(CC=C(CCC=C(CCC=2C(CCCC=2C)(C)C)C)C1=O)C1CC(OC1)=O	5.3	Poor	6.7
2347	s1c2c(nc1)c1nccc3-c4c(Nc(c13)c2CCNC(=O)C)cccc4	6.3	Excellent	6.7
665	S=C=NC(C)(C)C1OC(CC1)(C)C1C2C(CCC(O)(C)C2[NH+]=[CH-])C([NH+]=[CH-])(CC1)C	4.2	Good	6.7
335	OC1CC2C(C3CC=C(C=O)C(C=O)C13C)(CCC1C(CCCC12C)(C)C)C	4.0	Bad	6.7
7	O1C2CC(=O)CC(CCC3=CC(OC3=O)CC1(CC2=O)C)C(C)=C	5.8	Excellent	6.7
14491	O1C2CC(=O)CC(CC=C(CC(=O)CC1(CC2=O)C)C(OC)=O)C(C)=C	4.9	Poor	6.7
14444	O1C(C2(C=C(C=C(C)C2C(=CC(C(=O)CC)C)C)C)C)=C(C)C(=O)C(C)=C1OC	5.3	Good	6.7
14011	O1C2C(CC3OC3(CCC=C(CCCC(C)C2O)C)C)C(=C)C1=O	5.1	Excellent	6.7
13403	O1C2C3C(C(OC(=O)C)(CCC3C(C)C)C)C(=O)CC(O)(C1CCC2(O)C)C	4.7	Good	6.7
11846	ClC1CCC2(C(C1(C)C)C(=O)C=C(C)C2CC(O)C1CC(=O)NC1=O)C	4.7	Excellent	6.7
10922	O1CC2=C(C3C(C3)C(CC(OC(=O)C)C2C(CCC=C(C)C)C)C)C1=O	5.3	Excellent	6.7
9143	O=C1c2c(NC1=C1c3c(NC1=O)cccc3)cccc2	6.1	Poor	6.7
8394	ClC1CC2(C(C(C1)(C)C)C(=O)C=C(C)C2CC(O)C1CC(=O)NC1=O)C	4.8	Bad	6.7
7544	OC1=C(CC2(C3CCCC(=C)C3(CCC2C)C)C)C(=O)C(N)=CC1=O	5.2	Good	6.7
7524	OC1CCC2(C(CCC3(C2CC(=O)C3=C(C=CC=O)C)C)C1(CO)C)C	5.3	OK	6.7
6526	O=C1NC(=O)C2=C1c1c3c([nH]c1-n1c2cnc1)cccc3	6.1	Excellent	6.7
6017	O(C(=O)C)C1C2C(CCC3C(CCCC23C)(C)C)(C)C(=O)C(=C1)C(OC)=O	4.8	OK	6.7
5569	Brc1cc2[nH]cc(c2cc1)C1=NCCc2c1[nH]c1c2cccc1	4.4	Excellent	6.7
5166	OC1CCC2(C(CCC3(C2CC(=O)C3=C(C=CC=O)C)C)C1(C(O)=O)C)C	5.8	Excellent	6.7
4926	O1C2C(CC3C(C)(C(OC(=O)CC)CCC(=C2)C)C(OC(=O)C)CC=C3C)=C(C)C1=O			6.7
4693	O1C2C(CC3OC3(CCC(O)C(CCCC(C)C2OC(=O)C)=C)C)C(=C)C1=O	4.5	Bad	6.7
3946	O1C(CC=C(CCC=C(CCC2=C(C)C(=O)CCC2(C)C)C)C1O)C1=CC(OC1O)=O	3.9	Bad	6.7
3326	O1C(=O)C(CC1C=C(C)C)=CCCC(=CCCC(=CCC(OC(=O)C)C1=CC(OC1)=O)C)C	4.8	Bad	6.7
1912	O(C)C1=CC(=O)C(O)=C(CC23C4(C(CCC2C)C(CCC4)(C)C)C3)C1=O	5.2	OK	6.7
487	S=C=NC1C2C(CC(O)C1(C=C)C)C(c1c3c2c[nH]c3ccc1)(C)C	4.2	Good	6.7
449	O1C2OC(=O)C3C2C2(C(CC3)C3(C(CC2)C(CCC3)(C)C)C)C1O	4.8	Poor	6.7
14451	O(C)c1cc2c(c(CCC)c1C(OC)=O)C(=O)C1=C(C(C)(C)C(=O)C(C)=C1O)C2=O	5.2	Good	6.7
13725	O1C(=O)C(CC1C=C(C)C)=CCC(OC(=O)C)C(=CCCC(=CCCC1=CC(OC1)=O)C)C	4.9	Good	6.7
13183	O1C2CCC(C3C(CC(O)C12C)C(=CC(C=C(C)C)C3O)C=O)=C	4.8	Good	6.7
12762	OC1CC2=CCC3C4CCC(C(C=CCC(C)C)C)C4(CCC3C2(CC1)CO)C	4.4	OK	6.7
10676	OC1(CC(O)C2C(CCC2C(OC(=O)C)(C)C)(C=CC(CC=C1)C)C)C	4.7	Good	6.7
9323	O1C(=CC(CCCC(=CC=CC2(CCCc3c2occ3)C)C)C)C(O)=C(C)C1=O	4.6	Poor	6.7
9190	O1C2CC3C4C(CCC3(CC(O)C12C)C)(C)C(O)C=C4C(C)C	4.4	Excellent	6.7
8943	O=C1NC(=CC(C)C2CCC3C=4C(CCC23C)C2(C(CC(N)CC2)CC=4)C)C(=C1)C(C)C	4.3	Bad	6.7
8828	O1C2=C(CC3(C4CCCC(=C)C4(CCC13C)C)C)C(=O)C(OC)=CC2=O	5.0	Good	6.7
8549	O=C1CC2C(CCC3C(C)(C)C(=O)CCC23C)(C)C1=C(C=CC=C(C(=O)C=CC(=O)C)C)C	6.1	Bad	6.7
8197	O1C2C1(CC1C3(C(CCC1=C)C(CCC3)(C)C)C)C(=O)C=C(CO)C2=O	5.0	OK	6.7
8081	ClC1C(C2C(CC1Cl)(C)C(CC(O)C1CC(=O)NC1=O)C(CC2=O)=C)(C)C	5.7	Excellent	6.7
7488	O(C(=O)C)C1CCC2(C(CCC3(C2CC(=O)C3=C(C=CC(OC)=O)C)C)C1(C(OC)=O)C)C	4.8	Poor	6.7
7421	O(C(=O)C)C1C(C)=C(CCC(=CCO)C)C2(C(C1OC(=O)C)C(CC(OC(=O)C)C2)(C)C)C	5.3	OK	6.7
6955	O(C(=O)C)C1CC(C(=CCC=C(C)C)C)C(C2C(C)C(OC(=O)C)CC2=O)C1C=O	5.6	Good	6.7
2798	s1c2c3c(C(=O)c4c(-c3c1C(OC)=O)cccc4O)c(O)cc2	4.6	Excellent	6.7
2385	O1C2CC=C(C3C(C(COC3=O)CCC=C(C)C)C(O)CC12C)C=O	6.2	OK	6.7
1881	ClC(C)(C)C1OC(CC1)(C)C1C2C(CCC([NH+]=[CH-])(C)C2O)C([NH+]=[CH-])(CC1)C	3.7	Good	6.7

1712	O=C1c2c(NCC1)c1nc3c(c4C=Nc(c14)c2N(C)C)cccc3	5.9	OK	6.7
1536	OC1=C(CC2(C3CCCC(=C)C3(CCC2C)C)C)C(=O)C(NCCC(C)C)=CC1=O	5.5	Good	6.7
1320	O(C)c1cc2[nH]c3c(CC4N(C3C=C(C)C)C(=O)C3N(CCC3)C4=O)c2cc1	3.4	Good	6.7
210	OC1(C2CC(=O)C(C=CCC(O)(C=CC2(CC1)C)C)C)C(C)C	4.7	Excellent	6.7
16	O1C(C(C(=O)C(C)C=2OC(CC)=C(C)C(=O)C=2C)C)=C(C)C(=O)C(C)=C1CCCC C	5.9	Poor	6.7
14439	O1C(C2(C=C(C)C(=O)C(C)=C2C(=CC(C(=O)CC)C)C)C)=C(C)C(=O)C(C)=C1O C	5.8	Excellent	6.7
13351	O1C(C2C(C(CCC=C(CC2)C)=C)C1=O)(C=CC=C(C)C)CO	5.6	Excellent	6.7
13297	O1C2C1(CC1C3(C(CCC1=C)C(C)(C)C(=O)CC3)C)C(=O)C=C(CO)C2O	4.8	OK	6.7
12857	O1C2CC(=CC(=O)C=C(C)C(=O)CC(CCC(=C2)C1=O)C(C)=C)C	6.1	Excellent	6.7
11619	O(C)C1=CC(=O)C(O)=C(CC2(C3CCCC(=C)C3(CCC2C)C)C)C1=O	4.6	Excellent	6.7
10411	OC1CC2=CCC3C4CCC(C(=NO)C)C4(CCC3C2(CC1)C)C	4.6	OK	6.7
9226	O1C(OC)(C=2OC3(CCC4(C(CCCC4=C)C3(CC=2C1=O)C)C)C)CC(OC)=O	6.2	Good	6.7
9150	O=C1CCC2C(CCC3(C2CCC3C=C)C)C1(CCC(OC)=O)COC(=O)C	5.8	OK	6.7
8548	OC12CC(O)CCC1(C1C(C3CCC(C(CCCC(C)C)C)C3(CC1)C)=CC2=O)C	4.7	Bad	6.7
7522	OC1CCC2(C(CCC3(C2CC(=O)C3=C(C=CC=C(C=O)C)C)C)C1(CO)C)C	5.8	Poor	6.7
7465	O1C2C3OC(=O)C(C3CC(O)C1(CCC=C(CCCC2C)C)C)=C	5.5	Poor	6.7
7195	O1C23C(OC(=O)C12C)C=C(CCC(O)C1(C(C(C)C(=O)C=C1)C3O)C)C	5.3	Excellent	6.7
6550	O1CC2=C(C3(C(CC2)C2(C(CC3)C(CCC2)(C(O)=O)C)C)C)C1=O	5.6	Excellent	6.7
6048	O1C23CC(O)CCC2(C)C(=O)C(=CC13)C1CCC(C(CCC(C(C)C)=C)C)C1(CC=O) C	4.6	Bad	6.7
4932	Brc1cc2NC(=O)C(c2cc1)=C1Nc2c(ccc(Br)c2)C1=O	6.1	Bad	6.7
4812	O1C2C1(CCC1C(=C(C)C)C(=O)CC1(CC=C(CC2OC(=O)C)C)C)C	4.5	Poor	6.7
4659	O1c2c(cc(O)cc2)C=CC1(CCC1C(CC(=O)C=C1C)(C)C)C	4.9	Excellent	6.7
4382	O1C2(CC(OOC2CC1=O)(CC(CCCCCCc1ccccc1)C)C)C	5.3	Good	6.7
2551	O1C(=O)C(=C2CC3(C(CC12OC)C(OC(=O)C(=CC)C)CCC3C)C)C	5.6	Excellent	6.7
2283	O=C1N(C=Cc2c1[nH]c1c2cccc1)c1ccccc1C(OC)=O	4.6	Good	6.7
1631	O(C)C1=CC(O)(C(OC)=O)C(CC2(C3CCCC(=C)C3(CCC2C)C)C)C1=O	5.2	Good	6.7
1454	S(OC(CC1C2(C(CC=C1C)C(CCC2)(C)C)C)C(CCCc1ccoc1)C)(O)(=O)=O	5.4	Poor	6.7
1284	O1C2CC(=CC(=O)C=C(C)C(=O)CC(CCC(=C2)C1=O)C(C)=C)C	6.3	Excellent	6.7
12850	O1CC=2C(C3(C(CC=2)C2(C(CC3=O)C3(C(CC2)C(CCC3)(C)C)C)C)C)C1OC(= O)C	5.0	OK	6.7
11141	O1C(C(C)C2(O)CCC3C4C(CCC23C)C2(C(CC(O)CC2)=CC4)C)C1CC(C)C	4.0	Poor	6.7
10529	OC1CC2=CCC3C4CCC(C(=NO)C)C4(CCC3C2(CC1)C)C	4.6	OK	6.7
8735	O=C1N2C(CCC2)C(=O)NC1Cc1c2c([nH]c1)cccc2	4.8	Poor	6.7
6856	O1C(C2C3(CC=C4C(=CCC5C(C)(C)C(O)CCC45C)C3(CC2)C)C1=O)(CCCC(O C)(C)C)C	5.3	Poor	6.7
6128	OC12C=C3CC(O)CC3C(C=C(C=CC1CCCCCCC)C)C2=O	6.0	Excellent	6.7
5568	O(C(=O)C)C1CC2C(C3C=CC(=CC13C)C=O)(CCC1C(CCCC12C)(C)C)C	5.4	Excellent	6.7
5359	O=C1C(=C2NC(CC2C)CC(CC=CC#CC#CCCCCC)C)C(=O)N(C)C1C	4.3	Bad	6.7
4709	OC1CC2=CC(=O)C3C4CCC(C(=O)C)C4(CCC3C2(CC1)C)C	6.2	Good	6.7
3320	O1CC(=CC1=O)C=CC=C(CCC=1C2(C(CCC=1C)C(CCC2)(C)C)C)CO	5.0	Poor	6.7
2581	O=C1CC(=CC(=O)C=C(CCC=C(C)C(=O)CC1C(C)C)C)C(OC)=O	6.5	Excellent	6.7
1598	O1C(C(C)C)C(C)C(=O)C(C)=C1C(C)C=1OC(CC)=C(C)C(=O)C=1C	5.2	Excellent	6.7
628	O1C(OC(=O)C)C2C3(C(CCC2C(OC)=O)C2(C(CC3)C(CCC2)(C)C)C)C1OC(=O)C	5.0	Poor	6.7
14478	O1C2C(CCC(=CCCC(C)C(=O)CCC(=C2)C)C)=C(C)C1=O	4.9	Excellent	6.6
14364	S1CC(NC1=O)C(=O)CC1OC(=O)C=C(CCC=CC(CCC(OC)C1)C)C	4.9	Bad	6.6
13767	O1C2(CCC(CC3OC3(CCC=C(CCC2O)C)C)C(=C)C1=O)C	4.5	Excellent	6.6
11470	[NH+](C12C3C(C(CC(C3CCC1C)C)CC([NH+]=[CH-])(C)C)C(CC2)=C)=[CH-]	4.3	Excellent	6.6
9820	O1C2CCC1(C)C(O)CC=1C(OC(=O)C=1C)CC(=CCCC2C)C	5.8	Good	6.6

8020	O1C(O)C(=CC1=O)C=CC1C2(C(CCC1=C)C1(C(CC2)C(CCC1)(C)C)C)C	3.8	Bad	6.6
7376	Oc1ccc(cc1CC1(C2CCC=C(C)C2(CCC1C)C)C)C(OC)=O	3.7	Excellent	6.6
7052	O1C2C=C(CCC=C(CCC=C(CCC2C(O)(C)C)C)C)C1=O	6.4	OK	6.6
6387	n1cnc2N(CN(c2c1N)CC=C(CCC1C2(CCCC(C)C2(CCC1=C)C)C)C)C	4.4	Bad	6.6
6014	O1CC2C3(C(CCC2(O)C1=O)C1(C(CC3OC(=O)C)C(CCC1)(C)C)C)COC(=O)C	5.5	Poor	6.6
5843	O1C2(C3(OC1(CC3)C(CCCC(C)C)C)C1C(CC2OC(=O)C)C2(C(=CC(=O)C=C2) CC1)C)C	4.6	Good	6.6
4615	O1C2C1(CCC=C(CCC1(C3=C(CCC1C)C2(OC3)O)C)C)C	5.6	Excellent	6.6
4217	O1C2C(CCC3(OC3CCC(O)(C=CCC(=C2)C)C)C)C(=C)C1=O	4.9	Excellent	6.6
3782	O1C2C3C(C1CC(O)(C)C(OC)CCC2(OC(=O)C)C)C(O)(CCC3C(C)C)C	4.1	Excellent	6.6
3547	O1c2c(cc(OC)cc2C)CCC1(CC=1CC2(CCCC2(C)C(=O)C=1CC(O)(C)C)C)C	4.8	Bad	6.6
3497	OC1CC2=CC(=O)C3C4CCC(C(CCC(=O)C(C)C)C)C4(CCC3C2(CC1)C)C	5.5	Excellent	6.6
2532	O1C2C3C(CCC3(CC=C(CC(=O)CC(C2)C)C)C)=C(C)C1=O	4.7	Excellent	6.6
2240	O1OC(CCC1(CCC1C(C2C(CC1=C)CC(CC2)=C)(C)C)C)C(C(O)=O)C	5.9	Bad	6.6
1400	O1CC(C2CCC3(C(CCc4c3coc4)C2(CC1=O)C)C)C(OC)=O	4.4	Good	6.6
651	Brc1cc2nc3c4c(nccc4c2cc1)-c1ncccc1C3=O	5.9	Poor	6.6
445	O(C(=O)C)C1CC(CCC(C)C(OC(=O)C)CCC(CC(=O)CC1C)C)C(C)=C	4.2	OK	6.6
336	O=C1CC2C(C3CC=C(C=O)C(C=O)C13C)(CCC1C(CCCC12C)(C)C)C	3.8	Bad	6.6
13363	O1C2CCC(=CC=C(CCC(=CCCC12C)C(OC)=O)C(C)C)C(OC)=O	4.2	Good	6.6
12685	O1C2C(CCC(=CCCC(=CCCC(=C2)C)C(O)=O)C)C(=C)C1=O	4.4	Excellent	6.6
11943	O1CC(C2CC(C)C(OC(=O)CCC)C3C2C2OC3CC(=CCC(O)C12C)C)C	5.6	OK	6.6
11925	O=C1C=C2CCC3C4CCC(C=C)C4(CCC3C2(C=C1)C)COC(=O)C	4.4	Excellent	6.6
11566	O1CC2C(CCC(=CC(O)CC2=C)C)C(=CC=CC(O)(C)C)C1=O	6.1	Excellent	6.6
11412	O1C2=C(CC3C4(C(CCC13C)C(CCC4)(C)C)C)C(=O)C(OC)=CC2=O	4.6	OK	6.6
10807	OC1CC2=CCC3C4CCC(C(CCC(OC)=O)C)C4(CCC3C2(CC1)C)C	3.8	Bad	6.6
10407	O(C(=O)c1ccccc1)c1c2nc3c(nc2ccc1)cccc3	4.8	Excellent	6.6
8330	O(O)C1CCC(=C)C(=O)CC2(CC(=O)C(=C2CCC1=C)C(C)C)C	4.7	Excellent	6.6
7783	O1C2C1(CC=C(CCC=C(CCC=C(CO)C)C)C)C(=O)C=C(CO)C2OC(=O)C	5.2	Bad	6.6
7133	O1C23C4C(CCC4C)C(=CC2CC(OC3=C(C)C1=O)(CCCc1ccoc1)C)C	5.3	Excellent	6.6
5996	Brc1cc2NC(=C3c4c(NC3=O)cccc4)C(=O)c2cc1	5.8	OK	6.6
5676	O1C2=C(C3=C(C1)C(CCC1(O)C3=CC(=O)C=C1)C)CCCC2(C)C	5.8	Excellent	6.6
4940	O(C)c1cc2[nH]c(CC=C(C)C)c(c2cc1)CC1NC(=O)C2N(CCC2)C1=O	4.3	Good	6.6
4307	O1C2C3C(C1C(CC(=O)CCC2(OC(=O)C)C)C)C(CCC3C(C)C)=C	5.6	Excellent	6.6
4131	Oc1cc(CC(OC)=O)c(O)cc1CC1C2(C(CCC1=C)=C(C)C(CC2)C)C	4.7	Poor	6.6
3736	OC1CC2(C(C3C1(C)C(O)C=C3C(C)C)CCC(=CC2)C)C	5.1	Good	6.6
3445	O1C2CCC1(C)C(OC(=O)C)CCC(=CC=C(CCC2(O)C)C(C)C)C	3.9	Poor	6.6
3132	O1C(C)(C)C(O)CC(=O)C1(CCCC(=CC(=O)CC(=CCc1cc(O)cc(C)c1OC)C)C)C	4.1	Poor	6.6
2589	Oc1c(cc(cc1O)C(OC)=O)CC1C2(C(CC=C1C)C(CCC2)(C)C)C	4.5	Good	6.6
2236	O1CC1C1(CC2C(=CC1)C1(C(CC2O)C2(C(C2)C(OC(=O)C)C1)CO)C)C	5.2	Good	6.6
588	O1C(O)C(=CC1=O)C(O)CC=C(CCC=C(CCC1(CCCC1C)C(C)=C)C)C=O	4.9	Poor	6.6
462	O1Cc2c(c(C(C(OC)=O)C)c(cc2)C2(CC(CCC2)(C)C)C)C1=O	4.8	Good	6.6
14434	O1C(C2(C=C(C=C(C)C2C(=CC(C(=O)CC)C)C)C)C)=C(C)C(=O)C(C)=C1OC	5.4	Excellent	6.6
14404	O1C(O)C(=CC1=O)C=CC=C(CCC=C(CCC=1C(CCCC=1C)(C)C)C)C=O	5.9	Poor	6.6
11791	O1C2CCC1(C)C(O)C(OC(=O)C)c1c(occ1C)CC(=CCCC2C)C	4.4	Excellent	6.6
11653	O(C(=O)C)C1C2C(C3C(C1)(C)C(CC3OC(=O)C)C=C)CCC1=CC(=O)CCC12C	4.3	Good	6.6
11288	O1C(C)(C)C1CC(OC(=O)C)C=1C2C(C(CC(O)C=C(CC2)C)=C)C(OC=1)O	5.5	OK	6.6
11032	Clc1ccc(cc1)C(C(C)C)C(OC(C#N)c1cc(Oc2ccccc2)ccc1)=O	4.7	Poor	6.6
9520	O1C2C(CC3OC3(CCC=C(CCCC(C)C2O)C)C)C(=C)C1=O	5.5	Good	6.6
9048	O1C2(O)C3=C(CCC(CCC(CC(O)C=C(C2)C)=C)C3(C)C)C1=O	4.9	Excellent	6.6
8620	Oc1ccc(O)cc1CC1C2(C(CC=C1C)C(CCC2)(C)C)C	5.2	Excellent	6.6
5940	O1C2CC(CCC(=CCCC(O)(C=CCC12C)C)C)C(C(OC)=O)=C	5.1	OK	6.6
5858	O1CC2C(CCC(=CCCC2=C)C)C(=CCC(OC(=O)C)C(C)=C)C1=O	5.1	Good	6.6

5777	O1C(CC)C(O)CC=CCC=CCC=CCCCCC1=O	4.8	Excellent	6.6
5623	ClC1(CCC2C(CCC3C(CCCC23C)(C)C)(C)C1C(OCC(O)COC(=O)C)=O)C	5.5	Poor	6.6
5301	O=C1CCC2(C3C(C4CCC(C(CC(O)C(OC)=O)C)C4(CC3)C)C=CC2=C1)C	5.4	Poor	6.6
4923	O1C2(C3OC(C4C3C(CC(O)C4(O)C)C(C)C1=O)CC(=CCC2OC(=O)CCCCCCC)C)C	4.1	OK	6.6
4575	O1C2C3C(C1C(OC(=O)CCC)(CCC(O)C(=C)C2O)C)C(CCC3=C)C(C)C	4.2	Excellent	6.6
3239	Oc1ccc(cc1CC1C2(C(CC=C1C)C(CCC2)(C)C)C)C(OC)=O	5.0	Poor	6.6
3189	OC1=C(CC=C(CCC2(CCCC(C)C2=C)C)C)C(=O)C(NCCC(C)C)=CC1=O	4.2	Bad	6.6
1759	O1C2(CC(O)C1(C)C)C1(CCCC1(C)C2C(=O)C(Cc1cc(OC)cc(C)c1O)C(C)=C)C	3.7	Bad	6.6
1349	O1C(=CC(CCCC(=CCCC(=CCC(O)c2ccoc2)C)C)C)C(O)=C(C)C1=O	5.6	Bad	6.6
1011	O1C(=O)C(CCC1C(C)C1CCC2C3C(CCC12COC(=O)C)C1(C(=CC(=O)CC1)CC3)C)C	5.0	Bad	6.6
668	ClC1CCC(OC1(C)C)(C)C1C2C(CCC(O)(C)C2[NH+]=[CH-])C(CC1)=C	5.6	Excellent	6.6
14131	O1C(=CC(CC=CC(=CCCc2cocc2Cc2ccoc2)C)C)C(O)=C(C)C1=O	5.8	Excellent	6.6
13546	OC12CC(=O)CCC1(C1CCC3(C(CCC3C(C=CC(C(C)C)C)C)C1(O)C=C2)C)C	5.2	Good	6.6
13471	O1CC2C(CCC(=CC(=O)CC2=C)C)C(=CC=CC(O)(C)C)C1=O	6.3	Excellent	6.6
12854	O=C1CC2C(CCC3C(C)(C)C(=O)CCC23C)(C)C1=C(C=CC=C(C(=O)C=CC(=O)C)C)C	4.3	Bad	6.6
11950	O1C(C)=C(C2C(C(CCC=CCC2)=C)C1OC(=O)C)C(O)CC=C(C)C	5.7	Poor	6.6
11602	O1C2(CCC=C(CCC(=CC=C(CCC2=O)C)C(C)C)C1=O)C	4.5	Excellent	6.6
11167	O1C(OC)C2C(CCC(C3(CC(CCC3)(C)C)C)=C2C(C(O)=O)C)C1=O	6.4	Poor	6.6
10460	O(C(=O)C)c1ccc(OC(=O)C)cc1CC1(C2CCC=C(C)C2(CCC1C)C)C	5.9	OK	6.6
9766	Brc1cc(N2Cc3[nH]c4c(c3C=C2)cccc4)c(cc1)C(O)=O	4.7	Poor	6.6
9324	O1C(=CC(CCCC(=CC=CC2(CCCc3c2occ3)C)C)C)C(O)=C(C)C1=O	5.3	Good	6.6
7053	O1C2C=C(CCC=C(CCC=C(CCC2C(OC(=O)C)(C)C)C)C)C1=O	5.7	Excellent	6.6
6747	O1c2c(c(cc(OC)c2)C)C(=O)c2c1cc(O)cc2O	5.0	Poor	6.6
6617	OC1C(=O)C(=CC=C(CCC=C(CO)C(=O)CCC1C)C)C(C)C	4.9	Excellent	6.6
6602	O1C23C(CCC(=C2)C)C2(C(C(=O)C(CC12O)C(C)C)C(=C3)C(OC)=O)C	4.4	Excellent	6.6
6472	O1C(CCC1C([NH+]=[CH-])(C)C)(C)C1C2C(CCC(O)(C)C2[NH+]=[CH-])C(CC1)=C	3.5	Excellent	6.6
5950	O1OC(CCC1(CCC1C2(C(CCC1(O)C)C(CCC2)(C)C)C)C)C(C(OC)=O)C	4.2	Good	6.6
5401	Oc1ccc(cc1)-c1c2c([nH]c1)C(=O)c1n(cc3CCN=C2c13)C	4.1	Poor	6.6
5064	Oc1ccc(cc1)CCNC1=CC=2N(CCC=3C=2C(=NC=3)C1=O)C	3.5	OK	6.6
5007	O1C2C=C(C(O)CC(Cc3oc(cc3C(OC)=O)C2C(C)=C)C(C)=C)C1=O	4.5	Poor	6.6
4720	o1c2c(nc1)cc(O)c(O)c2CC1(C2CCC=C(C)C2(CCC1C)C)C	4.5	Good	6.6
3346	O1C(O)C(=CC1=O)C=CC=C(CCC=1C2(C(CCC=1C)C(CCC2)(C)C)C)CO	4.2	Bad	6.6
2530	O1C2C3C(C1CC(=C)C(OC(=O)C)CCC2(OC(=O)CCC)C)C(CCC3C(C)C)=C	4.8	Poor	6.6
1717	O(C)C1=CC(=O)C(O)=C(CC=C(CCC2(CCC=C(C)C2C)C)C)C1=O	4.7	Good	6.6
1401	O=C1CC(CCC(C)C(=O)CCC(CC(=O)CC1C)C)C(C)=C	4.6	Poor	6.6
1037	s1c2c(nc1)c1N(C=Cc3c1c(nc1c3cccc1)c2CCN(C)C)C	4.3	Excellent	6.6
895	O1C2C(CC3C(C)(C(OC(=O)C)CC4OC34C)C(OC(=O)CCC)CCC(=C2)C)=C(C)C1=O	4.2	Poor	6.6
451	O1CC23C(C(CCC2C2(C(C(O)C3O)C(CCC2)(C)C)C)C(OC)=O)C1=O	4.9	Poor	6.6
14237	O(C)C1=CC(=O)C(O)=C(CC2(C3CCCC(=C)C3(CCC2C)C)C)C1=O	4.7	Good	6.6
14125	O1C23C1CC1C(C2(C)C(=O)C=CC3)C(O)CC2(C1CCC2C(C)C1CC1C(C(C)C)C)C	4.0	Poor	6.6
14029	O(C(=O)C)C1CC2(C(CC=C(CCC=C1C=O)C)C(CC2)C(C)=C)C	6.5	Excellent	6.6
13996	OC1CCC(O)(C2C3C(CCC12C)C(CC=C3C)C(C)C)C	5.0	Excellent	6.6
13516	O1C2CC(OC(=O)C)C3(C(CC(=C(C(OC)=O)C)C(=O)C=C(CCC3)C)C12C)C	4.4	Excellent	6.6
13359	O1CC2C(=CCC3C2(C)C(OC(=O)C)CC2C3(CCC3C(CCCC23C)(COC(=O)C)C)C)C1=O	5.3	Poor	6.6
11384	Brc1cc(N2C=Cc3c([nH]c4c3cccc4)C2=O)c(cc1)C(O)=O	4.2	OK	6.6

11173	O1C(C2=C(C(CCC2C2(CC(CCC2)(C)C)C)C(OC)=O)C1=O)C	5.4	Good	6.6
10899	O1C(O)C2C(=CCC3C2(C)C(OC(=O)C)CC2C3(CCC3C(CCCC23C)(C)C)C)C1=O	4.5	Poor	6.6
6564	O(C(=O)C1C2(C(CCC1=C)C1(C(CC2)C(CCC1)(C)C)C)C)CC(O)COC(=O)C	4.4	Bad	6.6
5447	O1C(=O)C(C2CC(O)C(=CCC(O)C3(OC(CC3)C1(CC2)C)C)C)=C	5.3	Excellent	6.6
5335	O1CC2C(CCC(=CCCC2=C)C)C(=CC=CC(O)(C)C)C1=O	6.2	OK	6.6
5164	OC1CCC2(C(CCC3(C2CC(=O)C3=C(C=CC=C(C=CC=O)C)C)C)C1(C(O)=O)C)C	5.7	Bad	6.6
2344	s1c2c(nc1)-c1nccc3c1c(nc1c3cccc1)C2=O	6.1	OK	6.6
1455	O1c2c(cc(O)cc2C)CC2C3(C(CCC12C)C1(C(CC3)C(C)(C)C(=O)CC1)C)C	3.9	Bad	6.6
1283	O1C2C=C(CCC(CC(=O)C(=CC(=O)C2=C(C)C)C)C(C)=C)C1=O	5.0	Excellent	6.6
570	O=C(Cc1ccccc1)c1nccc2c1[nH]c1c2cccc1	5.1	OK	6.6
13094	O1C(O)C2C(CCC3C4(C(CCC23C)C(CCC4)(C)C)C)C1O	5.4	Excellent	6.6
12711	O=C1CC2CCC3C4CCC(C(COC(=O)C)C)C4(CCC3C2(C=C1)C)C	5.2	Good	6.6
12617	S(=O)(=O)(n1cc(c2c1cccc2)C=O)c1ccccc1	5.7	OK	6.6
11792	O1C2CCC1(C)C(O)C(O)c1c(occ1C)CC(=CCCC2C)C	4.3	Excellent	6.6
10413	O1C2CC(=CC(=O)C(OCC)CC(=O)CC(CC=C(C2O)C1=O)C(C)=C)C	5.4	Excellent	6.6
9555	O=C1N(CCc2c1[nH]c1c2cccc1)C(=O)c1ccccc1NC	4.5	Poor	6.6
8542	O1C2(O)C(=CC1=O)CC1C(CCC(=C1)C)C2(C)C	4.5	Poor	6.6
7932	O1C(=O)C(=C2CC3C(C4OC3(CC4)C)(CCC=C(CC12OC)C)C)C	5.7	Good	6.6
7540	Brc1cc(ccc1O)C1=C(Cl)C(OC1=Cc1cc(Br)c(O)cc1)=O	5.3	Excellent	6.6
7058	O=C1CC2CCC3C4CCC(C(C(O)C(O)C=C(C)C)C)C4(CCC3C2(C=C1)C)C	5.3	OK	6.6
6997	O1C(O)C2=C(CCC3C4(C(CCC23C)C(CCC4)(CO)C)C)C1=O	4.6	Poor	6.6
5896	OC1(CC(c2c(C1)ccc(C)c2C=CC=CC(O)=O)C)C	5.3	Excellent	6.6
5704	O(C(=O)C)C1CC2=CC(O)C3C4CCC(C=C)C4(CCC3C2(CC1)C)C	5.0	OK	6.6
5115	OC(=O)[C-]1[NH+]2C(CCCC2)=CC=C1c1ccccc1	5.1	Excellent	6.6
4882	O1C2(C3C(CCC2C2(CC(CCC2)(C)C)C)C(OC3O)O)C1C	4.6	Excellent	6.6
4843	O1CC2C(CCC(O)(C=CCC2=C)C)C(=CC=CC(O)(C)C)C1OC	5.7	Good	6.6
3895	Brc1cc2c3c4c(ncc3)C(=O)C(N)=Cc4nc2cc1	5.4	Excellent	6.6
3568	O(c1cc(ccc1OC)C1C=C(CC1)C(OC)=O)C1CCCC1	5.0	Good	6.6
3444	O1C2CCC1(C)C(O)CCC(=CC=C(CCC2(O)C)C(C)C)C	4.5	Excellent	6.6
3344	O1CC(=CC1=O)C=CC=C(CCC=1C2(C(CCC=1C)C(CCC2)(C)C)C)CO	4.7	Good	6.6
1914	OC1=C(CC2(C3CCCC(=C)C3(CCC2C)C)C)C(=O)C(NCCCCC)=CC1=O	4.8	OK	6.6
1805	O1C2(CC(=O)C=CC2=O)CC2C3(C(C45C(CC3)C(CCC4)(C)C(OC5)=O)CCC12C)C	4.3	Bad	6.6
1388	OC1CC(CCC(C)C(O)CCC(CC(=O)CC1C)C)C(C)=C	5.1	Excellent	6.6
13558	O1C2CCC3(C1)C(C(OC)=O)C(CCC23C)C(=CCC(O)C(O)(C)C)COC(=O)C	4.3	Good	6.6
11890	O(C)C1=C(C)C(=O)c2c(C1=O)c(ncc2)COC(=O)C(=CC)C	5.2	Excellent	6.6
10683	OC1C2C(CCC2C(OC(=O)C)(C)C)(C=CC(CC(O)C=C(C1)C)C)C	4.4	Poor	6.6
7777	O1C2C1(CC=C(CCC=C(CCC=C(C)C)C)C)C(=O)C=C(COC(=O)C)C2O	3.4	Poor	6.6
7743	OC12C(O)(C)C(=O)C34C(C1=O)C(CCC3C(CC4C2C(C)=C)C)C	5.0	Excellent	6.6
5754	S(OC1=C(C)C(OC1CC(=CCCC(C=CC=C(CCCc1ccoc1)C)C)C)=O)(O)(=O)=O	4.7	Bad	6.6
5201	S(C)C=1N(CN(C)C=1C(=O)c1nccc2c1[nH]c1c2cc(O)cc1)C	4.7	Good	6.6
4674	OC1CCC(C2CC=C(C(=O)C)C(C=O)C12C)(C(CC=C(CC)C)C)C	5.6	Poor	6.6
3907	O1C2(CCC1(C=CC(O)(CCC=C(CCC2O)C)C)C(C)=C)C	5.7	Excellent	6.6
3683	O1CC(CC(OC)=O)C(C(=C)C2CCC3C2(CCCC3(C)C)C)C1=O	5.6	Excellent	6.6
3446	O1C2CCC(O)(C1CCC(=CC=C(CCC2(O)C)C(C)C)C)C	4.3	Excellent	6.6
13009	O1C23C4C(CCC4C)C(=CC2CC(OC3=C(C)C1=O)(CCCc1ccoc1)C)C	5.6	Excellent	6.5
12325	OC1CC2C(C3CC=C(C=O)C(C=O)C13C)(CCC1C(CCCC12C)(C)C)C	4.1	OK	6.5
12105	OC1CC2C(C3CC=C(C=O)C(C=O)C13C)(CCC1C(CCCC12C)(C)C)C	3.8	Poor	6.5
11941	O1CC(C2CC(C)C(OC(=O)CCC)C3C2C2OC3CC(=C)C(O)CC(OC(=O)C)C12C)C	4.7	Good	6.5

11830	O1OC(CCC1(CCC1(C2CCCC(=C)C2(CCC1C)C)C)C)C(C(O)=O)C	4.6	Bad	6.5
11506	o1cc(cc1)C(OC(=O)C)CC1C(CC2C1(CCC1C(CCCC12C)(C=O)C)C)(C=O)C	4.0	OK	6.5
11464	O(C(=O)C)C1C2C(C3CCC(C=C)C3(C1)C)CCC1=CC(=O)CCC12C	4.6	Poor	6.5
11378	Brc1cc2n3c(-c4[nH]c5c(c4C=C3)cccc5)c(c2cc1)C(=O)C(OC)=O	4.0	Poor	6.5
10544	O1C(=O)C(=CC1=CC(CC=CC(=CCCc1cc(oc1)Cc1ccoc1)C)C)C	5.0	Poor	6.5
10285	O1C2C(CCC(=CCCC(=CCCC(=C2)C)C(O)=O)C)C(=C)C1=O	4.2	Excellent	6.5
10075	Oc1ccc(cc1CC1C2(C(CCC1=C)C(CCC2)(C)C)C)C(O)=O	5.1	Excellent	6.5
7778	O1C2C1(CC=C(CCC=C(CCC=C(C)C)C)C)C(=O)C=C(COC(=O)C)C2=O	3.5	Poor	6.5
6572	O1C2C(=CC1=O)CCC1C2(CCC2C3(C(CCC12C)C(CCC3)(C)C)C)C=O	4.5	Excellent	6.5
4448	O1C=2C(=CC13C1(C(CCC3C)C(CCC1)(C)C)C)C=C(O)C(=O)C=2C=O	4.7	Good	6.5
3649	OC1CC2=CCC3C4CCC(C(CCC(OC)=O)C)C4(CCC3C2(CC1)C)C	4.2	Poor	6.5
3347	O1C(CC=C(CCC=2C3(C(CCC=2C)C(CCC3)(C)C)C)CO)C(=CC1=O)CO	4.4	Bad	6.5
3122	O1C(C)(C)C(O)CC(=O)C1(CCCC(=CC(=O)CC(=CCc1cc(O)cc(C)c1OC)C)C)C	6.2	Poor	6.5
2591	O1C2OC(=O)CC2C(C2(C3C(CC2)C(CCCC3=C)(C)C)C)C1O	4.0	Bad	6.5
1938	O1C2CC(OC(=O)C)C3(C(Cc4c(occ4C)C=C(C)C(O)CC3)C12C)C			6.5
1421	O1OC(CCC1(CCC1(C2CCCC(=C)C2(CCC1C)C)C)C)C(C(O)=O)C	4.2	Good	6.5
1281	O1C2C3C(C1CC(=CCCC2(OC(=O)CCC)C)C)C(=C)C(O)CC3C(C)C	4.7	OK	6.5
627	O1C2OC(=O)C3C2C2(C(CC3)C3(C(C(CCC3)(C)C)C(OC(=O)CCC)C2)C)C1O C(=O)C	5.8	Good	6.5
551	O1C23OC4C(OC(=O)C4(O)CCC(CC1(O)C(=C2)C)C(C)=C)CC(=C3)C	5.3	Excellent	6.5
12858	O1C2C1(C)C(=O)CC(CCC1=CC(OC1=O)CC(=CC2=O)C)C(C)=C	5.3	OK	6.5
12170	O1C2C(CC(OC(=O)C)C(=CCCC(=CCCC(=C2)C)C(OC)=O)C)=C(C)C1=O	6.4	Good	6.5
11164	Clc1ccc(cc1)C(C(C)C)C(OC(C#N)c1cc(Oc2ccccc2)ccc1)=O	5.9	Poor	6.5
11029	Clc1ccc(cc1)C(C(C)C)C(OC(C#N)c1cc(Oc2ccccc2)ccc1)=O	6.2	Good	6.5
10653	O1C(CC(=CCCC(C=CC=C(CCCC2=CCN(CC(O)=O)C2=O)C)C)C)C(O)=C(C)C 1=O	5.5	Bad	6.5
9195	O(O)C1(CCC2C3C(C)(C(=O)CC2(C=C1)C)C(=O)C=C3C(C)C)C	6.5	Poor	6.5
8423	O1C2CCC1(C)C(O)C(O)C=1C(OC(=O)C=1C)CC(=CCCC2C)C	4.8	Bad	6.5
8409	O1C(=CC(CCCC(=CCCc2cc(oc2)Cc2ccoc2)C)C)C(O)=C(C)C1=O	5.6	Bad	6.5
8263	OC1(CCC(O)CC1=CC(=O)C1C2CCC(C(CCCC(C)C)C)C2(CCC1)C)C	5.6	Good	6.5
8083	ClC1C(C2C(CC1Cl)(C)C(CC(O)C1CC(=O)NC1=O)C(=CC2=O)C)(C)C	5.5	Excellent	6.5
7523	OC1CCC2(C(CCC3(C2CC(=O)C3=C(C=CC=C(C=O)C)C)C)C1(CO)C)C	5.1	Good	6.5
6681	O1CC=2C3(C(CCC=2)C(CCc2ccoc2)(C)C(CC3O)C)C1O	3.9	OK	6.5
6610	O1C23C(CCC(C2)=C)C2(C(C(=O)C(CC12O)C(C)C)C(=C3)C(OC)=O)C	5.2	Good	6.5
4717	ClC1C(C2CC(=O)C3=C(C2(CC1O)C)C(OC3)=O)(CCC(C(C)=C)C)C	4.4	Poor	6.5
2990	O1C2C1(CCC=C(CCC1(C)C(C)C(O)C=C(C1(O)C)C2=O)C)C	4.5	Excellent	6.5
1007	O1C(=O)C(C)=C(CC1C(C)C1CCC2C3C(CCC12C)C1(C(=CC(=O)CC1)CC3)C) C	5.5	Poor	6.5
435	O1OC(CCC1(CCC=1C(C2C(CC=1C)C=C(CC2)C)(C)C)C)C(C(O)=O)C	4.6	Good	6.5
14490	O1C2CC(=O)CC(CCC34OC3C(OC4=O)CC1(CC2=O)C)C(C)=C	5.5	Excellent	6.5
10934	O(C(=O)C)C1CC2CCC3C4CCC(C(C=CC(OC)=O)C)C4(CCC3C2(CC1)C)C	4.5	Bad	6.5
8925	O=C1C2CC(=O)CCC2(C2C(C3CCC(C(O)(C=CCC(C)C)C)C3(CC2)C)C1)C	5.1	OK	6.5
8128	OC1C2CC(O)CCC2(C=2C(C3CCC(C(CC(=O)CC(C)C)C)C3(CC=2)C)C1)C	4.3	Poor	6.5
7936	OC1=C(C)C(=O)C2=C(C1=O)C(CCC2C(CC=CC(O)(C)C)C)C	4.9	Bad	6.5
7272	O1C(=O)C2(C(C3C4(C(CCC(C)C4=O)C(C3)C)C2=O)C1(C)C)C	4.7	Excellent	6.5
7270	OC12C3(C(CCC1C)C(CC3C=C(C)C)C(=O)C(C)=C(O)C2=O	4.7	Good	6.5
6267	O1C2C=C(CCC(C(C)=C)C(=O)C(O)C(=CC(=O)C3CC23C)C)C1=O	4.9	Excellent	6.5
5993	O1C2C(CC3OC2C(CCCC(OO)(C=CCC3(O)C)C)C)C(=C)C1=O	5.3	Poor	6.5
5819	O1CC=C(CC(OC(=O)C)C2C3(C(CCC2=C)C(CCC3)(C)C)C)C1=O	4.5	Excellent	6.5
5023	ClC1CCC(OC1(C)C)(C)C1C2C(CCC(O)(C)C2[NH+]=[CH-])C(NC=O)(CC1)C	4.8	Good	6.5
4829	O1C2CCC1(C)C(O)CC1C(OC(=O)C1=C)C(OC(=O)C)C(CCCC2=C)C	5.5	Good	6.5
4823	O1C2CCC1(C)C(O)CC1C(OC(=O)C1=C)C(OC(=O)C)C(CCCC2=C)C	6.8	Good	6.5

4714	ClC1C(C2CC=C3C(C2(CC1O)C)C(OC3)=O)(CCC(C(C)=C)C)C	5.2	Good	6.5
4669	O1C2(C3OC(C4C3C(CCC4(O)C)C(C)C1=O)CC(=C)C(O)CC2OC(=O)CCCCC CC)C	5.5	OK	6.5
4613	O1C2C1(CCC1OC1(CCC1(C)C(O)(C(=CCC1C)C2=O)C)C)C	4.5	Excellent	6.5
4559	O1C23C1C(O)C=1C4CCC(C(C=CC(C(C)C)C)C)C4(CCC=1C2(CCC(O)C3)C)C	5.1	Poor	6.5
2976	O1C2C3C(C1CC(=C)C(OC(=O)C)CCC2(O)C)C(=CCC3C(C)C)C	4.9	Poor	6.5
2809	O(C)C1=CC(=O)C(O)=C(CC2(C3C(=CCC2C)C(CCC3)(C)C)C)C1=O	4.4	Excellent	6.5
2805	O1C2C=C(CC(=O)C(Cc3oc(cc3C(OC)=O)C2C(C)=C)C(C)=C)C1=O	3.9	Poor	6.5
2224	O1C2C=C(CCC(Cc3oc(cc3C(OC)=O)C2C(C)=C)C(C)=C)C1=O	4.6	Good	6.5
2120	O1CC(C2CCC3(C(CCc4c3coc4)C2(CC1=O)C)CO)(C(O)=O)C	5.3	Good	6.5
1787	s1c2c(nc1)c1nccc3-c4c(Nc(c13)c2CCN(C)C)cccc4	4.0	OK	6.5
1656	O1C(C=CC2C(CC=CCCCC1=O)C(=O)CC2O)CC=CCC	6.2	Good	6.5
1523	O1C2CC(=O)C(C3C(C1)C(OC(=O)C)CC3C(=CCC=C(C)C)C)C2C			6.5
1464	Oc1ccc(cc1)C1CC(CCC1C(C)C)(C)c1ccc(O)cc1	5.6	OK	6.5
345	O1C2C(C(=C)C1=O)C(O)CC(=CCCC(=CC(OC(=O)C)CC(=C2)C)C)C	5.6	Excellent	6.5
13561	O1C2CCC(=C)C(C(OC)=O)C(CCC12C)C(=CCC(O)C(O)(C)C)COC(=O)C	5.4	Good	6.5
13464	O1CC2C(CCC3(OC3CCC2=C)C)C(=CC=CC(O)(C)C)C1=O	6.0	Excellent	6.5
12467	O1CC2=C3C(O)CC(C)C2(CCC1(C)C(O)CCC1(OC1C3=O)C)C	5.1	Excellent	6.5
12320	O1C2(CCC=C(CCC(=CC=C(CCC2OC(=O)C)C(OC)=O)C(C)C)C1=O)C	5.7	Excellent	6.5
11953	O1C2C1(CCC1C(C(CC2O)=C)C(OCC1=CC=CC(O)(C)C)=O)C	5.0	Poor	6.5
11410	O(C)C1=CC(=O)C(O)=C(CC2(C3CCC=C(C)C3(CCC2C)C)C)C1=O	4.7	Good	6.5
9223	O1C(OC)(C=2OC34CCCC(=C)C3(CCC(C)C4(CC=2C1=O)C)C)CC(OC)=O	5.8	Good	6.5
6582	O(C)c1cc2C3=NC=Cc4c([nH]c5c4cccc5)C3=Nc2cc1	4.3	Poor	6.5
6473	S=C=NC1C2C(CCC1(O)C)C(CCC2C1(OC(CC1)C([NH+]=[CH-])(C)C)C)=C	4.3	OK	6.5
6356	O(C(=O)C)C1CC2(C(CC=C(C=O)C2COC(=O)C)C(C1)(C)C)C	5.1	Poor	6.5
6264	O1C2C=C(CCC(C(C)=C)C(OC(=O)C)C(=O)C(=CC(=O)C3CC23C)C)C1=O	5.8	Good	6.5
6016	O(C(=O)C)C1CC2C(CCCC2(C2CCC(C(OC)=O)C(O)C12C)C)(C)C	4.7	Excellent	6.5
5624	ClC1(CCC2C(CCC3C(CCCC23C)(C)C)(C)C1C(OCC(OC(=O)C)CO)=O)C	4.5	OK	6.5
5478	O(C)C1(CCC=C(CC(=O)C(CCC(C=C1)C(C)C)(CO)C)C)C	4.6	Poor	6.5
5291	O1C2C(=C(C)C1=O)C(O)C1C(C)(C(OC(=O)CCC)CCC(=C2)C)C(OC(=O)C)CC =C1C	3.8	Poor	6.5
5065	Oc1ccc(cc1)CCNC1=CC2=NCCc3c2c(n(c3)C)C1=O	4.1	Excellent	6.5
4957	O1C=C(C2C(C(CCC=C(CC2)C)=C)C1OC(=O)C)C(O)CC(O)C(C)=C	4.6	Bad	6.5
4653	O1c2c(cc(O)cc2)C=CC1(CCC1C(CC(=O)C=C1C)(C)C)C	5.3	Bad	6.5
4293	O=C1C=C(c2nc3c(c4c2c1ncc4)cccc3)CCNC(=O)C	5.3	Excellent	6.5
3655	O1C(C2C(C=CCCCCCCCC1=O)C(O)C(O)C2)CC	5.2	Excellent	6.5
3321	O1CC(=CC1=O)C(O)CC=C(CCC=1C2(C(CCC=1C)C(CCC2)(C)C)C)CO	5.3	Good	6.5
2326	O1C(C=CC2C(CC=CCCCC1=O)C(=O)C=C2)CC=CCC	4.9	Poor	6.5
2251	O1C(C=CC2C(CC=CCCCC1=O)C(O)CC2OC(=O)C)CC=CCC	5.7	Good	6.5
2206	O1C2CCC(O)(C=CCC(=CC3OCC(=C3CCC12C)C)C)C	4.9	OK	6.5
1788	s1c2c(nc1)c(c1Nc3c(-c4c1c2ncc4)cccc3)CCNC	4.2	OK	6.5
1593	S1CC(NC1=O)C1(OC2CCC(C=CC3OC3CCC(=CC(OC(C1)C2)=O)C)C)O	4.1	Poor	6.5
1547	S(C)c1c(c2Nc3c(-c4c2c(ncc4)c1OC)cccc3)CCNC(=O)CC	6.1	Good	6.5
1533	O(CC)C1=CC(=O)C(O)=C(CC2(C3CCCC(=C)C3(CCC2C)C)C)C1=O	4.7	Good	6.5
981	O(C)C1=CC(=O)C(O)=C(CC=C(CCC2(CCCC(C)C2=C)C)C)C1=O	5.3	Good	6.5
465	O1C2CC(=CC(=O)C=C(C)C(=O)CC(CCC(=C2)C1=O)C(C)=C)C	5.6	Poor	6.5
13463	O1CC2C(CCC(=CCCC2=C)C)C(=CC=CC(O)(C)C)C1=O	5.0	Excellent	6.5
11806	O1C(=O)C(c2c(c(ccc2C2(CC(CCC2)(C)C)C)C=O)C1OC)C	5.7	Good	6.5
11326	Clc1cc(N=C2C=C(Oc3c2cc(Cl)cc3)c2ccc(OC)cc2)ccc1OC	6.5	Good	6.5
10410	OC1CC2=CCC3C4CCC(C(=NO)C)C4(CCC3C2(CC1)C)C	4.3	Good	6.5
9677	Oc1cc(c2c(CC3C4(C(CCC23C)C(CCC4)(C)C)C)c1O)COC	5.5	Excellent	6.5
9608	Oc1c2c(ccc1)C(=O)c1c(c(cc(O)c1)C)C2=O	4.2	Excellent	6.5

9197	O(O)C1(CCC2C3C(C)(C(O)CC2(C=C1)C)C(=O)C=C3C(C)C)C	5.0	Poor	6.5
8493	O=C1N2C(CCC2)C(=O)NC1Cc1c2c([nH]c1)cccc2	4.3	OK	6.5
7723	O1CC2=C(C3(C(CC2OC(=O)C)C2(C(CC3O)C3(C(CC2)C(CCC3)(C)C)C)C)C)C1=O	4.2	Bad	6.5
6227	O1CC=2C(C3(C(C4C(CC3O)C3(C(CC4)C(CCC3)(C)C)C)CC=2)C)C1O	4.9	Bad	6.5
5705	O(C(=O)C)C1C2C3CCC(C=C)C3(CCC2C2(C(CC(O)CC2)=C1)C)C	4.4	OK	6.5
5100	O1C2C3OCC(=C3CCC(=CCCC(O)(C1CCC2(O)C)C)C)C	4.9	Excellent	6.5
4724	O1CC2C(CCC(O)(C=CCC2=C)C)C(=CC=CC(O)(C)C)C1=O	5.6	OK	6.5
4710	OC1CC2=CC(=O)C3C4CCC(C(=O)C)C4(CCC3C2(CC1)C)C	5.3	Poor	6.5
4245	O=C1C2C(CCC3C(CCCC23C)(C)C)(C)C(COC(=O)C)C(=C1)COC(=O)C	4.6	Good	6.5
964	OC12C(CCCC1(C)C)C(CCC(=CCn1c3c(nc1)N(CN(C)C3=N)C)C)(C)C(CC2)C	3.7	Bad	6.5
14352	S=C(NC1(C2C(C3C(CC1)C3(C)C)C(CC2)C)C)NCCc1ccccc1	4.2	OK	6.5
14351	S=C(NC1(C2C3C(CCC2(CCC1)C)C3(C)C)C)NCCc1ccccc1	5.5	Poor	6.5
13858	O1C2(O)C(=CC1=O)C(C1C(C2)C=C(CC1)C)(C)C	5.1	Excellent	6.5
13723	O1c2c(C=CC1(CCC1(CCC=C(C)C1C)C)C)c(O)c(OC)c(OC)c2OC	4.3	Excellent	6.5
13468	O1CC23C(CCC4(C5Cc6cc(O)ccc6OC5(CCC24)C)C)C(CCC3)(C)C1O	4.3	Bad	6.5
10368	O(C(=O)C)C1C=C2C(CC3(O)C1(CCCC3=C)C)(CCC2(O)C(C)C)C	4.9	OK	6.5
10357	[NH+](C1(C2C3C4C(CC2)C([NH+]=[CH-])(CCC4C(CC3CC1C)C)C)C)=[CH-]	5.5	Excellent	6.5
9777	O1C2=CC(C)(C(O)C(CCC3=CC(OC3=O)C2C(C)=C)C(C)=C)C1=O	5.0	Excellent	6.5
8829	OC1CCC2(C(CC(=O)C3=C2CCC(C3)(C=C)C)C1(CO)C)C	5.5	Good	6.5
7947	ClC1CC2C(C3(C)C1(O)CC=CC3=O)C(O)CC1(C2CCC1C(CCC(C(C)C)C)C)C	5.5	Bad	6.5
7183	Brc1c(cc2C3C(CCC3C(C)C)(C)C(=O)C(=O)c2c1O)C	5.7	Excellent	6.5
6566	OC1(CCC2C(CCC3C(CCCC23C)(C)C)(C)C1C(OCC(O)COC(=O)C)=O)C	5.0	Bad	6.5
5340	O1C(O)(CC)C(C)C(OC(=O)C(C(O)C(C(=O)CC)C)C)C(C)C1C(C)C	4.0	Good	6.5
4665	O1C2OC2C(CCC=C(CCC=C(CC(O)C=C(C)C)C)C)C1=O	4.9	Bad	6.5
4518	OC1(CCC=C(CC(=O)C(CCC(C=C1)C(C)C)(COC(=O)C)C)C)C	4.5	Poor	6.5
3242	O1C23CC(OC(=O)C)CCC2(C)C(=O)C(=CC13)C1CCC(C(C=CC(C)C)C)C1(CCOC(=O)C)C	4.4	Poor	6.5
3038	O1c2c(cc(O)cc2C)C=CC1(C=CC=C(C=O)C)C	5.6	Excellent	6.5
2123	o1cc2c(CCC3C4(C(CCC23C)C(C)=C(O)C(=O)C4)C)c1	5.1	Excellent	6.5
1600	O1C(C2C3(CC=C4C(CCC5C(C)(C)C(O)CCC45C)C3(CC2=O)C)C1=O)(CC(=O)CC(C)C)C	5.6	Good	6.5
1548	S(C)c1c(c2Nc3c(-c4c2c(ncc4)c1OC)cccc3)CCNC(=O)C	4.2	Excellent	6.5
11048	O1C2=CC(=O)C=3NC(CC(OC)C=3C2=Nc2c1cccc2)C	4.6	Excellent	6.5
6569	O1C(=CC(N)=C(C=O)C1=O)C1C2C(CC(O)CC2)C=CC1C	6.4	Excellent	6.5
4677	O(C(=O)C)C1CCC(C2CC=C(C(=O)C)C(C=O)C12C)(C(CC=C(CC)C)C)C	4.5	Bad	6.5
4550	O(C)c1ccc(O)c(O)c1CC=1C2(C(CCC=1C)C(CCC2)(C)C)C	5.8	Good	6.5
4517	OC1(CCC=C(CC(=O)C(CCC(C=C1)C(C)C)(CO)C)C)C	6.1	Excellent	6.5
3747	O1CC=2C(C3(C(CC=2)C2(C(CC3OC(=O)C)C3(C(C(CCC3)(C)C)C(=O)C2)C)C)C)C1O	4.3	OK	6.5
3060	O(C(C(=CC(OC(=O)C)OC(=O)C)C=O)CC=C(C#CC=C(C)C)C)C(=O)C	4.7	Good	6.5
2426	OC12CC(O)CCC1(C1C(C3CCC(C(CCC(C(C)C)C)C)C3(CC1)C)CC2=O)C	4.4	Poor	6.5
1003	O=C1NC(Cc2c3c([nH]c2)c(ccc3N(C)C1C(C)C)C(CCC=C(C)C)(C=C)C)COC	4.5	Bad	6.5
13770	O1C2CCC(CCC(CC(O)C=C(CC1=O)C)=C)C(C)(C)C2=O	5.2	Excellent	6.4
12323	O1C2OC(O)C3C2C(CC3)C(CCC=C(CCC=C(C)C)C)C1O	5.2	Good	6.4
9454	Clc1ccc(cc1)C(C(C)C)C(OC(C#N)c1cc(Oc2ccccc2)ccc1)=O	4.8	Good	6.4
8790	O1CC(=CC1=O)C(O)CC=C(CCC1(C2CCCC(=C)C2(CC(OC(=O)C)C1C)C)C)C	5.2	Bad	6.4
8603	O1C23C1C(O)C1C4CCC(C(CCC(C(C)C)=C)C)C4(CCC1C2(CCC(O)C3)C)C	4.4	Bad	6.4
6307	O1OC(CCC1(CCC1=C(C)C(=O)CC2C1(CCCC2(C)C)C)C)C(C(OC)=O)C	5.1	Bad	6.4
5503	s1c2c(nc1)-c1nccc3c1c(nc1c3cccc1)C2(O)CCNC(=O)CC	4.8	Bad	6.4
4930	O1CC(C2C3C(C4OC3C1(C)C(=O)CC(O)C(C4)=C)C(OC(=O)CCCCCCC)(CC2)C)C	5.1	Poor	6.4

3306	O1CC1(C(C)C)CCC(C)C1CCC2C3C(CCC12C)C1(C(CC(O)CC1)=CC3O)C	3.6	Poor	6.4
3187	O(CCCC)C1=CC(=O)C(O)=C(CC=C(CCC2(CCCC(C)C2=C)C)C)C1=O	4.7	Bad	6.4
2807	O1C2C(c3oc(CC(C(C)=C)C(O)C=C(C2O)C1=O)c(c3)C(OC)=O)C(C)=C	6.1	Good	6.4
2382	O1C2OC(=O)CC(C2C(=C)C2CCC3C2(CCCC3(C)C)C)C1OC(=O)C	5.6	OK	6.4
2245	O1C(C=CC2C(CC=CCCCC1=O)C(O)CC2OC(=O)C)CCCCC	4.9	Good	6.4
1429	O1C2C3C(C1CC(=C)C(O)CCC2(OC(=O)CCC)C)C(CCC3C(C)C)=C	4.9	Good	6.4
368	O1C2OC(=O)CC(C2C2(CCC3C(=CCCC3(C)C)C2C)C)C1OC(=O)C	3.9	Bad	6.4
164	O1C(OC(=O)C)c2c(ccc(C3(CC(CCC3)(C)C)C)c2C(=O)C)C1=O	4.6	Good	6.4
13761	O1C(C)(C)C1CC=C1C2C(C(CC(O)C=C(CC2)C)=C)C(OC1)=O	5.3	Poor	6.4
13433	OC1=C(CC2C3(C(CC=C2C)C(CCC3)(C)C)C)C(=O)C=CC1=O	5.5	Excellent	6.4
12965	O1C2C=C(C3OC3C(Cc3oc(cc3C(OC)=O)C2C(C)=C)C(C)=C)C1=O	5.4	Excellent	6.4
12080	O1C(CC=C(CCC=C(CCC=2C(CCCC=2C)(C)C)C)C1O)C1=CC(OC1O)=O	3.6	Bad	6.4
11847	ClC1CCC2(C(C1(C)C)C(=O)CC(=C)C2CC(O)C1CC(=O)NC1=O)C	4.6	Good	6.4
11590	o1cc2c(CCC3C4(C(CCC23C)C(COC(=O)C)(C)C(OC(=O)C)C(=O)C4)C)c1	4.0	OK	6.4
10680	OC1(CC(O)C2C(CCC2C(OC(=O)C)(C)C)(C=CC(CC=C1)C)C)C	4.7	Poor	6.4
9137	O(C(=O)C)C1CC2C(C3CC(O)C(C(=O)C)C(O)C13C)(CCC1C(CCCC12C)(C)C) C	5.9	OK	6.4
7296	O1C23C1C=CC(=O)C2(C1C(C2CCC(C(C=CC(C(C)C)C)C)C2(CC1O)C)CC3O) C	4.5	Poor	6.4
6890	O1C23C1C(O)C=1C4CCC(C(C=CC(C(C)C)C)C)C4(CCC=1C2(CCC(O)C3)C)C	5.0	Poor	6.4
6547	O1C(OC)C2=C(C3(C(CC2)C2(C(CC3)C(CCC2)(C(O)=O)C)C)C)C1=O	5.1	OK	6.4
6409	OC1=C(C)C(=O)C23C(C1=O)C(CCC2C(CC3C=C(C)C)C)C	4.4	Excellent	6.4
6054	O1C23C(CCC(C2)C)C2(C(C(=O)C(CC12O)C(C)C)C(=C3)C(OC)=O)C	5.1	OK	6.4
5175	O1C(CCC(C)C)C(C2C3(C(CC2=O)C2C(CC3)C3(C(=CC(=O)CC3)CC2)C)C1O) C	4.3	OK	6.4
4922	O1C2CC(=C3C2C(C2=C(CC(=O)CC(C2)C(C)=C)C3=O)C1=O)C	5.8	Good	6.4
4671	O1C2(C3OC(C4C3C(CCC4(O)C)C(C)C1=O)CC(=C)C(O)CC2OC(=O)CCCCC CC)C	5.0	Poor	6.4
3319	O1C(O)C(=CC1=O)C=CC=C(CCC=1C2(C(CCC=1C)C(CCC2)(C)C)C)CO	4.9	Good	6.4
1389	O=C1CC(CCC(C)C(=O)CCC(CC(=O)CC1C)C)C(C)=C	4.8	Excellent	6.4
952	O1C(CC=C(CCC=C(CCC2(CCCC2C)C(C)=C)C)C1O)C1=CC(OC1)=O	3.6	Bad	6.4
14215	O1C(OC(=O)C)C2C(CC=C(C3(CC(CCC3)(C)C)C)C2=CC)C1OC(=O)C	4.2	OK	6.4
13717	O1CC1(C(C)C)CC(O)C(C)C1CCC2C3C(CCC12C)C1(C(CC(O)CC1)=CC3)C	5.1	Bad	6.4
13677	O1C2C(CCC(=CCCC(OC)(C)C(O)CCC(=C2)C)C)=C(C)C1=O	5.1	Bad	6.4
13186	O1C2C(CC3OC3(CCC=C(CCC=C(C)C2O)C)CO)C(=C)C1=O	4.9	Poor	6.4
12239	OC1=C(CC2(C3CCCC(=C)C3(CCC2C)C)C)C(=O)C(NCC(C)C)=CC1=O	4.5	Bad	6.4
11875	OC1C=C2C3(C(C1C)C(O)=CC(=C3)C)C(CC2C)C=C(C)C	4.2	Excellent	6.4
10974	O=C(C(=O)c1c2c([nH]c1)cccc2)c1c2c([nH]c1)cccc2	5.7	Poor	6.4
10601	Oc1ccc(cc1)C=1N=C(C=2N(C=1)C(=O)C(N=2)=Cc1ccc(O)cc1)Cc1ccccc1	4.8	Bad	6.4
10412	O1C2CC(=CC(=O)C(OCC)CC(=O)CC(CC=C(C2O)C1=O)C(C)=C)C	5.9	Excellent	6.4
9225	O1C(OC)(C=2OC3(CCC4(C(CCCC4=C)C3(CC=2C1=O)C)C)C)CC(OC)=O	6.0	OK	6.4
8864	O1CC2C(CCC(=C)C(O)CCC2=C)C(=CC=CC(O)(C)C)C1=O	5.1	Excellent	6.4
8465	OC1C2CC(O)CCC2(C=2C(C3CCC(C(=O)C)C3(CC=2)C)C1)C	4.6	Excellent	6.4
7561	O1C(c2c(c3CC(Cc3cc2C)(C)C)C1=O)CO[N+](=O)[O-]	5.3	OK	6.4
7545	Oc1cc(c2c(CC3C4(C(CCC23C)C(CCC4)(C)C)C)c1O)C(OC)=O	5.1	OK	6.4
5521	O(C)c1cc(cc(CC2(C3CCCC(=C)C3(CCC2C)C)C)c1O)C(OC)=O	5.5	Excellent	6.4
4984	O1C2CCC(C)C1(CCC1C34OC(O)(CC3)C(OC4CCC1C)(C)C)C2(CCC(=O)C(C) C)C	5.3	Poor	6.4
4598	O1C=2C3C=C(CCC(OC(=O)C)C1(CCC(=O)C=2CCC3(O)C(C)=C)C)C	5.6	Excellent	6.4
3730	o1c2c3c(c1)C(=O)CCC3(c1c(cc3c(c1)C(=O)C=C(OC)C3=O)C2=O)C	5.8	OK	6.4
3186	O1C2=C(C=CC1(CCC1(CCCC(C)C1=C)C)C)C(=O)C(OC)=CC2=O	5.6	Excellent	6.4
2991	O1C2C1(CCC=C(CCC1(C)C(C)C(O)C=C(C1=C)C2=O)C)C	5.5	Good	6.4

2094	O1C2CC3(C(CCC4(OC4CCC12C)C)C(=CC3)C(O)(C)C)C	5.9	Excellent	6.4
1480	O1CC(C(COC(=O)C)C2(C3C(CC2)C(CCCC3=C)(C)C)C)C(OC(=O)C)C1=O	5.4	OK	6.4
1364	O1C2C3C(C1CC(O)(C)C(O)CCC2(OC(=O)C)C)C(CCC3C(C)C)=C	5.2	Good	6.4
1162	O1C2CC(=CCCC(=C(C2C(CCC=C(C)C)C)C1=O)COC(=O)C)C			6.4
953	O1CC(=CC1=O)C(O)CC=C(CCC=C(CCC1(CCCC1C)C(C)=C)C)C=O	4.6	Poor	6.4
792	O1C(C(=C)C2C3C4C(CCC4C)C3(C)C(O)C2)C1C=C(COC(=O)C)C	4.2	Poor	6.4
629	O1C2OC(=O)C3C2C2(C(CC3)C3(C(C(CCC3)(C)C)C(OC(=O)CCC)C2)C)C1O	5.4	OK	6.4
569	Brc1cc2c3c([nH]c2cc1)c(ncc3)C(=O)Cc1ccccc1	6.2	Good	6.4
564	O1CC(=C2C1C=C(CCC(O)C(O)(CCC=C(CC2)C)C)C)C	4.6	Good	6.4
545	O1C(CC(CCCC(=CC=CC2(CCCc3c2occ3)C)C)=C)C(O)=C(C)C1=O	3.3	Good	6.4
14488	O1C(CC(=CCCC(=CCCc2cc(oc2)Cc2ccoc2)C)C)C(O)=C(C)C1=O	5.2	Bad	6.4
14032	O1C23N4C5(OC(CC(C5)C)C4)CCC2(C)C(C2C(=O)C(C4C(CC(=CC4=O)C)C2(C3)C)C)(CC1=O)C			6.4
13933	O(C(=O)C)C1CC2(C(CC=C(CCC=C1CO)C)C(CC2)C(C)=C)C	4.6	Excellent	6.4
12198	O=C(C)C1CCC2C3C(CCC12C)C1(C(CC(=NO)CC1)CC3)C	3.9	Bad	6.4
11874	OC1CC2C3(C(C1C)C(O)=CC(=C3)C)C(CC2C)C=C(C)C	5.0	Excellent	6.4
11331	O1C(CC=C(CCC=C(CCC=2C(CCCC=2C)(C)C)C)C1OC)C1=CC(OC1O)=O	3.6	Bad	6.4
10366	O(C(=O)C)C1C2C(CCC2C(O)(C)C)(C=CC(CCC=C(C1)C)C)C	4.2	Excellent	6.4
7400	O(C(=O)C)C12C3(C(CC1)C(CCCC)C3C=CC(OC(=O)C)CCC(OC)=O)C(=O)C=C2	6.2	OK	6.4
7000	O1CC=2C(C3(C(CC=2)C2(C(CC3=O)C3(C(CC2)C(CCC3)(C)C)C)C)C)C1O	4.1	Bad	6.4
6565	O(C(COC(=O)C1C2(C(CCC1=C)C1(C(CC2)C(CCC1)(C)C)C)C)CO)C(=O)C	4.6	Bad	6.4
6102	O(C)c1ccc(cc1)CC=1N(C)C(=N)N(C=1)Cc1ccc(O)cc1	3.8	OK	6.4
6033	o1c(-c2c3c([nH]c2)cccc3)c(nc1C(N(C)C)CC(CC)C)C(O)=O	4.3	Good	6.4
6001	O1C(C)C(O)C=CC(OC(CC(OC(CC=CC1=O)C)=O)C)=O	5.5	Excellent	6.4
5957	O1CC2=C(C3(C(CC2)C2(C(CC3O)C3(C(CC2)C(C)(C)C(O)CC3)C)C)C)C1=O	3.9	Poor	6.4
5828	OC(=O)CCc1ncc2c(c1)C1(C(CC2)C2(C(CC1)C(CCC2)(C)C)C)C	6.2	Good	6.4
3616	O1OC(CCC1(CCC1(C)C(CCC2C1(O)CCCC2(C)C)C)C)C(C(O)=O)C	4.6	Bad	6.4
3362	O1C2(CCC=C(CCC(=CC3C2(O)CCC3=C)C(C)C)C1=O)C	4.4	Excellent	6.4
3318	O1C(O)C(=CC1=O)C=CC=C(CCC=1C2(C(CCC=1C)C(CCC2)(C)C)C)C(OC)OC	4.8	Poor	6.4
3285	O1C=2C(=CC1=O)C(OC(=O)C)CC1C(CCC3C4(C(CCC13C)C(CCC4)(C)C)C)C=2	4.6	Bad	6.4
2859	O1C2CCC(=CCCC(=CC3OC(=O)C(C3CC(OC(=O)C)C12C)CN(C)C)C)C	5.3	OK	6.4
1120	O1CC(C(OC(=O)C)C(OC)=O)C(=C1)C1(C2C(CC1)C(CCCC2=C)(C)C)C	4.9	Good	6.4
344	O1C2C(C(=C)C1=O)C(O)CC(=CCCC(=CC(O)CC(=C2)C)C)C	4.4	Good	6.4
14299	O(C(=O)C1C2(C(CC=C1C)C1(C(CC2)C(CCC1)(C)C)C)C)CC(O)COC(=O)C	3.9	Good	6.4
14061	O(C(=O)C)C1CC(C(C(OC(=O)C)CC=C(C)C)C)C(O)C2C(CC=C2C)C1=C	5.5	OK	6.4
13113	O(C(=O)C)CC(CCC=C(CCC1C(CCCC1C)(C)C)C)=CC=O	5.4	Poor	6.4
12351	OC1=C(C)C(=O)c2c(C1=O)c(ncc2)COC(=O)C(=CC)C	5.2	Excellent	6.4
12012	O1C2C=C(C(C)C)C(=O)C(O)C(CCC(=O)C(CCCC12C)CO)C	5.8	OK	6.4
11586	o1cc2c(CCC3C4(C(CCC23C)C(CO)(C)C(O)C(=O)C4)C)c1	5.2	Good	6.4
11175	O1CC2C(C(C(C(O)=O)C)=C(CC2)C2(CC(CCC2)(C)C)C)C1OC	5.4	Poor	6.4
11166	O1CC2C(C(C(C(O)=O)C)=C(CC2)C2(CC(CCC2)(C)C)C)C1OC	5.6	Good	6.4
10959	O1OC(CCC1(CC=CC(O)(CCC=1C(CCCC=1C)(C)C)C)C)C(C(O)=O)C	4.5	Bad	6.4
10823	OC(=O)C1(C2CCC(=C)C(CCC(=CC(O)=O)C)C2(CCC1)C)C	6.0	Excellent	6.4
9837	Oc1ccc(O)cc1CC1(C2CCC=C(C)C2(CCC1C)C)C	5.0	OK	6.4
9550	OC1CC2=CCC3C4CCC(C(CCC(O)=O)C)C4(CCC3C2(CC1)C)C	4.2	Bad	6.4
8966	O1C23C1C(O)C=1C4CCC(C(CCC(C(C)C)CC)C)C4(CCC=1C2(CCC(O)C3)C)C	5.0	Poor	6.4
7161	O1C(CC(=CCCC(C=CC=C(CCCC2=CC(=O)N(C2)CCO)C)C)C)C(O)=C(C)C1=O	5.0	Bad	6.4
7075	O1C2C3=C(C(CCC3C(CC(C=C(C)C)C2(O)C)C)C)C1=O	5.7	Excellent	6.4

6892	O1C2(OC)C(C3C(C(OC(=O)C)CC3C(=CCC=C(C)C)C)C1OC)C(C)C(OC)C2	4.5	Poor	6.4
6810	O1C(C2C3(CC=C4C(CCC5C(C)(C)C(O)CCC45C)C3(CC2=O)C)C1=O)(CCC=C(C)C)C	4.6	Poor	6.4
6640	OC1CCC2(C(CC=C3C2CCC(C3)(C=C)C)C1(CO)C)C	5.9	Poor	6.4
6438	BrC1CC(CCC1(O)C)C(=CC(O)C1C(=C)C(CCC1=C)C)C	5.7	OK	6.4
5438	o1cc(cc1C=C(CCCc1cc(oc1)C=C(C(=O)NCCN)C)C)CCCc1ccoc1	4.0	Bad	6.4
4851	O1CC2C(CCC(O)(C=CCC2=C)C)C(=CC=CC(C)C)C1=O	5.7	Excellent	6.4
4482	O1C2CCC(C3C(CCC12C)C(=CC(C=C(C)C)C3O)C=O)=C	5.1	Excellent	6.4
3929	O1C2C3C(C1CC(O)(C)C(OC)CCC2(O)C)C(CCC3C(C)C)=C	5.2	Excellent	6.4
3335	O(C)C1=CC(=O)C(O)=C(CC=C(CCC2C(CCC=C2C)(C)C)C)C1=O	5.6	OK	6.4
2813	O(C(COC(=O)C)CO)C(=O)CC(CCC=1C2(C(CCC=1C)C(CCC2)(C)C)C)C	3.4	Poor	6.4
2378	O1CC2=C(C3C(C3)C(CC(O)C2C(CCC=C(C)C)COC(=O)C)C)C1=O	5.1	Poor	6.4
2370	O1OC(CCC1(CCC1C2(CCCC(C)(C)C2(O)CCC1C)C)C)C(C(OC)=O)C	5.0	Poor	6.4
2017	O1CC(C(C(=C)C2CCC3C2(CCCC3(C)C)C)COC(=O)C)C(O)C1=O	6.3	Good	6.4
1978	O1C23CC(CCC(=O)C(C)C)(C)C1(O)CCC2(COC(=O)C)C(O)CCC3=C	4.2	Excellent	6.4
1486	O(C(=O)C)C1C2(CCCC(=C)C2(O)CC2(C(C1OC(=O)C)=C(CC2)C(C)C)C)C	4.6	OK	6.4
1124	O1CC(C(CO)C(O)C1=O)C1(C2C(CC1)C(CCCC2=C)(C)C)C	5.5	Excellent	6.4
655	O(C)c1cc2c(c(CCC)c1C(OC)=O)c(O)c1c(c2)C(C)(C)C(=O)C(CCC(=O)C)(C)C1=O	4.1	Bad	6.4
14462	O1C2C=C(CCC(C(C)=C)C(O)c3oc(cc3C)C2C(C)=C)C1=O	3.9	Excellent	6.4
13237	O1c2c(cc(O)cc2C)CCC1(CCC=C(CCC=C(C=O)C)C)C	4.2	Bad	6.4
13188	O1C2C(CC=C(CCC=C(CCC=C(C2)C)C)CO)C(=C)C1=O	4.6	Excellent	6.4
13070	OC1CC2C3C(CCC2(C)C1C(O)(CCCC(C)C)C)C1(C(=CC(=O)CC1)CC3)C	4.8	OK	6.4
12065	O1C(CCC1C(NC=O)(C)C)(C)C1C2C(CCC(O)(C)C2[NH+]=[CH-])C(CC1)(C#N)C	4.1	Poor	6.4
12035	[NH+](C1(C2C3C(C(CC(C3CC1)C)CC([NH+]=[CH-])(C)C)C(CC2)=C)C)=[CH-]	4.8	OK	6.4
10848	o1c2c(cc1)C(O)CC1C2(C)C(OC(=O)C)CC2C1(CCC1C(CCCC12C)(C)C)C	4.9	Poor	6.4
10757	OC1CC2C3C(CCC2(CC1(C=O)C)C)(C)C(=O)C=C3C(C)C	5.3	Excellent	6.4
8267	OC1CC2CCC3C4CCC(C(CCC(O)=O)C)C4(CCC3C2(CC1)C)C	4.5	Bad	6.4
7082	[NH+](C1(C2C3C(C(CC(C3CC1)C)CC([NH+]=[CH-])(C)C)C(CC2)=C)C)=[CH-]	4.4	Good	6.4
6893	O1C2(OC)C(C3C(C(OC(=O)C)CC3C(=CCC=C(C)C)C)C1OC)C(C)C(OC)C2	5.5	Good	6.4
6570	O1C(=CC(N)=C(C=O)C1=O)C1C2C(CC(O)CC2)C=CC1C	5.4	Excellent	6.4
5311	Oc1ccc(cc1)CC1N(C(=O)C=C(OC)CCNC(=O)CCCCC(C)(C)C)C(=O)C=C1	7.1	Poor	6.4
4931	O1CC(C2CC(O)(C(=O)CCCCCCC)C(O)(C3C2C2OC3CC(CCCC12C)=C)C)C	5.0	Poor	6.4
4828	O(C(=O)CC(CCC1(C=2C(CCC1C)C(CCC=2)(C)C)C)C)CC(O)CO	4.9	Bad	6.4
4798	O1C23CC(O)CCC2(C)C(=O)C(=CC13)C1CCC(C(C=CCC(C)C)C)C1(CCO)C	4.8	Poor	6.4
3843	O1c2c(c(cc(OC)c2)C)C(=O)c2c1cc(OC)cc2O	5.0	Poor	6.4
3697	O(C(=O)C1C2(C(CC=C1C)C1(C(CC2)C(CCC1)(C)C)C)C)CC(O)COC(=O)C	4.7	Bad	6.4
2631	Brc1cc(cc(Br)c1O)C1=CC(OC1=Cc1ccc(O)cc1)=O	5.7	Good	6.4
982	O1c2c(C=CC1(CCC1(CCCC(C)C1=C)C)C)c(O)c(OC)cc2OC	4.1	Excellent	6.4
14098	O(C(C)c1c2nc3c(nc2ccc1)c(ccc3)C(O)=O)C(=O)CO	5.6	Excellent	6.4
14097	OC(C)c1c2nc3c(nc2ccc1)c(ccc3)C(O)=O	4.8	Excellent	6.4
13291	O1C2C1(CC1C3(C(CCC1=C)C(CCC3)(C)C)C)C(=O)C=C(CO)C2O	4.8	Excellent	6.4
12975	O(C(=O)C)C1C2C(CCC(C1CC=C2C=O)=C)C(CCC=C(C)C)C			6.4
12785	O(C(=O)C)C1CC2C3(C(CCC2(C2CC=C(C(=O)C)C(C=O)C12C)C)C(CCC3)(C)C)CO	5.7	Poor	6.4
9833	O(C(=O)c1ccccc1[NH+]1C=Cc2c([nH]c3c2cccc3)[CH-]1)C	4.4	Good	6.4
7913	O1C(=O)C(=C2CC3(C(CC12OC)C(OC(=O)C)CCC3C)C)C	4.9	Good	6.4
6842	OC(CO)C1(CC=2C(CC1)C1(C(CC=2)C(CCC1)(C)C)C)C	4.2	Bad	6.4
6375	Brc1cc2[nH]cc(c2cc1)-c1nc(ncc1)N	4.2	OK	6.4

6047	O1C23CC(O)CCC2(C)C(=O)C(=CC13)C1CCC(C(C=CC(C(C)C)C)C)C1(CC=O)C	3.8	Bad	6.4
4775	O(C(=O)CO)C1CC2C(NC1C)CCCC2C=CC=CCCC(=O)C	3.6	OK	6.4
4360	o1c2c(CCC3C(CC(OC(=O)C)CC23C)(CO)C)cc1	4.8	Good	6.4
3265	O1C23CC(O)CCC2(C)C(=O)C(=CC13)C1CCC(C(CCCC(C)C)C)C1(CCO)C	5.2	Bad	6.4
2698	O=C1C=C2CCC3C4CCC(C(O)(C=CCC(C)C)C)C4(CCC3C2(C=C1)C)CO	4.4	Good	6.4
2116	O=C1c2c(cccc2)C(=O)C1=C(NN(C(=O)C)C(OCC)=O)C	4.8	Excellent	6.4
13377	O=C1C=C2CCC3C4CCC(C(C=CC(C(OC)=O)C)C)C4(CCC3C2(C=C1)C)C	4.2	Bad	6.3
8980	O1C2C(CC3OC2C(CCCC(OO)(C=CCC3(O)C)C)C)C(=C)C1=O	6.1	OK	6.3
7370	O1C23C4C(CCC(C2)(C)C(O)CC13)C(=CC(C=C(C)C)C4O)C=O	6.2	OK	6.3
7249	O(C(=O)C)C1C=C2C(CCC3(C2CCC3C(C=CC(C(C)C)C)C)C)C2(CCC(O)CC12O)C	5.8	Bad	6.3
7179	OC1(C(OC)=O)C2(C(c3c(C1=O)c(O)cc(c3)C)C(CC2)C(C)C)C	4.4	Excellent	6.3
6831	OC1(CC(=O)C(CCC(C=CC(O)(CC=C1)C)C(C)C)(COC(=O)C)C)C	5.0	Bad	6.3
6736	O1C(O)C(OC)=C(C(=O)CC2(C3CCCC(=C)C3(CCC2C)C)C)C1=O	4.4	Excellent	6.3
6545	O1C2C1(CC1C3(C(CCC1=C)C(CCC3)(C)C)C)C(=O)C=C(CO)C2OC(=O)CC(O)=O	6.1	Bad	6.3
6012	O1CC23C(CCC(C2COC(=O)C)C1=O)C1(C(CC3)C(CCC1)(C)C)C	5.0	Good	6.3
4960	O1C(C)(C)C1CCC(C)C1CCC2C3C(CCC12C)C1(C(=CC(=O)CC1)C(O)C3)C	5.0	Bad	6.3
4195	O1CC(C2CC(C)C(OC(=O)C)C3C2C(OC3CC(=O)C)C1(C=CC=O)C)C	6.1	Poor	6.3
3720	O=C1CC2C(CCC3C(CCCC23C)(C)C)(C)C(CCC(C(O)=O)C)C1C	6.2	Bad	6.3
2518	O1C2C3C(CCC3(CCC(CC(=O)CC(C2)C)C)C)=C(C)C1=O	4.5	Bad	6.3
1682	S(C)C=1C(=O)c2nccc3c2c(nc2c3cccc2)C=1CCNC(=O)C	5.8	Excellent	6.3
297	o1c(ccc1CO)-c1nc(cc2c1[nH]c1c2cccc1)C(OC)=O	3.7	Bad	6.3
18	O1C(C(C(=O)C(C)C=2OC(CC)=C(C)C(=O)C=2C)C)=C(C)C(=O)C(C)=C1CCCCC	4.2	Poor	6.3
14232	O1C(CCC1C(OC(=O)C)(C)C)(C)C1CCC(C2C(C1O)C(=CC2)C)=C	4.1	Excellent	6.3
12860	O1C2CC(=CC(=O)C=C(C)C(=O)CC(CCC(=C2)C1=O)C(C)=C)C	5.7	Excellent	6.3
12834	O1C(=CC(CCCC(=CCCC(O)(CCCc2ccoc2)C)C)C)C(O)=C(C)C1=O	5.5	Poor	6.3
12712	O=C1CC2CCC3C4CCC(C(OC(=O)C)C)C4(CCC3C2(C=C1)C)C	3.9	Bad	6.3
11790	O1C2CCC1(C)C(O)C(O)c1c(occ1C)CC(=CCCC2C)C	5.4	OK	6.3
11221	O1C2CCC(C3C(CCC12C)C(C3)(C(=O)C=CC(O)(C)C)C)=C	5.7	Excellent	6.3
9224	O1C(OC)(C=2OC34CCCC(=C)C3(CCC(C)C4(CC=2C1=O)C)C)CC(OC)=O	5.9	Good	6.3
8750	O1C(O)C2C(=CCC3C2(C)C(OC(=O)C)CC2C3(CCC3C(CCCC23C)(C)C)C)C1=O	4.8	Poor	6.3
7849	O1C2C(CCC(=CC(O)CC(=CC(OC(=O)C)CC(=C2)C)C)C)C(=C)C1=O	5.2	Poor	6.3
6015	O(C(=O)C)C1CC2C(CCCC2(C2CCC(C(OC)=O)C(O)C12C)C)(C)C	5.3	Good	6.3
6000	O1C(C)C(O)C=CC(OC(CC=CC(OC(CC1=O)C)=O)C)=O	4.0	Excellent	6.3
5155	OC1CCC2(C(CCC3(C2CC(=O)C3=C(C=CC=C(C=CC=O)C)C)C)C1(C(O)=O)C)C	5.1	Bad	6.3
5055	O1C(CC2C3(C(CCC12C)C1(C(CC3)C(CCC1)(C)C)C)C)C1=CC(OC1O)=O	3.7	Bad	6.3
4750	O(C(=O)C)C1CC2C3C(CCC2(C)C1C=C)C1(C(CC(=O)C=C1)CC3)C	4.1	OK	6.3
2981	O=C1N(CC2=C1C1(C(CC2)C2(C(CC1)C(CCC2)(C(O)=O)C)C)C)CCc1ccccc1	5.4	Excellent	6.3
2860	O1C2C(CC(OC(=O)C)C(CCC(=CCCC(=C2)C)C)C(=O)C)C(CN(C)C)C1=O	6.2	Excellent	6.3
1851	O1C23CC(O)CCC2(C2C(C4CCC(C(CCC(C(C)C)=C)C)C4(CC2)C)CC13)CO	5.0	OK	6.3
1630	O(C)C1=CC(O)(C(OC)=O)C(CC2(C3CCCC(=C)C3(CCC2C)C)C)C1=O	5.0	Good	6.3
433	S1CC(NC1=O)C1(OC2CCC(C=CCCC(=CC(OC(C1)C2)=O)C)C)OC	6.8	OK	6.3
243	O1C(C2C(C(=CC(C(=O)CC)C)C)C(=CC(C)=C2C)C)=C(C)C(=O)C(C)=C1OC	5.8	OK	6.3
240	O1C(CC(CCCC(=CC=CC2(CCCc3c2occ3)C)C)=C)C(O)=C(C)C1=O	4.7	Bad	6.3
13467	O1CC23C(CCC4(C5Cc6cc(O)ccc6OC5(CCC24)C)C)C(CCC3)(C)C1O	4.6	Bad	6.3
13200	O(C(=O)C)C1CC2=CCC3C4CCC(=O)C4(CCC3C2(CC1)C)C	4.7	Poor	6.3
12199	O=C(C)C1CCC2C3C(CCC12C)C1(C(CC(=NO)CC1)CC3)C	3.7	Bad	6.3

11795	O=C1N2C(CCC2)C(=O)NC1Cc1c2c([nH]c1)cccc2	4.4	Excellent	6.3
11383	Brc1cc(N2C=Cc3c([nH]c4c3cccc4)C2=O)c(cc1)C(OC)=O	5.0	OK	6.3
11332	O1C(CC=C(CCC=C(CCC=2C(CCCC=2C)(C)C)C)C1OC)C1=CC(OC1O)=O	3.7	Poor	6.3
11174	O1CC2C(C(C(C(OC)=O)C)=C(CC2)C2(CC(CCC2)(C)C)C)C1=O	5.6	Excellent	6.3
10800	OC1CC2CCC3C4CCC(C(CCC(OC)=O)C)C4(CCC3C2(CC1)C)C	4.8	Poor	6.3
10675	O(C(=O)C)C1C2C(CCC2C(O)(C)C)(C=CC(CCC=C(C1)C)C)C	4.5	Excellent	6.3
10558	O1C23C1C(O)C1C4CCC(C(CCCC(C)C)C)C4(CCC1C2(CCC(O)C3)C)C	5.7	OK	6.3
9120	O(C)c1cc(CC2(C3CCC(=O)C(CCC(O)=O)C3(CCC2C)C)C)c(O)c(c1)C	4.8	Excellent	6.3
8619	Oc1ccc(O)cc1CC1C2(C(CCC1=C)C(CCC2)(C)C)C	5.3	Excellent	6.3
8185	O(C(=O)C)C1CC2=CCC3C4CCC(C(CC(O)=O)C)C4(CCC3C2(CC1)C)C	5.3	Bad	6.3
7180	Brc1c(C)c(Br)c2C3C(CCC3C(C)C)(C)C(=O)C(=O)c2c1O	6.0	Excellent	6.3
6769	O1CCCCC1n1c2ncnc(NCc3ccccc3)c2nc1	5.3	Good	6.3
6631	O1C(C2=C(C3(C(CC2)C2(C(CC3=O)C3(C(CC2)C(CCC3)(CC)C)C)C)C)C1=O)C	4.1	Poor	6.3
6453	O(C(=O)C(=CC(CCCC)C)C)C1C2=CC(=O)C(CC2(C)C(CC1)C(O)=O)C(C=O)=C	4.4	Bad	6.3
6451	O(C(=O)C(=CC(CCCC)C)C)C1C2=CC(=O)C(CC2(C)C(CC1)C(O)=O)C(C=O)=C	4.8	OK	6.3
6132	O1OC(CCC1(CCC=C(CCC1(O)C(CCCC1=C)(C)C)C)C)C(C(O)=O)C	5.5	Bad	6.3
5459	O(C(=O)C)C1CC2=CCC3C4CCC(=O)C4(CCC3C2(CC1)C)C	4.6	Good	6.3
5412	OC12C=C3CC(O)CC3C(C=C(C=CC1CCCCCCC)C)C2=O	4.1	Good	6.3
4640	O=C(C1=NCCc2c1[nH]c1c2cccc1)c1n(cnc1)C	4.9	OK	6.3
4528	O(C(=O)C)c1c2c(cc3c1c(OC(=O)C)ccc3)cccc2OC(=O)C	5.1	Excellent	6.3
3795	ClC1(CCC=C(CCC=C(CC2OC(=O)C(C2CC1O)=C)C)C)C	5.2	Excellent	6.3
3679	O1CC(CC(OC)=O)C(C2(C3C(CC2)C(CCCC3=C)(C)C)C)C1=O	5.1	Excellent	6.3
2119	O1CC2(C(CCC3(C2CCc2c3coc2)CO)C(CO)(C)C1=O)C	6.0	Poor	6.3
1798	O1C2(CC(=O)C=CC2=O)CC2C3(C(C45C(CC3)C(CCC4)(C)C(OC5)=O)CCC12C)C	4.0	Bad	6.3
1513	O(C)c1ccc(cc1O)CC=1N(C)C(=N)N(C)C=1Cc1ccc(OC)cc1	5.0	Excellent	6.3
14300	O(C(COC(=O)C1C2(C(CC=C1C)C1(C(CC2)C(CCC1)(C)C)C)C)CO)C(=O)C	5.3	Good	6.3
14156	O1C=C(C2C(C(CCC=C(CC2)C)=C)C1OC(=O)C)C(O)CC=C(CO)C	4.7	Excellent	6.3
14035	O1C(CCC1C(C)=C)(C)C1C2C(CCC(O)(C)C2[NH+]=[CH-])C([NH+]=[CH-])(CC1)C	4.2	OK	6.3
13793	O1C(=CC(NCCO)=C(C=O)C1=O)C1C2C(CCCC2)C=CC1C	7.6	Bad	6.3
13488	O1C2CCC(O)(C)C(CCC3C(C4C(CC5OC35C)C(O)(CC4)C)(C)C)C2(CCC(=O)C1(C)C)C	5.0	Excellent	6.3
12879	ClCC(O)(C)C1C2OC(CC1)(C)C(OC(=O)C)CCC(=CCCC(=C2)C)C	5.2	Excellent	6.3
12301	OC1CC(C2C3C(CCC3C)C12C)C(CC=CC(O)(C)C)=C	5.5	OK	6.3
11679	O(C(=O)C)C1C2C(CCCC2(C2CCC(O)(CC2(C1)C)C1CC(=O)C(=CC1)C)C)(C)C	4.4	Good	6.3
10908	O1c2c(cc(O)cc2)CC2C3(C(CCC12C)C1(C(CC3)C(CCC1)(C)C)C=O)C	5.7	OK	6.3
10491	OC1C2CC(O)CCC2(C=2C(C3CCC(C(CC(=O)C)C)C3(CC=2)C)C1)C	3.9	Poor	6.3
9626	o1cc(cc1CC(=CCCC(=CC(OCc1ccoc1)=O)C)C)C	5.5	OK	6.3
9196	OC1(CCC2C3C(C)(C(=O)CC2(C=C1)C)C(=O)C=C3C(C)C)C	5.7	Excellent	6.3
9017	O1C(O)C(=CC1=O)C(O)CC=C(CCC=C(CCC1C(CCC=C1C)(C)C)C)C=O	4.9	Bad	6.3
8164	O1C(CC=C(CCC2(C3CCCC(=C)C3(CCC2C)C)C)C)C(=CC1=O)CO	4.6	Poor	6.3
8010	O1CC12C1(C3(OC3C2O)C(O)C2C1C(OC(=O)CCCCCCC)C(C2)(C)C)C			6.3
6803	OC(=O)c1c2nc3c(nc2ccc1)c(ccc3)C(O)=O	5.2	Excellent	6.3
6462	Oc1cc(c2Nc3c(-c4c2c1ncc4)cccc3)C=O	5.7	Excellent	6.3
5878	O(C(=O)C)c1c2c(ccc1)C(=O)c1c(C2=O)c(OC(=O)C)ccc1	4.8	Excellent	6.3
5778	O1C(CC=CCC=CCCCCCCCC1=O)C(O)CC	4.0	Excellent	6.3
5776	O1C(CC)C(O)CC=CCC=CCCCCCCCC1=O	4.5	Good	6.3

5194	O1C2C=C(C1CC(=CCCC(=CCCC2(O)C)C)C)C(O)(C)C	5.0	Good	6.3
5125	S=C=NC1(C2C3C(CC1)C(CC1CC(C)C(C(C13)CC2)(C#N)C)C)C	4.0	Excellent	6.3
4715	ClC1C(C2CC=C3C(C2(CC1O)C)C(OC3)O)(CCC(C(C)=C)C)C	5.0	Excellent	6.3
4699	O1C2C(CC3OC3(CCC(O)C(CCCC(C)C2O)=C)C)C(=C)C1=O	5.4	Poor	6.3
4614	O1C2C1(CCC=C(CCC1(C)C(O)(C(=CCC1C)C2=O)C)C)C	5.1	Poor	6.3
3601	OC1C2C(CC=C2C)C(=C)C(O)CC1C(C(OC(=O)C)C(OC(=O)C)C=C(C)C)C	4.7	OK	6.3
2350	O1C2C(c3c(cc(OC)c(c3)C)C(C)C2O)=C(C)C1=O	6.0	Excellent	6.3
2016	O1CC(CC(OC)=O)C(C(=C)C2CCC3C2(CCCC3(C)C)C)C1=O	5.7	Excellent	6.3
1854	O(C)C1C=C2C(CCC3(C2CCC3C(C=CC(C(C)C)C)C)C)C2(CCC(O)CC12O)C	5.2	OK	6.3
1288	O1C(O)C2=C(C3C(C3)C(CC(OC(=O)C)C2C(CCC=C(C)C)C)C)C1O	5.1	Poor	6.3
14436	O1C(C2(C=C(C3OC3(C)C2C(=CC(C(=O)CC)C)C)C)C)=C(C)C(=O)C(C)=C1OC	5.2	Excellent	6.3
14347	O(C(=O)C)C1CC2C(C3CC=C(CC13C)C=O)(CCC1C(CCCC12C)(C)C)C	5.7	Bad	6.3
13220	O1C(O)C2=C(C3C(C3)C(CC(O)C2C(CCC=C(C)C)C)C)C1=O	4.9	OK	6.3
13195	O1C2CCC3(C1)C(C=O)C(CCC23C)C(=CC=CC(O)(C)C)C=O	5.7	Poor	6.3
13122	O1CC23C(CCC2C2C(CC3)C3(C(=CC(=O)C=C3)CC2)C)C(C)C1(O)CCC(C)C	4.3	OK	6.3
12839	O1C(O)C(=CC1=O)C=CC=C(CCC1(C2CCCC(=C)C2(CCC1C)C)C)C	3.8	Bad	6.3
12447	O(C)c1c(OC)c2c(cc1OC)C(=O)c1c(C2=O)c(O)cc(c1)C	5.5	Good	6.3
11866	O(C(=O)c1c2nc3c(nc2ccc1)c(ccc3)C(O)C)C	4.7	Excellent	6.3
11808	O1C(OC)c2c(ccc(C3(CC(CCC3)(C)C)C)c2C(C(O)=O)C)C1OC	5.7	Good	6.3
11703	O(C(=O)C)C1CC2C(C3CC=C(C=O)C(C=O)C13C)(CCC1C(CCCC12C)(C)C)C	4.1	Poor	6.3
8580	OC1CC2C3C(CCC2(C)C1C(O)(CCCC(C)C)C)C1(C(=CC(=O)CC1)CC3)C	4.7	OK	6.3
6600	O1C2(O)C3CC(CCC2=CC1=O)(C)C(=C)C(C3)C	4.5	Excellent	6.3
6311	S(CCN(C)C)C=1C=C(O)C2=NCCc3c2c(nc2c3cc(O)cc2)C=1	3.8	Excellent	6.3
5760	o1c2c(CCCC2(CC(=O)CC(=CCCc2ccoc2)C)C)cc1	4.6	Excellent	6.3
5097	O=C1N2C(CC3(c4c(NC23)cccc4)C(C=C)(C)C)C(=O)NC1=Cc1n(cnc1)CC	4.3	Poor	6.3
4815	O1C2(CCC=C(CCC(CC=C(CCC2O)C)C(C)=C)C1=O)C	5.0	OK	6.3
4375	o1c(ccc1COC)-c1nc(cc2c1[nH]c1c2cccc1)C(O)=O	6.8	Good	6.3
4087	Clc1c([nH]cc1Cl)-c1oc(-c2ccc(O)cc2)c(Cl)n1	4.9	Poor	6.3
3652	OC1CCC2C3C(CCC12C)C1(C(=CC(=NO)CC1)CC3)C	4.2	Poor	6.3
3597	O1C2C3C(C1CC(=C)C(O)CCC2(O)C)C(CCC3C(C)C)=C	4.8	Excellent	6.3
2995	O1C(CC=C(CCC=C(CCC=2C(CCCC=2C)(C)C)C)CO)C(CC1=O)CO	4.6	Bad	6.3
2379	O1C(CCC1C(O)(C)C)(C)C1CCC(C2C(C1O)C(=CC2)C)=C	4.3	OK	6.3
1936	o1cc(c2CC3C(CCCC(=Cc12)C)(C)C(=O)C=CC3(O)C)C	5.2	OK	6.3
1263	O1C(OC(=O)C)C2C(CCC(C(C)=C3CCCC3(C)C)C2(C)C)C1OC(=O)C	5.1	OK	6.3
752	o1cc2c(CCC3C4(C(CCC23C)C(CO)(C)C(=O)C(O)C4)C)c1	4.7	OK	6.3
667	ClC1CCC(OC1(C)C)(C)C1C2C(CCC(O)(C)C2[NH+]=[CH-])C(N=C=S)(CC1)C	4.8	Excellent	6.3
13206	o1cc(cc1)CCC=C(CC=CC(O)(CC=Cc1ccoc1)C)C	5.1	Excellent	6.3
13117	O1C2(O)C(C3CCC(C(C=CC(C(C)C)CC)C)C3(CC2)C)=CC1=O	4.3	Bad	6.3
12437	O1C=CC(=C)C1OC(=O)C=C(CCC=C(Cc1occ(c1)C)C)C	3.9	Poor	6.3
12314	OC1CC(C2C3C(CCC3C)C12C)C(CCC(O)C(C)=C)=C	4.8	Good	6.3
12284	O(C(=O)C)C1CC2=CCC3C4CCC(C(O)C)C4(CCC3C2(CC1)C)C	4.4	Poor	6.3
11052	O1C2=C(CC1C(O)(C)C)C(=O)c1c(cccc1)C2=O	4.7	Poor	6.3
10136	O=C(c1nccc2c1[nH]c1c2cccc1)c1[nH]cnc1	4.6	Excellent	6.3
9478	O(C)c1cc(C)c(CCC(=CCC2=CC(=O)C=CC2=O)C)c(C)c1C	4.5	Poor	6.3
8992	O1C23CC(O)CCC2(C)C(=O)C(CC13)C1CCC(C(CCC(C(C)C)C)C)C1(CCO)C	4.6	Good	6.3
8538	O1C(=O)C(=C2CC3C(CC12O)(C=CC=C3COC(=O)C)C)C	5.7	Excellent	6.3
8097	O(C(=O)C)C1CC2=CCC3C4CCC(C(=O)C)C4(CCC3C2(CC1)C)C	4.1	Excellent	6.3
8095	O(C(=O)C)C1CC2=CCC3C4CCC(C(=O)C)C4(CCC3C2(CC1)C)C	4.2	Bad	6.3
6618	O1C2C=C(C(C)C)C(=O)C(O)C(CCC(=O)C(=CCCC12C)CO)C	5.2	Excellent	6.3
6567	OC1(CCC2C(CCC3C(CCCC23C)(C)C)(C)C1C(OCC(OC(=O)C)CO)=O)C	4.7	Poor	6.3
6476	OC1(CCC2C(C1[NH+]=[CH-])C(CCC2([NH+]=[CH-])C)C(CCC=C(C)C)C)C	4.9	Excellent	6.3

6247	O1C(CC2C3(C(CCC12COC(=O)C)C1(C(CC3)C(CCC1)(C)C)C)C)C1=CC(OC1O)=O	4.5	Bad	6.3
5827	O=C1CC2C(C=C1CC(O)=O)CCC1C3(C(CCC12C)C(CCC3)(C)C)C	4.6	Bad	6.3
5797	OC1(C2C3C(CCC(O)(C2CC1)C)C3(CCC=C(C)C)C)C	5.8	Good	6.3
5491	O1C(O)C(CCC1C(C)C)C1(O)CCC2C3C(CCC12C)C1(C(=CC(=O)CC1)CC3)C	4.9	Poor	6.3
4703	O1C2(O)C(C3CCC(C(C=CC(C(C)C)C)C)C3(CC2)C)=CC1=O	4.0	Bad	6.3
4199	S=C=NC(C)(C)C1OC(CC1)(C)C1C2C(CCC(=C2)C)C(CC1)(C#N)C	4.9	Poor	6.3
4159	Oc1ccc(O)cc1CC=C(CCC1C(CC(=O)C=C1C)(C)C)C	5.0	Bad	6.3
2435	O1C(CNCC1c1c2c([nH]c1)cccc2)c1cc(OC)c(OC)c(OC)c1	3.6	Poor	6.3
2309	o1cc(c2-c(c1)c(cc2)C=O)CC(OC(=O)C)C=C(CCC=C(C)C)C	5.9	Poor	6.3
1524	O(C(=O)C)C1CC(C(=CCC=C(C)C)C)C(C2C(C=CC2=O)C)C1COC(=O)C	5.6	Bad	6.3
1173	O1C2CC(=CCCC(C(C2C(CCC=C(C)C)C)C1=O)C=O)C			6.3
1101	O(C(=O)C)C1CC(C(CC=CC(O)(C)C)C)C(O)C2C(CC=C2C)C1=C	5.8	Poor	6.3
13395	O1C2CC(O)C3C(CCC3C(O)(C)C)(C=CC(CCC12)C)C	5.2	Excellent	6.3
11222	O1C2CCC(C3C(CCC12C)C(C3)(C(=O)CCC(OC(=O)C)C)C)=C	4.8	Good	6.3
10330	O=C1N2NC(c3ccccc3)=C(C2=NC=C1C(OCC)=O)C	5.8	Good	6.3
6558	O(C)c1c(cc(O)cc1C)CC=C(CC(=O)C=C(CCCC(=O)C)C)C	5.5	Poor	6.3
6475	S=C=NC1(C2C(C(CC1)C1(OC(CC1)C(C)=C)C)C([NH+]=[CH-])C(O)(CC2)C)C	4.4	OK	6.3
6291	OC1CC2(C(CC=C1C)C(CC2)C(O)(CCC=C(C)C)C)C	5.1	OK	6.3
4380	O1OC(CC1(CC(CCCCCCc1ccccc1)C)C)(CC(O)=O)C	5.7	Excellent	6.3
1947	O1C(=O)C(=C2CC3(C(CC12O)C(OC(=O)C)CCC3C)C)C	4.5	Excellent	6.3
1799	O1Cc2c(C=C1C(CC(CC)C)C)cc1c(C(=O)C(=O)C(OC)=C1)c2O	5.1	Good	6.3
1796	O1c2c(cc(O)cc2)CC2C3(C(CCC12C)C1(C(CC3)C(CCC1)(C=O)C)C)C	4.5	Poor	6.3
1594	S1CC(NC1=O)C1(OC(CCC1)CCC(C=CCCC(=CC(OC)=O)C)C)O	4.7	OK	6.3
858	[nH]1c(ccc1C=C1N=C(C)C(=C1)CCCCC)-c1[nH]ccc1	6.1	Good	6.3
582	O1c2c(OC1)cc1c(C(=O)c3c(ccc(O)c3OC)C1=O)c2OC	4.7	Excellent	6.3
14435	O1C(C2(C=C(C)C(=O)C(C)=C2C(=CC(C(=O)CC)C)C)C)=C(C)C(=O)C(C)=C1OC	5.4	Good	6.2
13547	OC12CC(=O)CCC1(C1CCC3(C(CCC3C(C=CC(C(C)C)CC)C)C1(O)C=C2)C)C	4.1	Poor	6.2
13187	O1C2C(CC3OC3(CCC=C(CCC=C(C)C2O)C)C)C(=C)C1=O	5.0	Excellent	6.2
12998	O1C2CCC(=CCC(CCC(=CC(O)CC12C)C)C(O)(C)C)C	3.7	Good	6.2
12628	O1C(O)C(=CC1=O)C(O)CC=C(CCC=C(CCC=1C(CCCC=1C)(C)C)C)C=O	4.7	OK	6.2
9016	O1C(CC=C(CCC=C(CCC2C(CCC=C2C)(C)C)C)C1OC)C1=CC(OC1O)=O	4.1	Bad	6.2
8791	O1CC(=CC1=O)C(O)CC=C(CCC1(C2CCCC(=C)C2(CCC1C)C)C)C	5.1	Excellent	6.2
8591	O1CC2=C(C3(C(CC2)C2(C(CC3O)C3(C(CC2)C(C)(C)C(OC(=O)C)CC3)C)C)C)C1=O	4.7	Poor	6.2
8410	O1C(=CC(CCC=C(CCCc2cc(oc2)Cc2ccoc2)C)C)C(O)=C(C)C1=O	5.4	Poor	6.2
8302	O1C(=CC(CCC=C(CCCc2cc(oc2)Cc2ccoc2)C)C)C(O)=C(C)C1=O	5.2	Poor	6.2
7576	O=C(C(C)(c1c2c([nH]c1)cccc2)c1c2c([nH]c1)cccc2)C	4.4	Excellent	6.2
7508	OC12CC(O)CCC1(C1C(C3CCC(C(CCC=C(COC(=O)C)C)C)C3(CC1)C)CC2=O)C	3.8	Bad	6.2
7227	O=C1CC2C3C(CCC2(C)C1C(O)(CCCC(C)C)C)C1(C(CC(=O)C=C1)CC3)C	4.5	Good	6.2
6469	O(C(=O)C)C1C2C(CCC2C(OC(=O)C)(C)C)(C=CC(CC(=O)CC(=C1)C)C)C	5.0	OK	6.2
5985	O1C(C)C(O)C=CC(OC(CC=CC(OC(CC1=O)C)=O)C)=O	4.8	Excellent	6.2
5688	O=C(NC1(C2CC(C3CC2(CC1C3)C)C(C)C)C)NCCc1ccccc1	4.8	Bad	6.2
5558	O(C(=O)C)C1CC2C(C3CC=C(C=O)C(CO)C13C)(CCC1C(CCCC12C)(C)C)C	4.6	Excellent	6.2
3447	OC1(CCC(=CC=C(CCC(=O)C(CCC1O)C)C)C(C)C)C	5.2	Good	6.2
1745	S(OC1CC2=CCC3C4CCC(C(=O)C)C4(CCC3C2(CC1)C)C)(O)(=O)=O	4.2	Excellent	6.2
1632	O(C)C1=CC(O)(C(OC)=O)C(CC2(C3CCCC(=C)C3(CCC2C)C)C)C1=O	5.5	Good	6.2
1623	O(C)C1=CC(O)(C(OC)=O)C(CC2(C3CCCC(=C)C3(CCC2C)C)C)C1=O	4.8	Excellent	6.2
1278	O1C2C=C(CCC(CC(=O)C(=CC(=O)C2=C(C)C)C)C(C)=C)C1=O	4.6	Good	6.2
14448	O1C2C=C(CCC(C(C)=C)C(O)C(=O)C(C)=C(O)C(=O)C2C(C)=C)C1=O	4.0	Excellent	6.2

14432	O1C(C2(C3C2(C=C(C)C3C(C(=O)CC)=C)C)C)=C(C)C(=O)C(C)=C1OC	6.3	OK	6.2
12631	O1C(CC=C(CCC=C(CCC=2C(CCCC=2C)(C)C)C)CO)C(=CC1=O)CO	4.1	Poor	6.2
12134	FC1=CN(Cc2ccccc2)C(=O)N(Cc2ccccc2)C1=O	6.4	Excellent	6.2
11942	O1CC(C2CC(C)C(OC(=O)CCC)C3C2C2OC3CC(=C)C(=O)CC(OC(=O)C)C12C)C	3.7	Poor	6.2
10414	O1C2CC(=CC(=O)C(OCC)CC(=O)CC(CC=C(C2O)C1=O)C(C)=C)C	5.5	Excellent	6.2
9159	ClC1CC2(C(CCC(=C)C2CC(O)C2CC(=O)NC2=O)C(C1)(COC(=O)C)C)C	5.1	Bad	6.2
9024	O1C(CC(=CCCC(C=CC=C(CCCC2=CC(OC2=O)OC)C)C)C)C(O)=C(C)C1=O	4.5	Bad	6.2
8977	O1C2CCC(=C)C(O)CCC(O)(C=CC(CCC12C)C(C)C)C	5.3	Excellent	6.2
8436	Oc1ccc(cc1)-c1nc(Cc2ccccc2)c(nc1)N	4.8	Poor	6.2
8188	O1c2c(C=CC1(CCC1(CCCC(C)C1=C)C)C)c(O)c(O)cc2OC	4.5	Excellent	6.2
8177	O(C)C1=CC(O)(C(OC)=O)C(CC2(CCC3(C(CCC=C3C)C2C)C)C)C1=O	4.7	Good	6.2
8176	O(C)C1=CC(O)(C(OC)=O)C(CC2(CCC3(C(CCC=C3C)C2C)C)C)C1=O	4.2	Good	6.2
7998	O(C(=O)C)C1CC2=CCC3C4CCC(=O)C4(CCC3C2(CC1)C)C	4.6	Poor	6.2
7933	O1C(=O)C(=C2CC3C(C4OC3(CC4)C)(CCC=C(CC12OC)C)C)C	5.5	Excellent	6.2
7490	O(C(=O)C)C1CCC2(C(CCC3(C2CC(=O)C3=C(C=CC=C(C=O)C)C)C)C1(C(O)= O)C)C	5.8	Bad	6.2
7481	O1C(O)C(OC)=C(C(=O)CC2(CCC3(C(CCC=C3C)C2C)C)C)C1=O	4.6	Good	6.2
6551	O1CC2=C(CCC3C4(C(CCC23C)C(CCC4)(C(O)=O)C)C)C1=O	4.4	Good	6.2
6454	O(C(=O)C(=CC(CCCC)C)C)C1C2=CC(=O)C(CC2(C)C(CC1)C(O)=O)C(C=O)= C	5.0	Good	6.2
6090	s1c2c(nc1)c1nccc3-c4c(Nc(c13)c2CCN)cccc4	3.9	OK	6.2
5492	O1C(O)C(CCC1C(C)C)C1(O)CCC2C3C(CCC12C)C1(C(CC(=O)CC1)CC3)C	3.8	Bad	6.2
5267	O(C)c1cc(O)cc(C=C2C3(C(CCC2C)C(CCC3)(C)C)C)c1O	3.8	Bad	6.2
4918	O1C2C(CC3C(C)(C(OC(=O)CCC)CCC(=C2)C)C(OC(=O)C)CC=C3C)=C(C)C1= O	5.0	Poor	6.2
4842	O(C)c1ccc(O)c(O)c1C=C1C2(C(CCC1C)C(CCC2)(C)C)C	4.4	Good	6.2
4756	O1C=C(C2C(C(CC=CC(OC(=O)C)(CC2)C)=C)C1OC(=O)C)C(=O)C=CC(O)(C) C	5.1	Poor	6.2
4551	O(C)c1ccc(O)c(O)c1C=C1C2(C(CCC1C)C(CCC2)(C)C)C	4.3	OK	6.2
2936	O1C2(O)CCC3C4CCC(C(CCCC(C)C)C)C4(CCC3C2(CC1=O)C)C	5.3	Poor	6.2
2791	O(C(=O)C)C1CCC(=CC2C(C(O)(CCC2C(C)C)C)C(OC(=O)C)CC1=C)C	4.6	Good	6.2
652	Oc1c2c(cc(C)c1C(=O)C)cc1c(C(=O)C(C)(C)C(=O)C1(C)C)c2O	3.4	Bad	6.2
347	O1C2C(CCC(=CCC(OC(=O)C)C(=CCCC(=C2)C)C)C)C(=C)C1=O	5.0	OK	6.2
14127	O1C2CC3OC(CC4=CCC(C(C)C)C4(CC(=O)C3(O)C)C)C12C	4.1	Excellent	6.2
13891	OC1CC2=CCC3C4CCC(C(O)C)C4(CCC3C2(CC1)C)C	4.5	OK	6.2
13406	O1C2C3C(C(CCC3C(CO)C)=C)C(=O)CC(O)(C1CCC2(OC(=O)CCC)C)C	6.0	Excellent	6.2
12774	O(C(=O)C)C1CC(C(=CCC=C(C)C)C)C(C2C(C)C(OC(=O)C)CC2=O)C1CO	5.3	Good	6.2
11588	o1cc2c(CCC3C4(C(CCC23C)C(CO)(C)C(O)C(=O)C4)C)c1	4.9	Excellent	6.2
11165	O=C1C(C(C(O)=O)C)=C(CCC1COC=O)C1(CC(CCC1)(C)C)C	4.9	Excellent	6.2
10392	O(C(=O)C)C1C2C(CC=C2C)C(CCC1C(CCC(=O)C(C)C)C)=C	4.6	Good	6.2
9266	O(C(=O)C)C1CC2C(C3CCC(C(CC(O)C(C(C)C)C)C)C13C)CC=C1CC(O)CCC1 2C	4.5	Bad	6.2
9222	O1C2=C(CC3(CCC4(C(CCCC4=C)C13C)C)C)C(=O)C(OC)=CC2=O	5.1	Good	6.2
9199	OC1C=C(C2C3CCC(O)(C=CC3(CC(=O)C12C)C)C)C(C)C	5.9	Good	6.2
7721	O1CC2=C(C3(C(CC2O)C2(C(CC3O)C3(C(CC2)C(CCC3)(C)C)C)C)C)C1=O	5.6	Good	6.2
7059	O(C(COC(=O)C)CO)C(=O)CC(CCC1(C=2C(CCC1C)C(CCC=2)(C)C)C)C	4.4	Excellent	6.2
6693	O1C2C(CC3C(C)(C(OC(=O)C=C(C)C)CCC(=C2)C)C(OC(=O)C)CC=C3C)=C(C)C1=O	5.0	Good	6.2
6042	O1C2(C(=C(C(=O)CCCCCC(O)C)C1=O)C(=C1C(=COC(=C1)C)C2O)C=O)C	6.4	Bad	6.2
4758	O1C=C(C2C(C(CCC=C(CC2)C)=C)C1OC(=O)C)C(O)CC=C(C)C	5.1	OK	6.2
4420	O(C(=O)C)C1C2C(C=CC=C2C)(C)C(=O)CC2C1(CCC2(O)C(C)C)C	4.7	Good	6.2

3696	O(C(COC(=O)C1C2(C(CC=C1C)C1(C(CC2)C(CCC1)(C)C)C)C)CO)C(=O)C	4.2	Bad	6.2
3339	O1C(=O)C(CC1C=C(C)C)=CCCC(=CCCC(=CCC(O)C1=CC(OC1)=O)C)C	5.1	Bad	6.2
2399	ClC1CCC(OC1(C)C)(C)C1C2C(CCC(O)(C)C2NC=O)C(N=C=S)(CC1)C	4.3	Good	6.2
945	O(C)C1=CC(=O)C(O)=C(CC2(C3CCCC(C)(C)C3(O)CCC2C)C)C1=O	5.3	Excellent	6.2
14441	OC1C2C3C(CCC2(CC(=O)C(=C1)COC(=O)C)C)(CCC3C(C)=C)C	6.3	Excellent	6.2
13911	O1OC(CCC1(CCC=1C(CCCC=1C)(C)C)C)C(C(OC)=O)C	4.8	Excellent	6.2
12878	O1C2C=C(CCC=C(CCC(O)C1(CCC2C1(OC1)C)C)C)C	4.9	Excellent	6.2
12772	O(C(=O)C)C1CC(=O)C(C2C(C=O)C(O)CC2C(=CCC=C(C)C)C)C1C	5.0	Good	6.2
11781	OC1C2CC(O)CCC2(C=2C(C3CCC(=C(CC(=O)CC(C)C)C)C3(CC=2)C)C1)C	3.8	Bad	6.2
11593	n1c2c(n(C)c1N)C1=NC(=NC1=CC=C2C)NC	5.9	Excellent	6.2
10799	O(C(=O)C)C1CC2=CCC3C4CCC(C(CCC(OC)=O)C)C4(CCC3C2(CC1)C)C	3.7	Bad	6.2
10462	O(C(=O)C)C1CC2C(CCC3(C2CCC3C(CCCC(C)C)C)C)C2(CCC(O)CC12O)C	5.4	OK	6.2
9630	O(C(=O)C)C1CC2C(C3CC=C(C=O)C(C=O)C13C)(CCC1C(CCCC12C)(C)C)C	5.5	Bad	6.2
9166	O1c2c(cc(OC)c(O)c2)C(OC)C2C3(C(CCC12C)C(CCC3)(C)C)C	5.6	Bad	6.2
8293	o1cc(cc1)CCCC(=CC(=O)CC(=CCCc1ccoc1)C)C	4.8	Good	6.2
8011	O1CC12C1(C3(OC3C2=O)C(O)C2C1C(OC(=O)C(O)CCCCCC)C(C2)(C)C)C			6.2
7590	O=C1N2C(=CCC2)C(=O)NC1Cc1c2c([nH]c1)cccc2	4.6	Excellent	6.2
7458	O=C1CC23C(CCC1(C2)C)C1(C(CC3)C(CCC1)(C(O)=O)C)C	5.5	Good	6.2
7295	O(C(=O)C)C1CC2C(C3(C)C1(O)CC=CC3=O)C(O)CC1(C2CCC1C(CCC(C(C)C)C)C)C	6.3	Good	6.2
6135	O1C(O)C(=CC1=O)C(O)CC1(C=2C(CCC1C)C(CCC=2)(CCCC(C)=C)C)C	4.3	OK	6.2
6129	O1OC(CCC1(CCC1C(CCCC1=C)(C)C)C)C(C(O)=O)C	4.8	OK	6.2
5096	O(C(=O)c1[nH]ccc1)CC1=CCCC2C(CCC(O)(C=C)C)(C)C(CCC12C)C	4.0	Bad	6.2
4841	O(C)c1ccc(O)c(O)c1CC=1C2(C(CCC=1C)C(CCC2)(C)C)C	4.9	OK	6.2
3964	O1C(C2=C(C3(C(CC2)C2(C(CC3O)C3(C(CC2)C(CCC3)(CC)C)C)C)C)C1=O)C	5.2	Good	6.2
3906	O1C2(CCC1(C=CC(O)(CCC=C(CCC2O)C)C)C(C)C)C	5.8	Excellent	6.2
3719	OC(=O)C(CC1C(CCC2C1(CCC1C(CCCC12C)(C)C)C)(C=O)C)C	5.5	Good	6.2
3651	OC1CCC2C3C(CCC12C)C1(C(=CC(=NO)CC1)CC3)C	5.0	OK	6.2
3206	O1C2C1(CCC=C(CCC1(C)C(C(=CCC1C)C2=O)C=O)C)C	5.1	Excellent	6.2
2376	O1CC(C2C(C(=CCC=C(CC2O)C)C=O)C1=O)CCC=C(C)C	6.2	Excellent	6.2
2241	O1C2(C34OOC(CC3)(C=C4CC(C)C2(CC1C1(OOC(CC1)C(C(O)=O)C)C)C)C) C	5.1	Good	6.2
566	O1C2CC(c3cc(O)c(cc3C2=C(C)C1=O)C)C	5.9	Excellent	6.2
17	O1C(C(C(=O)C(C)C=2OC(CC)=C(C)C(=O)C=2C)C)=C(C)C(=O)C(C)=C1CC(C) C	4.6	Bad	6.2
14160	BrC1CCC2(C(CC(O)C3C2=CCC(C3)(C(Br)CO)C)C1(C)C)C	5.0	OK	6.2
14081	N1C=2C(N=C1N)=C(C=CC1=NC(=NC1=2)N(C)C)C	4.7	Bad	6.2
13567	O=C1NC(N(C)C1CC(CC)C)C(=O)c1c2c([nH]c1)cccc2	5.5	Poor	6.2
13116	O1C2(O)C(C3CCC(C(C=CC(C(C)C)C)C)C3(CC2)C)=CC1=O	4.6	Excellent	6.2
12002	O(C(=O)C)C1CC2C(C3CCC(C(CCC(=O)C(C)C)C)C13C)CCC1=CC(=O)CCC12 C	4.8	Bad	6.2
11958	O1C2CCC(C3C(CCC12C)C(C3)(CCC(OC(=O)C)C(O)(C)C)C)=C	4.8	Excellent	6.2
11077	O1C2C3C(C1CC(=CCCC2(O)C)C)C(O)(CCC3C(C)C)C	4.4	Excellent	6.2
8640	O(C)C1CC2=CCC3C4CCC(O)(C(=O)C)C4(CCC3C2(CC1)C)C	3.9	Poor	6.2
8539	O1C(=O)C(=C2CC3C(CC12O)(C=CC=C3C)C)C	4.9	Good	6.2
8356	O(C(=O)C)C1CC2=CCC3C4CCC(C(CCC(OC)=O)C)C4(CCC3C2(CC1)C)C	3.7	Bad	6.2
6044	O1C2(C(C=C3C(=COC(=C3)C)C2O)=C(C(=O)CCCCCCC)C1=O)C	7.1	Excellent	6.2
5748	O1CC(=CC1=O)CCC=C(CCC(O)C(CCC1(C)C(CC(=O)C=C1C)C)=C)C	5.5	OK	6.2
5691	O(C)c1c(O)c(ccc1OC)Cc1n(C)c(nc1Cc1ccc(OC)cc1)N	4.5	OK	6.2
5501	O1C23CC(OC(=O)C)CCC2(C)C(=O)C(=CC13)C1CCC(C(C=CC(C(C)C)C)C)C1 (CC=O)C	5.1	Bad	6.2
5489	O1C(O)C(CCC1C(C)C)C1(O)CCC2C3C(CCC12C)C1(C(=CC(=O)C=C1)CC3)C	4.2	Bad	6.2

5387	O1C(CC(CCC(CC(C)C(=O)N(C=CCC1=O)C)C)C)C(C)(C)C	4.6	Excellent	6.2
4539	O1C2C3C(C1CC(O)(C)C(O)CCC2(OC(=O)CCC)C)C(=C)C(OC(=O)C)CC3C(C)C	4.6	Good	6.2
4367	O(C)c1c2c(cc(OC)c1)C(=O)c1c(C2=O)c(O)cc(O)c1C(=O)CCC	6.0	OK	6.2
4206	ClC1CC(OC(=O)C)C(OC(CC1OC(=O)C)C(OC(=O)C)CC)CC=CC#C	4.9	OK	6.2
3987	O1C(CC2C3(C(CCC12COC(=O)C)C1(C(CC3)C(CCC1)(C)C)C)C)C1=CC(OC1O)=O	5.8	OK	6.2
3935	O1C2C3C(C1CC(O)(C)C(OCC)CCC2(O)C)C(CCC3C(C)C)=C	4.6	Excellent	6.2
3408	OC1C2C(C=CC2(O)C)C(CCC1C(CC(OC(=O)C)C=C(C)C)C)=C	5.4	OK	6.2
2359	O1C(CCC(C)C(O)CC1=O)C(C(=O)C(=CCC(O)C(C)C)C)C	4.9	Poor	6.2
521	O(C(=O)C)C1C(=CC2(C(=CC(OC(=O)C)CC2C)CCC1=O)C)C	4.7	Good	6.2
14040	ClC1CCC(OC1(C)C)(C)C1C2C(CCC(O)(C)C2[NH+]=[CH-])C([NH+]=[CH-])(CC1)C	5.6	Good	6.2
13831	OC1C(C2CCC3(C(CC=C(C)C3C(OCCC)=O)C2(CC1O)C)C)(C)C	4.8	Excellent	6.2
13760	O1C2CCC(C3C(CCC12C)C(C3)(CCC(O)C(O)(C)C)C)=C	4.1	Poor	6.2
13404	O1C2C3C(C(OC(=O)CCC)(CCC3C(C)C)C)C1(O)CC(=C)C(OC(=O)C)CCC2(O)C	5.6	Good	6.2
12302	OC1CC(C2C3C(CCC3C)C12C)C(CC(O)C=C(C)C)=C	4.9	Excellent	6.2
11685	O1C2C=3C(CCC2C(=C)C1=O)(C)C(O)CCC=3C	5.3	Excellent	6.2
10682	O(C(=O)C)C1C2C(CCC2C(O)(C)C)(C=CC(CC(OC(=O)C)C=C(C1)C)C)C	4.0	Good	6.2
9496	O1C2OC(=O)CC12CCCC(CC(O)CC(=CCCc1ccoc1)C)C	4.4	Poor	6.2
9283	O1C23C1C(O)C=1C(CCC4(C=1CCC4C(CCCC(C)C)C)C)C2(CCC(O)C3)C	5.5	OK	6.2
8705	O1CC2C(=CCC3C2(C)C(O)CC2C3(CCC3C(CCCC23C)(C)C)C)C1=O	4.4	OK	6.2
8417	O1c2cc(C)c(O)cc2C=CC1(CCC=C(CC(=O)CC(C)C)C)C	4.2	Excellent	6.2
8065	O1C(=O)C(CC1C=C(CCCc1ccoc1)C)CCCc1ccoc1	4.6	Bad	6.2
7974	O(CC1=CCCC2C(CCC(O)(C=C)C)(C)C(CCC12C)C)C(=O)C	4.1	Bad	6.2
6814	O(C(=O)c1c2nc3c(nc2ccc1)c(ccc3)C(OC)=O)C	4.5	Good	6.2
6632	O1C(C2=C(C3(C(CC2)C2(C(CC3=O)C3(C(CC2)C(CCC3)(C)C)C)C)C)C1=O)C	3.7	Bad	6.2
6571	O1C(=CC(N)=C(C=O)C1=O)C1C2C(CCCC2)C=CC1C	4.7	Excellent	6.2
6559	O(C)c1c(cc(O)cc1C)CC=C(CC(=O)C=C(CCCC(=O)C)C)C	5.6	OK	6.2
5285	O1C(CCCCCC1=O)C1CC1C=CC(O)C(O)CC=CCCCCC	4.5	Poor	6.2
5180	O1C(C)(C)C1CC=C(C)C1C2C3C(=CCCC2CC1)COC3O	4.8	Excellent	6.2
4423	O1C(CCCC1=O)C1CC1C=CC(O)C(O)CC=CCC=CCC	3.4	OK	6.2
3506	OC1CC2C(C3CCC(C(CCC(OC)=O)C)C13C)C(O)CC1CC(=O)CCC12C	3.7	Excellent	6.2
3302	O=C1CCC2(C3C(C4CCC(C(CCC(O)=O)C)C4(CC3)C)CCC2=C1)C	5.2	Bad	6.2
2667	O1C(CCCCCC(CCCCc2ccc(O)cc2)C)(C)C(O)=C(C)C1=O	5.9	Bad	6.2
2377	OC1CC(=CCC=C(C=O)C(C=O)C1C(CCC=C(C)C)COC(=O)C)C	6.3	Good	6.2
1481	O1CC(C(OC(=O)C)C(OC)=O)C(C2(C3C(CC2)C(CCCC3=C)(C)C)C)C1OC(=O)C	4.6	OK	6.2
538	S(OC1CC2CCC3C4CCC(C(CCCC(C)C)C)C4(CCC3C2(CC1)C)C)(O)(=O)=O	3.8	Bad	6.2
499	O1C(=CC(OC)=C(C=O)C1=O)C1C2C(CCCC2)C=CC1C	5.8	Excellent	6.2
13646	O1C(O)(C(C(=O)C(CC(=CCC)C)C)C)C(C)C(O)C(C)C1C(C(=O)CC)C	3.6	Good	6.2
13000	O1C2=CC(O)(C3C(CC2=C(C)C1=O)C(CC3)=C)C	4.8	Excellent	6.2
12165	OC12CC3(C(CCC1(C)C(O)CCC2=C)=C(CC3)C(C)C)C	5.3	Excellent	6.2
10612	O1C23CC(CCC(=O)C(C)C)(C)C1(O)CCC2(C)C(=O)CCC3=C	5.2	Excellent	6.2
9839	BrC12OC1C1OC1(C)C1(CC(Br)C(Cl)(CC1O)C)C2(C)C			6.2
9310	O=C1C(=C(O)CCCCCCCCC(CC)C)C(=O)N(C)C1C	5.3	Bad	6.2
8995	Brc1cc2[nH]c3c(c2cc1)ccnc3C1ON(CC1)C	4.2	Excellent	6.2
8725	o1cc(cc1Cc1ccoc1)CCCC(=CCCC(CC(O)=O)C)C	4.9	OK	6.2
8515	O1CC23C(CCC1(C)C2C(O)Cc1ccoc1)C(CCC3)(C)C	4.9	OK	6.2
7090	O1CC(C2C(C(CCC=C(CC2)C)=C)C1=O)=CC=CC(CO)=C	5.6	Excellent	6.2
6504	O1C(=CC(OC)=C(CO)C1=O)C1C2C(CCCC2)C=CC1C	4.9	OK	6.2

5440	o1cc(cc1C=C(CCCc1cc(oc1)C(=O)NCCN)C)CCCc1ccoc1	4.3	Bad	6.2
3620	O=C1NCCCCC1NC(=O)C=C(CCC1(C)C(CCC=C1C)C)C	4.9	Bad	6.2
774	O1c2c(C(=O)C=C1CCC)c(O)cc1c2c(O)cc(OC)c1	6.0	OK	6.2
244	O=C1N=C(N=C1c1c2c([nH]c1)cccc2)N(C)C	4.8	Excellent	6.2
14489	O1C(CC(=CC=CC(=CCCc2cc(oc2)Cc2ccoc2)C)C)C(O)=C(C)C1=O	4.2	Bad	6.1
14033	O1C(CCC1C([NH+]=[CH-])(C)C)(C)C1C2C(CCC(O)(C)C2[NH+]=[CH-<br>])C([NH+]=[CH-])(CC1)C	4.7	Poor	6.1
13669	O1OC(CC1(CC(C=CCC(C=Cc1ccccc1)C)C)C)(CC(O)=O)C	5.1	Bad	6.1
12102	OC1CC(CCC(C)C(=O)CCC(CC(=O)CC1C)C)C(C)=C	5.8	Excellent	6.1
11456	O1C(C2(C3C2(C)C(=O)C(C)C3C(=CC(C(=O)CC)C)C)C)=C(C)C(=O)C(C)=C1O<br>C	5.6	Poor	6.1
11276	n1c2c(n(C)c1N)C1=NC(=NC1=CC=C2C)N(C)C	5.9	Excellent	6.1
10756	O1C2CC3(CC(O)C4(C(C3CCC12C)C(=CC4=O)C(C)C)C)C	6.2	Excellent	6.1
10652	O1C(CC(=CCCC(C=CC=C(CCCC2=CC(OC2=O)OC)C)C)C)C(O)=C(C)C1=O	4.8	Bad	6.1
8976	O1C2CCC(=C)C(O)CCC(O)(C=CC(CCC12C)C(C)C)C	5.8	Good	6.1
8845	OC1=C(C)C(=O)C2=C(C1=O)C(CCC2C(CCC=C(C)C)C)C	4.7	Excellent	6.1
8043	OC1CC2=CC(=O)C3C4CCC(C(=O)C)C4(CCC3C2(CC1)C)C	4.9	Excellent	6.1
6452	O(C(=O)C(=CC(CCCC)C)C)C1C2=CC(=O)C(CC2(C)C(CC1)C(O)=O)C(C=O)=<br>C	3.9	Bad	6.1
6113	O1C2CC(=CC(=O)CC(OCC)C(=O)CC(C(C)=C)C(=O)CC(=C2)C1=O)C	5.2	Good	6.1
5490	O1C(O)C(CCC1C(C)C)C1(O)CCC2C3C(CCC12C)C1(C(CC(=O)C=C1)CC3)C	4.3	Poor	6.1
4858	O1CC2C(CCC(O)(C=CCC2=C)C)C(=CC=CC(O)(C)C)C1=O	6.2	OK	6.1
4473	O=C(CNC(=O)C(N(C)C)Cc1ccccc1)c1c2c([nH]c1)cccc2	4.7	Excellent	6.1
14034	ClC(C)(C)C1OC(CC1)(C)C1C2C(CCC(O)(C)C2[NH+]=[CH-])C([NH+]=[CH-<br>])(CC1)C	5.1	Excellent	6.1
13892	OC1CC2=CCC3C4CCC(C(O)C)C4(CCC3C2(CC1)C)C	4.8	OK	6.1
13204	O1C2CC3C4(C(CCC3(C3CC=C(C(C23C)C1=O)C(O)C)C)C(CCC4)(C)C)CO	5.0	Bad	6.1
13022	OC1CC(=CCC=C(C=O)C(COC(=O)C)C1C(CCC=C(C)C)C)C	5.3	Poor	6.1
12151	O(C(=O)CC(O)C)C1CC2C(C3CC=C(C(=O)C)C(C=O)C13C)(CCC1C(CCCC12<br>C)(C)C)C	6.1	OK	6.1
12039	o1cc2c(CCC3C4(C(CCC23C)C(CCC4)(C(O)=O)C)C)c1	5.1	Excellent	6.1
11298	BrC1CCC(=C)C(C=CC(O)(C)C2CC(Br)C(O)(CC2)C)C1(C)C	4.8	Poor	6.1
11140	O1C(C(C)C2(O)CCC3C4C(CCC23C)C2(C(CC(O)CC2)=CC4)C)C1C(C(C)C)C	4.4	Bad	6.1
10213	OC1CC2=CCC3C4CCC(C(O)C)C4(CCC3C2(CC1)C)C	4.0	OK	6.1
8523	O1C2(O)C(=CC1=O)C(C1CC(O)C(=CC1C2)C)(C)C	5.1	Excellent	6.1
8411	O=C1C=C(C)C(=O)C=C1CC=C(CCC=C(CC(=O)CC(C)C)C)C	3.6	Bad	6.1
8327	O1C2CCC(=C)C(=O)CC3(C(=CCC12C)C(C(C)C)C(=O)C3)C	5.0	Excellent	6.1
7798	O1C(O)C(=CC1=O)CCC1(C2C3(CC3(CCC2)C)CCC1C)C	5.2	Excellent	6.1
6894	O1C2(OC)C(C3C(C(OC(=O)C)CC3C(=CCC=C(C)C)C)C1OC)C(C=C2)C	5.0	Excellent	6.1
4431	O(C(=O)C)C1C2C(C=CC=C2C)(C)C(=O)CC2C1(CCC2(O)C(C)C)C	4.7	Excellent	6.1
2578	O(C(=O)C)C1C2C(CCC3C1(CCC3(O)C(C)C)C)(C=CC=C2C)C	4.2	OK	6.1
1668	O=C1c2c(cccc2NC=O)C(C2C1=CC(CC2)(C=C)C)(C)C	3.7	Excellent	6.1
1649	O1C(O)(CC=C(C(O)CC=C(C)C)C)C2(C(CCC2)=C(C)C1=O)C	4.7	Good	6.1
1599	O1C(CC)C(C)C(=O)C(C)=C1C(C)C=1OC(CC)=C(C)C(=O)C=1C	4.8	Excellent	6.1
1423	OC(=O)c1c2nc3c(nc2ccc1)c(ccc3)C(=O)C	5.4	Excellent	6.1
13034	BrC1CCC(C)(C1(C)C)C1=CC2(O)C(CC1=O)(C)C(Br)CCC2(O)C	4.4	Excellent	6.1
11865	O(C(C)c1c2nc3c(nc2ccc1)c(ccc3)C(OC)=O)C	5.6	Good	6.1
11807	O1C(OC)c2c(ccc(C3(CC(CCC3)(C)C)C)c2C(C(O)=O)C)C1OC	5.9	OK	6.1
11514	O(C(=O)C(NC(=O)c1ccccc1)CCCNC(=O)c1ccccc1)C	4.9	Good	6.1
11036	O(C(=O)C)C1CC(=C)C(COC(=O)C)C(CCC(=C1)C)C(=CC=CC(O)(C)C)COC(=<br>O)C	5.4	Poor	6.1
10650	O1C(O)C(=CC1=O)CCCC(=CC=CC(CCC=C(CC(O)C(OC)=O)C)C)C	5.1	Bad	6.1

10072	[nH]1c2C3=NC(=NC3=C(C=Cc2nc1N(C)C)C)NC	5.0	Excellent	6.1
9200	OC1C=C(C2C3CCC(O)(C=CC3(CCC12C)C)C)C(C)C	5.2	Excellent	6.1
9187	O1C2CC3(CC(O)C4(C(C3CCC12C)C(=CC4O)C(C)C)C)C	5.2	Excellent	6.1
9045	O(C(=O)C)C1CC2C3C(CCC2(C)C1C(O)(CCCC(C)C)C)C1(C(=CC(=O)CC1)CC3)C	4.0	Poor	6.1
8541	O1C2=CC3(C(CC2=C(C)C1=O)C(=CC=C3)COC(=O)C)C	5.7	Excellent	6.1
7847	O1C2C(C(=C)C1=O)C(OC(=O)C)CC(=CC(OC(=O)C)CC(=CC(O)CC(=C2)C)C)C	5.2	Poor	6.1
7543	BrC1=C(C(OC1=O)=Cc1cc(Br)c(O)cc1)c1cc(Cl)c(O)cc1	5.8	Excellent	6.1
6366	O1C=C(COC(=O)C)C(C2(CCC3C(CCCC3(C)C)(C)C2C=O)C)C1OC(=O)C	5.1	OK	6.1
5557	O(C(=O)C)C1CC2C(C3CC=C(C=O)C(C=O)C13C)(CCC1C(CCCC12C)(C)C)C	4.8	Good	6.1
5437	o1cc(cc1CC(CCCc1cc(oc1)C=C(C(O)=O)C)C)CCCc1ccoc1	5.1	Bad	6.1
4158	O1c2c(cc(O)cc2)C=CC1(CCc1c(C)c(CO)c(OC)cc1C)C	4.9	Poor	6.1
3619	O=C1NCCCC1NC(=O)C=C(CCC1(C)C(CCC=C1C)C)C	5.4	Bad	6.1
3259	S(O)(=O)(=O)CCNC1=CC(=O)C(=CC1=O)CC1(C2CCC=C(C)C2(CCC1C)C)C	6.2	OK	6.1
2091	O1C23C(CCC(C2)C)C2(C(C1(O)C(CC2)C(C)C)C(=C3)C(OC)=O)C	4.7	Good	6.1
1549	S(OC1CCC2(C(=CCC3C4CCC(C(=O)C)C4(CCC23)C)C1O)C)(O)(=O)=O	4.9	Poor	6.1
14322	o1c2c(CCC3C4(C(CCC23C)C(CCC4)(C)C)C(O)=O)cc1	5.2	Excellent	6.1
13951	O1OC(CCC1(CCC=1C(CCCC=1C)(C)C)C)C(C(O)=O)C	5.1	Good	6.1
13617	O(C(=O)C)C1C(=CC2(C(=CC(=O)CC2C)CCC1=O)C)C	4.5	Good	6.1
13194	O(C(=O)C)c1ccc(O)c(CC2C3(C(CC=C2C)C(CCC3)(C)C)C)c1O	4.1	Poor	6.1
13002	O1C2=CC(O)(C)C3(O)C(CC2=C(C)C1=O)C(CC3)=C	5.3	Excellent	6.1
13001	O1C2=CC(O)(C)C3(O)C(CC2=C(C)C1=O)C(CC3)=C	6.2	Excellent	6.1
10649	O1C(=O)C(=CC1CC(=CCCc1ccoc1)C)CC=Cc1ccoc1	6.2	Excellent	6.1
9151	O=C1CCC2C(CCC3(C2CCC3C=C)C)C1(CCC(OC)=O)C	5.1	OK	6.1
8292	o1cc(cc1)CCCC(=CC(=O)CC(=CCCc1ccoc1)C)C	5.5	Good	6.1
8094	O(C)c1c2c(cc(OC)c1O)C(=O)c1c(C2=O)c(O)cc(c1)C	5.2	OK	6.1
7559	O=C1c2c(CC1(C)C)c(C)c(CCO[N+](=O)[O-])c(c2)C	4.8	Good	6.1
7541	Brc1cc(cc(Br)c1O)C=C1OC(=O)C(Cl)=C1c1ccc(O)cc1	5.3	OK	6.1
7076	O1C2(O)C3=C(C(CCC3C(CC(C=C(C)C)C2(O)C)C)C)C1=O	6.1	Excellent	6.1
6983	O(C(=O)C)C1CC(C2C3C(CCC3C)C12C)C(CC(O)C=C(C)C)=C	4.6	OK	6.1
5567	O(C(=O)C)C1CC2C(C3CC(O)C(=CC13C)C=O)(CCC1C(CCCC12C)(C)C)C	4.4	Excellent	6.1
5198	O(C(=O)C)C1CC2=CCC3C4CCC(C(=O)C)C4(CCC3C2(CC1)C)C	4.1	Bad	6.1
5009	O1C2C(C=C(C)C1C=CCCCCCCC)C1CC(O)CC1=CC2=O	4.3	Poor	6.1
4831	O1CC2C(CCC(O)(C=CCC2=C)C)C(=CC=CC(OC)(C)C)C1OC	5.3	Poor	6.1
2632	O1C(=Cc2ccc(O)cc2)C(=CC1=O)c1ccc(O)cc1	5.5	Excellent	6.1
2336	O1C2C(c3c(cc(OC)c(c3)CO)C(C)C2O)=C(C)C1=O	6.6	Excellent	6.1
872	OC1C2C(CC=C2C)C(=C)C(O)CC1C(C(OC(=O)C)CC=C(C)C)C	5.8	Good	6.1
13950	ClC1CCC(OC1(C)C)(C)C1C2C(CCC(O)(C)C2[NH+]=[CH-])C([NH+]=[CH-])(CC1)C	4.4	Good	6.1
13795	O1C(=CC(OC)=C(C=O)C1=O)C1C2C(CCCC2)C=CC1C	4.7	Excellent	6.1
13709	O(CC=1CCC(C(CCC=C(CCC=C(C)C)C)C=O)C=1C=O)C(=O)C	4.1	Bad	6.1
13203	O1C(C2=C(C3(C(CC2)C2(C(CC3O)C3(C(CC2)C(CCC3)(C)C)CO)C)C)C1=O)C	4.4	OK	6.1
12201	O(C(=O)C)C1CC(=CCC=C(C=O)C(C=O)C1C(CCC=C(C)C)C)C	5.0	Excellent	6.1
12152	O(C(=O)C)C1CC2C(C3CC=C(C(=O)C)C(C=O)C13C)(CCC1C(CCCC12C)(C)C)C	5.0	Bad	6.1
11442	O(C(C(C=COC(=O)C)=COC(=O)C)CC=C(C#CC=C(C)C)C)C(=O)C	4.2	Bad	6.1
11380	Brc1cc([NH+]2C=Cc3c([nH]c4c3cccc4)[CH-]2)c(cc1)C(OC)=O	4.4	OK	6.1
9937	OC1CC2(CC(O)C3(C(C2C2CC12C=O)C(CC3)C(C)C)C)C	4.8	Excellent	6.1
8299	O1c2c(c(cc(O)c2)C)C(=O)c2c1cc(OC)cc2O	5.8	Poor	6.1
8226	O1C(=O)C2C(=CC(O)C3C(CCCC23C)(C)C)C1=O	4.8	Excellent	6.1

7965	O1C2CCC(O)(C)C(CC=C3C(C4C(CCC3C)C(O)(CC4)C)(C)C)C2(CCC(=O)C1(C)C)C	4.9	Poor	6.1
7869	OC1CC2C3C(CCC2(C)C1C(CCCC(C)C)C)C1(C(CC(=O)CC1)C(=O)C3)C	4.4	Poor	6.1
7091	O1CC(C2C(C(CCC=C(CC2)C)=C)C1=O)=CCC=C(CO)CO	5.4	Excellent	6.1
6211	O1C(C2CCC3(C(CCc4c3coc4)C2(CC1=O)C)C)(CO)C	5.0	Excellent	6.1
5629	o1cnc(C(O)=O)c1-c1c2c([nH]c1C(N(C)C)CC(CC)C)cccc2	5.2	Good	6.1
5526	O1C23CC(O)CCC2(C)C(=O)C(=CC13)C1CCC(C(CCCC(C)C)C)C1(CC=O)C	4.1	Poor	6.1
5441	o1cc(cc1C=C(CCCc1cc(oc1)C(=O)NCCNC(=O)C)C)CCCc1ccoc1	4.7	Bad	6.1
5016	O1C2C(C=C(C)C1C=CCCCCCCC)C1CC(O)CC1=CC2O	3.4	Good	6.1
4510	O1C2C(C(C)C1(O)CCC(C)C)C1(C(C3C(CC1)C1(C(=CC(=O)C=C1)CC3)C)C2)C	5.3	OK	6.1
4362	O(C(=O)C)C1CC2(O)C(CC1)(C)C(=O)C(=CC2O)C1CCC(C(CCCC(C)C)C)C1(CC=O)C	4.7	Bad	6.1
4130	BrC1CCC2(C(CCC(=C)C2Cc2cc(O)c(cc2O)C=O)C1(C)C)C	5.2	Bad	6.1
3598	O(C(C(C=COC(=O)C)=COC(=O)C)CC(O)C(C#CC=C(C)C)=C)C(=O)C	3.9	Bad	6.1
3476	O1C2(C3C(CC(C)C2(CC1C1(OOC(CC1)C(C(O)=O)C)C)C)C=C(CC3)C)C	5.3	OK	6.1
2514	O1C(C2C(C3(C(CC2O)C2(C(CC3=O)C3(C(CC2)C(CCC3)(CC)C)C)C)C)C1=O)C	4.4	Poor	6.1
2134	O1C2CC=C3C(C(CCC12C)C(CCC=C(C)C)C)C(OC3)O	4.5	Excellent	6.1
1084	O1C(=O)C(=CC1CC(=CCCc1ccoc1)C)CC=Cc1ccoc1	5.0	Good	6.1
884	O1C2CCC(CCC3(C4(OC4CC12CO)C(CC3)C(O)(C)C)C)=C	5.9	Excellent	6.1
680	ClC1CCC(OC1(C)C)(C)C1C2C(CCC(O)(C)C2[NH+]=[CH-])C([NH+]=[CH-])(CC1)C	5.2	Good	6.1
216	OC1(C2C=C(CCC(=CCCC(O)(C(C1)C2)C)C)C(C)C)C	4.9	Good	6.1
12641	O(C(=O)C)c1ccc(O)cc1CC=C(CCC=C(C)C)C	4.7	OK	6.1
12424	O1C(C2C3(CCC4C(=CCC5C(C)(C)C(O)CCC45C)C3(CC2)C)C1=O)(CC(O)CC(C)=C)C	4.4	Bad	6.1
11473	O1c2c(cc(OC)cc2)CC2C3(C(CCC12C)C1(C(CC3)C(CCC1)(C(O)=O)C)C)C	5.7	Excellent	6.1
9494	O1C2OC2C(CCCC(CC(O)CC(=CCCc2ccoc2)C)C)C1=O	5.2	Excellent	6.1
8229	O1C(OCC)C2=C(C(O)CC3C(CCCC23C)(C)C)C1=O	5.1	Excellent	6.1
7485	O(C(=O)C)C1CCC2(C(CCC3(C2CC(=O)C3=C(C=CC=C(C=O)C)C)C)C1(C(OC)=O)C)C	4.1	OK	6.1
7170	OC1(CCC2C(CCCC2(C)C)(C)C1CCC(=CCO)C)C	4.7	Excellent	6.1
7147	OC(=O)CCc1nc2c(nc1)cc1c(c2)cccc1	3.5	Good	6.1
6895	O1C2(OC)C(C3C(C(OC(=O)C)CC3C(=CCC=C(C)C)C)C1OC)C(CC2)C	4.8	Bad	6.1
6327	N(CCCN1CCCCCCCCC=CCCCC1)CCCN	2.3	Bad	6.1
6292	OC(CC=CC(O)(C)C)(C)C1C2CCC(=CCC2(CC1)C)C	5.1	Good	6.1
6250	OC1CC2C(CCC3C2(CCC2C(CCCC23C)(C)C)C)(CC)C(C=O)C1C(=O)C	5.0	Poor	6.1
5283	O1C(CC=CCCC1=O)C1CC1C(O)C=CC(O)CC=CCCCCC	5.2	Poor	6.1
5282	O1C(CCCCCC1=O)C1CC1C(O)C=CC(O)CC=CCC=CCC	5.6	Poor	6.1
4874	O(C(COC(=O)C)CO)C(=O)C=C(CCC=1C(CCCC=1C)(C)C)C	4.5	Poor	6.1
4780	S(C)c1[nH]cnc1Cn1c2c(c3c1cncc3)cccc2	4.7	Excellent	6.1
4537	O1C2CC3C4(CCC(C(CCC=C(C)C)C)C4(C)C(O)CC3(C)C(CCCO)C12C(=O)C)C	4.8	Bad	6.1
4463	O(C)c1cc(O)cc(C(=O)c2cc(cc(OC)c2O)C)c1C=O	6.2	Poor	6.1
3284	O1C2C(=CC1=O)C(OC(=O)C)CC1C(CCC3C4(C(CCC13C)C(CCC4)(C)C)C)C2O	4.8	Bad	6.1
2953	OC1(CCC(C(=CCC=C(C)C)C)C1C1C(C=CC1=O)C)COC(=O)C	5.0	OK	6.1
1012	O1C(=O)C(CCC1C(C)C1CCC2C3C(C4(C(CC(=O)CC4)CC3)C)C(OC(=O)C)CC12C)C	3.6	Bad	6.1
252	O1C23CC(CCC(=O)C(C)C)(C)C1(O)CCC2(C)C(O)CCC3=C	4.8	Excellent	6.1
221	O1C2C(=CC1OO)C(C1C(C=C(CC1)C)C2OC)(C)C	5.4	Excellent	6.1
9846	O(C)c1c2c(ccc1)C(=O)c1c(C2=O)c(O)ccc1	5.0	Excellent	6.1

8170	O(C(=O)C)C1C=C(CO)C(=O)C(O)C1C(=CC=CC(C=CCC(C)C)C)C	4.0	Good	6.1
7808	O1C2C(CCC(=C)C(OO)CCC(=C2)C)C(=C)C1=O	5.0	OK	6.1
5295	O1C(CCCCCC1=O)C1CC1C(O)C=CC(O)CC=CCCCCC	5.1	Good	6.1
4346	O(C)c1c2c(cc(CCC)c1C(OC)=O)C(=O)c1c(C2=O)c(OC)cc(OC)c1	5.0	OK	6.1
2797	S(=O)(=O)(C)c1c2c([nH]c3c2cccc3)c(nc1)CC	4.9	Excellent	6.1
661	S(OC1CC2=CCC3C4CCC(=O)C4(CCC3C2(CC1)C)C)(O)(=O)=O	6.0	Excellent	6.1
192	o1cc(cc1)CCCC(CC(=O)C=C(CCCc1ccoc1)C)C	4.1	OK	6.1
11170	O(C(=O)C)C1C=C(C)C(=O)CC1C1(O)CC2(C(CC1)C1(C(C(CCC1)(C)C)C(OC(=O)C)C2)C)C	3.6	Poor	6.0
9194	O1C2CC3C4C(CCC3(CC(=O)C12C)C)(C)C(=O)C=C4C(C)C	4.3	Excellent	6.0
9189	OC1C=C(C2C3CCC(=C)C(O)CC3(CCC12C)C)C(C)C	5.1	Excellent	6.0
9053	OC1CC2=CCC3C4CCC(C(CCC(O)=O)C)C4(CCC3C2(CC1)C)C	4.2	Bad	6.0
8189	O1c2c(C=CC1(CCC1(CCCC(C)C1=C)C)C)c(O)c(OC)cc2O	5.3	OK	6.0
7833	O1C2(O)C(=CC1=O)C(C1C(C2)C=C(CC1)C)(C)C	5.5	Good	6.0
7160	O1C(CC(=CCCC(C=CC=C(CCCC2=CCN(CCO)C2=O)C)C)C)C(O)=C(C)C1=O	5.3	Bad	6.0
6041	O1C2(C(=C(C(=O)CCCCCCC)C1=O)C(=C1C(=COC(=C1)C)C2O)C=O)C	4.7	Excellent	6.0
6019	O1C2C1(C(OC)=O)C(=O)C1(C(C3(C(CC1)C(CCC3)(C)C)C)C2=O)C	5.0	Good	6.0
5522	O(C)c1cc(cc(CC2(C3CCCC(=C)C3(CCC2C)C)CO)c1O)C(OC)=O	4.5	Poor	6.0
4916	O1C2C(CC3C(C)(C(OC(=O)CC)CCC(=C2)C)C(OC(=O)C)CC=C3C)=C(C)C1=O			6.0
3936	O1C2CC(=CCCC(C(C2C(CCC=C(C)C)C)C1=O)=COC(=O)C)C	5.6	OK	6.0
3617	O1OC(CCC1(CCC1(O)C2(C(CCC1C)C(CCC2)(C)C)C)C)C(C(O)=O)C	4.9	OK	6.0
2095	OC1CC2(C(CCC(=C)C(O)CC=C1C)C(CC2)=C(C)C)C	5.3	Excellent	6.0
1286	OC1=C(C)C(=O)C2=C(C1=O)C(CCC2C(CC(O)C=C(C)C)C)C	5.5	Good	6.0
86	OC1CC2=CCC3C4CCC(C(O)C)C4(CCC3C2(CC1)C)C	4.2	Good	6.0
14431	O1C(C2(C=C(C3OC3C2C(C(O)C(C(=O)CC)C)=C)C)C)=C(C)C(=O)C(C)=C1OC			6.0
13560	O1C(OC)C(C2C(C(CC(O)C=C(CC2)C)=C)C1OC)=CC=CC(C)C	5.2	Excellent	6.0
13221	O1C(O)C2=C(C3C(C3)C(CC(OC(=O)C)C2C(CCC=C(C)C)C)C)C1=O	3.9	Poor	6.0
12627	O1C(CC=C(CCC=C(CCC=2C(CCCC=2C)(C)C)C)CO)C(=CC1=O)CO	5.5	Bad	6.0
10181	O(C(CO)CO)C(=O)CC(CCC1(C=2C(CCC1C)C(CCC=2)(C)C)C)C	4.3	Good	6.0
9291	O1OC(CCC1(CCC1(C=2C(CCC1C)C(CCC=2)(C)C)C)C)C(C(O)=O)C	5.4	Bad	6.0
9181	Oc1c2c(cc(c1)C)C(=O)c1c(C2=O)c(O)ccc1	5.5	Poor	6.0
8536	O1C2CC3(C(CC2=C(C)C1=O)C(=CC=C3)COC(=O)C)C	5.5	Excellent	6.0
8463	O1OC(CCC1(CCC1(O)C(CCCC1=C)(C)C)C)C(C(OC)=O)C	5.1	Excellent	6.0
7939	O1C2C(CCC(=C)C(=O)CCC(=C2)C)C(=C)C1=O	5.5	Excellent	6.0
7371	O1C2CCC(=C)C(C(OC)=O)C(CCC12C)C(=CC=CC(O)(C)C)COC(=O)C	5.7	Poor	6.0
6450	o1c2c(cc1)C(=O)CC1C2(C)C(O)CC2C1(CCC1C(CCCC12C)(C)C)C	4.3	Poor	6.0
6060	O(C)c1cc(O)c2c(c1)C(=O)c1c(C2=O)c(O)cc(c1)COC(=O)C	6.6	Good	6.0
3947	O1C(CC=C(CCC=C(CCC(=O)C(CCCC(=O)C)(C)C)C)C1O)C1=CC(OC1O)=O	4.2	Bad	6.0
2994	O1CC(=CC1=O)C(O)CC=C(CCC=C(CCC=1C(CCCC=1C)(C)C)C)CO	4.8	Poor	6.0
2815	O=C1CC2C(CCCC2(C)C)(C)C(CCC(CC(OCC(O)CO)=O)C)=C1C	4.5	Good	6.0
2515	O1C(C2=C(C3(C(CC2)C2(C(CC3O)C3(C(CC2)C(CCC3)(CC)C)C)C)C)C1=O)C	5.6	Good	6.0
1665	Brc1cc2c3CCN4OCSCC(NC(=O)C)C4c3[nH]c2cc1	5.6	Good	6.0
928	O1C2C3C(CC2C1=O)=C(CCC3COC(=O)CC(C)C)C1(CC(CCC1)(C)C)C	6.2	Good	6.0
267	O1C2(O)C(C)C3(OC1(CC)C(C(O3)C2C)C)C(C(O)C(=CC(=CCC)C)C)C	4.8	Good	6.0
253	O1C23CC(CCC(=O)C(C)C)(C)C1(O)CCC2(C)C(OC(=O)C)CCC3=C	5.6	Poor	6.0
218	O1C2CC=C(C1CC1=CCC(C(C)C)C1(CC(=O)C2(O)C)C)C	4.0	OK	6.0
12779	OC12C(C3C1(C)C(O)CC3C(=CCC=C(C)C)C)C(CC2)C	5.2	Excellent	6.0
12616	Oc1cc(O)ccc1C=CC(OCC1C2(C(CCC1=C)C(CCC2)(C)C)C)=O	4.6	Bad	6.0
11505	o1cc(cc1)C(O)CC1C(CC2C1(CCC1C(CCCC12C)(C=O)C)C)(C=O)C	5.7	Poor	6.0
11001	O1C(C2(C=C(C3OC3(C)C2C(=CCC)C)C)C)=C(C)C(=O)C(C)=C1OC	5.2	Excellent	6.0

9560	O1c2c(cc(O)cc2C)CC2C3(C(CCC12C)C1(C(CC3)C(C)(C)C(O)CC1)C)C	4.8	Bad	6.0
9146	O1CC(C2C(C(CCC=C(CC2)C)=C)C1O)=CC=CC(O)(C)C	5.3	Excellent	6.0
8584	O(C(=O)C)C1CC2=CCC3C4CCC(C(=O)C)C4(CCC3C2(CC1)C)C	4.2	Bad	6.0
8063	O=C(NN=C(CCc1ccccc1)c1ccccc1)N	5.5	Bad	6.0
7776	O1C2C1(CC=C(CCC=C(CCC=C(C)C)C)C)C(=O)C=C(CO)C2O	4.0	Excellent	6.0
7562	O([N+](=O)[O-])C(CO)c1c(c2CC(Cc2cc1C)(C)C)C	5.1	OK	6.0
6246	O1C(CC2C3(C(CCC12COC(=O)C)C1(C(CC3)C(CCC1)(C)C)C)C)C1=CC(OC1O)=O	4.2	Bad	6.0
5403	O(C(=O)C)C1CC(C)(C)C(=C=CC(=CC=CC(=O)C)C)C(O)(C1)C	5.3	Poor	6.0
5103	O(C(=O)C)c1c2c(cccc2)c(OC(=O)C)cc1CC=C(C)C	3.9	Excellent	6.0
4787	O(C(=O)C)C1CC(=O)C=C2CCC3C(C12C)C(O)CC1(C3CCC1C(=O)COC(=O)C)C	5.7	Poor	6.0
4154	O(C)C1=CC(C)(C)C(CCC(=CCc2cc(O)ccc2O)C)=C(C)C1=O	4.2	OK	6.0
2418	O1C23C1C(O)C=1C4CCC(C(CCC(C(C)C)=C)C)C4(CCC=1C2(CCC(O)C3)C)C	4.8	Bad	6.0
2371	O1OC(CCC1(CCC1(C2CCCC(C)(C)C2(O)CCC1C)C)C)C(C(O)=O)C	6.3	Excellent	6.0
759	O1C2OC(=O)C3(CC4(C(C3)C(=CC=C4)COC(=O)C)C)C12C	5.3	Good	6.0
14183	Brc1cc(OC)c2[nH]cc(Br)c2c1-c1c2cc(Br)ccc2[nH]c1	4.5	Excellent	6.0
13564	O1C(=O)C(C)=C(O)C(C)=C1C(=CC(CC(CC(CCC)C)C)C)C	4.9	Good	6.0
13400	O1c2c(cc(O)cc2C)CC2C3(C(CCC12C)C1(C(CC3)C(C)(C)C(O)CC1)C)C	4.4	Poor	6.0
13256	OC1CC2=CCC(C3CCC(C(CCCC(C)C)C)C3(CCO)C)C(=O)C2(CC1)C	4.0	Bad	6.0
13209	O1C(OC(=O)C)C2C(=CCC3C(CCCC23C)(C)C)C1OC	5.3	OK	6.0
12917	O(C)c1c(cc2c(c1OC)C(=O)c1c(cccc1O)C2=O)C	4.1	Excellent	6.0
12735	Brc1cc(OC)c2[nH]cc(Br)c2c1-c1c2cc(Br)ccc2[nH]c1	4.9	Excellent	6.0
9063	O(C(C(C(O)CC=CC=CC(=O)N)C)C(CC)C)C(=O)C=Cc1ccccc1	4.6	Good	6.0
8724	o1cc(cc1Cc1ccoc1)CCC=C(CCCC(CC(O)=O)C)C	4.7	Poor	6.0
7226	OC1CC2C3C(CCC2(C)C1C(O)(CCCC(C)C)C)C1(C(CC(=O)CC1)CC3)C	3.4	Bad	6.0
6225	O1CC=2C(C3(C(C4C(CC3O)C3(C(CC4)C(C)(C)C(=O)CC3)C)CC=2)C)C1O	4.9	Good	6.0
5298	O1C(CC=CCCC1=O)C1CC1C(O)C=CC(O)CC=CCC=CCC	4.1	Good	6.0
5284	O1C(CC=CCCC1=O)C1CC1C(O)C=CC(O)CC=CCC=CCC	3.7	Bad	6.0
4875	O(C(COC(=O)C)COC(=O)C)C(=O)C=C(CCC=1C(CCCC=1C)(C)C)C	4.8	Bad	6.0
4857	O1CC2C(CCC(O)(C=CCC2=C)C)C1(C=CC=C(C)C)CO	4.5	Good	6.0
4799	O1C23CC(O)CCC2(C)C(=O)C(=CC13)C1CCC(C(C=CC(C(C)C)C)C)C1(CCO)C	4.9	Poor	6.0
4778	Brc1cc2n(c3c(c2cc1)ccnc3)Cc1nc[nH]c1SC	5.3	Excellent	6.0
4725	O1CC2C(CCC(O)(C=CCC2=C)C)C1(C=CC=C(C)C)CO	5.7	Good	6.0
4694	O1C2C(CC3OC3(CCC(OC(=O)C)C(CCCC(C)C2OC(=O)C)=C)C)C(=C)C1=O	4.5	Bad	6.0
3670	O1C(CC=CC#C)C(OC(=O)C)CC(O)C(O)CC1C(OC(=O)C)CC	5.1	Excellent	6.0
1247	O(C)C1C=C2C(CCC3(C2CCC3C(CCCC(C)C)C)C)C2(CCC(O)CC12O)C	5.0	Poor	6.0
771	O1c2c(c(OC)c(OC)c3c2c(OC)cc(O)c3)C(=O)C=C1CCC	5.6	Excellent	6.0
14450	O(C)c1cc2c(c(CCC)c1C(OC)=O)c(O)c1c(c2)C(C)(C)C(=O)C(O)(C)C1=O	4.4	Poor	6.0
13662	O1C(O)(C(C(=O)C(CC(=CCC)C)C)C)C(C)C(O)C(C)C1C(C(=O)CC)C	4.4	Good	6.0
13640	O1C(C)C(OC(=O)C)C(O)C(O)C1OC1CC2CCC3C4CCC(C=C)C4(CCC3C2(CC1)C)C	4.4	Bad	6.0
13470	O1CC(C2C(C(CCC(O)C(CC2)=C)=C)C1=O)=CC=CC(O)(C)C	6.7	OK	6.0
13066	O=C1C23C(CCC2(C=C(C(O)=O)C3C)C)C(C1)C	5.2	Good	6.0
11882	O(C(=O)C)C1CC2C(C)(C(C=O)C1C(=O)C)C(O)CC1C2(CCC2C(CCCC12C)(CC)C)C	4.3	Poor	6.0
11742	O1c2c(C(=O)C=C1c1cc(O)c(OC)cc1)c(OC)cc(O)c2	4.9	Good	6.0
10960	O1OC(CCC1(CCC(O)C(CCC=1C(CCCC=1C)(C)C)=C)C)C(C(O)=O)C	3.8	Poor	6.0
9935	OC1CC2(C(C3C(CC2)(CCC3C(C)C)C)C2CC12CO)C	4.6	Excellent	6.0
9547	BrC1=CC2(Oc3c(C2)cc(cc3Br)CO)C=C(Br)C1=O	4.3	Excellent	6.0
9493	O1C(O)C(=CC1=O)CCC=C(CC(O)CC(CCCc1ccoc1)C)C	5.1	Excellent	6.0

9490	O1C(=O)C(=CC1O)CCCC(CC(O)CC(=CCCc1ccoc1)C)C	5.8	Poor	6.0
9391	O1C(O)C2C(=CCC3C2(C)C(O)CC2C3(CCC3C(CCCC23C)(C)C)C)C1=O	4.3	Bad	6.0
8450	Brc1c2c(n(C)c1Br)C(=O)N1C3(N=C(NC23)N)CCC1	2.9	OK	6.0
8230	O1C(OCC)C2=C(C(O)CC3C(CCCC23C)(C)C)C1=O	5.1	Excellent	6.0
8064	O1C(=O)C(CC1C=C(CCCc1ccoc1)C)=CCCc1ccoc1	4.4	Poor	6.0
6489	OC1(CCC2C(=C1)C(CCC2(C#N)C)C(C)C)C	5.7	Excellent	6.0
6442	OC1CC2(C(CC1C(=O)C)C(CC2)C(O)(CCC=C(C)C)C)C	5.3	Good	6.0
6145	O=C1NC(=NC1(CC(=O)C)c1c2c([nH]c1)cccc2)N(C)C	5.5	OK	6.0
5362	n1c2c3C(CC(C)C2CC=CCCC)CCc3nc1N	4.4	Excellent	6.0
4296	O=C1N=C(NC)N(C)C1=Cc1c2c([nH]c1)cccc2	4.8	Poor	6.0
3625	o1cc(cc1)CCC=C(CC(=O)CC(CC=Cc1ccoc1)C)C	4.2	Poor	6.0
13445	O(C(=O)C=CC(O)(C)C)C1C2C(CCC1C(=O)C)(C)C(O)CCC2=C	5.3	Good	6.0
13115	O1C2(O)C(C3CCC(C(C=CCC(C)C)C)C3(CC2)C)=CC1=O	4.4	Good	6.0
12368	O1C2CC3(C(CC(=O)CC3(C)C)C1=O)C=CC2(O)C	5.6	Excellent	6.0
12147	O=C1CC2C3(C(CCC2(C2CC=C(C(=O)C)C(C=O)C12C)C)C(CCC3)(C)C)COC(=O)C	4.7	OK	6.0
12003	O1C(C)(C)C1CCC(C)C1CCC2C3C(CC(OC(=O)C)C12C)C1(C(=CC(=O)CC1)CC3)C	5.1	OK	6.0
10674	OC1C2C(CC=C2C)C(CCC1C(O)(CCC=C(C)C)C)=C	4.0	OK	6.0
10396	O1C2CC3C(C(O)C(CCC3=C)C(CC(O)C=C(C)C)C)C12C	4.9	Excellent	6.0
10393	O(C(=O)C)C1C2C(CC=C2C)C(CCC1C(CCC(O)C(C)=C)C)=C	4.2	Poor	6.0
9810	n1c2c(n(C)c1NC)C(=C1N=C(N=C1C=C2)N(C)C)C	4.8	Good	6.0
8341	O1C=C2C(=CC(=O)C(C(O)=O)=C2O)C(C)=C1C(CC)C	5.0	Excellent	6.0
8225	O1C(=O)C2C(=CCC3C(CCCC23C)(CO)C)C1=O	4.3	Excellent	6.0
8105	O1OC(CCC1(CCC=C(CCC(=O)C(CCCC(=O)C)(C)C)C)C)C(C(O)=O)C	5.1	Poor	6.0
5524	BrC=1CC23N(C=1Br)C(=O)C=1N(C2NC(=O)N3)CCC=1	6.5	Excellent	6.0
5516	O1C(=O)C(=C2CC=3C(CCC=3C)C(CC12O)C)C	5.0	Excellent	6.0
5211	O1OC(OC)(CC(C)C1CC(O)=O)CCCC=CC(=CCC)C	4.8	Excellent	6.0
4241	O1C(C1(C)C)C1OC2OC(O)C3C(C2=C1)CCC(O)(C1OC1CC3=C)C	4.8	Good	6.0
3892	[NH+]12CCc3c([nH]c4c3cccc4)[C-]1C=C1NC=NC1=C2	4.7	Excellent	6.0
3550	O(C(=O)C)C1C(=CC2(C(CCC1=O)=CCCC2C)C)C	5.4	Good	6.0
3396	O1CC2C(C1(O)C)C(OC(=O)C)CC1C2(C)C(=O)CC2C1(CCC1C(CCCC12C)(CC)C)C	5.8	Poor	6.0
3185	O1c2c(C=CC1(CCC1(CCCC(C)C1=C)C)C)c(O)c(OC)c(OC)c2OC	4.8	Bad	6.0
2633	O1C(=Cc2ccc(OC)cc2)C(=CC1=O)c1ccc(O)cc1	4.8	Excellent	6.0
1962	Brc1cc2[nH]cc(c2cc1)C1N(C)C(=NC1)C(=O)c1c2c([nH]c1)cc(Br)cc2	4.5	Bad	6.0
1872	O(C)C1=CC(=NC1=CNCCc1ccccc1)c1[nH]ccc1	4.4	Excellent	6.0
1856	o1cc(cc1)CCCC(=CC(=O)C=C(CCCc1ccoc1)C)C	5.5	Poor	6.0
1066	O1C(=O)C(=C2CC3(C(CC12O)C(=O)CCC3C)C)C	5.3	Excellent	6.0
13609	O(C=O)C1CCC2=CC(O)CC(C)C2(C)C1C(=O)C	5.0	Excellent	6.0
7731	O1C(=O)C(=CC1CC(=O)C)CCC(C(O)c1oc(cc1C)C=O)C(C)=C	5.4	Poor	6.0
6515	BrC1=C2C(C=3S(=O)(=O)C=CNC=3C1=O)=CN(CC2)C	4.9	Excellent	6.0
5613	O1C(O)(C)C(=O)C(C)=C1C(=CC(CC(CC(CCC)C)C)C)C	4.9	Good	6.0
4319	O(CC(OC)=O)c1c2nc3c(nc2ccc1)cccc3	5.6	Good	6.0
1159	o1cc(cc1)CCCC(=CC(O)CC(=CCCc1ccoc1)C)C	5.2	Excellent	6.0
14071	O1CC2(CC2C=C(CCC=C(C)C)C)C2C(C(=CC2)C=O)C1=O	4.0	OK	5.9
13446	O(C(=O)C=CC(O)(C)C)C1C=C(CCC(O)C(CCC1C(=O)C)=C)C	4.2	Poor	5.9
13208	O1C=C2C(C3(C(CC2OC(=O)C)C(CCC3)(C)C)C)C1OC(=O)C	5.3	Good	5.9
13205	O1C2CC3C4(C(CCC3(C3CC=C(C(C23C)C1=O)C(O)C)C)C(CCC4)(C)C)COC(=O)C	4.6	Poor	5.9
12877	O1C2C=C(CCC=C(CCC(OC(=O)C)C1(CCC2C1(OC1)C)C)C)C	4.8	Excellent	5.9
12011	OC1C(=O)C(=CC=C(CCCC(CO)C(=O)CCC1C)C)C(C)C	4.7	Excellent	5.9

11774	OC1C2CC(O)CCC2(C=2C(C3CCC(=C(CC(=O)CC(C)C)C)C3(CC=2)C)C1)C	4.3	Bad	5.9
11704	O(C(=O)C)C1CC2C(C3CC=C(C=O)C(C=O)C13C)(CCC1C(CCCC12C)(C)C)C	5.2	Poor	5.9
11492	O1CC(C2C(C(CC(O)C=C(CC2)C)=C)C1=O)=CC=CC(O)(C)C	5.3	Excellent	5.9
8526	O(C)c1c2c(cc(C)c1O)cc1c(c2)C(=O)C(=O)C=C1	5.9	Excellent	5.9
5417	O(C)C1C(O)(c2c(NC1=O)cccc2)c1ccc(OC)cc1	4.6	OK	5.9
4317	O(C(=O)C)C1CC=C(C)C(=O)CC2C(CCC2(O)C(C)C)(CC=C1C)C	4.0	Excellent	5.9
3098	O1C2CC3C4(C(CCC3(C3CC(O)C(C(C23C)C1=O)C(=O)C)C)C(CCC4)(C)C)CO	3.6	Excellent	5.9
14010	OC1C2CCC3C4CCC(C=C)C4(CCC3C2(CCC1O)C)C	4.1	Excellent	5.9
13848	O(C(=O)C)C1Cc2c(C1(O)C)c(ccc2C=O)C=C(C)C	6.6	Excellent	5.9
13634	OC1CC2CCC3C4CCC(C=C)C4(CCC3C2(CC1)CO)C	4.5	Bad	5.9
13311	O1c2c(cc(O)cc2C)C=CC1(CCC=C(C=O)C)C	5.6	Excellent	5.9
12935	BrC(CO)C1(CC2C(=CC1)C1(C(CC2O)C2(C(C2)C(OC(=O)C)C1)C)C)C	5.1	OK	5.9
11477	O1CC23C(CCC4(C5Cc6cc(OC)ccc6OC5(CCC24)C)C)C(CCC3)(C)C1=O	4.0	Bad	5.9
11215	o1cc(cc1)CCCC(CC1CC2(C(CCC1=C)C(CCC2)(CO)C)C)CO	5.8	Poor	5.9
10783	OC1CC2CC(C3C(C4CCC(C(O)(C(OC(=O)C)C=CC)C)C4(CC3)C)=CC2=O)=C C1	3.9	Poor	5.9
9357	O1C(CC(O)CC=CC(OC(CC=CC1=O)C)=O)C	5.2	Excellent	5.9
9124	O(C)c1c2c(cc(C)c1O)C(=O)c1c(C2=O)c(O)ccc1	4.6	Excellent	5.9
8500	OC12CC3(C(CC1)C1(C(CC3)C(CCC1)(CO)C)C)CC2=C	3.5	Excellent	5.9
7420	O(C(=O)C)C1C(C)=C(CCC(=CCO)C)C2(C(C1OC(=O)C)C(CCC2)(C)C)C	4.9	OK	5.9
6965	Brc1cc2n(cc(S(=O)(=O)c3c4c([nH]c3)cc(Br)cc4)c2cc1)C(O)=O	5.0	Poor	5.9
6192	O(C(=O)C)c1cc(ccc1O)CCC=CC=CC=CCCOC(=O)C	3.7	Bad	5.9
6169	O1C(CC=C(CC=CC(=CC(O)C)C)C)=C(C)C(=O)C(C)=C1OC	5.0	Good	5.9
5350	O1C(O)C(=CC1=O)C(O)CC1(C2C(=CCC1C)C(CCC2)(CCCC(C)=C)C)C	3.9	Bad	5.9
4825	O1C2C(CC3OC(CCC3(OC)C)C(O)(CCCC(C)C2OC(=O)C)C)C(=C)C1=O	6.8	Good	5.9
4683	O1C2C(CC3OC3(CCC(OC(=O)C)C(CCCC(C)C2OC(=O)C)=C)C)C(=C)C1=O	5.4	Poor	5.9
3986	O1C(CC2C3(C(CCC12COC(=O)C)C1(C(CC3)C(CCC1)(C)C)C)C)C1=CC(OC1 O)=O	4.6	Good	5.9
3978	O1C2C1(CCC=C(CCC1(C)C(C(CCC1C)C2=O)C=O)C)C	5.5	Good	5.9
3739	O1C2CC(=CCC(OC)C3=C(C1NC3=O)C2C(CCC=C(C)C)C)C	5.7	Poor	5.9
2765	o1cc(cc1)C(O)CC1C(CC2C1(CCC1C(CCCC12C)(C)C)C)(C=O)C	5.3	OK	5.9
1212	O1C(CCC1C([NH+]=[CH-])(C)C)(C)C1C2C(CCC([NH+]=[CH-])(C)C2O)C([NH+]=[CH-])(CC1)C	4.2	Good	5.9
537	O=C1CC2C3(C(CCC2(C2CC=C(CC12C)C(=O)C)C)C(CCC3)(C)C)C(O)=O	6.1	Bad	5.9
14301	O(C(=O)C1C2(C(CC=C1C)C(CCC2)(C)C)C)CC(O)COC(=O)C	4.1	OK	5.9
14073	O(CC=1CCC(C(CCC=C(CCC=C(C)C)C)C=O)C=1C=O)C(=O)C	3.7	Bad	5.9
13523	O(C(=O)C)C1CC(C2C3C(CCC3C)C12C)C(=CC=CC(O)(COC(=O)C)C)COC(= O)C	4.9	Poor	5.9
12993	O1CC2=C(C3C(C3)C(CC(O)C2C(CCC=C(C)C)C)C)C1=O	4.7	Good	5.9
12095	O1C(CCC=C(CCC=C(CC(=O)C=C(C)C)C)C)(C)C1CO	3.1	Good	5.9
11049	O=C1N(C)C(=NC)N(C)C1=Cc1c2c([nH]c1)cccc2	4.3	OK	5.9
10436	O1c2c(c(cc(c2)C)CO)C(=O)c2c1cccc2O	4.7	Excellent	5.9
9985	O(C)c1c(O)c(CC=C(CCC=C(C)C)C)c(C)c(O)c1OC	5.0	Excellent	5.9
9403	O(C(=O)CN(CC(=O)NC1C2(C=C(CC2)C)C(CCC1C(C)C)C)C)C	5.0	OK	5.9
8654	O=C1CC2C(CC(CCC2C)C(OC(=O)C)(C)C)=C1C	6.0	OK	5.9
8412	O=C1C=C(C)C(=O)C=C1CC=C(CCCC(=CC(=O)CC(C)C)C)C	4.9	Poor	5.9
8227	O1C(=O)C2C(=CCC3C(CCCC23C)(C)C)C1=O	5.4	Excellent	5.9
8132	O1c2c(cc(O)cc2C)CC2C3(C(CCC12C)C1(C(CC3)C(C)(C)C(O)CC1)C)C	4.5	Poor	5.9
7566	O=C1c2c(CC1(C)C)c(CO)c(CCO[N+](=O)[O-])c(c2)C	4.4	Excellent	5.9
7542	Brc1cc(ccc1O)C=C1OC(=O)C(Cl)=C1c1ccc(O)cc1	5.6	Excellent	5.9
7372	O1CC(C2C(C(CCC(O)C(=CC2)C)=C)C1=O)=CC=CC(C)C	5.9	Good	5.9
6682	OC1CC2=CCC3C4CC(=O)C(C(O)(CCC(C(C)C)=C)C)C4(CCC3C2(CC1)C)C	4.4	Poor	5.9

6524	O1CC12C1OC3C=C(CCC3(COC(=O)C)C2(C)C(OC(=O)C)C1)C	5.8	Excellent	5.9
6437	BrC1CC(CCC1(O)C)C(=CC(O)C1C(C)=C(CCC1=C)C)C	5.5	Excellent	5.9
6260	O(C(=O)C)C1C23C(CCC2(C=C(C(OC)=O)C3C)C)C(C1)C	4.6	Poor	5.9
5807	O1C(CC2C(CCC3C4(C(CCC23C)C(CCC4)(C)C)C)C1OC(=O)C)C1=CC(OC1O)=O	4.1	Bad	5.9
5779	O1C(CC=CCC=CCC=CCCCCC1=O)C(O)CC	5.1	Good	5.9
5500	O1C23CC(OC(=O)C)CCC2(C)C(=O)C(=CC13)C1CCC(C(CCCC(C)C)C)C1(CC=O)C	5.5	Bad	5.9
5019	O1C2C(C=C(C)C1C=CCCCCCCC)C1CC(O)CC1=CC2=O	5.1	OK	5.9
4868	O(C(C(C(O)CC=CC=CC(=O)N)C)C(CC)C)C(=O)C=Cc1ccccc1	4.7	Good	5.9
4716	ClC1C(C2CC=C(C=O)C(C=O)C2(CC1O)C)(CCC(C(C)=C)C)C	5.8	Excellent	5.9
4316	O(C(=O)C)C1CC=C(C)C(=O)CC2C(CCC2(O)C(C)C)(CC=C1C)C	5.4	Poor	5.9
3257	O1c2c(cc(O)cc2C)CC2C3(C(CCC12C)C1(C(CC3)C(C)(C)C(O)CC1)C)C	4.9	Bad	5.9
1810	O1C2C=3C(CCC2C(=C)C1=O)(C)C(O)CCC=3C	4.6	Excellent	5.9
1134	O(C(=O)C)C1c2c(C(=O)C1(C)C)c(C)c(OC)cc2C(C)C	5.6	Excellent	5.9
13902	O1C(=O)C(C)=C(O)C(C)=C1C(=CC(CC(CC(CC)C)C)C)C	3.5	Excellent	5.9
13758	OC1CCC(C2C(CCC1=C)C(C2)(CC(O)C=C(C)C)C)=C	4.8	Excellent	5.9
13559	O(C(=O)C)CC(=CCC(O)C(O)(C)C)C1CCC(=CCCC(=C)C1C(OC)=O)C	5.3	OK	5.9
13352	O1C2CCC(=CC(CCC12C)C(=CC=CC(O)(C)C)C=O)C=O	4.8	Excellent	5.9
12999	O1C2=CC(O)(C3C(CC2=C(C)C1=O)C(CC3)=C)C	6.1	Excellent	5.9
10745	O(C(=O)C)C1CCC(O)(C=CC(CCC1=C)C(C)C)C	4.6	Excellent	5.9
9934	OC1CC2(CC(O)C3(C(C2C2C1(CCC2C(C)C)C)C3)C)C	4.5	Excellent	5.9
9241	O(C)c1c2nc3c(nc2c(cc1)CO)cccc3C(O)=O	5.2	Poor	5.9
7454	O(C(=O)C)CC=C(CCC=C(CCC1C(CCCC1C)(C)C)C)C=O	6.1	Poor	5.9
7397	OC(=O)c1c2c(n(c1)Cc1ccccc1)cccc2	7.0	Excellent	5.9
7019	O(C)c1c2c(cc(OC)c1)C(=O)c1c(C2=O)c(OC)cc(OC)c1	5.3	Poor	5.9
6468	O(C(=O)C)C1C2C(CCC2C(OC(=O)C)(C)C)(C=CC(CC(=O)C=C(C1)C)C)C	4.6	Good	5.9
6467	O(C(=O)C)C1C2C(CCC2C(OC(=O)C)(C)C)(C=CC(CC(=O)C=C(C1)C)C)C	4.5	Poor	5.9
5892	O(C(=O)C)C1C(C=2C(C1(C)C)C(OC(=O)C)CC(C)C=2C=O)(C=O)C	5.1	Excellent	5.9
5824	O1CC(OC)C(O)C(OC)C1Oc1cc(c2nccc(c2c1)C)C	4.3	Good	5.9
5351	n1c2c3C(CC(C)C2CC=CCCC)CCc3nc1N	5.0	Excellent	5.9
2685	O1C2C1(CC=C(C)C)C(=O)c1c(cccc1)C2=O	4.1	Excellent	5.9
1880	O1C(CCC1C([NH+]=[CH-])(C)C)(C)C1C2C(CCC(=C2)C)C([NH+]=[CH-])(CC1)C	5.0	Good	5.9
1705	O(C)C=1C(=O)C(=CC(=O)C=1OC)CC=C(CCC=C(C)C)C	4.1	Good	5.9
1432	O1C2C3C(C1CC(=CCCC2(OC(=O)CCC)C)C)C(=C)C(OC(=O)C)C(OC(=O)C)C3C(C)C	4.9	Good	5.9
780	O(C)C1=NC(=Cc2ccccc2)C(=O)NC1=Cc1ccccc1	5.8	Bad	5.9
461	O1C(OC(=O)C)C2C(CCC(C3(CC(CCC3)(C)C)C)C)C2=CC)C1OC(=O)C	4.9	OK	5.9
14370	OC(=O)C1C2(C(CC=C1C=O)C(CCC2)(C)C)C	6.1	Excellent	5.9
13734	O(C(=O)C1C2(C(CCC1=C)C(C(O)=O)(C)C(CC2)C(C=O)(C)C)C)C	5.5	Excellent	5.9
13082	O1C2CCC(O)(C3C(C3)C(CCC12C)(CCC(OO)C(C)=C)C)C	4.6	OK	5.9
12014	O1C2C(C3(C(=CC(O)CC3C)CC2)C)C(C)C1=O	4.9	Excellent	5.9
11179	O(C(=O)C)C=CC(=CCC1C(CCCC1=C)(C)C)C=O	6.0	Poor	5.9
11137	BrC1CCC(O)(CC1(C)C)CC1C2C(CC1)(C)C(Br)CCC2(O)C	4.7	Good	5.9
10829	O(C)c1c2c(ccc1)C(=O)c1c(C2=O)c(OC)ccc1	3.8	OK	5.9
10812	OC1C2C(C=CC2(O)C)C(CCC1C(CCC=C(C)C)C)=C	4.6	Poor	5.9
9530	O(C)C1=CC(=NC1=Cc1[nH]c(cc1)CCCCCCCCCCC)c1[nH]ccc1	3.6	Poor	5.9
7294	O(C(=O)C)C1CC2C(C3(C)C1(O)CC=CC3=O)C(O)CC1(C2CCC1C(C=CC(C(C)C)C)C)C	6.6	Poor	5.9
5210	O1OC(OC)(CC(C)C1CC(O)=O)CCCC=CC(=CC)C	6.0	Good	5.9
4885	O1C(O)C(=CC1=O)CCC=C(CCC=C(C)C)C	4.1	Bad	5.9

3575	OC1=C(C)C(=O)c2c(C1=O)c(ncc2)COC(=O)C	4.4	Good	5.9
2882	O1C(=O)C(=C2CC=3C(CC12O)(CCCC=3C)C)C	4.9	Excellent	5.9
1858	o1cc(cc1)CCCC(=CC(=O)C=C(CCCc1ccoc1)C)C	4.0	Poor	5.9
191	o1cc(cc1)CCCC(CC(=O)C=C(CCCc1ccoc1)C)C	5.0	Poor	5.9
13442	O1C2C3C(CCC2C(C)C1=O)(C)C(O)CCC3=C	5.4	Good	5.9
13386	OC(=O)CC1C(CCC2CC(CCC12C)(C=C)C)(C(=O)C)C	4.9	Excellent	5.9
13340	OC1(CCC(C)(C1=C)c1cc(O)c(cc1)C)C	4.6	Excellent	5.9
12843	O1C(CCC(=O)C=CC=CC)(C)C(OC(=O)C)=C(C)C1=O	4.9	Good	5.9
11564	O1C2=C(CC13C1(C(CCC3C)C3(C(CC1)C(C)(C)C(O)CC3)C)C)C(=O)C(=O)C=C2C	5.5	Good	5.9
10729	O1C(=O)C(C2C3(C(CCC12O)=CCCC3C)C)C	4.7	Good	5.9
10611	O1C23C(O)C(CCC(=O)C(C)C)(C)C1(O)CCC2(CCCC3=C)C	4.5	Good	5.9
9489	O1C2OC(=O)CC12CCC=C(CC(O)CC(CCCc1ccoc1)C)C	5.3	Excellent	5.9
9173	O(C(=O)C)C1C(C2C(CC1=O)(C1C(CC2OC(=O)C)=CC(CC1)(C=C)C)C)(C)C	5.2	Good	5.9
9136	O(C(=O)C)C1CC2C(C3CC(O)C(CC13C)C(=O)C)(CCC1C(CCCC12C)(C)C)C	5.0	Poor	5.9
5159	O1c2c(c(cc(O)c2)C)C(=O)c2c1cc(O)cc2OC	5.3	Excellent	5.9
4135	S(CC1N2C3(C(CCCC3)C(=O)CC2CCCCCC)CC1)C#N	4.8	Excellent	5.9
4134	O=C1CC(N2C3(C1CCCC3)CCC2COC(=O)C)CCCCCC	4.4	Good	5.9
2412	OC12CC3(C(CCC1(CCCC2=C)CO)=C(CC3)C(C)C)C	4.6	Good	5.9
1160	O(C(=O)C)C1CC2C(C)(C(C=O)C1C(=O)C)C(=O)CC1C2(CCC2C(CCCC12C)(C)C)C	5.6	Poor	5.9
1132	O(C)c1cc(c2c(C(=O)C(C)(C)C2=O)c1C)C(C)C	4.7	Excellent	5.9
13739	O1C(=O)C(=CC1=CC1(OOC(CC1)C(=CCO)C)C)C	3.4	Excellent	5.9
13736	O1C(=O)C(=CC1=CC1(OOC(CC1)C(=CCO)C)C)C	3.6	Excellent	5.9
12155	O1c2c(CC13C1(C(CCC3C)C3(C(CC1)C(C)(C)C(O)CC3)C)C)cc(O)cc2C	4.2	Bad	5.9
11571	O(C(=O)C)C=C(C(O)CC=C(C=CC=C(C)C)C)C=CO	5.3	Poor	5.9
6122	O(C(C(O)(C)C)CC=C(C)C1CC(C(C)=C)C(CC1)(C=C)C)C(=O)C	4.3	Poor	5.9
5053	O1C(O)C(=CC1=O)C1OCC2(O)C(C1)C1(C(CC2)C2(C(CC1)C(CCC2)(C)C)C)C	4.6	Bad	5.9
5050	O1C(O)C(=CC1=O)C1OCC2(O)C(C1)C1(C(CC2)C2(C(CC1)C(CCC2)(C)C)C)C	4.6	Bad	5.9
4090	Clc1cc[nH]c1-c1oc(cn1)-c1ccc(O)cc1	5.0	Excellent	5.9
3092	Brc1cc2[nH]c3c(c2cc1)ccnc3C(N)CC(C)C	3.7	Poor	5.9
14289	BrC1CCC(O)(C2C3C4(CC(O)C3CC4C(C)C)CCC12C)C	5.5	Good	5.8
13733	OC1C2(C3CCC(=C)C(C(OC)=O)C3(CCC2C(CC1O)(C)C)C)C	5.4	OK	5.8
12736	Brc1cc(OC)c2[nH]ccc2c1-c1c2cc(Br)ccc2[nH]c1Br	5.2	Excellent	5.8
11450	O(C)c1cc(cc(OC)c1O)Cc1n(C)c(nc1Cc1ccc(OC)cc1)N	5.1	Excellent	5.8
10907	O1c2c(cc(O)cc2)CC2C3(C(CCC12C)C1(C(CC3)C(CCC1)(C)C)CO)C	5.0	Bad	5.8
9509	O(C)c1cc(O)c2c(c1)C(=O)c1c(C2=O)c(O)cc(c1)C	6.4	Poor	5.8
7900	BrC1C2C(CCC2(O)C)C(C)(C)C1C(=C)C1CC(Br)C(O)(CC1)C	4.7	Good	5.8
4719	O1C(CCC1=O)C=CC=C1C(OC(=O)C)(C=CC1=O)C=CCCCCCC	6.6	Good	5.8
2630	Brc1cc(cc(Br)c1O)C=C1OC(=O)C=C1c1ccc(O)cc1	5.0	OK	5.8
12538	O1C(C2(C3C2(C=C(C)C3C(=CC(C(=O)CC)C)C)C)C)=C(C)C(=O)C(C)=C1OC	5.7	OK	5.8
12150	O1c2c(cc(O)cc2C)CC2C3(C(CCC12C)C1(C(CC3)C(C)(C)C(O)CC1)C)C	4.1	Poor	5.8
12128	O(C)c1c(O)c(CC=C(CCC=C(C)C)C)c(C)c(O)c1OC	4.8	OK	5.8
11512	S(CC=1CCC2C(C=1)Cc1c(occ1)C2(C)C(=O)C	4.5	Poor	5.8
10837	O1C2CCC(=CC(O)C(CCC(=CCCC12C)C)C(C)=C)CO	4.4	Poor	5.8
10365	OC1C2C(CCC2C(O)(C)C)(C=CC(CCC=C(C1)C)C)C	4.0	Excellent	5.8
8104	O1CC2C(CCC(=CCCC2=C)C)C(CC=CC(O)(C)C)C1=O	3.6	Excellent	5.8
6746	Oc1ccc(cc1)CC(NC(=O)C=CC(=CC(CCCCCC)C)C)C(OC)=O	5.0	Poor	5.8
6386	O1C2(C3C(CC(C)C2(CC1C1(OOC(CC1)C(C(O)=O)C)C)C)C=C(CC3)C)C	4.6	Bad	5.8
6385	O1C2(C3C(CC(C)C2(CC1C1(OOC(CC1)C(C(O)=O)C)C)C)C=C(CC3)C)C	3.6	Bad	5.8
6212	O1C(C2CCC3(C(CCc4c3coc4)C2(CC1=O)C)C)(CO)C	5.1	Good	5.8
5962	OC1C2C(C(C=CC=CC(O)=O)C(C(=CC)C)C(=C2)C)C(CC1)C	4.5	Poor	5.8

5354	N1C=2C3C(CC(C)C=2C=CCC)CCC3N=C1N	4.2	Excellent	5.8
4684	OC1C2C(C(C=CC=CC(O)=O)C(C(=CC)C)C(=C2)C)C(CC1)C	4.5	Bad	5.8
4100	OC1(C2CCC(=CCCC(C=CC2(CC1)C)C)C)C(O)(C)C	4.6	Excellent	5.8
3780	O1C2C3C(C1CC(O)(C)C(OC)CCC2(OC(=O)C)C)C(OC(=O)C)(CCC3C(C)C)C	5.2	Poor	5.8
3752	S1(=O)(=O)C2=C(NCC1)C(=O)c1c(cc3c(c1)C1(C(=COC3=O)C(=CCC1)C)C)C2=O	5.9	OK	5.8
3012	o1c2c(CCCC2(C=CC=C(CCCC(CC(O)=O)=C)C)C)cc1	4.8	Excellent	5.8
2358	O1C(C(=O)C(C)C2OC(=O)CC(O)C(CC2)C)(C)C1CC(O)C(C)C	5.3	Excellent	5.8
14332	O=C1C23C(CCC2(C=C(C(O)=O)C3C)C)C(C1)C	5.3	Good	5.8
13794	O1C(=CC(OC)=C(CO)C1=O)C1C2C(CCCC2)C=CC1C	5.4	OK	5.8
13079	O1C2CCC(O)(C3C(C3)C(CCC12C)(CC=CC(O)(C)C)C)C	5.2	Good	5.8
12971	O(C=C1C2(C(CC=C1C=O)C(CCC2)(C)C)C)C(=O)C	4.0	Excellent	5.8
12769	O(C(=O)C)C1C2(C=O)C(CCC1(CC=C2C=O)C)C(CCC=C(C)C)C	5.0	OK	5.8
12166	OC12CC3(C(CCC1(CCC(O)C2=C)C)=C(CC3)C(C)C)C	4.5	Good	5.8
11394	Oc1ccccc1[NH+]1C=Cc2c([nH]c3c2cccc3)[CH-]1	4.1	Poor	5.8
9798	BrC12OC3C(CC(Br)C(Cl)(C3)C)(C1(C)C)C(=C)C(O)C2O	4.2	Excellent	5.8
9321	ClC=C1CCNc2c1cc(OC1OCC(OC)C(O)C1OC)cc2C	4.4	Excellent	5.8
8146	O1c2c(C(=O)C=C1CCC)c(O)cc1c2c(OC)cc(O)c1	5.7	Excellent	5.8
7564	O([N+](=O)[O-])C1c2c(CC1(C)C)c(C)c(CCO)c(c2)C	4.0	Excellent	5.8
6851	O(C(=O)c1ccccc1)CC(=O)c1c2c([nH]c1)cccc2	5.0	Bad	5.8
6766	O1C(=O)C(CCC=CC=CN(C)C(=O)C(CCC(CC1C(C)(C)C)C)C)C	4.9	Good	5.8
6252	O1C(CC2C(CCC3C4(C(CCC23C)C(CCC4)(C)C)C)C1OC(=O)C)C1=CC(OC1O)=O	5.1	Bad	5.8
6043	O1C2(C(C=C3C(=COC(=C3)C)C2O)=C(C(=O)CCCCCC(O)C)C1=O)C	5.8	Poor	5.8
5566	O(C(=O)C)C1CC2C(C3CC(O)C(=CC13C)C=O)(CCC1C(CCCC12C)(C)C)C	4.6	Good	5.8
5296	O1C(CCCCCC1=O)C1CC1C(O)C=CC(O)CC=CCC=CCC	4.9	Poor	5.8
4270	O1C(CCC1C([NH+]=[CH-])(C)C)(C)C1C2C(CCC(=C2)C)C([NH+]=[CH-])(CC1)C	5.0	Good	5.8
2416	O1C23C1C(O)C=1C4CCC(C(CCCC(C)C)C)C4(CCC=1C2(CCC(O)C3)C)C	5.4	OK	5.8
2285	O1CC(C2C(C3(O)CC(CC2)(C)C(O)CC3)C1=O)=CC=CC(C)C	3.6	OK	5.8
1163	O1C2CC(=CCCC(=O)C(C2C(CCC=C(C)C)C)C1=O)C	4.3	Poor	5.8
770	O1c2c(c(O)c(OC)c3c2c(OC)cc(O)c3)C(=O)C=C1CCC	5.8	Excellent	5.8
251	O1C23CC(CCC(=O)C(C)C)(C)C1(O)C(O)CC2(CCCC3=C)C	5.8	Poor	5.8
177	Brc1cc(O)c2[nH]c3c(c2c1)ccnc3C1=NCCC1	3.9	Excellent	5.8
14471	O1CC2C(CCC3C2(C)C(OC(=O)C)CC2C3(CCC3C(CCCC23C)(CC)C)C)C1(O)C	3.8	OK	5.8
14440	OC1C2C3C(CCC2(CC(=O)C(=C1)CO)C)(CCC3C(C)=C)C	5.0	Excellent	5.8
13447	O(C(=O)C=CC(O)(C)C)C1C=C(CCC(=O)C(CCC1C(=O)C)=C)C	5.6	Good	5.8
13139	Brc1cc2[nH]cc(c2cc1)C=C1N(C)C(=N)N(C)C1=O	4.2	Excellent	5.8
12784	O=C1CC2C3(C(CCC2(C2CC=C(C(=O)C)C(C=O)C12C)C)C(CCC3)(C)C)C(O)=O	5.2	Bad	5.8
12525	OCC=C(CCC(=O)C(CCC=C(CC(=O)C=C(C)C)C)=C)C	5.2	Poor	5.8
11776	OC1C2CC(O)CCC2(C=2C(C3CCC(=C(CC(=O)C=C(C)C)C)C3(CC=2)C)C1)C	5.0	Bad	5.8
11775	OC1C2CC(O)CCC2(C=2C(C3CCC(=C(CC(=O)C=C(C)C)C)C3(CC=2)C)C1)C	5.5	Poor	5.8
10394	O(C(=O)C)C1C2C(CC=C2C)C(CCC1C(CCC(O)C(O)(C)C)C)=C	4.0	Excellent	5.8
9666	N1C=2C(N=C1N)=C(C1=NC(=NC1=CC=2)N(C)C)C	3.6	Excellent	5.8
9492	O1C(O)C(=CC1=O)CCCC(CC(O)CC(=CCCc1ccoc1)C)C	5.4	OK	5.8
9491	O1C(=O)C(=CC1O)CCC=C(CC(O)CC(CCCc1ccoc1)C)C	4.7	Poor	5.8
8266	O1C(=O)C(=C2CC3(C(CC12O)C(=O)CCC3C)C)C	5.4	Excellent	5.8
7225	O=C1CC2C3C(CCC2(C)C1C(O)(CCCC(C)C)C)C1(C(CC(=O)CC1)CC3)C	3.8	Bad	5.8
5736	OC1(c2nc(nc3CCC(CC1C)c23)N)CCCC	4.8	Good	5.8
5413	O1C23C(C=C(C)C1C=CCCCCCCC)C1CC(O)CC1(OC2=O)CC3O	3.3	Bad	5.8

5297	O1C(CC=CCCC1=O)C1CC1C(O)C=CC(O)CC=CCCCCC	5.1	OK	5.8
4873	O1C(O)(C)C(C)C(O)CC1CC=CC=CC(CC(=CCCC=C)C)C	3.7	Bad	5.8
3535	BrC1C(C)C2(C3(C(CC3OC(=O)C)(C)C(Br)C2)C(C)C1O)C	4.7	Excellent	5.8
2577	OC1C2C(CCC3C1(CCC3(O)C(C)C)C)(C=CC=C2C)C	4.5	Excellent	5.8
2183	O1OC(C=C(CC)C1CC(O)=O)(CC(CC(CC)C=CCC)C)CC	4.9	Good	5.8
1420	Brc1cc(cc(Br)c1OCC1OC(=O)NC1)C1OC(=O)NC1	4.8	Excellent	5.8
30	ClC1=CC(O)(CC=CCCCCC)C(=CC=CCCCC(OC)=O)C1=O	5.3	OK	5.8
13525	OC(CC(=O)CC(=CCCC(=CCCC(=CCO)C)C)C)(C)C	4.7	Excellent	5.8
12562	O1C(C2C3(CCC4C(=CCC5C(C)(C)C(O)CCC45C)C3(CC2=O)C)C1=O)(CCCC(C)=C)C	4.5	Bad	5.8
12140	O(C)c1ccc(cc1O)CCNC(=O)C=C(CCCCCCCCC)C	5.1	Bad	5.8
10390	o1cc(cc1)CCCC(C(O)CC=C(CCCc1ccoc1)C)C	5.1	OK	5.8
10307	O(C)c1c2NC(=O)CN3C=Cc4nccc(c1)c4c23	5.1	Excellent	5.8
9027	o1cc(cc1)CCCC(=CC=CC(CCC=C(CC(O)C(O)=O)C)C)C	4.7	Bad	5.8
6787	O1C2(CCC1C1(CCC3OC(C)(C)C(O)CCC13C)C)C1(C(OC(C)(C)C(=O)CC1)CCC2C)C	4.4	Bad	5.8
4282	O1C2CC(C=C)(C)C(CC2=C(C)C1=O)C(C(OC)=O)=C	5.5	Excellent	5.8
3861	O1C(C(C(OC(=O)CC)C(C(=O)CC)C)C)C(C)C(=O)C(C)=C1CC	5.4	Excellent	5.8
3522	O1C2OC=C(C3C2C(CC3)C1OC(=O)C)C(=O)CCC(C)C	5.9	Good	5.8
2734	O1c2c(C=C(OC)C1=O)cc1C=C(OCc1c2O)C(=CC(CC)C)C	4.2	Poor	5.8
1951	S(CC=1CCC2C(C=1)Cc1occc1C2(C)C)C(=O)C	4.4	Excellent	5.8
535	O(C(=O)C)C1CC2C3(C(CCC2(C2CC=C(C(=O)C)C(C=O)C12C)C)C(CCC3)(C)C)C=O	5.4	OK	5.8
25	ClC1=CC(O)(CC=CCCCCC)C(=CC=CCCCC(OC)=O)C1=O	6.6	Good	5.8
13738	O1C(=O)C(=CC1=CC1(OOC(CC1)C(=CCO)C)C)C	4.9	Excellent	5.8
10905	O1C2C(CCC(=C)C(O)CCC(=C2)C)C(=C)C1=O	4.6	Excellent	5.8
10548	BrC1CCC(=C)C(Cc2cc(O)c(Br)cc2O)C1(C)C	5.2	Excellent	5.8
10324	OC1CC(=O)C(CCC(=O)CCCCC)C1CC=CCCCC(O)=O	6.8	Excellent	5.8
9875	BrC1CCC(CC1(C)C)=CC1C2C(CC1O)(C)C(Br)CCC2(O)C	5.0	Good	5.8
9298	O=C1C(=C(O)CCCC(CCCCCCC)C)C(=O)N(C)C1C	4.5	Excellent	5.8
9145	O1CC(C2C(C(CC(OO)CC(CC2)=C)=C)C1=O)=CC=CC(O)(C)C	5.8	Poor	5.8
8616	O1OC(CC(CC)C1CC(OC)=O)(CC)C=CCC(C)C	5.0	Good	5.8
8283	OC1(CCC=C(CCC=C(CCC(O)C=C1)C)C)C	4.1	Excellent	5.8
8101	O(C(C=CC(OC(=O)C)C(C=CC=C(C=C)C)C)(C)C)C(=O)C	5.4	Good	5.8
7081	O(C)c1cc(OC)ccc1C(=O)C=Cc1ccc(OC)cc1OC	4.4	Poor	5.8
5759	o1cc(cc1)CCC=C(CC(=O)CC(=CCCc1ccoc1)C)C	5.7	OK	5.8
4016	S1CC(N=C1C1CC1C)C=CCCC=CC=C(CCC(OC)CC=C)C	4.3	Bad	5.8
3921	O1CC=2C(C3(C(CC=2)C2(C(CC3OC(=O)C)C3(C(CC2)C(C)(C)C(=O)CC3)C)C)C)C1O	4.0	Poor	5.8
3199	O1C23C1C(OC(=O)C)CC(C)C2(C)C(C(=O)C)C(=O)CC3	4.6	Good	5.8
1797	O1c2c(cc(O)cc2)CC2C3(C(CCC12C)C1(C(CC3)C(CCC1)(CO)C)C)C	4.1	Bad	5.8
861	ClC1C2C(CCC1(O)C)C([NH+]=[CH-])(CCC2C1(OC(CC1)C([NH+]=[CH-])(C)C)C)C	4.2	Poor	5.8
29	ClC1=CC(O)(CC=CCCCCC)C(=CC=CCCCC(OC)=O)C1=O	4.7	OK	5.8
8519	IC1=CC(O)(CC=CCCCCC)C(=CC=CCCCC(OC)=O)C1=O	4.2	Good	5.8
7399	o1cc(cc1)CCCC(O)(CC(O)C=C(CC=Cc1ccoc1)C)C	5.7	Poor	5.8
6394	ClC(Cl)(Cl)C(CC1N(C)C(=O)C(N(C)C1=O)=CC(C(Cl)(Cl)Cl)C)CO	4.9	OK	5.8
13684	OC1C2C(CC=C2C)C(CCC1C(CCC(O)C(C)=C)C)=C	4.7	Good	5.7
12615	Oc1cc(ccc1O)C=CC(OCC1C2(C(CCC1=C)C(CCC2)(C)C)C)=O	4.9	Bad	5.7
11615	O(C(C(O)(C)C)CC=C(COC(=O)C)C1CC(C(C)=C)C(CC1)(C=C)C)C(=O)C	5.9	Good	5.7
10677	O(C(=O)C)C1C2C(CCC2C(OC(=O)C)(C)C)(C=CC(CC=CC(O)(C1)C)C)C	4.2	Excellent	5.7
8224	O1C(=O)C2C(=CCC3C(C)(C)C(O)CCC23C)C1=O	4.6	Excellent	5.7

8093	O(C)c1c2c(cc(OC)c1)C(=O)c1c(C2=O)c(O)cc(c1)CO	5.2	Excellent	5.7
7293	OC(=O)C1(C2CCC3(C(CCC(=C3)C=O)C2(CCC1)C)C)C	5.5	Excellent	5.7
7269	O1C2OC(=O)CC12CCC=C(CCC=C(CC(O)C=C(C)C)C)C	5.5	OK	5.7
5820	O1C2(OC)C(=CC1=O)C(C1CC(=O)C(=CC1C2)C)(C)C	5.0	Excellent	5.7
5667	O(C(=O)C)C1CC2C=3C(C(C=C(C)C)C(=O)C2C)C(CCC=3C1C)=C	4.4	Good	5.7
5502	O1C23CC(OC(=O)C)CCC2(C)C(=O)C(=CC13)C1CCC(C(C=CCC(C)C)C)C1(CC=O)C	6.3	Poor	5.7
4711	O1C(CCC1=O)C=CC=C1C(OC(=O)C)(C=CC1=O)C=CCCCCCC	5.0	Good	5.7
3748	OC1CC2C(C3CCC(C(CCC(OC)=O)C)C13C)C(O)CC1CC(=O)CCC12C	3.3	Bad	5.7
3003	O(C(=O)CO)C1CC2C(NC1C)CCCC2C=CC=CCCCC	3.8	OK	5.7
654	O(C)c1cc2c(c(CCC)c1C(OC)=O)c(O)c1c(c2)C(C)(C)C(O)=C(C)C1=O	4.7	Good	5.7
14481	O1C(OC)C2=C(C3C(C3)C(CC(OC(=O)C)C2C(CCC=C(C)C)C)C)C1OC	4.9	OK	5.7
13774	Brc1cc2c3c([nH]c2cc1)c(ncc3)C1=NCCC1	4.8	Excellent	5.7
12439	o1cc(cc1)COC(=O)C=C(CCC=C(CC(=O)CC(C)C)C)C	4.1	Bad	5.7
12397	O1C23CC(CCC(=O)C(C)C)(C)C1(O)CCC2(CCC(O)C3=C)C	4.9	Good	5.7
12373	O(C)c1c2c(cc(c1)C(=O)CC)C(=O)c1c(C2=O)c(OC)cc(OC)c1	5.3	Poor	5.7
11956	OC(C(O)CCC1(C2C(C1)C(CCC=C(CC2)C)=C)C)(C)C	5.0	Excellent	5.7
11651	O=C1NCCC=C1CC(=O)c1c2c([nH]c1)cccc2	4.6	Excellent	5.7
11382	Brc1cc([NH+]2C=Cc3c([nH]c4c3cccc4)[CH-]2)c(O)cc1	4.5	Good	5.7
10415	O(C)C=1C(=O)C(CC=C(CCC=C(C)C)C)=C(C)C(=O)C=1OC	3.9	Good	5.7
9498	O1c2c(c(O)c(OC)c(O)c2)C(=O)C=C1c1ccc(OC)cc1	6.0	Good	5.7
8863	OC1(C=CC(=O)c2c1n(O)c1c2cccc1C(OC)=O)C=O	5.0	Excellent	5.7
8294	BrC12OC3C(CC(Br)C(Cl)(C3)C)(C1(C)C)C1(OC1C2O)C	5.6	Excellent	5.7
8103	O1CC2C(CCC(=CCCC2=C)C)C(CCC=C(CO)C)C1=O	4.9	Poor	5.7
7902	O(C(=O)c1c2nc3c(nc2ccc1)cccc3)C	5.1	OK	5.7
7787	O1CC2C(CCC(=CC(O)CC2=C)C)C1(C=CC=C(C)C)CO	4.5	Poor	5.7
7568	ClCC1OC(=O)c2c1c(cc1c2CC(C)(C)C1O)C	5.0	Excellent	5.7
7487	O(C(=O)C)C1CCC2(C(CCC3(C2CC(=O)C3=C(C(OC)=O)C)C)C1(C(OC)=O)C)C	5.3	Poor	5.7
5419	OC(CCCCC(O)=O)C=CC=1CC2C(CC=CC2)C=1CCCCC	4.4	OK	5.7
4271	O1C(CCC1C([NH+]=[CH-])(C)C)(C)C1C2C(CCC(=C2)C)C([NH+]=[CH-])(CC1)C	4.0	Good	5.7
2332	O(C)c1cc(CC=C(CC(=O)CCCC(=O)C=C(C)C)C)c(O)c(c1)C	5.9	Excellent	5.7
2146	O1C(=CC(OC)=CC1=O)C(NC(=O)C)Cc1ccccc1	5.1	OK	5.7
13821	O(C(=O)C1C2(C(CC=C1C)C(CCC2)(C)C)C)CC(O)CO	4.8	Good	5.7
13775	Brc1cc2c3c([nH]c2cc1O)c(ncc3)C1=NCCC1	4.3	Excellent	5.7
13639	O1C(C)C(O)C(OC(=O)C)C(O)C1OC1CC2CCC3C4CCC(C=C)C4(CCC3C2(CC1)C)C	4.2	Bad	5.7
11885	O1CC2C(C1(O)C)C(OC(=O)C)CC1C2(C)C(=O)CC2C1(CCC1C(CCCC12C)(CC)C)C	4.0	Bad	5.7
11630	OC1C2CC(O)CCC2(C=2C(C1)C1=C(CC=2)C(CC1)(C(CC(=O)CC(C)C)C)C)C	3.5	Excellent	5.7
10780	O=C(Cc1ccccc1)C(=O)Nc1ccccc1	5.9	Excellent	5.7
10661	OC1C2C(CC=C2C)C(O)(CCC1C(CCC=C(C)C)C)C	4.7	Good	5.7
9812	N1C=2C(N=C1N(C)C)=C(C1=NC(=NC1=CC=2)N(C)C)C	6.1	Good	5.7
9722	Brc1cc(cc(Br)c1OCC1OC(=O)NC1)C1OC(=O)NC1	5.3	Excellent	5.7
8284	O1C2CCC(O)(C3C(C=CCCC12C)C(O)(CC3)C)C	4.5	Excellent	5.7
7839	O(C)C1=CC(=O)C=C(CC=C(CCC=C(C)C)C)C1=O	5.0	Excellent	5.7
6789	O1C2CCC3(C(CCC3=O)C2(CCC(O)C1(C)C)C)C	4.4	OK	5.7
6130	O1OC(CCC1(CCC1C(CCCC1=C)(C)C)C)C(C(OC)=O)C	4.7	Good	5.7
5336	O1CC(C2C(C(CCC=C(CC2)C)=C)C1=O)=CC=CC(O)(C)C	5.9	OK	5.7
4089	Clc1c([nH]cc1Cl)-c1oc(cn1)-c1ccc(O)cc1	4.2	Bad	5.7
3452	O(C(=O)CCCCC=CCC(=CC=CC=CC(=O)CC)C=O)C	5.7	Good	5.7

3207	O(C(=O)C)C1C2C(CCCC2(C)C)(C)C(CCC(=CCO)C)=C(C)C1O	3.6	OK	5.7
3059	O(C(C(C=COC(=O)C)=COC(=O)C)CC=C(C#CC=C(C=O)C)C)C(=O)C	5.9	Bad	5.7
2425	OC12CC(O)CCC1(C1C(C3CCC(C(CCC(C(OC(=O)C)(C)C)C)C)C3(CC1)C)CC2=O)C	3.7	Bad	5.7
2389	O=C1C23C(C(C)C(=CC2C=CCC(C=C(CC(=O)C=C1)C)C)C)C(NC3=O)Cc1c2c([nH]c1)cccc2	3.8	Bad	5.7
2237	O1C2(c3c(CC(C)C2(CC1C1(OOC(CC1)C(C(OC)=O)C)C)C)cc(cc3)C)C	5.0	Bad	5.7
1731	OC1CC(=CCCC(C2C(C=C1C(C)C)C(O)(CC2)C)=C)C	4.8	Excellent	5.7
1587	S(CC=1CCC2C(CC3(OC(=O)C=C3C2(C)C)O)C=1)C(=O)C	4.5	Good	5.7
1434	O1CC12CCCC1C(CCC(CC(O)=O)C)(C)C(CCC12C)C	5.6	Excellent	5.7
13903	O1C(O)(C)C(=O)C(C)=C1C(=CC(CC(CC(CC)C)C)C)C	5.1	Good	5.7
13350	O1CC(C2C(C34CCC(OC3)C4(CC2)C)C1=O)=CC=CC(O)(C)C	5.1	Excellent	5.7
13253	O1OC(CC(CC)C1CC(O)=O)(CCCC(CC)C=CC(=O)C)CC	5.7	Good	5.7
12846	O1C2C3C(CCC2C(C)C1=O)(C)C(O)CCC3=C	4.7	Good	5.7
12438	O1C=CC(=C)C1OC(=O)C=C(CCC=C(CC(=O)CC(C)C)C)C	4.1	Bad	5.7
12051	O=C1C=CC(CCC(=O)CCCCC)C1CC=CCCCC(O)=O	5.1	Good	5.7
11643	n1c2c(n(C)c1N)C(=C1N=C(N=C1C=C2)NC)C	4.4	Excellent	5.7
11612	O(C(=O)C)CC(=CCC(O)C(O)(C)C)C1CC(C(C)=C)C(CC1)(C=C)C	4.6	OK	5.7
10662	OC1C2C(C(CCC1C(CCC=C(C)C)C)=C)C(O)C=C2C	4.2	OK	5.7
9604	S(OC1CC2=CC(=O)C3C4CCC(C(CCCC(C)C)C)C4(CCC3C2(CC1)C)C)(O)(=O)=O	5.8	Bad	5.7
6317	Brc1cc2c3c([nH]c2cc1Br)c(ncc3)C1=NCCC1	4.2	Bad	5.7
6190	O(C(=O)C)c1ccc(cc1)CCC=CC=CC=CCCOC(=O)C	5.4	Bad	5.7
3093	Brc1cc2[nH]c3c(c2cc1)ccnc3C(=O)CC(C)C	4.7	Bad	5.7
2728	OC1C2CC(O)CCC2(C)C(=O)C(=C1)C1CCC(C(CCCC(C)C)C)C1(CC=O)C	4.5	Good	5.7
2117	O=C1c2c(cccc2)C(=O)C1=CNN(C(OCC)=O)C	4.2	Excellent	5.7
1934	O1C(O)(C)C(=O)C(C)=C1C(=CC(CC(CC(CCC)C)C)C)C	4.1	Excellent	5.7
709	S1CC(N)C2N(OC1)CCc1c2[nH]c2c1cccc2	4.0	OK	5.7
14316	O(C)C1=C(C)C(=O)c2c(C1=O)c(ncc2)COC(=O)C	4.8	Poor	5.7
13932	OC1CC2(C(CC=C(CCC=C1CO)C)C(CC2)C(C)=C)C	5.1	Good	5.7
13625	O1C(=O)C(C)=C(O)C(C)=C1C(=CC(=CC(CC(CCC)C)C)C)C	4.6	OK	5.7
12970	O1C2(CC(=O)C(=CC=CC(=O)C)C)C(CC(O)CC12C)(C)C	5.7	Poor	5.7
12722	O1C(O)C2=C(CCC3C(CCCC23C)(C)C)C1=O	4.8	Poor	5.7
11381	Brc1cc([NH+]2C=Cc3c([nH]c4cc(Br)ccc34)[CH-]2)c(cc1)C(OC)=O	3.6	OK	5.7
10603	ClC1C2OC(C(C1)C2C=CCC=CCCCCC(O)=O)CC	5.4	Bad	5.7
10376	O(C(=O)C)c1c2nc3c(nc2ccc1)cccc3	5.4	Good	5.7
8290	O1C(O)(C2=C(C3(C(CC2)C2(C(CC3O)C3(C(CC2)C(CCC3)(CC)C)C)C)C)C1=O)C	3.7	Good	5.7
5051	O1C(O)C(=CC1=O)C1OCC2(O)C(C1)C1(C(CC2)C2(C(CC1)C(CCC2)(C)C)C)C	4.2	Bad	5.7
4592	O1C2CCC(C)C(O)(CCC3C4(C(OC(C)(C)C(=O)CC4)CCC3=C)C)C2(CCC(=O)C1(C)C)C	4.1	Bad	5.7
2380	O1C2OCC(OC(=O)C)C(C2C2(C3C(CC2)C(CCCC3=C)(C)C)C)C1OC(=O)CC(C)C	5.6	Bad	5.7
518	OC1(C2C(C=C(CC1)C(CC(=O)CC(C)C)C)C(O)(CC2)C)C	5.5	Excellent	5.7
224	O1C(=O)C(=CC1O)CC1C=C(CCC1C(C)=C)C	6.0	Excellent	5.7
13701	N1C=2C3C(CC(C)C=2CCCC)CCC3N=C1N	3.8	Poor	5.7
13047	O=C1N(CCCCN=C(N)N)C(CC1)=CC=CCC=CCC	5.1	OK	5.7
12726	OC(C(O)CCC(=CCCC(=CCCC(=O)C)C)C)(C)C	3.7	Good	5.7
9884	O=C1CCC(C=CC(O)CCCCC)=C1CC=CCCCC(OC)=O	5.1	Good	5.7
9720	O(C(C(CCC(=CC=O)C)CC=C(CCC(O)C(C)=C)C)(C)C)C(=O)C	4.6	Bad	5.7
9432	S(OC1CC2C(C=3C(C4CCC(C(=O)C)C4(CC=3)C)CC2O)(CC1)C)(O)(=O)=O	5.5	Poor	5.7
9039	Brc1cc(cc(Br)c1OCC[C-]([N+](C)(C)C)C(O)=O)CCNC(=O)C	4.4	Bad	5.7

8228	O1C(OCC)C2=C(C3(C(CC2O)C(CCC3)(C)C)C)C1=O	5.0	Excellent	5.7
7600	S(OC1CC2C(C=3C(C4CCC(C(=O)C)C4(CC=3)C)CC2O)(CC1)C)(O)(=O)=O	4.5	Bad	5.7
7305	O1C(C=2C(C3(C(CC=2)C2(C(CC3O)C3(C(CC2)C(CCC3)(C)C)COC(=O)C)C)C)C1O)C	5.1	Bad	5.7
6004	OC(CC=CCC)C=CC=CCC=CCCCCC(O)=O	5.4	Bad	5.7
5936	O1C(O)C2(O)C(C3(C(CC2)C(CCC3)(C)C)C)C1OC	4.6	Excellent	5.7
5794	O=C1C(=CC=C2CC(=O)CCC12C)C1CCC(C(CCCC(C)C)C)C1(CC=O)C	3.8	Bad	5.7
4210	O1C2CC(OC(C=CC2O)CC)C1C(O)C=CC#C	3.7	Excellent	5.7
12821	OC1(C=CC(=O)C1=CC=CCC)CCCCCCCC(O)=O	7.1	Excellent	5.7
11316	O1OC(CC(CC)C1CC(OC)=O)(CC)C=CCCC	4.5	Poor	5.7
10055	O(C(C)(C)C)C(=O)n1cc(c2c1cccc2)C=O	6.5	Excellent	5.7
9301	O=C1CN(C)C(=O)C1=C(O)CCCCCCCCCCC(CC)C	4.4	Bad	5.7
8222	O1CC=2C(C3(C(C(CCC3)(C)C)C(O)C=2)C)C1O	4.9	Excellent	5.7
6382	ClC(Cl)(Cl)C(CC1N(C)C(=O)C(N(C)C1=O)=CC(C)=C(Cl)Cl)C	4.4	OK	5.7
3250	o1ccc(C(C)C)c1CC(C(O)=O)C1CCC2C3C(CCC12C)C1(CC(=O)C(O)=CC1CC3)C	5.9	OK	5.7
14476	O1C(C2C(C3(C(CC2O)C2(C(CC3OC(=O)C)C3(C(CC2)C(CCC3)(CC)C)C)C)C)C1=O)C	4.2	Good	5.6
13080	O1C2CCC(O)(C3C(C3)C(CCC12C)(CC=CC(OO)(C)C)C)C	5.8	Good	5.6
12250	O1C(C2OC2C(=C)C2C3C4C(CCC4C)C3(C)C(O)C2)C1(C)C	5.1	Excellent	5.6
10003	O1C(=O)C(=CC1O)CCC1C(CC=CC1=C)(C)C	5.0	Excellent	5.6
5753	Brc1cc2NC(=C3Nc4c(ccc(Br)c4)C3=O)C(=O)c2cc1	4.8	Poor	5.6
5746	O1C2=CC(=O)C(NC(=O)C)=CC2=Nc2c1cccc2	4.2	Good	5.6
3523	O1C2OC=C(C3C2C(CC3)C1OC(=O)C)C(=O)CC=C(C)C	5.0	Excellent	5.6
3058	O(C(C(=CC=O)COC(=O)C)CC=C(C#CC=C(C)C)C)C(=O)C	4.6	OK	5.6
2699	O=C1C=C2CCC3C4CCC(C(O)(C=CC(C(C)C)C)C)C4(CCC3C2(C=C1)C)CO	4.8	Poor	5.6
13635	O(C(=O)C)C1CC2CCC3C4CCC(C=C)C4(CCC3C2(CC1)C=O)C	4.2	OK	5.6
13083	OC1(C2C(C2)C(CCC(=CCC1)C)(CC=CC(O)(C)C)C)C	4.4	Excellent	5.6
12018	O1C2C(C3(C(=CC(=O)CC3C)CC2)C)C(C)C1=O	5.5	Excellent	5.6
10928	S(OC1CC2=CCC3C4CCC(=O)C4(CCC3C2(CC1)C)C)(O)(=O)=O	5.5	OK	5.6
9358	O1C(CC=CC(OC(CCC(O)C=CC1=O)C)=O)C	4.3	Excellent	5.6
7914	O=C1NC(=O)N(c2nccnc12)c1ccccc1	5.1	Excellent	5.6
6606	OC1C=C(C)C2(C(C1)C(CCC(O)(C=C)C)(C)C(CC2)C)C	4.5	Poor	5.6
6605	OC1C=C(C)C2(C(C1)C(CCC(O)(C=C)C)(C)C(CC2)C)C	5.3	Excellent	5.6
6200	OC(=O)c1ccccc1N(C(=O)CCc1ccccc1)C	5.6	Excellent	5.6
6127	O1C23C(C=C(C)C1C=CCCCCCCC)C1CC(O)CC1(OC2=O)CC3O	5.2	Poor	5.6
5895	OC1(C2C(CC(CC2C)C)C=CC1C)C=CC=CC(O)=O	5.3	Excellent	5.6
5436	o1cc(cc1CC(CCCc1cc(oc1)CC(C(O)=O)C)C)CCCc1ccoc1	5.6	Good	5.6
5281	O1C(CCCCCC1=O)C1CC1C(O)C=CC(O)CC=CCCCCC	5.3	OK	5.6
3860	O1C(C(C(OC(=O)C(CC)C)C(C(=O)CC)C)C)C(C)C(=O)C(C)=C1CC	5.6	Excellent	5.6
2628	O1C(O)C(O)C(CCC=C(CCC=C(CC(=O)C=C(C)C)C)C)C1=O	4.9	Bad	5.6
1422	O(C(C)c1c2nc3c(nc2ccc1)c(ccc3)C(O)=O)C	4.6	Good	5.6
995	O1C(C=C(CCC=C(CCCC(=O)C)C)C)C(CCO)C(=C)C1=O	4.2	Good	5.6
13607	O1C2(OCC)C(=CC1=O)C(C1CC(O)C(=CC1C2)C)(C)C	4.9	Excellent	5.6
13527	OC(C(CCC=C(CCC=C(CC(=O)C=C(C)C)C)C)=C)CO	3.8	OK	5.6
13081	O1C2CCC(O)(C3C(C3)C(CCC12C)(CCC(O)C(C)=C)C)C	4.4	Excellent	5.6
12425	O1C(C2C3(CCC4C(=CCC5C(C)(C)C(O)CCC45C)C3(CC2)C)C1=O)(CC(O)CC(C)C)C	3.5	Poor	5.6
12310	O(C=O)C1CCC2=CC(O)CC(C)C2(C)C1C(=O)C	5.2	Excellent	5.6
10704	IC1=CC(O)(CC=CCCCCC)C(=CC=CCCCC(OC)=O)C1=O	5.9	Good	5.6
10545	Brc1cc(O)c(cc1O)C(=O)C=C(CCC=C(C)C)C	5.4	Excellent	5.6
10391	o1cc(cc1)CCCC(C(=O)CC=C(CCCc1ccoc1)C)C	4.3	Excellent	5.6

9939	O(C)C1=CC(=NC1=Cc1[nH]c(cc1)CCCCCCCCCCC)c1[nH]ccc1	5.1	OK	5.6
9529	O(C(=O)C1N(Cc2ccccc2)C(CC1)C(OCC)=O)CC	4.3	Excellent	5.6
8381	O(C)c1cc(OC)ccc1C=CC(=O)c1ccc(OC)cc1O	6.1	Good	5.6
8231	O1C(O)C2=C(C(O)CC3C(CCCC23C)(C)C)C1=O	5.4	Excellent	5.6
6749	O(C(=O)C)c1ccc(OC(=O)C)cc1C#CC(C)=C	4.2	Poor	5.6
4088	Clc1c([nH]c(Cl)c1Cl)-c1oc(cn1)-c1ccc(O)cc1	4.2	Bad	5.6
769	O1c2c(C(=O)C=C1CCC)c(O)c1c(cc(O)cc1OC)c2OC	5.3	Good	5.6
664	O(C)C1=CC(=O)C=C(CC=C(CCC=C(C)C)C)C1=O	4.1	Excellent	5.6
14325	O=C1C23C(CCC2(C=C(C(OC)=O)C3C)C)C(C1)C	4.2	Excellent	5.6
13690	O(Cc1ccc(cc1[N+](=O)[O-])C(OC)=O)c1ccccc1	5.9	Good	5.6
13626	O1C(=O)C(C)=C(O)C(C)=C1C(=CC(=CC(CC(CCC)C)C)C)C	5.2	Excellent	5.6
12374	O1C2(OC)C3CC(CCC2=CC1=O)(C)C(O)(C)C(C3)=C	5.3	OK	5.6
12144	O1c2c(CC13C1(C(CCC3C)C3(C(CC1)C(C)(C)C(O)CC3)C)C)cc(O)cc2C	3.9	Bad	5.6
9719	O1C=C(c2ccc(OC)cc2OC)C(=O)c2c1cc(OC)cc2O	3.4	Poor	5.6
9278	O(C)c1ccc(cc1)CC=1N(C)C(=N)N(C)C=1Cc1ccc(OC)cc1	4.5	Poor	5.6
6603	O(O)C1C=C(C)C2(C(C1)C(CCC(O)(C=C)C)(C)C(CC2)C)C	4.9	Excellent	5.6
3994	O1CC=2C(C3(C(CC=2)C2(C(CC3OC(=O)C)C3(C(CC2)C(C)(C)C(=O)CC3)C)C)C)C1O	4.8	OK	5.6
3980	O(C(=O)C)C=C(CCC=C(CCC=C(C)C)C=O)C=COC(=O)C	6.0	Bad	5.6
2092	O(C(C(CC=O)=COC(=O)C)CC=C(C#CC=C(C)C)C)C(=O)C	5.4	Poor	5.6
1935	O1C2=CC(C=C)(C)C(CC2=C(C)C1=O)C(C(OC)=O)=C	6.1	Excellent	5.6
1698	O1C(CC=CCCCC1=O)C1CC1C(O)C=CC(O)CCCCC	4.5	Poor	5.6
1053	Brc1cc2[nH]c3C4N(OCS(=O)CC4N)CCc3c2cc1	3.7	Poor	5.6
13605	O=CC1(CC1C=C(CCC=C(C)C)C)C1CC=C(C=O)C1C=O	4.5	Good	5.6
12938	BrC(COC(=O)C)C1(CC2C(=CC1)C1(C(CC2O)C2(O)C(CC2)C(OC(=O)C)C1)C)C	5.0	Good	5.6
12842	O1C(CCC(=O)C=CC=CC)(C)C(OC)=C(C)C1=O	6.5	Poor	5.6
10147	ON=CCc1c2c([nH]c1)cc(cc2)CC=C(C)C	4.0	OK	5.6
9811	N1C=2C(N=C1NC)=C(C1=NC(=NC1=CC=2)N(C)C)C	4.6	Poor	5.6
8630	N(CCCN1CCCCCC=CCC=CCCCC1)CCCN	3.7	Poor	5.6
7858	Oc1cc([n+]([O-])c2c1cccc2)CCCCCCCCC	4.3	Excellent	5.6
7772	O1C(C2=C(C3(C(CC2)C2(C(CC3OC(=O)CC(O)C)C3(C(CC2)C(CCC3)(CC)C)C)C)C)C1=O)C	4.8	Poor	5.6
5408	OC(C(CCCC(CCC1C(CCCC1C)(C)C)C)=C)CO	5.8	Excellent	5.6
4132	O=C1CC(N2C3(C1CCCC3)CCC2CO)CCCCCC	4.2	Bad	5.6
3992	O1OC(C=CC1(OC)CCCC1OOC(C=C1)CCC)CC(OC)=O	3.3	Good	5.6
3898	O1OC(CC(CC)C1CC(O)=O)(CCCC(CC)C=CCC)CC	4.5	Bad	5.6
2733	O1Cc2c(C=C1C(=CC(CC)C)C)cc1c(C(=O)C(=O)C(OC)=C1)c2O	4.4	Poor	5.6
1133	O(C)c1cc(c2c(C(=O)C(C)(C)C2O)c1C)C(C)C	5.3	Excellent	5.6
536	OC1CC2C3(C(CCC2(C2CC=C(C(=O)C)C(C=O)C12C)C)C(CCC3)(C)C)C(O)=O	5.3	Bad	5.6
436	O1C(CC2C(C3C(CC12C)C=C(CC3)C)(C)C)C1(OOC(CC1)C(C(O)=O)C)C	5.7	Good	5.6
12568	O1C(OC(=O)C)C2C(=CCC3C4(C(CCC23C)C2(C(CC4)C(CCC2)(C)C)C)C)C1=O	5.3	Excellent	5.6
11507	O1C(O)C2=C(C(O)CC3C2(C)C(OC(=O)C)CC2C3(CCC3C(CCCC23C)(C)C)C)C1=O	5.3	Bad	5.6
10703	IC1=CC(O)(CC=CCCCCC)C(=CC=CCCCC(OC)=O)C1=O	5.0	Good	5.6
7975	OCC1=CCCC2C(CCC(O)(C=C)C)(C)C(CCC12C)C	5.1	Excellent	5.6
6259	OC1C23C(CCC2(C=C(C(OC)=O)C3C)C)C(C1)C	4.6	Good	5.6
5060	BrC1CCC(CC1(C)C)C12OCC(O)(CC1)C(Cl)C2O			5.6
4432	O1C(CCCC1=O)C1CC1C=CC(O)C(O)CC=CCCCCC	5.7	Good	5.6
4204	Brc1[nH]c(cc1)C1=NC(=CNCC(C)C)C(OC)=C1	4.5	Good	5.6
2606	o1c(ccc1CC(O)=O)CC=CCC=CCC=CCC=CCC	5.1	Poor	5.6

2013	O1c2c(CC1C=CC=CC)cc(OC(=O)C)cc2OC(=O)C	4.2	OK	5.6
1661	O1C2=C(C=CC(=O)C2(CC)C)C(=O)C(C)=C1C	5.9	Excellent	5.6
1277	OC1C23C(CCC2(C=C(C(O)=O)C3C)C)C(C1)C	5.5	Excellent	5.6
13125	O(C(=O)c1ccccc1C(OCCCC)=O)Cc1ccccc1	3.8	Good	5.5
10730	O1CC(C2C3(C(=CC(=O)CC3C)CCC12O)C)C	5.2	Excellent	5.5
9495	O1C2OC2C(CCC=C(CC(O)CC(CCCc2ccoc2)C)C)C1=O	4.8	OK	5.5
6811	O1C(C2C3(CC=C4C(CCC5C(C)(C)C(O)CCC45C)C3(CC2=O)C)C1=O)(CCCC(O)(C)C)C	3.8	Bad	5.5
5525	O1C(O)C2=C(C=CC3C2(C)C(OC(=O)C)CC2C3(CCC3C(CCCC23C)(C)C)C)C1=O	5.5	Bad	5.5
4580	[nH]1cnc(CCN)c1CC=C(CCC1C(CCCC1=C)(C)C)C	4.6	Excellent	5.5
4462	O(C)c1c(cc(cc1OC)C)C(=O)c1cc(O)cc(OC)c1C=O	5.6	Good	5.5
4347	OC1C=C(C)C2(C(C1)C(CCC(O)(C=C)C)(C)C(CC2)C)C	5.6	Excellent	5.5
1857	o1cc(cc1)CCCC(=CC(=O)C=C(CCCc1ccoc1)C)C	3.9	Good	5.5
1699	O1C(CC=CCCCC1=O)C1CC1C(O)C=CC(O)CC=CCC	4.6	Good	5.5
1347	BrC(C(OC(=O)C)CC1OC(OC=C=C)CC1OC(=O)C)CC=CCC	5.2	Excellent	5.5
14472	O(C(=O)C)C1C23C(C(C(=O)C)C(O)CC2C2(C(C1)C1(C(CC2)C(CCC1)(CC)C)C)C)C(O)C3	5.9	OK	5.5
12116	s1nc(nc1C(=O)c1c2c([nH]c1)cccc2)N(C)C	4.9	OK	5.5
11773	O(C(=O)C)c1c2c(cc(OC(=O)C)c1)cccc2OC(=O)C	3.2	Excellent	5.5
11474	O1CC23C(CCC4(C5Cc6cc(O)ccc6OC5(CCC24)C)C)C(CCC3)(C)C1=O	5.0	Bad	5.5
11131	O1C(O)C2=C(C(O)CC3C(CCCC23C)(C)C)C1=O	5.2	Excellent	5.5
9721	O(C(C(CCC(=CC=O)C)CC=C(CCC(O)C(C)=C)C)(C)C)C(=O)C	5.4	Excellent	5.5
9718	O1CC2(CC(C3C2(O)C(C(CC3OC(=O)C)C)C1O)(C)C)C	4.3	Good	5.5
9636	O1C(C2C3(CC=C4C(CCC5C(C)(C)C(O)CCC45C)C3(CC2=O)C)C1=O)(CCCC(OC)(C)C)C	3.5	Bad	5.5
9392	O1C(O)C2=C(C(OC)CC3C2(C)C(OC(=O)C)CC2C3(CCC3C(CCCC23C)(C)C)C)C1=O	5.7	Poor	5.5
8917	O1C(C(O)C(C(=O)C(C(O)CC(=O)CC)=C)COC(=O)C)(C)C1C(C=CC)C	5.4	Good	5.5
8631	O=CNCCCNCCCN1CCCCCCCCC=CCCCC1	4.0	Bad	5.5
8590	O1C2(O)C(CC3(C(CCCC3C)C2=O)C)=C(C)C1=O	5.8	Excellent	5.5
8232	O1C(O)C2=C(C(O)CC3C(CCCC23C)(C)C)C1=O	5.3	Excellent	5.5
8220	O1C(O)C2C(=CCC3C(CCCC23C)(C)C)C1=O	4.9	Good	5.5
8181	O1C23CC(O)CCC2(C)C(=O)C(CC13)C1CCC(C(C)C2CC2(C(C(C)C)C)C)C1(CCO)C	4.4	Poor	5.5
5822	O1C(=O)C(=CC1OC)CCC1(C)C(CC(=O)C=C1C)C	5.3	Excellent	5.5
5821	O1C(=O)C(=CC1O)CCC1(C)C(CC(=O)C=C1C)C	5.0	Excellent	5.5
3979	O1C2OC=C(C3C2C(CC3)C1OC(=O)C)CCC=C(C)C	4.5	Good	5.5
2409	O(C(=O)C)C1C(=CC2(C(=CC(O)CC2C)CCC1=O)C)C	4.5	Excellent	5.5
1346	BrC(C(OC(=O)C)CC1OC(OC(Br)C#C)CC1O)CC=CCC	5.3	Good	5.5
1121	O1C(C(OC)=O)C(CO)C(C2(C3C(CC2)C(CCCC3=C)(C)C)C)C1O	3.9	Good	5.5
939	S1(=O)(=O)C2=C(NCC1)C(=O)c1c(cc3c(c1)C(=O)c1occ4c1C3(CCC4)C)C2=O	5.6	Poor	5.5
298	Oc1ccc(O)cc1CC=C(CCC=C(C)C)C	4.3	OK	5.5
14059	OC1C2C(CC=C2C)C(CCC1C(CC(O)C=C(C)C)C)=C	4.2	Poor	5.5
13737	O1C(=O)C(=CC1=CC1(OOC(CC1)C(=CCO)C)C)C	3.9	OK	5.5
13287	O1C(C2=C(C3(C(CC2)C2(C(CC3O)C3(C(CC2)C(CCC3)(C(OC(=O)C)C)C)C)C)C)C1=O)C	4.3	Poor	5.5
13138	O1CC2(C3=C(C(C)(C)C2O)C(O)CC(C3=C1)C)C	4.4	Excellent	5.5
12614	OC(CO)(C)C1=CC2(C(CC1)=CCCC2C)C	3.9	Excellent	5.5
12526	OC(CCC=C(CC(=O)C=C(C)C)C)(C=CCC(=CCO)C)C	5.1	OK	5.5
12315	O1C2C(C3(C(=CC(O)CC3C)CC2)C)C(C)C1O	4.7	Excellent	5.5
11039	O1C2C3C(CCC2C(C)C1=O)(C)C(=O)CCC3=C	4.9	Excellent	5.5

10968	OC(C=CC=C(CO)C1CC2C(CC1)(CCCC2=C)C)(C)C	3.9	Excellent	5.5
8741	O(Cc1ccccc1)c1cc2c([nH]cc2CCO)cc1	5.0	OK	5.5
6374	Brc1cc2c([nH]cc2-c2nc(ncc2)N)cc1	4.3	Poor	5.5
5052	O1C(O)C(=CC1=O)C1OCC2(O)C(C1)C1(C(CC2)C2(C(CC1)C(CCC2)(C)C)C)C	5.8	Poor	5.5
3587	O(C(=O)C)C1CC2CCC3C4CCC(C(=O)C)C4(CCC3C2(CC1)C)C	4.3	Poor	5.5
3415	Oc1ccc(cc1)C=CNC1=CC2=NCCc3c2c(n(c3)C)C1=O	3.3	Bad	5.5
2548	O(Cc1ccccc1)c1ccc(cc1[N+](=O)[O-])C(OC)=O	5.4	Good	5.5
2112	O1C(CCCC1=O)C1CC1C(O)C=CC(O)CC=CCCCCC	4.9	OK	5.5
2110	O1C(CCCC1=O)C1CC1C(O)C=CC(O)CC=CCCCCC	3.9	Bad	5.5
1741	o1cc(cc1)CCCC(O)(CC(O)C=C(CC=Cc1ccoc1)C)C	3.9	Bad	5.5
1259	ClC(Cl)(Cl)C(CC1N(C(=O)CC(C(Cl)(Cl)Cl)C)C(=O)C(C)(C)C1O)C	6.1	Excellent	5.5
13791	OC(CC=CCC)C=CC=CCC=CCC=CCCCC(O)=O	5.3	Bad	5.5
13777	Brc1cc2[nH]c3C4N(OCSCC4N)CCc3c2cc1	4.2	Excellent	5.5
13389	OC(CC=CCC)C=CC=CCC=CCC=CCCCC(O)=O	4.3	Bad	5.5
13207	O1C(C=2C(C3(C(CC=2)C2(C(CC3OC(=O)C)C3(C(CC2)C(CCC3)(C)C)CO)C)C)C1O)C	4.6	OK	5.5
12771	OC1CC(C(=CCC=C(C)C)C)C(C2C(C=CC2=O)C)C1C=O	4.8	Excellent	5.5
12768	OC1C2(C=O)C(CCC1(CC=C2C=O)C)C(CCC=C(C)C)C	4.0	Bad	5.5
12185	BrC1CC2OC2CC(OC1CC)C(O)C(Cl)C=CC#C	4.8	Poor	5.5
11949	BrC12OC3C(CC(Br)C(O)(C3)C)(C1(C)C)C(O)(C=C2)C	4.4	Excellent	5.5
11476	O1CC23C(CCC4(C5Cc6cc(OC(=O)C)ccc6OC5(CCC24)C)C)C(CCC3)(C)C1=O	4.5	Bad	5.5
9476	O(CC)C1=C(C)C(=O)C2=C(c3n(C=C2)c(nc3)C)C1=O	4.8	Excellent	5.5
8965	O1C23C1C(O)C=1C(CCC4(C=1CCC4C(CCC(C(C)C)CC)C)C)C2(CCC(O)C3)C	4.1	Poor	5.5
8221	O1CC=2C(C3(C(CC=2)C(CC(O)C3)(C)C)C)C1=O	5.9	Excellent	5.5
5481	O1C(CCC1=O)C=CCC1C(C=CC1=O)C=CCCCCCC	4.2	Poor	5.5
3450	O(C(=O)CCCCC=CCC(=CC=CC=CC(O)CC)C=O)C	5.9	OK	5.5
2604	o1c(ccc1CC(O)=O)CC=CCC=CCC=CCC=CCC	5.1	Excellent	5.5
2598	Brc1c2c3c4n(c2ccc1O)CN(C)C(c4ncc3)CSC	3.5	Good	5.5
2398	S=C=NC1(C2C(=CC(O)(CC2)C)C(CC1)C(C)C)C	4.5	Excellent	5.5
1980	O1C23CC(CCC(=O)C(C)C)(C)C1(O)CCC2(CCCC3=C)C	4.3	Excellent	5.5
1826	S(OC1CC2=CCC3C4CCC(C(CCCC(C)C)C)C4(CCC3C2(CC1)C)C)(O)(=O)=O	5.4	Bad	5.5
889	O1C2(CC(=O)C(=CC=CC(O)C)C)C(CC(O)CC12C)(C)C	4.3	Good	5.5
308	OC(CC=CCC)C=CC=CCC=CCC=CCCCC(O)=O	5.2	OK	5.5
31	ClC1=CC(O)(CC=CCCCCC)C(=CC=CCCCC(OC)=O)C1=O	5.6	Good	5.5
13611	OC1CCC2=CC(O)CC(C)C2(C)C1C(=O)C	4.6	Poor	5.5
13019	O(C(CC(=CCCC(=CC(O)CC(O)(C=C)C)C)C)C=C(C)C)C(=O)C	5.2	Poor	5.5
11304	O=C1N(CCCCCN=C(N)N)C(CC1)=CC=CCC=CCC	4.5	Bad	5.5
9857	OC1C2C3C(C(=C)C2(CCC3C(C)C)C)C1O	4.3	Poor	5.5
8116	Oc1cc([n+]([O-])c2c1cccc2)CCCCCCC	4.9	Excellent	5.5
7886	ClC(Cl)C(C=C1N(C)C(=O)C(N(C)C1=O)CC(CCl)C)C	4.9	Poor	5.5
7222	Brc1cc(Br)cc(C(=O)Nc2ccccc2)c1O	5.5	OK	5.5
6807	O1c2c(CC(NC(=O)C=CC(=CC(CCCCCC)C)C)C1O)cccc2	6.0	Good	5.5
4248	O1C(=O)C(=CC1O)CCC1(C)C(CC=CC1=C)C	5.1	Good	5.5
4183	S1CC(N=C1C1CC1C)C=CCCC=CC=C(CCC(OC)CC=C)C	4.5	Bad	5.5
2391	O=C1CCC(C=CC(O)CCCCC)=C1CC=CCCCC(O)=O	6.1	Poor	5.5
663	O(C)C1=CC(=O)C=C(CC=C(CCCC(O)(C)C)C)C1=O	4.6	Excellent	5.5
13557	O1CC(C2C(C=3CC(CC2)(C)C(O)CC=3)C1=O)=CC=CC(O)(C)C	5.8	Poor	5.5
13531	Clc1cc(C(CCCC(O)(C)C)C)c(O)cc1C	4.5	Excellent	5.5
12532	OC1CCC2(O)CC1(CCC1C2CC1(C)C)C	4.5	Excellent	5.5
12309	O(C(=O)C)C1CCC2=CC(O)CC(C)C2(C)C1C(C=O)C	4.8	Good	5.5
12138	Oc1ccc(cc1)CCNC(=O)C=C(CCCCCCCCC)C	5.6	Poor	5.5
11369	Oc1cc(C)c(O)cc1C(CCC=C(C)C)C	4.7	Excellent	5.5

10981	O(C(=O)C)c1c2c(ccc1)cccc2OC(=O)C	5.0	Excellent	5.5
9527	Oc1c(C=O)c(CCCCCCC)c(O)cc1CC=C(C)C	5.7	Excellent	5.5
8376	O=C1C=CC(C=CC(O)CC=CCC)C1CC=CCCCC(O)=O	6.1	Good	5.5
8295	o1cc(cc1)CCCC(CC(=O)CC(CCCc1ccoc1)C)C	5.0	Poor	5.5
7567	ClCCc1c(c2CC(C)(C)C(O)c2cc1C)CO	4.1	Excellent	5.5
6254	O1C(CC2C(CCC3C4(C(CCC23C)C(CCC4)(COC(=O)C)C)C)C1O)C1=CC(OC1O)=O	4.1	Bad	5.5
3223	O1C2C(C(CC2O)C1CC=CCC)C=CCCCCC(O)=O	4.9	Excellent	5.5
3200	O(C)c1c2c(c(O)c(c1)C=O)C(CCC2C)C(C)C	5.1	Excellent	5.5
1922	O1c2c(CC1(C=CC=CC)C)cc(O)cc2O	4.4	Poor	5.5
555	OC(C(=O)c1c2c([nH]c1)cccc2)CC(=O)C	5.5	Excellent	5.5
2340	ClC=1C2=NCCc3c2c(n(C)c3SC)C(=O)C=1N	5.0	Excellent	5.5
13327	O1CC(=C)C2(CC3CC(O)CC(C)C3(C)C2O)C1OC	4.4	Excellent	5.4
12187	O(C(=O)C=C(CCC=C(CCC=C(C)C)C)C)CC(O)CO	5.9	OK	5.4
10546	Brc1cc(O)c(cc1O)C(=O)C=C(CCC=C(C)C)C	6.3	Poor	5.4
7863	O1c2c(C(=O)C=C1CCC)c(OC)c1c(cc(O)cc1OC)c2	5.6	Good	5.4
7261	OCC=C(CCC=C(CCC(=O)C(CC(=O)C=C(C)C)=C)C)C	6.1	Poor	5.4
6649	Oc1cccc(-c2cc(ncc2)C)c1NC(=O)CCCCCCCCC	4.7	OK	5.4
3520	OC1C2CC(O)CCC2(C)C(=O)C(=C1)C1CCC(C(C=CC(C)C)C)C1(CCOC(=O)C)C	5.1	Poor	5.4
3332	O1C(=O)C(CC1C=C(C)C)=CCCC(=CCCC(=O)C)C	6.0	Good	5.4
2102	OC(C=CC(=O)C(O)(C)C)(C)C1CC(C(C)=C)C(CC1)(C=C)C	4.3	OK	5.4
1438	O1CC12CCCC1C(CCC(CC(O)=O)C)(C)C(CCC12C)C	6.3	OK	5.4
14340	OC(CC=CCC)C=CC=CCC=CCC=CCCCC(O)=O	5.0	Excellent	5.4
13778	Brc1cc2c3CCN4OCSCC(N)C4c3[nH]c2cc1	3.9	Excellent	5.4
11848	O1C2C13C(CCC1(C3CCC1C(CCCC(C)C)C)C)C1(CCC(OC(=O)C)CC1(O)C2O)C	3.3	Poor	5.4
10472	OC1C2C(CC=C2C)C(=C)C(O)CC1C(CCC=C(C)C)C	4.5	Excellent	5.4
8210	BrC1C(C)(C)C2(CCC(O)(C=C2)C(Br)Br)C(CC1O)=C	4.7	Excellent	5.4
7866	OC1CC(=O)C(CC=CCCCC(OC)=O)C1C=CC(O)CCCCC	6.2	Excellent	5.4
7837	OC(CCC)C=Cc1cc(ccc1C=CCC(=O)N)C	4.8	Good	5.4
7369	O1CC(C2C(C3(OC)CC(CC2)(C)C(O)CC3)C1=O)=CC=CC(C)C	5.9	Good	5.4
7210	OC1C(C2C(=CC1=O)C=CC2)C=C(C=CC(O)CCCCCCC)C	4.7	Bad	5.4
6869	O1C(O)C2=C(CCC3C(CCCC23C)(C)C)C1=O	4.8	Excellent	5.4
6657	O(C)c1cc(ccc1C(C=CC=C(C)C)C)C(O)=O	5.4	Excellent	5.4
6490	O1c2c(Oc3c1ccc(O)c3C=O)ccc(O)c2C=O	5.6	Excellent	5.4
6243	O1C(=O)C(=CC1OC)CCC=C(C(O)CC=C(C)C)C	4.5	Excellent	5.4
6191	Oc1ccc(cc1)CCC=CC=CC=CCCOC(=O)C	4.3	Bad	5.4
6173	Oc1c2c(c3[nH]c(cc3c1C)COC)C(=O)CC2	4.4	Excellent	5.4
5482	O1C(CCC1=O)C=CCC1C(C=CC1=O)C=CCCCCCC	6.9	Excellent	5.4
5361	n1c2c3C(CC(C)C2CCCC)CCc3nc1N	4.9	OK	5.4
5188	O1C2(C)C(O)C(COC(=O)C)C1(OC2C(C=CC)C)C(C(O)CC(=O)CC)=C	3.9	Bad	5.4
4832	O(C(=O)C)C1C2C(CCCC2(C2CCC(=CC2(C1)C)C1CC(=O)C(=CC1O)C)C)(C)C	4.5	Bad	5.4
3922	ClC=C(CNC(=O)CCC=CCC(OC)CCCCC)C=1C(=O)C(CCC=1C)C	4.7	Bad	5.4
3581	OC1C2C(CC=C2C)C(CCC1C(CCC(O)C(OC)(C)C)C)=C	4.6	Good	5.4
3395	O1CC2C(=CCC3C2(C)C(=O)CC2C3(CCC3C(CCCC23C)(CC)C)C)C1(O)C	4.9	Bad	5.4
1946	O1C(=O)C(=C2CC3(C(CC12O)=CCCC3C)C)C	5.4	Excellent	5.4
1761	O(C)C1=C(C)C(=O)c2c(C1=O)c(ncc2)COC(=O)CC	5.0	Excellent	5.4
1686	OC1CCC23C(CCC4C2(CCC2(C)C4(CCC2C(CC(=O)C=C(C)C)C)C)C3)C1(C(O)=O)C	6.3	Poor	5.4
1519	OC1C2C(CC=C1C)C(O)(CCC2C(CCC=C(C)C)C)C	4.5	Excellent	5.4
517	OC1(C2C(C=C(CC1)C(CC(=O)CC(C)C)C)C(O)(CC2)C)C	3.9	Good	5.4

74	O1C(C2C3(CC=C4C(CCC5C(C)(C)C(O)CCC45C)C3(CC2)C)C1=O)(CC(=O)CC(C)C)C	5.2	Good	5.4
13867	O(C(=O)C)C=1C(=O)C(=CC(=O)C=1C)C(CCC=C(C)C)C	4.3	Poor	5.4
13759	OC1CCC(C2C(CC=C1C)C(C2)(CC(O)C=C(C)C)C)=C	5.4	Excellent	5.4
13453	N1C=2C3C(CC(C)C=2CCCC)CCC3N=C1N	3.6	Excellent	5.4
12477	N1C2C3C(CC(C)=C2CCCC)CCC3N=C1N	3.4	Excellent	5.4
8678	O1CC2=C(C3C(CO)C2(CCC3C(C)C)C)C1=O	5.2	Excellent	5.4
7771	O1C(C2=C(C3(C(CC2)C2(C(CC3OC(=O)CC(O)C)C3(C(CC2)C(CCC3)(CC)C)C)C)C)C1=O)C	4.2	Good	5.4
6656	Oc1c(O)c(ccc1C(C=CC=C(C)C)C)C=O	5.0	Excellent	5.4
6179	O1OC(C=C(CC)C1CC(O)=O)(CC(CCCC)C)CC	5.6	Poor	5.4
5061	BrC1CCC(CC1(C)C)C1(Cl)C2OC2C(O)(CC1)CO	5.1	Poor	5.4
4648	S1CC(N=C1C1CC1C)C=CCCC=CC=CCCC(OC)CC=C	4.7	Bad	5.4
1921	OC1CC(C=CCC(=C)C(O)CCC1=C)(C)C	4.4	Excellent	5.4
637	OC1CCC(O)(C=CC(CCC1=C)C(C)C)C	4.7	Good	5.4
13946	ClC(Cl)(Cl)C(CC(OC)=CC(=O)N1C(C(C)C)C(OC)=CC1=O)C	4.3	Excellent	5.4
13843	OC1CC(=CCC=C(C=O)C(C=O)C1C(CCC=C(C)C)C)C	4.9	Bad	5.4
12139	O(C)c1cc(ccc1O)CCNC(=O)C=C(CCCCCCCCC)C	4.4	Bad	5.4
11486	ClC(Cl)(Cl)C(CC1N(C)C(=O)C(N(C)C1=O)=CC(C(Cl)(Cl)Cl)C)C	3.6	Excellent	5.4
10389	OC1C2C3C(C(=C)C2(CCC3C(C)C)C)C1O	5.3	Excellent	5.4
9303	O=C1CN(C)C(=O)C1=C(O)CCCCCCCCCCCC(C)C	4.3	Poor	5.4
8964	O(C(=O)CCC(O)=O)C1CC2=CCC3C4CCC(C(=O)C)C4(CCC3C2(CC1)C)C	5.5	Bad	5.4
8660	S1SC2(N(C)C(=O)C1(N(C)C2=O)Cc1ccccc1)CO	4.0	OK	5.4
8592	O1CC2=C(C3(C(CC2)C2(C(CC3O)C3(C(CC2)C(CCC3)(COC(=O)C)C)C)C)C)C1=O	4.5	Bad	5.4
6953	OC(C(=C)C1CCC(NC=O)(CC1)C)C=CC(C)C	5.4	Excellent	5.4
6767	O1C(CC(CCCCC(C)C(=O)N(C=CCC1=O)C)C)C(C)(C)C	4.2	Poor	5.4
6389	ClC(Cl)(Cl)C(CC1N(C)C(=O)C(N(C)C1=O)=CC(C(Cl)(Cl)Cl)C)C	4.8	OK	5.4
5661	ClC(Cl)C(CC(N(C(=O)CC(C(Cl)Cl)C)C)C(=O)NCc1sccn1)C	4.1	Good	5.4
4821	O1C(O)C(=CC1=O)CCCC(CCCC(CCCC(C)C)C)C	4.0	Bad	5.4
4205	Brc1[nH]c(cc1)C1=NC(=CNCC(CC)C)C(OC)=C1	4.3	Good	5.4
3671	ClC(Cl)(Cl)C(CC1NC(=O)C(N(C)C1=O)=CC(C(Cl)(Cl)Cl)C)C	4.7	Excellent	5.4
3486	O1c2cc(ccc2OC1)C=C1N=C(N)N(C)C1=O	4.4	Excellent	5.4
697	OCC(CO)=C1CC=2C(CC1)(CCCC=2C)C	5.4	Excellent	5.4
14338	O(C(=O)C)c1c(C(CCC=C(C)C)C)c(O)cc(C)c1O	3.8	Excellent	5.4
13444	O(C(=O)C=CC(O)(C)C)C1C(C(C)=C)C(CCC1C(=O)C)(C=C)C	5.3	Excellent	5.4
9306	O=C1CN(C)C(=O)C1=C(O)CCCCCCCCCCC(C)C	4.8	Poor	5.4
8747	Brc1cc2OC(C=Cc2cc1O)(CCC(O)C(C)=C)C	5.3	Excellent	5.4
8334	OC=1C(=O)C(=CC(=O)C=1C(CCC=C(C)C)C)C	4.7	Excellent	5.4
8014	O(C)C=1C(=O)C(=CC(=O)C=1C(CCC=C(C)C)C)C	5.4	OK	5.4
7801	O1OC(CC(CC)C1CC(O)=O)(CC)C=CCC(CC)C=CCC	4.1	Bad	5.4
6270	ClC(Cl)C(CC1N(C)C(=O)C(N(C)C1=O)=CC(C(Cl)Cl)C)C	5.0	Excellent	5.4
5732	O1C2CCC(=C)C(CCC3(O)C4(C(OC(C)(C)C(=O)CC4)CCC3C)C)C2(CCC(O)C1(C)C)C	4.7	Bad	5.4
5551	OC=1C=C(C)C(=O)C(=O)C=1C(CCC=C(C)C)C	5.2	Excellent	5.4
5528	O1c2c(cc(O)cc2)C=CC1(CC(=O)CC(C)C)C	4.7	Excellent	5.4
1746	OC12CC(=O)C(C)C1(C1CC(CC1(O)C2)(C)C)C	5.3	Excellent	5.4
13671	O1OC(CC(CC)C1CC(O)=O)(CC(CC)C=CCC)C	4.8	Excellent	5.4
12807	ClC(Cl)(Cl)C(CC(N(C(=O)CC(C(Cl)Cl)C)C)C(=O)NCc1sccn1)C	4.4	Excellent	5.4
12204	OC(=O)CC1C2CCC(C)C1(CC2(C)C)C(=O)C			5.4
12175	O1C(Cc2c(C1=O)c(O)ccc2)CCCCCCCCCCC	5.2	Poor	5.4
11458	O1C(C)(C2CC(O)CCC23OC3)C1CC=C(C)C	5.5	Excellent	5.4

11301	o1c(ccc1C)CC(CCC(=O)c1ccoc1)C	4.2	Excellent	5.4
10868	o1cc(cc1C=C(CCC=C(C=C)C)C)C(O)=O	4.6	Excellent	5.4
10094	o1c(CCCCCCCCC(O)=O)c(C)c(C)c1CCC	4.9	Bad	5.4
8130	O(C(=O)C)C12C3CC(CC(C1(CC2)CO)C(=C3)C)(C)C			5.4
6326	N(CCCN1CCCCCCCCCCCCC1)CCCN	3.3	Bad	5.4
2559	O1C(CCC=C(CCC=C(C)C)C)(C)C1CCC(=CCO)CO	5.4	Good	5.4
12755	Oc1c(cc(O)cc1C)CC=C(CCC=C(C)C)C	4.8	Good	5.3
8745	Brc1cc2OC(CC(=O)c2cc1O)(CCCC(O)(C)C)C	5.6	Poor	5.3
6760	O1c2c(C(=O)C1=Cc1cc(OC)c(OC)cc1)c(OC)cc(OC)c2	4.6	OK	5.3
4889	O1C(=O)C(=CC1O)CCC=C(CCC=C(C)C)C	4.7	Poor	5.3
2862	O1C(=O)C(=CCC1C(O)(C)C)C1CC(C(C)=C)C(CC1)(C=C)C	6.4	OK	5.3
1554	O1C2C(OC3CC4OC(CC(O)C4OC3(CC2)C)CC(C=O)=C)CCC(O)(C)C1CCC=C C=C	4.2	Poor	5.3
13214	o1cc(cc1)CCC=C(CC(O)C=C(CC=Cc1ccoc1)C)C	4.7	Poor	5.3
13046	O=C1N(CCCCCN=C(N)N)C(CC1)=CC=CCC=CCC	5.0	Poor	5.3
12013	O1C2C(C3(C(=CC(O)CC3C)CC2)C)C(C)C1O	4.0	Good	5.3
11339	O1C2C(CCC(=C)C(=O)CCC(=C2)C)C(C)C1=O	5.4	Excellent	5.3
10744	OC1CCC(O)(C=CC(CCC1=C)C(C)C)C	5.4	Excellent	5.3
8296	o1cc(cc1)CCCC(CC(=O)CC(=CCCc1ccoc1)C)C	4.3	Excellent	5.3
8217	o1cc(cc1)CCCC(CC(O)CC(=CCCc1ccoc1)C)C	4.5	Poor	5.3
7899	BrC1CC(CCC1(O)C)C(=CC=CC(O)(CCC=C(C)C)C)C	4.2	Excellent	5.3
7563	O([N+](=O)[O-])Cc1c2CC(Cc2cc(C)c1CCO)(C)C	4.7	Good	5.3
4065	O1OC(CCC1(OC)CCCC=CC(=CCC)C)C(C(O)=O)C	4.5	Poor	5.3
4064	O1OC(CCC1(OC)CCCC=CC(=CCC)C)C(C(O)=O)C	5.0	Excellent	5.3
4060	O1OC(CCC1(OC)CCCC=CC(=CCC)C)C(C(O)=O)C	6.6	Excellent	5.3
1650	OC(C(=CCC(=O)C1(CCCC1C(=O)C)C)C)CC=C(C)C	3.9	Poor	5.3
1062	OC(CC=CCC=CCCCC(O)=O)C=CC=CC(=O)C=CCC	4.9	Bad	5.3
14070	O(C(=O)C)C=C(C(O)CC=C(CCC=C(C)C)C)C=CO	3.5	Excellent	5.3
14069	OCC=1CCC(C(CCC=C(CCC=C(C)C)C)C=O)C=1C=O	4.3	Bad	5.3
13610	OC1CCC2=CC(O)CC(C)C2(C)C1C(=O)C	4.8	Excellent	5.3
11947	O1OC(CC(CC)C1CC(OC)=O)(CC(CC)C=CCC)C	4.8	Good	5.3
10731	ClC(Cl)(Cl)C(CC(OC)=CC(=O)N1C(C(C)C)C(OC)=CC1=O)C	4.0	Poor	5.3
9958	OCC1=CCCC2C(CCC(O)(C=C)C)(C)C(CCC12C)C	4.5	Excellent	5.3
9488	S1SC2(N(C)C(=O)C1(N(C)C2=O)Cc1ccccc1)CO	3.6	Poor	5.3
9435	OC(=O)C12C3(C(CC1(C1C(C3)C(CC1)C)C=O)C=C2C(C)C)CO	4.9	Poor	5.3
8746	Brc1cc2OC(C=Cc2cc1O)(CCCC(O)(C)C)C	3.9	Excellent	5.3
8572	OC1CC(=O)C(CC=CCCCC(OC)=O)C1C=CC(O)CCCCC	4.9	OK	5.3
8223	O1C(=O)C2(O)C(C3(C(CC2)C(CCC3)(C)C)C)C1O	5.1	Excellent	5.3
7884	ClC(Cl)(Cl)C(CC1NC(=O)C(N(C)C1=O)=CC(C(Cl)(Cl)Cl)C)C	4.5	Excellent	5.3
7648	O=C1C=C(Nc2c1cccc2)CCCCCCCCCO	4.5	Excellent	5.3
6182	O1OC(C=C(CC)C1CC(OC)=O)(CC(CCCC)C)CC	4.5	Excellent	5.3
5409	OC(CCCC(CCC1C(CCCC1C)(C)C)C)(CCO)C	5.1	OK	5.3
5062	BrC1CCC(CC1(C)C)C12OC1C(O)C(Cl)(CC2)CO	4.9	Good	5.3
4647	S1CC(N=C1C1CC1C)C=CCC=CC=C(CCC(OC)CC=C)C	3.8	Bad	5.3
4509	O1C2C(C(C)C1(O)CCC(C)C)C1(C(C3C(CC1)C1(C(CC(=O)C=C1)CC3)C)C2)C	3.9	Poor	5.3
4422	O1C(CCCC1=O)C1CC1C=CC(O)C(O)CC=CCCCCC	5.9	Good	5.3
3566	O(Cc1ccccc1)C(=O)NCCCC(NC(=O)C)C(OC)=O	4.7	Poor	5.3
673	O1C=C(c2ccc(OC)cc2OC)C(=O)c2c1cc(OC)cc2OC	3.0	OK	5.3
14128	BrC1C(C23CC(CC2)C(CC3(O)CC1O)=C)(C)C	4.6	Excellent	5.3
13658	o1cc(cc1)CCCC(O)(CCC=C(CCCC(O)=O)C)C	4.2	Excellent	5.3
13443	O1C2C(CCC(=C)C(O)CCC(=C2)C)C(C)C1=O	5.6	Excellent	5.3
12586	O1C(CCC(=O)C=CC=CC)(C)C(O)=C(C)C1=O	6.4	Excellent	5.3

12206	O1C(C(=CC2(OOC(CC(OC)=O)C(C2)CC)CC)CC)C1CC	4.6	Poor	5.3
11457	OC1CCC2(C(=CCC(O)CC2)C1C(C)C)C	3.7	Excellent	5.3
11340	O1C2C(CCC(=C)C(O)CCC(=C2)C)C(C)C1=O	5.5	Excellent	5.3
9829	O1OC(CC(CC)C1CC(O)=O)(CC)C=CCC(CC)C=CCC	5.0	Bad	5.3
8684	O1CC12C1C3C(C2(CCC3C(C)C)C)C(O)C1O	4.4	Excellent	5.3
8575	O(C)c1cc(cc(OC)c1O)C=CC(OC(C)C)=O	4.8	Excellent	5.3
7304	OC1CC2C3(C(CCC2(C2CC=C(C(=O)C)C(C=O)C12C)C)C(CCC3)(C)C)COC(=O)C	4.9	Good	5.3
7007	S1CC(N=C1CC(C(CC(CCN(C(=O)C(CC)C)C)C)C)C)C=C	4.5	Bad	5.3
6392	ClC(Cl)C(CC1N(C)C(=O)C(N(C)C1=O)=CC(C(Cl)Cl)C)C	4.6	Excellent	5.3
6329	O1OC(CC(CC)C1CC(OCC)=O)(CC)C=CCC	5.5	Excellent	5.3
6318	O1C(O)(c2c(C1=O)c(O)ccc2)CCCCCC	5.0	Excellent	5.3
5026	Oc1ccc(cc1[N+](=O)[O-])CCNC(=O)C(C)C	5.4	Excellent	5.3
14302	O(C(=O)C=C(CCC1C(CCCC1=C)(C)C)C)CC(O)CO	4.8	Bad	5.3
14177	o1cc(cc1)CC1=CC(O)(CCC1C(C)C)CO	4.7	Excellent	5.3
13650	OC1(C2C(C=C(CC1)C(C)C)C(O)(CC2)C)C	4.4	Excellent	5.3
13308	O(C)C1=CC(=NC1=CNCC(C)C)c1[nH]ccc1	4.4	Excellent	5.3
12005	OC1(C2C(C=C(CC1)C(C)C)C(O)(CC2)C)C	4.3	Excellent	5.3
11626	S(C)C1(N(C)C(=O)C(SC)(N(C)C1=O)CO)Cc1ccccc1	5.0	Excellent	5.3
9499	S(C)C1(N(C)C(=O)C(SC)(N(C)C1=O)CO)Cc1ccccc1	3.9	Poor	5.3
9111	O(C)c1cc(cc(OC)c1O)C=CC(OCCCC)=O	5.0	Good	5.3
7855	O1c2c(cc(OC(=O)C)cc2)CC2C3(C(CCC12C)C1(C(CC3)C(CCC1)(C(O)=O)C)C)C	5.4	Bad	5.3
7570	OCc1c2CC(Cc2cc(C)c1CCO)(C)C	5.0	Excellent	5.3
7308	O(C)C1=CC(=O)c2c(C1=O)c(OC)c(OC)cc2OC	5.4	Excellent	5.3
6390	ClC(Cl)(Cl)C(C=C1N(C)C(=O)C(N(C)C1=O)CC(C(Cl)Cl)C)C	4.8	Excellent	5.3
3629	ClC(Cl)(Cl)C(C=C1N(C)C(=O)C(N(C)C1=O)=CC(C(Cl)(Cl)Cl)C)C	4.2	Poor	5.3
3628	ClC(Cl)C(CC1N(C)C(=O)C(N(C)C1=O)CC(C(Cl)Cl)C)C	4.4	OK	5.3
3614	ClC1C2CCC3C4CCC(C(CC(=O)C(C(C)C)=C)C(O)=O)C4(CCC3C2(CCC1O)C)C	5.6	Poor	5.3
3418	O1c2cc(ccc2OC1)Cc1nc(n(c1)C)N	4.2	Good	5.3
1896	O1C2CCC(=CC3C(CCC12C)C3(C)C)COO	5.8	Excellent	5.3
988	s1cc(nc1C(C(O)CC=CC(CCNC(OC)=O)=C)(C)C)CC=CCC=C	3.6	Poor	5.3
13166	Clc1c(O)c2c(CC(OC2=O)C)cc1OC	4.3	OK	5.3
12225	O1CC(=C2C1CC1CCCC(C)C1(C)C2O)CO	4.4	Excellent	5.3
11280	O1C(CCC1(C)C)C1(OC(=O)C23CC=C4C(=CCC5C(C)(C)C(O)CCC45C)C2(CCC13O)C)C	4.4	OK	5.3
11202	O1C(CCC2OC2(C)C)(C)C1CCC(=CC(OC)=O)C	4.8	OK	5.3
9307	O=C1C(=C(O)CCCCCCCCCCCC)C(=O)N(C)C1C	4.4	OK	5.3
8742	Brc1cc(OC)c(cc1O)CC=C(CCCC(O)(C)C)C	4.8	Poor	5.3
7489	O(C(=O)C)C1CCC2(C(CCC3(C2CC(=O)C3=C(C=CC=C(C=CC=O)C)C)C)C1(C(O)=O)C)C	5.8	Bad	5.3
7197	Brc1cc(cc(Br)c1OCCCNC(=O)C#N)CCNC	2.9	Poor	5.3
6398	ClC(Cl)(Cl)C(CC1(O)N(C)C(=O)C(N(C)C1=O)=CC(C(Cl)(Cl)Cl)C)C	6.2	Excellent	5.3
6396	ClC(Cl)(Cl)C(CC1(O)N(C)C(=O)C(N(C)C1=O)=CC(C(Cl)(Cl)Cl)C)C	3.7	Excellent	5.3
4255	Clc1c(O)c2c(CC(OC2=O)C)cc1OC	5.5	Excellent	5.3
2554	OC(CC=CCC=CCC)C=CC=CCCCCC(O)=O	5.7	Bad	5.3
1415	O1C(CCC2OC2(C)C)(C)C1CCC(=CC(OC)=O)C	4.8	OK	5.3
1140	OC(CC=CCC=CCC)C=CC=CCC=CCCCC(O)=O	4.6	Bad	5.3
13422	OC1CC(CCC(=C)C(O)CCC1=C)C(C)=C	4.6	Excellent	5.2
13259	O1C(=O)C(=CC1O)CCC1C(CCCC1=C)(C)C	5.0	Excellent	5.2
12476	N1C=2C3C(CC(C)C=2CCCC)CCC3N=C1N	4.2	Poor	5.2

10591	OC1=CC(=O)C(C=CC(O)CCCCC)C1CC=CCCCC=O	4.8	Poor	5.2
9304	O=C1CN(C)C(=O)C1=C(O)CCCCCCCCCCC(CC)C	5.2	Bad	5.2
13957	O1C(O)(CC)C(C)C(=O)C(C)C1C(=CC(CCC)C)C	3.8	Excellent	5.2
13612	OC1CCC2=CC(O)CC(C)C2(C)C1C(=O)C	4.5	Poor	5.2
13500	Oc1ccc(O)cc1C1C=C(CCC1C(OC)(C)C)C	4.5	Excellent	5.2
12655	S(OC1CC2=CCC3C4CCC(C(C=CCC(C)C)C)C4(CCC3C2(CC1)C)C)(O)(=O)=O	6.5	Excellent	5.2
11614	OC(C(O)CC=C(C)C1CC(C(C)=C)C(CC1)(C=C)C)(C)C	3.7	Poor	5.2
11305	O=C1N(CCCCN=C(N)N)C(CC1)=CC=CCC=CCC	4.7	Bad	5.2
9748	O=C(NCCc1ccccc1)NCCc1ccccc1	5.3	Excellent	5.2
9300	O=C1CN(C)C(=O)C1=C(O)CCCCCCCCCCCCC(C)C	4.2	Bad	5.2
8615	O1OC(CC(CC)C1CC(OC)=O)(CC)C=CC(=O)CC	4.3	Good	5.2
7592	O1C(CCCCC(CC(=CC(=CC(O)=O)C)C)C)C(CO)C1=O	5.8	Excellent	5.2
6952	O=C(C(=C)C1CCC(NC=O)(CC1)C)C=CC(C)C	4.7	Poor	5.2
6647	O1C23CC(OC(=O)C)CCC2(C)C(=O)C(=CC13)C1CCC(C(CCCC(C)C)C)C1(CCO)C	3.9	Bad	5.2
6335	O1OC(C=C(CC)C1CC(OCC)=O)(CC(CCCC)C)CC	3.6	Good	5.2
6171	O=C1CCc2c1c1[nH]c(cc1c(c2)C)COC	4.8	Excellent	5.2
4136	S(CC1N2C3(C(CCCC3)C(=O)CC2CCCC)CC1)C#N	4.5	Excellent	5.2
3991	O1OC(C=CC1(OC)CCCC1OOC(C=C1)CCC)CC(O)=O	3.7	Excellent	5.2
2393	ClC(Cl)C(CC1N(C(=O)CC(C(Cl)Cl)C)C(=O)C(C)(C)C1O)C	4.7	Excellent	5.2
1742	o1cc(cc1)CCC=C(CC(O)C=C(CC=Cc1ccoc1)C)C	4.2	OK	5.2
1491	O(C(=O)C=CC=CC(CC)C)C1C=CC2=CC(=O)C(O)CC2(C)C1C	5.0	OK	5.2
662	O(C)C1=CC(=O)C=C(CC=C(CCCC(O)(C)C)C)C1=O	4.5	OK	5.2
13970	OC(CCCC(O)=O)C=CC=CCC=CCC=CCC=CCC	4.9	Bad	5.2
13613	OC1CC(C)C2(C(=C1)CCC(=O)C2C(=O)C)C	3.8	Excellent	5.2
13598	O(C(=O)CC(O)C(CC(=CC(=O)CC(CC)C=CCC)C)CC)C	5.7	OK	5.2
13597	O1OC(CC(CC)C1CC(OC)=O)(CC(CC)C=CCC)C	4.7	Good	5.2
13532	Brc1cc(C(CCCC(O)(C)C)C)c(O)cc1C	5.5	Excellent	5.2
12587	Oc1c(C)c(O)c(cc1C(=O)C=CC=CC)C	4.3	Excellent	5.2
12375	O1C2(OC)C3CC(CCC2=CC1=O)(C)C(=C)C(O)(C3)C	5.1	Excellent	5.2
12207	O1C(C(=CC2(OOC(CC(OC)=O)C(C2)CC)CC)CC)C1CC	4.6	Poor	5.2
11933	O1OC(CC(CC)C1CC(OC)=O)(CC(CCCC)CC)C	4.4	Good	5.2
8617	O1OC(CC(CC)C1CC(OC)=O)(CC)C=CCCC	4.8	Excellent	5.2
8051	O(C)c1ccc(cc1)C=Cc1cc(O)cc(O)c1	4.0	OK	5.2
6393	ClC(Cl)(Cl)C(CC1N(C)C(=O)C(N(C)C1=O)=CC(C(Cl)Cl)C)C	4.5	Good	5.2
6244	O1C(CC2C3(C(CCC12CO)C1(C(CC3)C(CCC1)(C)C)C)C)C1=CC(OC1O)=O	3.7	Bad	5.2
2488	BrC1CC(O)C(=C)C(CCC(O)(C=C)C)C1(C)C	3.9	Good	5.2
2353	O=C1C=CC(C=CC(O)CCCCC)C1CC=CCCCC(O)=O	4.6	OK	5.2
55	O(C)C=1C(=O)C(=CC(=O)C=1C(CCC=C(C)C)C)C	5.6	Excellent	5.2
14409	O=C1C=CC(CC=CCCCCC)C1CC=CCCCC(OC)=O	5.9	Good	5.2
12750	BrC(CCOc1ccc(Cl)cc1Cl)(C(O)=O)C(O)=O	5.8	Bad	5.2
10639	O(C(=O)C1N(CC(C1)C#N)Cc1ccccc1)CC	3.7	OK	5.2
9725	BrC1CCC(O)(C)C2(CC(Br)C(Cl)(CC2O)C)C1(C)C	4.5	Excellent	5.2
9379	S(C)C1(N(C)C(=O)C(SC)(N(C)C1=O)CO)Cc1ccccc1	4.9	OK	5.2
8574	OC1(CC=C(C)C2(CCC(O)(C=C2)C)C1=C)C	4.4	Excellent	5.2
7832	OC1C(C(=O)C)C(O)C=C(C)C1=CC=CC(=CC=CC(C)C)C	4.2	OK	5.2
7388	O=C1C(=C(O)CCC(CCCCCCCC)C)C(=O)N(C)C1C	5.3	Good	5.2
6391	ClC(Cl)(Cl)C(C=C1N(C)C(=O)C(N(C)C1=O)CC(C(Cl)Cl)C)C	4.9	Poor	5.2
4751	O(C(=O)C)C1CC2C3C(CCC2(C)C1C=C)C1(C(CC(OC(=O)C)C=C1)CC3)C	4.4	Bad	5.2
4066	O1OC(CCC1(OC)CCCC=CC=CCC)C(C(O)=O)C	5.4	Good	5.2
2341	S(C)c1n(c2c3c1CCN=C3C=C(N)C2=O)C	3.8	Excellent	5.2
561	O1C(C)(C2C(OC)C(O)CCC23OC3)C1CC=C(C)C	3.6	Excellent	5.2

13586	OC1CCC(C2=CC(CCC12C)C(O)(C)C)C	4.6	OK	5.2
13230	O(C)c1c2NC=Cc3nccc(cc1OC)c23	4.7	Excellent	5.2
12098	O1C(OC)(CCCCCCCCCCC(OC)=O)C(C)=C(C)C1=O	5.8	OK	5.2
11625	S(C)C1(N(C)C(=O)C(SC)(N(C)C1=O)CO)Cc1ccccc1	5.9	Good	5.2
9790	OC1=C(CCCCCCCCCCC)C(=O)C(O)=CC1=O	5.3	Good	5.2
9309	O=C1C(=C(O)CCCCCCCCCC(C)C)C(=O)N(C)C1C	4.8	Excellent	5.2
8680	O1CC2C(C3C(=C)C2(CCC3C(O)(C)C)C)C1=O	5.3	Excellent	5.2
8521	OC1C2CC=C3C4CCC(C(CC5=[N+]([O-])CCC5=C(C)C)C)C4(CCC3C2(CCC1N(C)C)C)C	3.7	Poor	5.2
6954	O(C(C=CC(O)C(C)C)(C)C1CCC(NC=O)(CC1)C)C	5.7	Excellent	5.2
6397	ClC(Cl)(Cl)C(CC1(O)N(C)C(=O)C(N(C)C1=O)=CC(C(Cl)(Cl)Cl)C)C	3.7	Good	5.2
5435	O(CCCCCCCC)c1cc(O)c(cc1)C(=O)c1ccccc1	5.6	Bad	5.2
3836	O1C2OC3OC2(CC1=O)C(OC(=O)C)CC1C3CCC2C3(C(CCC12C)C(CCC3)(C)C)C			5.2
3555	O1C23C1C(O)CC(C)C2(C)C(C(=O)C)C(=O)CC3	4.4	Excellent	5.2
639	OC(CC=CCC=CCC)C=CC=CCC=CCCCC(O)=O	5.2	Poor	5.2
214	O1CC1(C(CCC)C=C=CC)C(O)C=CCC=CCCCC(O)=O	5.1	Good	5.2
12558	OC=C(CCC1C(CCC=C1C)(C)C)C=CO	4.2	Excellent	5.2
12283	O1C(=O)C(CCC1(CCCCCCCCC)CO)C	4.2	Excellent	5.2
9305	O=C1C(=C(O)CCCCCCCCCCCCC)C(=O)N(C)C1C	4.5	Poor	5.2
7611	ON=CCCCCCCCCCCCCc1cccnc1	4.6	Bad	5.2
6790	O1C2CCC(=O)CC2(CCC1C(O)(C)C)C	4.8	Excellent	5.2
5865	ClC1C(O)C2(OC(OC)C(NC(=O)C=CC(=CC(CCCCCC)C)C)C2)C=C(Cl)C1=O	4.1	Bad	5.2
4782	BrC=1C(O)C2(ON=C(C2)C(O)=O)C=C(Br)C=1OC	4.7	Excellent	5.2
1897	O1C2CCC(=C)C(OO)C3C(CCC12C)C3(C)C	4.4	Excellent	5.2
573	OC(CC(O)c1ccccc1)c1ccccc1	4.5	Excellent	5.2
15	O1C(C(CC)C)C(C)C(=O)C(C)=C1C(C(O)CC)C	4.9	Excellent	5.2
13849	O(C)C1(c2c(CC1O)c(ccc2C=C(C)C)C=O)C	4.6	Excellent	5.1
13720	O(C)C1=CC(=NC1=CNCCC=CCCCCCCCC)c1[nH]ccc1	5.4	Poor	5.1
12137	Oc1ccc(cc1)CCNC(=O)C=C(CCCCCCCCC)C	6.1	Poor	5.1
2474	Brc1cc2[nH]cc(c2cc1)C=CC(OC)=O	4.1	OK	5.1
13268	o1cc(cc1CC(CCC=C(C=C)C)C)C(O)=O	4.9	Excellent	5.1
13212	O1C(CCC=C(C)C)(C)C1C=CC(=CC(=O)c1[nH]ccc1)C	5.5	Bad	5.1
12100	O1C(OC)(CCCCCCCCC(OC)=O)C(C)=C(C)C1=O	4.6	Bad	5.1
11716	O1C2C3C(CCC(C)(C(C2)C)C3=C)(CC=O)C1=O	3.8	Excellent	5.1
11543	O(C(=O)C(C(O)C(C=C(CC)C=C(C=Cc1ccccc1)C)C)C)C	3.4	OK	5.1
10547	Brc1cc(O)c(cc1O)CC=C(CCC=C(C)C)C	5.5	Excellent	5.1
9916	O(C)C1=CC(=O)C(O)=C(CCCCCCCCCCC)C1=O	5.2	Good	5.1
8618	O1OC(CC(CC)C1CC(O)=O)(CC)C=CCCC	4.7	Excellent	5.1
8488	Brc1c2c3c([nH]c2ccc1O)c(ncc3)C(N(C)C)CSC	3.6	Excellent	5.1
5414	OC1C2C3C(CCCC2(C)C)(C)C1(CC3O)C	4.0	Excellent	5.1
5027	Oc1ccc(cc1[N+](=O)[O-])CCNC(=O)CC(C)C	4.6	Excellent	5.1
3224	O1C2C(C(CC2O)C1CC=CCC)C=CCC=CCCCC(O)=O	5.3	Bad	5.1
2600	O1C(C2=C(C3(C(CC2)C2(C(CC3OC(=O)C)C3(C(CC2)C(CCC3)(CC)C)C)C)C)C1=O)C	5.2	Good	5.1
1490	o1cc(cc1CC(CCC=C(C=C)C)C)C(O)=O	5.6	Excellent	5.1
13342	OC1(CCC(C)(C1=C)c1ccc(cc1O)C)C	4.4	Excellent	5.1
12674	O(C(CCCC=CC#CCCC=CCC=CCC)C(O)=O)C	4.4	OK	5.1
12653	O1C2(C=CC(=CC=O)C)C(CC(O)CC12C)(C)C	4.2	Excellent	5.1
12582	Brc1cc(cc(Br)c1OCCCN(C)C)C(O)CNC(=O)CC	4.5	Poor	5.1
12042	OC(CC=CCC=CCC)C=CC=CCC=CCCCC(O)=O	4.6	Poor	5.1
9504	S(C)C1(N(C)C(=O)C(SC)(N(C)C1=O)CO)Cc1ccccc1	5.3	Good	5.1

9299	O=C1CN(C)C(=O)C1=C(O)CCCCCCCCCCCCCCC	4.2	OK	5.1
8960	Oc1ccc(O)cc1C=CC(OC)(CCC=C(C)C)C	4.6	Good	5.1
7587	O=C1C=CC(CC=CCCCCC)C1CC=CCCCC(OC)=O	7.0	OK	5.1
6245	O1C(CC2C3(C(CCC12CO)C1(C(CC3)C(CCC1)(C)C)C)C)C1=CC(OC1O)=O	4.7	Bad	5.1
4508	ClC(Cl)(Cl)C(C=C1N(C)C(=O)C(N(C)C1=O)=CC(C(Cl)(Cl)Cl)C)C	5.1	Excellent	5.1
4061	O1OC(CCC1(OC)CCCC=CC=CCC)C(C(O)=O)C	5.8	OK	5.1
2804	O1C(=O)C(CC1CO)C(O)CCCCC(C)C	4.9	Excellent	5.1
2250	O(C)c1c2c(C(=O)C=C(O)C2=O)c(OC)cc1OC	5.3	Excellent	5.1
1213	O=C1C=CC(CC=CCCCCC)C1CC=CCCCC(O)=O	6.2	Excellent	5.1
13600	O1OC(CC(CC)C1CC(OC)=O)(CC(CC)C=CCC)CC	4.8	OK	5.1
13581	OC(=O)CCCCC(=O)C=CC=CCC=CCCC	4.1	Good	5.1
13476	BrC1CCC(O)(C)C(CCC(O)(C=C)C)C1(CCC=C(C)C)C	4.4	Excellent	5.1
13360	O1C(OC(=O)C)C2C(=CCC3C4(C(CCC23C)C2(C(CC4)C(CCC2)(COC(=O)C)C)C)C)C1=O	3.7	Bad	5.1
11263	O1CCC2(CC(=O)C(CCC(=O)C)C2(C)C)C1=O	5.6	Excellent	5.1
11118	OC1CC(CC2C(CCC2(O)CC1=C)C)(C)C	4.2	Excellent	5.1
11082	O1OC(CC(CC)C1CC(OC)=O)(CC(CC)C=CCC)C	5.6	Excellent	5.1
5708	ClC(Cl)(Cl)C(CC1N(C)C(=O)C(N(C)C1=O)CC(C(Cl)Cl)C)C	5.8	Excellent	5.1
4221	O1C=C(OCc2ccccc2)C(=O)C=C1CO	4.7	Excellent	5.1
3804	ClC(Cl)(Cl)C(CC(N(C(=O)CC(C(Cl)(Cl)Cl)C)C)c1sccn1)C	5.4	OK	5.1
3554	O1C23C1C(O)CC(C)C2(C)C(C(=O)C)C(=O)CC3	5.1	OK	5.1
3364	ClC(Cl)(Cl)C(CC(N(C(=O)C=CCC(C(Cl)(Cl)Cl)C)C)C(OC)=O)C	6.1	Good	5.1
3198	O1C23C1C(O)CC(C)C2(C)C(C(=O)C)C(=O)CC3	3.6	Excellent	5.1
638	OC(CC=CCC=CCCCC(O)=O)C=CC=CCC=CCC	3.9	Bad	5.1
13579	OC(CCCCC(O)=O)C=CC=CCC=CCC=C	5.2	OK	5.1
12585	Oc1c(C)c(O)c(cc1C(=O)CCC=CC)C	4.1	Good	5.1
11952	O1CC(C2C(C(CC(OC(=O)C)C=C(CC2)C)=C)C1=O)=CC=CC(O)(C)C	4.7	Good	5.1
11408	OC=C(CCC=C(CCC=C(C)C)C)C=CO	4.2	OK	5.1
9652	OC(CC(O)c1ccccc1)c1ccccc1	4.3	OK	5.1
8753	Oc1c(C=O)c(C=CC=CC=CC)c(O)cc1CC=C(C)C	5.9	Good	5.1
5662	BrC1CC(O)C(OC1CC)CC(=O)CCC=CC#C	4.2	Good	5.1
5254	O(CC#CC#CCCCCCCCCC=C)CC(O)=O	4.9	Bad	5.1
3803	ClC(Cl)(Cl)C(CC(NC(=O)CC(C(Cl)(Cl)Cl)C)c1sccn1)C	5.1	Excellent	5.1
3615	ClC1=C2CCC3C4CCC(C(CC(=O)C(C(C)C)CC)C(O)=O)C4(CCC3C2(CCC1=O)C)C	5.6	Bad	5.1
3518	Oc1ccc(cc1)C(CCC(O)C(C)=C)(C=C)C	5.5	Excellent	5.1
3385	O1C(CCC2OC2(C)C)(C)C1CCC(=CC(OC)=O)C	5.5	OK	5.1
3090	O(C(=O)C=CC=1[N+](C)(C)[C-]=NC=1CC=C(C)C)C	4.8	Excellent	5.1
2330	O(C(=O)c1c2c(n(c1)C(OCC)=O)cccc2)CC	6.1	Excellent	5.1
1207	O=C(NN)CCCCCCCCCCCc1ccccc1	4.6	Bad	5.1
437	O1C(C(O)C=CCCCCCCCCC(O)=O)C1CC=CCC	5.4	Bad	5.1
392	BrC1=CC(O)(CC=CCCCCC)C(=CC=CCCCC(OC)=O)C1=O	4.5	Poor	5.1
13616	O1C23C1CCC(C)C2(C)C(C(=O)C)C(O)CC3	5.1	Excellent	5.1
11611	O1C(=O)C(CCC1(CCCCCCCCC)CO)C	5.1	Excellent	5.1
5458	Brc1c2c(NC(=O)C2(O)CC(=O)C)cc(Br)c1	5.6	Poor	5.1
14348	Oc1cc(O)cc(C(OC)=O)c1C(=O)CCCCCCC	2.9	Good	5.0
13601	OC1(CCC(C)(C1C)c1ccc(cc1O)C)C	4.6	Excellent	5.0
12092	O1C(O)C(C2C3(C(CCC12O)=CCCC3C)C)C	3.5	Good	5.0
11427	OC1(CCCCCCC)C(=O)c2c(NC1=O)cccc2	5.0	Excellent	5.0
10263	OC1(C2C(C=C(CC1)C(C)C)C(O)(CC2)C)C	4.4	Excellent	5.0
10103	O(C(=O)C(Cc1c2c([nH]c1)cccc2)C)CC	5.2	Excellent	5.0
8466	OC1C2CC(O)CCC2(C=2C(C3CCC(C(=CC(=O)CC(C)C)C)C3(CC=2)C)C1)C	5.1	Bad	5.0

5247	O=C(NCCc1c2c([nH]c1)cccc2)CC(C)C	3.5	Excellent	5.0
5094	O=[N+]([O-])CCCCCCCCCCC#CCCc1cccnc1	4.1	Bad	5.0
3613	ClC1=C2CCC3C4CCC(C(CC(=O)C(C(C)C)=C)C(O)=O)C4(CCC3C2(CCC1=O)C)C	4.5	Good	5.0
1749	OC(=O)CC(NC(=O)C=CC=CC=CC)c1ccccc1	5.4	Good	5.0
607	Oc1cc(ccc1C(CCCC(O)(C)C)C)C	4.5	Excellent	5.0
391	IC1=CC(O)(CC=CCCCCC)C(=CC=CCCCC(OC)=O)C1=O	6.3	Good	5.0
208	o1cc(cc1)CCCC(CC(=O)CC(CCCc1ccoc1)C)C	4.8	Poor	5.0
13580	OC(CC=CCCCCC(O)=O)C=CC=CCC=C	4.4	Poor	5.0
12933	BrC1C(C23CC(Cl)C(CC2(O)CC1O)(CC3)C)(C)C	4.7	Excellent	5.0
12696	OC(CC=CCC=CCC)C=CC=CCC=CCCCC(O)=O	3.4	Bad	5.0
12398	O1OC(CC(CC)C1CC(OC)=O)(CC(CC)C=CCC)CC	3.9	OK	5.0
5143	Brc1cc(cc(Br)c1OCCCNC(=O)C#N)CCN	4.5	Poor	5.0
4740	BrC=1C(O)C2(ON=C(C2)C(=O)N)C=C(Br)C=1OC	5.1	OK	5.0
3342	ClC1CCC(C)C(CC(=O)C)(C)C12OC(=O)CC2	5.5	Excellent	5.0
2706	OC(=O)CC(CCCC(CCC(=O)C(C)C)C)C	5.1	Excellent	5.0
2553	O(C(=O)C)CC=CC=CCC=CCCCC(OC)=O	3.9	Good	5.0
2489	BrC1CC(O)C(C)=C(CCC(O)(C=C)C)C1(C)C	4.9	Excellent	5.0
2243	BrC1CCC(=C)C2(O)CC(CCC12C)C(=CC=CC(O)(C)C)C	4.7	Bad	5.0
1226	OC(CC=CCCCC(O)=O)C=CC=CCC=CCC=CCC	5.0	OK	5.0
13577	OC(CC=CCCCCC(O)=O)C=CC=CCCC	4.5	Excellent	5.0
13309	Brc1cc[nH]c1C1=NC(=CNCC(C)C)C(OC)=C1	3.0	Excellent	5.0
12326	OC(CC(O)c1ccccc1)c1ccccc1	4.4	Excellent	5.0
9634	OC(CC(O)c1ccccc1)c1ccccc1	4.6	Poor	5.0
9302	O=C1CN(C)C(=O)C1=C(O)CCCCCCCCCCCCC	4.3	Poor	5.0
8147	OCC1C2(C(CC=C1CO)C(CCC2)(C)C)C	4.4	Excellent	5.0
6896	O1C(=Cc2c(c(O)c(OC)c(OC)c2)C1=O)C	5.1	Excellent	5.0
6695	OCC1C2(C(CC=C1CO)C(CCC2)(C)C)C	3.6	Excellent	5.0
5086	O=[N+]([O-])CCCCCCCCCCCCc1cccnc1	4.4	Excellent	5.0
3634	ClC(Cl)(Cl)C(CC1N(C)C(=O)C(N(C)C1=O)CC(C(Cl)(Cl)Cl)C)C	4.5	OK	5.0
2339	OC(CCCCCCCC(O)=O)C=CC=CCC=CCC	4.8	Bad	5.0
187	O=C1N(CCCCCC)C(=O)N(c2ncn(c12)C)C	5.4	Good	5.0
14150	O(C)C1=CC(=O)C=C(CCCCCCCCCCC)C1=O	4.2	Bad	5.0
13622	BrC=1C(O)C2(ON=C(C2)C(OC)=O)C=C(Br)C=1OC	4.2	Poor	5.0
13142	O1C(CCCC1=O)C=CC=CC=CC(O)CC=CCCCCC	4.7	Poor	5.0
10095	o1c(CCCCCCCCC(O)=O)c(cc1CCCCC)C	4.7	Bad	5.0
8938	O1C2CC3C4(C(CCC3(C3CC=C(C(C23C)C1=O)C(=O)C)C)C(CCC4)(C)C)CO	5.3	Bad	5.0
7916	O1C2=C(CC3C4(C(OC(CC4)C(O)(C)C)CCC13C)C)C(=O)C=C(C(C(=O)C(CC)C)C)C2=O	4.8	Poor	5.0
6457	Brc1cc(cc(Br)c1OCCCN(C)C)CCNC(=O)C	4.0	Good	5.0
1591	O1C(C=CC=CC=CCCCCCCC)C(O)CC=CCCC1=O	4.6	OK	5.0
1496	O(C(=O)C=CC=CC(CC)CO)C1C=CC2=CC(=O)C(O)CC2(C)C1C	5.1	Poor	5.0
14151	O(C(=O)C)C1C(C=CC2(C(C(CC2)C(O)(C)C)C(OC(=O)C)CC(=CC1OC(=O)C)C)C)C	5.9	Poor	5.0
11257	OC1(CC2(C(=CCC3C4CCC(C(C=CC(C(C)C)CC)C)C4(CCC23)C)C1=O)C)C(OC)=O	4.4	Poor	5.0
4713	OC(CCCCCCCC(O)=O)C=CC=CCC=CCC	4.4	Poor	5.0
2238	O1C2(C34OOC(CC3)(C=C4CC(C)C2(CC1C1(OOC(CC1)C(C(OC)=O)C)C)C)C)C	4.7	Good	4.9
13528	OC(C(CCC=C(CC(=O)CC(C)C)C)=C)CCC(=CCO)C	5.8	Excellent	4.9
12212	OC(CC=CCC=CCCCCC)C#CCCCC(O)=O	4.9	OK	4.9
11780	OC1C2CC(O)CCC2(C=2C(C3CCC(C(=CC(=O)CC(C)C)C)C3(CC=2)C)C1)C	3.6	Poor	4.9

11171	O(C(=O)C)C1C2C(CCCC2(C2CC=C(CC2(C1)C)C1CC(=O)C(=CC1O)C)C)(C)C	5.1	Bad	4.9
9308	O=C1C(=C(O)CCCCCCCCCCC)C(=O)N(C)C1C	4.4	Bad	4.9
8049	Brc1cc2c(n(cc2OC(=O)C)C(=O)C)cc1	4.5	Excellent	4.9
6167	O1C2(CCC1C1(CCC3OC(C)(C)C(=O)CCC13C)C)C1(C(OC(C)(C)C(=O)CC1)C CC2C)C	5.6	OK	4.9
4203	Brc1[nH]c(cc1)C1=NC(=CNCCC)C(OC)=C1	4.3	Good	4.9
3832	O1C2OC3OC2(CC1=O)C(OC(=O)C)CC1C3CCC2C3(C(CCC12C)C(CCC3)(C) C)C			4.9
2803	O1C(=O)C(CC1CO)C(O)CCCCC(C)C	5.7	OK	4.9
13614	O1C23C1C(O)CC(C)C2(C)C(C(=O)C)C(O)CC3	3.6	Excellent	4.9
13582	OC(CCCCC(O)=O)C=CC=CCC=CCCC	5.7	Bad	4.9
12205	OC(=O)C12CC(C(CCC1C)C2CC(O)=O)(C)C			4.9
12195	O1C(=O)C(CCC1(CCCCCCCCC)CO)C	3.9	Excellent	4.9
9758	OC(CC(O)c1ccccc1)c1ccccc1	3.5	Good	4.9
4498	O1C(C)(C1C=CC(OC)(CO)C)C1CC(C(C)=C)C(CC1)(C=C)C	3.9	Bad	4.9
4285	ClC(CCCC(CCCC(C)C)C)(C(OC)=O)C(O)=O	5.9	Excellent	4.9
4202	Brc1[nH]c(cc1)C1=NC(=CNCC)C(OC)=C1	4.3	Excellent	4.9
4119	O(C(=O)C=CC=1[N+](C)(C)[C-]=NC=1CC=C(C)C)C	4.3	Excellent	4.9
3807	ClC(Cl)C(CC(N(C(=O)CC(C(Cl)Cl)C)C)c1sccn1)C	5.0	Excellent	4.9
14239	Ic1c(OC(=O)C)cc(OC(=O)C)cc1OC(=O)C	3.7	Good	4.9
12710	OC(CC=CCCCCCCCC(O)=O)C=CC=CCC	4.1	Poor	4.9
9281	O(C)c1ccc(cc1)C=C1N=C(N)N(C)C1=O	4.4	Excellent	4.9
7612	ON=CCCCCCCCCCCCc1cccnc1	3.7	Bad	4.9
6724	Oc1cc(ccc1C(CC(O)CC(C)C)C)C=O	4.6	Excellent	4.9
4606	Brc1c2c(NC(=O)C2(O)CC(=O)C)cc(Br)c1	4.0	Excellent	4.9
4277	ClCC(=O)C1(C2C(CC(C)(C)C2O)C=C1CO)C	5.1	Excellent	4.9
4169	O1C2=C(CC3C4(C(OC(CC4)C(O)(C)C)CCC13C)C)C(=O)C=C(C(C(=O)C(CC) C)C)C2=O	4.1	Poor	4.9
3532	O1C2CC(OC2CC=CC#C)C1CC(O)C(O)C=C	3.0	OK	4.9
13307	Brc1[nH]c(cc1)C1=NC(=CN)C(OC)=C1	3.9	Excellent	4.9
13298	S(=O)(=O)(CCC(OC)(OC)C)c1ccccc1	3.9	Excellent	4.9
10176	S(O)(=O)(=O)n1cccc1CCCCCCC	5.2	Excellent	4.9
8244	O(C)C1=C[C-]([NH+](C=C1)CC(O)=O)CCCCCCCC=C	5.6	Bad	4.9
7030	O1C(CCCC1=O)(CCCCCCCCCCC)CO	3.6	OK	4.9
6395	ClC(Cl)(Cl)C(CC1(O)N(C)C(=O)C(N(C)C1=O)CC(C(Cl)(Cl)Cl)C)C	3.5	Excellent	4.9
5253	O(CC#CC#CCCCCCCCC)CC(O)=O	5.5	Bad	4.9
4748	OC(CC=CCCCCCCCC(O)=O)C=CC=CCC	5.6	Bad	4.9
3806	ClC(Cl)(Cl)C(CC(N(C(=O)CC(C(Cl)Cl)C)C)c1sccn1)C	5.1	Excellent	4.9
1825	OC(=O)CCCCCCCC(=O)C=CC=CCC=CCC	4.5	Poor	4.9
717	O(CC(O)CO)C=CC#CC=CCCCCCCCC(C)C	5.2	Bad	4.9
6406	OC(CCCCCCCC(O)=O)C=CC=CCC=CCC	4.5	Bad	4.9
5554	O1C(C=CC=CC=CC(=O)C)=C(C)C(OC)=CC1=O	5.2	Bad	4.9
2462	Brc1cc(cc(Br)c1OCCCN(C)C)C=CC(O)=O	4.1	Bad	4.9
691	s1ccnc1C(=O)c1cc(OC)c(O)cc1	5.8	Poor	4.9
6330	O1OC(CC(CC)C1CC(OC)=O)(CC)C=CCC	4.4	Excellent	4.8
13615	O1C23C1C(O)CC(C)C2(C)C(C(=O)C)C(O)CC3	5.0	Excellent	4.8
13578	OC(=O)CCCCC(=O)C=CC=CCC=CCC=C	4.8	Bad	4.8
13260	OC=C(CCC1C(CCCC1=C)(C)C)C=CO	4.9	Excellent	4.8
13007	OC(CCCC(O)=O)C=CC=CCC=CCC=CCC=CCC	5.2	Poor	4.8
12861	OC1(CCC=C(CCC(C=C1)C(O)(C)C)C)C	5.3	Excellent	4.8
12168	OC1CC2C(C3CC=C(C(=O)C)C(C=O)C13C)(CCC1C(CCCC12C)(C)C)C	5.3	Excellent	4.8
10970	O(C)C1=C(OC)C(=O)c2c(C1=O)c(OC)cc(OC)c2	4.8	Excellent	4.8

7867	O=C1C=CC(C=CC(O)CCCCC)C1CC=CCCCC(OC)=O	5.7	OK	4.8
2929	O1C2(CCC1=O)C(CC(=O)C)(C)C(CCC2O)C	5.2	Excellent	4.8
904	S(C)C1(N(C)C(=O)C(SC)N(C)C1=O)Cc1ccc(OCC(OC)=CC)cc1	4.8	Good	4.8
13958	O(C(C(C(=O)CC)C)C(=CC(CCC)C)C)C(=O)CC	4.3	Excellent	4.8
12430	O1C(OC(=O)C)C2C(=CCC3C4(C(CCC23C)C2(C(CC4)C(CCC2)(C)C)COC(=O)C)C)C1=O	4.6	Bad	4.8
11059	o1c(CCCCC)c(C)c(C)c1CCC(O)=O	4.5	Excellent	4.8
10798	O(C(=O)C)C1CC2=CCC3C4CCC(C(C=CC(OC)=O)C)C4(CCC3C2(CC1)C)C	3.8	Poor	4.8
10175	S(O)(=O)(=O)n1cccc1CCCCCCCC	3.7	Excellent	4.8
8683	O1CC2C3C(C=O)C1(CO)C2(CCC3C(C)C)C			4.8
6655	Oc1cc(ccc1C(CC(OC(=O)C)CC(C)C)C)C=O	4.7	Poor	4.8
6535	O(CC(O)CO)C=CC#CCCCCCCC(C)C	4.4	Bad	4.8
11259	OC1(CC2(C(=CCC3C4CCC(C(C=CC(C(C)C)CC)C)C4(CCC23)C)C1=O)C)C(OC)=O	5.5	Bad	4.8
8475	OC(C(O)CC=CC#C)CC=CCC=CCC	5.4	Excellent	4.8
6531	O(CC(O)CO)C=CC#CC=CCCCCC(C)C	5.0	Bad	4.8
3724	O(C(=O)C)c1cc2c([nH]cc2)cc1OC(=O)C	3.8	Excellent	4.8
1583	O1C(C=CC=CC=CCCCCC)C(O)CC=CCCCC1=O	4.5	OK	4.8
8355	O(CC#CC#CCCCCCC=C)CC(O)=O	5.5	Excellent	4.8
6532	O(CC(O)CO)C=CC#CC=CCCCCCC(CC)C	4.7	Bad	4.8
2727	O1C(C=CC=CC=CCCCCC)C(O)CC=CCCCC1=O	4.4	Poor	4.8
723	O(CC(O)CO)C=CC#CC=CCCCCCCC(C)C	4.7	Bad	4.8
7013	BrC1=CC(O)(C=C(Br)C1=O)CC(OCC)=O	5.4	Good	4.8
5978	BrC1CC(OCC1(O)C)C(O)(C=CCl)C	5.7	OK	4.8
4484	OC(CC=CCCCCCCCC(O)=O)C=CC=CCC	4.9	OK	4.8
716	O(CC(O)CO)C=CC#CC=CCCCCCCCC	4.8	Bad	4.8
11863	O(C(CC=CCCC(O)=O)C=C(CCCCCCC)C)C	5.0	Bad	4.7
11313	O1C(OCC)(CC(OCC)=O)C(CC1(CC)C=CCCC)CC	3.2	Good	4.7
10177	S(O)(=O)(=O)n1cccc1CCCCCC	3.8	Excellent	4.7
5263	BrC=1C(O)C2(ON=C(C2)C(OC)=O)C=C(Br)C=1OC	4.5	Poor	4.7
3805	ClC(Cl)(Cl)C(CC(NC(=O)CC(C(Cl)Cl)C)c1sccn1)C	5.2	OK	4.7
269	S(OC1CC2CCC3C4CCC(C(CCC(C(C)C)=C)C)C4(CCC3C2(CC1)C)C)(O)(=O)=O	4.4	Bad	4.7
6536	O(C(=O)CCCCCCC=CC(O)C#C)C	3.7	Good	4.7
6530	O(CC(O)CO)C=CC#CC=CCCCCCC	5.0	Bad	4.7
5594	ClC(Cl)C(CCC(=O)C(CCOC(=O)C)C)=CCl	4.6	Bad	4.7
1592	O1C(C=CC=CC=CCCCCCC)C(O)CC=CCCC1=O	4.3	Poor	4.7
9418	OC(CCCCCCCC(O)=O)C=CC=CCC=CCC	5.0	Bad	4.7
9135	O(C(=O)C)C1CC2C(C3CC(O)C(C(=O)C)C(C=O)C13C)(CCC1C(CCCC12C)(C)C)C	5.5	Good	4.7
8497	O1C(CCCC1=O)(CCCCCCCCCC)CO	3.6	Excellent	4.7
8351	O(CC#CC#CCCCCCC=C)CC(O)=O	3.9	Poor	4.7
6692	O1C2C(CC3C(C)(C(OC(=O)c4ccccc4)CCC(=C2)C)C(OC(=O)C)CC=C3C)=C(C)C1=O			4.7
4968	Brc1cc(cc(Br)c1OC)C[C-]([N+](C)(C)C)C(=O)NCCc1nc[nH]c1	5.6	Bad	4.7
4607	Brc1c2c(NC(=O)C2(O)CCCl)cc(Br)c1	5.2	Excellent	4.7
1967	S1CCc2c(c(SSC)c(OC)c(OC)c2N(C)C)C1=O	5.5	Excellent	4.7
714	O(CC(O)CO)C=CC#CC=CCCCCCCC	4.9	Bad	4.7
7073	OC(CCCCCCCCCCCCC)C(O)=O	4.4	Bad	4.7
6756	Brc1c(OC(=O)C)c(Br)cc(OC(=O)C)c1Br	4.5	Excellent	4.7
2008	BrC1=CC(O)(C=C(Br)C1(OCCCCC)OC)CC(=O)N	4.9	Excellent	4.7
1495	O(C(=O)C=CC=CC(O)(CC)C)C1C=CC2=CC(=O)C(O)CC2(C)C1C	5.8	Bad	4.7

1354	Brc1c2c([nH]c(S(=O)C)c2SC)cc(Br)c1	3.7	Excellent	4.7
13576	OC(CCCCCCC)C=CCCCCCC(O)=O	4.7	Bad	4.7
11434	[Si](O[Si](O[Si](O[Si](OC(=O)C)(C)C)(C)C)(C)C)(C(=O)C)(C)C	5.4	Excellent	4.7
7074	OC(CCCCCCCCCCCCC)C(O)=O	4.0	Bad	4.7
12096	O=Cc1[nH]c(cc1)CCCCCC=CCCCCCC#N	4.6	OK	4.6
12032	O1C(=O)C(CCC1(CCCCCCCCC)CO)C	4.7	Poor	4.6
11980	O1C(CCCCC)C(O)CC1C(O)CCCCCCCC=C	3.7	Poor	4.6
11169	O(C(=O)C)C1C2C(CCCC2(C2CC=C(CC2(C1)C)c1cc(O)c(cc1)C)C)(C)C	4.1	Bad	4.6
7548	OC1(CCC(C(O)(CCC=C(C)C)C)C1C)C	4.0	Excellent	4.6
6328	O1OC(CC(CC)C1CC(OCC)=O)(CCCC)CC	5.3	Good	4.6
5593	ClC(Cl)C(CCC(=O)C(CCOC(=O)C)C)=CCl	5.1	OK	4.6
1338	O(C(=O)C)c1c(OC(=O)C)cc(cc1OC(=O)C)COC	4.0	Good	4.6
454	O1C(C(O)C=CCCCCCCCC(O)=O)C1CC=CCC	5.1	Poor	4.6
14100	OC(=O)CCCCCCCC=CCC(=O)C=CC=CCC	4.1	Bad	4.6
12049	Brc1cc(cc(Br)c1OC(=O)C)COC(=O)C	3.6	Good	4.6
6232	O1C(C(O)CC=C(C)C)(C)C1CCc1ccoc1	4.8	Excellent	4.6
5099	OC1C2C3CCC1(C)C3(CCCC2(CO)C)C			4.6
2007	BrC1=CC(O)(C=C(Br)C1(OCCCC)OC)CC(=O)N	5.6	Good	4.6
1311	OC(CC=CCCCCCCCC(O)=O)C=CC=CCC	5.0	Bad	4.6
720	O(CC(O)CO)C=CC#CC=CCCCCCC(C)C	4.8	Bad	4.6
11880	O(C(=O)C)C1CC2C(C)(C(C=O)C1C(=O)C)C(O)CC1C2(CCC2C(CCCC12C)(C)C)C	4.3	OK	4.6
8106	O(C(CCCC=CCCCCCC(C)C)C(O)=O)C	4.6	Excellent	4.6
6332	O(C(=O)CC(O)C(CC(O)(CCCC)CC)=CC)CC	3.8	Good	4.6
4236	O1C(C=CC=CC=CCC=CCC)C(O)CC=CCCCC1=O	4.2	Poor	4.6
2373	O(C(=O)C=CC=CC(CC(O)=O)C)C1C=CC2=CC(=O)C(O)CC2(C)C1C	5.2	Bad	4.6
12571	O1C(C)(C)C(CC12OC1C3C45C(C6C(C(OC4OC23C)C5)C2(C(CC(OC(=O)C)CC2)CC6)C)C1)C	3.7	Bad	4.6
11319	O(C(=O)CC(O)C(CC(O)(CC)C=CCCC)CC)CC	4.3	Good	4.6
11315	O(C(=O)CC(O)C(CC(O)(CC)C=CCCC)CC)CC	4.4	Excellent	4.6
8067	S(C(=O)C)CCC(=O)C=CCCCCCC	5.7	Poor	4.6
7513	Brc1c(O)c(cc(Br)c1OC)CC(=O)N	5.0	Excellent	4.6
4457	O(C(=O)CCCCCCCCCCCC=O)C	4.3	Excellent	4.6
12581	Brc1cc(cc(Br)c1OCCCN(C)C)C(O)CN	4.3	Poor	4.5
4476	Brc1c(CCNCC(O)C)c(Br)cc(Br)c1OC	3.6	OK	4.5
9674	OC1(C)C(CCC(O)(C=C)C)C(=CCC1C)C	4.2	Excellent	4.5
9637	OCC1=CC2C(CCO)C1(CCCC2(C)C)C	4.6	Poor	4.5
7162	O1C(CCCC(C)C)(C)C2(O)C3(CC=C4C(=CCC5C(C)(C)C(O)CCC45C)C3(CC2)C)C1=O	3.7	Good	4.5
5979	BrC1CC(OCC1(O)C)C(O)(C=CCl)C	4.2	Poor	4.5
4504	ClC(Cl)(Cl)C(CC=CC=CC(=O)NC(C(C)C)c1sccn1)C	5.0	Bad	4.5
2952	O1C(C(CC)C)=C(C)C(=O)C=C1CC(O)C	6.4	Excellent	4.5
2338	OC(CC=CCCCCCCCC(O)=O)C=CC=CCC	4.4	Good	4.5
1355	Brc1c2c([nH]c(SC)c2S(=O)C)cc(Br)c1	4.3	Good	4.5
11521	OC(CCCCCCC)CC=CCCC(O)=O	4.4	Excellent	4.5
9727	Brc1c(O)c(O)cc(COCC)c1Br	4.5	Excellent	4.5
8474	OC(C(O)CC=CC#C)CC=CCC=CCC	5.1	Excellent	4.5
8069	S(C(=O)C)CCC(=O)CCCCCCCC	4.6	Good	4.5
6533	O(CC(O)CO)C=CC#CCCCCCCCC	4.2	Bad	4.5
4833	O(C(=O)C)C1C2C(CCCC2(C2CCC(O)(CC2(C1)C)C1CC(=O)C(=CC1O)C)C)(C)C	4.8	Bad	4.5
14422	Brc1cc2[nH]cc(c2cc1)CSCCN	3.9	Excellent	4.4

13865	OC(CCCCCCCC(O)=O)C=CC=CCC=CCC	4.3	Bad	4.4
9673	OC1(C)C(CCC(O)(C=C)C)C(=CCC1C)C	4.1	Excellent	4.4
5977	BrC1CC(OCC1(O)C)C(O)(C=CBr)C	4.7	Excellent	4.4
3313	BrC(C(=O)CCC(OC(=O)C)CC)=C(Br)Br	5.1	Excellent	4.4
3286	O1C(CC2C(CCC3C4(C(CCC23C)C(CCC4)(C)C)C)C1OC(=O)C)C1=CC(OC1)=O	3.7	Bad	4.4
2904	Brc1cc(cc(Br)c1O)CC(=O)C(O)=O	4.6	Excellent	4.4
2726	O1C(C=CC=CC=CCC=CCC)C(O)CC=CCCCC1=O	5.1	Poor	4.4
1601	O1C(C2C3(CCC4=C(CCC5C(C)(C)C(O)CCC45C)C3(CC2=O)C)C1=O)(CC(=O)CC(C)C)C	4.4	Poor	4.4
11520	O(C(CCCCCCC)CC=CCCC(O)=O)C	5.6	Good	4.4
11358	BrC=1C(=O)C(Br)=C(Br)OC=1C(=O)CC	5.4	Excellent	4.4
9574	Brc1c(O)c(O)cc(COCCC)c1Br	5.5	Excellent	4.4
6745	Clc1cc([N+]([O-])=NC(=O)N)cc(Cl)c1OC	5.0	Bad	4.4
6331	O(C(=O)CC(O)C(CC(O)(CCCC)CC)CC)CC	5.0	Good	4.4
722	O(CC(O)CO)C=CC#CC=CCCCCCCCC	4.7	Bad	4.4
230	[As](CC1OC(OCC2OC(OC2)(C)C)C2OC(OC12)(C)C)(C)C	4.5	Excellent	4.4
9511	BrC=1C(O)C(O)(C=C(Br)C=1OC)CC#N	5.3	Excellent	4.4
8732	Brc1cc2c([nH]cc2CCN)cc1Br	4.2	Excellent	4.4
7012	BrC1=CC(O)(C=C(Br)C1(OC)OC)CC(=O)N	4.6	OK	4.4
6534	O(CC(O)CO)C=CC#CCCCCCC(CC)C	4.0	Bad	4.4
5787	O(C(OC)CCCCCCCCCC(OC)=O)C	4.8	OK	4.4
434	Brc1cc(cc(Br)c1O)COC(=O)C	4.6	Excellent	4.4
14475	O(C(=O)C)C1CC2C3(C(CCC2(C2=CC(=O)C(C(=O)C)=C(C(O)=O)C12C)C)C(CCC3)(CC)C)C	6.6	Poor	4.3
7015	Brc1cc(cc(Br)c1O)CC(OCC)=O	5.0	Good	4.3
715	O(CC(O)CO)C=CC#CC=CCCCCCCCC	5.1	Bad	4.3
231	[As](=O)(CC1OC(OCC2OC(OC2)(C)C)C2OC(OC12)(C)C)(C)C	5.2	Excellent	4.3
11105	BrC1CC(O)C(=CC1(C)C)C(O)CCl	3.7	Excellent	4.3
8461	Brc1c(O)c(cc(Br)c1OC)CC(O)=O	5.7	Excellent	4.3
7791	BrC=1C(O)C(O)(C=C(Br)C=1OC)CC#N	4.9	Excellent	4.3
7527	BrC=1C(O)C(O)(C=C(Br)C=1OC)CC#N	4.3	Excellent	4.3
2394	ClC(Cl)C(CC1NC(=O)C(C)(C)C1O)C	5.6	Excellent	4.3
927	O(C(=O)C=CC=CC(CCO)C)C1C=CC2=CC(=O)C(O)CC2(C)C1C	4.9	Bad	4.3
721	O(CC(O)CO)C=CC#CC=CCCCCCCCC	5.7	Bad	4.3
11748	O(C(=O)CCCCC(=O)CCCCC)CC	3.4	Excellent	4.3
11109	OC(CCCCCCCCCCCC)C(O)=O	4.2	Good	4.3
11405	ClC=CCCCC(O)CC(O)CCCCC	5.1	Poor	4.3
5510	S(C(SC)=NC=Cc1ccc(O)cc1)C	4.1	Excellent	4.3
11881	O1CC2C(C1(O)C)C(OC(=O)C)CC1C2(C)C(=O)CC2C1(CCC1C(CCCC12C)(C)C)C	6.1	Good	4.3
1567	O1C(CCCC=CCCC(=O)CC(O)CC=CC=CC(O)CC=CC=CC1=O)C	5.2	OK	4.3
1492	O(C(=O)C=CC=CC(CC)C)C1C=CC2=CC(=O)C(O)(CC2(C)C1C)C(CO)=C	4.6	Bad	4.3
8677	OCC1C2C=C(CO)C1(CCC2C(C)C)C	4.9	Excellent	4.2
1969	S1CCc2c(c(SSC)c(OC)c(OC)c2N(C)C)C1(OC)C(=O)C	3.8	OK	4.2
812	S=C=NC=CCCCCCCCCCC=O	4.7	Poor	4.2
8767	BrC1=CC(O)(C=C(Br)C1(OCC)OC)CC(=O)N	5.7	Excellent	4.2
7361	OC(CCCCCCCCCCCC)C(O)=O	4.4	Poor	4.2
1970	S1CCc2c(C1OC)c(SOC)cc(O)c2N(C)C	5.6	Excellent	4.2
13201	O(C(=O)CCCCCCCCCC=O)CC	5.5	Poor	4.2
9949	OC1CCC2(C(CCC3(C2CC=C2C4CC(CCC4(CCC23C)C)(C(O)=O)C)C)C1(C)C)C	5.4	Bad	4.2

7211	Brc1c(COC)c(Br)c(Br)c(O)c1O	4.4	Excellent	4.2
5864	ClC1=CC2(OC(O)C(NC(=O)C=CC(=CC(CCCCCC)C)C)C2)C=C(Cl)C1=O	4.8	Bad	4.2
3768	Brc1cc(cc(Br)c1OCCCN)CCN	4.3	Poor	4.2
1971	S1CCc2c(c(SSC)c(OC)c(OC)c2N(C)C)C1OC	3.9	Excellent	4.2
10901	Brc1cc(Br)cc(C(OCC)=O)c1O	4.9	Excellent	4.2
10932	OC(=O)CCCCCCCCCCCC=O	5.0	Poor	4.1
9149	BrC(C(O)CC(Cl)C(O)(C=CBr)C)(CCl)C	5.6	Excellent	4.1
5511	S(C(SC)=NC=Cc1ccc(O)cc1)C	4.9	Poor	4.1
7484	O(C(=O)C)C1CCC2(C(CCC3(C2CC(=O)C3=C(C=CC=C(C=CC=O)C)C)C)C1(C(OC)=O)C)C	5.2	Bad	4.0
8776	BrC(C(O)CC=C(C(O)CBr)C)(CCl)C	4.4	Excellent	4.0
649	BrC(Br)C(O)CC(OC(=O)C)CCC	5.0	Excellent	4.0
11152	O1C(CCC1(C)C)C1(OC(=O)C23C1CCC2(C=1C(=CC3)C2(C(CC=1)C(C)(C)C(O)CC2)C)C)C	4.1	Bad	4.0
12050	Brc1cc(cc(Br)c1OC(=O)C)C=O	4.3	Excellent	3.9
9228	O1C2(CCC1C1(CCC3OC(C)(C)C(=O)CCC13C)C)C1(C(OC(C)(C)C(O)CC1)CCC2C)C	4.8	Poor	3.9
8777	BrCC(Cl)(C(O)CC=C(C(O)CBr)C)C	4.8	Excellent	3.9
14469	O(C(=O)C)C1CC2C(C3CC(O)C(C(=O)C)C(C=O)C13C)(CCC1C(CCCC12C)(CC)C)C	5.7	Good	3.9
4299	S(C)c1c(OC)c(O)cc(CCN)c1SC	3.8	Excellent	3.9
876	O1C(CCC1(C)C)C1(OC(=O)C23CC(=O)C4C(=CCC5C(C)(C)C(O)CCC45C)C2(CCC13O)C)C	4.7	Poor	3.9
2114	O1C(C)(C)C1CCC1(C=C(CCC1O)C=CC=CC1=CC(CCC2OC2(C)C)(C)C(O)CC1)C	4.4	Bad	3.9
11887	Brc1c(CO)c(Br)c(Br)c(O)c1OC	4.5	Excellent	3.8
2111	O1C(C)(C)C1CCC1(C=C(CCC1O)C=CC=CC1=CC(CCC2OC2(C)C)(C)C(O)CC1)C	4.7	Bad	3.8
8288	O1C(C2=C(C3(C(CC2)C2(C(CC3O)C3(C(CC2)C(CC)(C)C(OC(=O)C)CC3)C)C)C)C1=O)C	4.6	Bad	3.8
4011	O(C(=O)C=CC=CC(CC)C)C1C=CC2=CC(=O)C(O)(CC2(C)C1C)C(C=O)=C	4.0	Poor	3.7
8287	O1C(C2=C(C3(C(CC2)C2(C(CC3O)C3(C(CC2)C(CC)(C)C(OC(=O)CC)CC3)C)C)C)C1=O)C	4.9	Bad	3.7
13638	O1C(C)C(O)C(OC(=O)C)C(O)C1OC1CC2=CCC3C4CCC(C=C)C4(CCC3C2(CC1)C)C	4.9	Bad	3.5
2374	O(C(=O)C=CC=CC(CC)C)C1C=CC2=CC(=O)C(CC2(C)C1C)C(C)=C	4.0	Poor	3.5
3854	Oc1ccc(cc1)C=CNC1=C[C-]2[NH+](CCc3c2c(n(c3)C)C1=O)C	3.7	Poor	3.5
2375	O(C(=O)C=CC=CC(CC)C)C1C=CC2=CC(=O)C(CC2(C)C1C)=C(C)C	5.3	Bad	3.4
7970	O1C(O)(C=CC2C3(C(OC(C)(C)C(=O)CC3)CCC2(O)C)C)C(CCC2C(CCCC12C)(C)C)C	4.0	Poor	3.4
9390	OC1CCC2(C(CCC3(C2CC=C2C4CC(CCC4(CCC23C)C(O)=O)(C)C)C)C1(C)C)C	4.6	Bad	3.1
1845	O1CC2C(C1(OC)C)C(OC(=O)C)CC1C2(C)C(=O)CC2C1(CCC1C(CCCC12C)(CC)C)C	4.7	Bad	3.0
4378	OC1C2CC=C3C4CCC(C(CC5=NCCC5=C(C)C)C)C4(CCC3C2(CCC1NC(=O)C)C)C	5.3	Bad	2.9
5929	O1C(C)C(OC(=O)C)C(O)C(O)C1OC1CC2=CCC3C4CCC(C=C)C4(CCC3C2(CC1)C)C	4.6	Bad	2.9
12287	OC1CCC2(C(CCC3(C2CC=C2C4C(C)C(CCC4(CCC23C)C(O)=O)C)C)C1(C)C)C	4.9	Poor	2.6
12063	OC1CCC2(C(CCC3(C2CC=C2C4C(C)C(CCC4(CCC23C)C(O)=O)C)C)C1(C)C)C	4.3	Bad	2.5

9003	OC1CCC2(C3C(CCC2C1(C)C)(C)C1(C(C2CC(CCC2(CC1)C)(C(O)=O)C)=CC3=O)C)C	5.1	Bad	2.3
7821	O(C(=O)C=CC(=CC(CC)C)C)C1C=CC2=CC(=O)C(O)(CC2(C)C1C)C(COC(=O)C)=C	4.2	Bad	2.0
8286	O1C(C2=C(C3(C(CC2)C2(C(CC3O)C3(C(CC2)C(CC)(C)C(OC(=O)CCC)CC3)C)C)C)C1=O)C	4.0	Bad	1.9
6687	O1C2(CC(=O)C(=CC=CC(=CC=CC=C(C=CC=O)C)C)C)C(CC(O)CC12C)(C)C	6.1	Bad	1.8
7720	O1C(C)(C2C(OC)C(OC(=O)C=CC=CC=CC=CC(OC)=O)CCC23OC3)C1CC=C(C)C	4.2	Bad	1.7
10633	O(C(=O)C)c1ccc(cc1)C=CC=CC=CC=CC(=O)C	4.0	Bad	1.7
10381	O1C(C)(C2C(OC)C(OC(=O)C=CC=CC=CC=CC(OC)=O)CCC23OC3)C1CC=C(C)C	4.8	Bad	1.5
4262	Brc1cc[nH]c1C=CC=CC=CC=CC=1OC(=O)C(C)=C(O)C=1	5.2	Bad	1.5
6768	O1C(C)(C2C(OC)C(OC(=O)C=CC=CC=CC=CC(O)=O)CCC23OC3)C1CC=C(C)C	5.8	Bad	1.3
13322	O1C(C)(C)C(CC12OC1C(C2(O)C)C2(CC(=O)C3C(C2C1)CCC1CC(O)CCC13C)C)C	4.8	Poor	1.1
4263	Brc1c([nH]cc1Br)C=CC=CC=CC=CC=1OC(=O)C(C)=C(O)C=1	4.6	Bad	0.4
2760	[As](=O)(CC1OC(OC)C2OC(OC12)(C)C)(C)C	5.3	Poor	0.3

[a] Were the QSAR values are missing the alignment calculation failed. [b] From QSAR calculations. [c] From Docking calculations.

References

1. Floresta. G.; Pistarà. V.; Amata. E.; Dichiara. M.; Marrazzo. A.; Prezzavento. O.; Rescifina. A.. Adipocyte fatty acid binding protein 4 (FABP4) inhibitors. A comprehensive systematic review. *Eur J Med Chem* **2017**. *138*. 854–873.
